# Defining Aldol Chemoselectivity in the Presence of Henry Nucleophiles (Nitroalkanes)

**DOI:** 10.3390/molecules30244688

**Published:** 2025-12-07

**Authors:** Kritika B. Dwivedi, Patrick Knäbe, Nilesh N. Shitole, Aida H. Lakew, Ruslan Levochkin, Luis Paredes-Soler, Sofiia-Stefaniia Zhylinska, Diana Kochubei, Gabriela Guillena, Rafael Chinchilla, Diego A. Alonso, Thomas C. Nugent

**Affiliations:** 1 School of Science, Constructor University, Campus Ring 1, 28759 Bremen, Germany; 2 Department of Organic Chemistry, Institute of Organic Synthesis (ISO), University of Alicante, P.O. Box 99, 03080 Alicante, Spain

**Keywords:** chemoselectivity, organocatalysis, in-water reactions, aldol reaction, Henry reaction, nitroaldol reaction

## Abstract

This study evaluates the feasibility of achieving chemoselective aldol reactions over competing Henry reactions and employs competition experiments to establish proof of concept. A typical reaction involved using in-water reaction conditions where a concentrated organic layer containing an aldol nucleophile (1.5 equiv), a Henry nucleophile (1.5 equiv), an aldehyde electrophile (1.0 equiv), and a proline-based amino acid catalyst (2.5 mol%) constituted one phase, while the second phase was water (15 equiv). Highly enantioenriched aldol products were formed in practical yields, and a variety of Henry nucleophiles (nitroalkanes, allylic nitro compounds, and ethyl nitroacetate) were tolerated. This systematic examination of nitro compounds (pK_a_ 5.5–10.0) established a pK_a_ of ≈7.0 as the critical threshold at which nitronate formation results in Henry product formation under catalysis with **1**. Reactions alternatively performed in MeOH/H_2_O (3:2 equiv) solvent combinations, at times, provided improved chemoselectivity or product *dr* over the use of water (15 equiv) alone but required longer reaction times to produce similar yields. Reactions constrained by solubility were investigated using mechanochemical methods, but these conditions failed to deliver practical yields of either competition product. In summary, defining this category of aldol chemoselectivity may provide new tactical opportunities for the synthesis of complex molecular targets.

## 1. Introduction

With the advent of organocatalysis [1,2], a wide variety of mild reaction conditions became available and enabled new opportunities for bond formation with chemo-, regio-, and stereocontrol. In this context, chemocontrol has been infrequently reviewed [3] (for selected examples, see [4,5,6,7,8,9]), and the field remains underdeveloped. In that light, we recently demonstrated that enantioselective aldol reactions can occur despite the presence of Knoevenagel nucleophiles [10,11].

Aldehydes are common electrophiles for both aldol and Henry reactions, so the purpose of this study was to determine if aldol chemoselectivity was feasible in the presence of nitroalkanes with pK_a_ values ranging from 5.5 to 10.0. Proposing base-catalyzed competition reactions between common aldol pronucleophiles (e.g., ketones) and Henry pronucleophiles (e.g., nitroalkanes) would not lead to aldol chemoselectivity because the latter have significantly lower pK_a_ values [12]. On the other hand, aldol, but not Henry, reactions can proceed via enamine-based catalysis, and such an approach might allow the stated goal of aldol chemoselectivity. However, the required enamine catalysts, invariably chiral primary or secondary amines [13,14,15,16,17,18], can alternatively act as weak bases and catalyze the Henry reaction. In this context, knowledge of Henry reaction conditions is essential to determine whether aldol-over-Henry chemoselectivity is feasible or not.

**Scheme 1:**
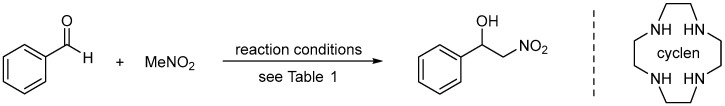
A commonly studied Henry reaction relevant to this study.

**Figure 1:**
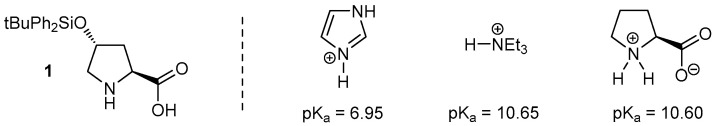
Left panel: organocatalyst used in this study. Right panel: water-based pK_a_ values for relevant protonated amines.

Reviews of enantioselective transition metal [19,20,21,22,23] and tertiary amine [19,20,21,24] catalyzed Henry reactions are known, but we required knowledge of reaction conditions allowing Henry bond formation in the absence of activating agents, e.g., metals or hydrogen bond donors. A good starting point, which also dovetails with this study, is the addition of nitromethane to benzaldehyde (Scheme 1) [25,26,27,28,29,30]. Methods employing fluoride, hydroxide, alkoxide, carbonate, DBU, or tetramethylguanidine (TMG) catalysis for Henry product formation have been reported [31], but we were interested in reaction conditions that more closely resembled the mild conditions we would employ, i.e., amino acid catalysis (Figure 1, catalyst **1**) in the presence of water. Accordingly, Table 1 summarizes reports that detail mild catalysis while adhering to the following criteria: room temperature reactions, reasonable nitromethane-to-benzaldehyde ratios, and reported yields. The mild nature of the Table 1 Henry reaction conditions is striking, e.g., Borah’s imidazole method at room temperature in water (Table 1, entry 4) has been extensively used by Pan [32,33] and others [34], implying the usefulness of this method for nitromethane deprotonation and subsequent addition to aromatic aldehydes. And therein lies the chemoselectivity quandary at hand: the water-based pK_a_ of protonated imidazole (6.95) [35,36] is significantly lower than that of Et_3_N (10.65) [37,38] or proline (10.60) [39,40], suggesting that the Hayashi [41] popularized aldol catalyst **1** (Figure 1) would promote Henry over aldol chemoselectivity due to the ease of deprotonation versus enamine reaction pathways.

## 2. Discussion and Results

### 2.1. Aldol Versus Henry Competition Reactions

**Scheme 2:**
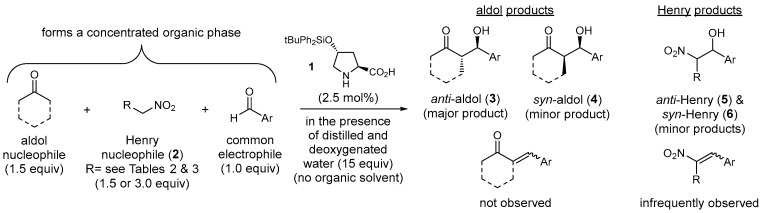
In-water competition reaction conditions and possible products.

We performed our competition reactions using in-water reaction conditions, a concentrated organic phase in the presence of a water phase, because they are well known for permitting highly enantioselective aldol reactions [13,14,15,16,17,18]. For a summary or comprehensive description of on-water versus in-water reaction conditions, respectively, see [18] or [42]. Scheme 2 provides a global overview of our competition reactions, and, unless otherwise stated, the aldol and Henry nucleophiles were fully soluble in the concentrated organic phase at t = 0 h. In this way, fair competition reactions were possible. Finally, only catalyst **1** was examined because it has historically been proven to be an outstanding organocatalyst for the generic type of aldol reactions examined in this study [18,41].

To establish the viability of our chemoselectivity concept, we started with the reaction of cyclohexanone with different *o*-, *m*-, and *p*-substituted benzaldehydes in the presence of different aliphatic nitroalkanes: nitromethane, 1-nitropropane, and methyl 4-nitrobutanoate (Table 2). To test the limits of this methodology, we first examined the least hindered nitroalkane, i.e., nitromethane (Table 2, entries 1–4). However, before arriving at the Table 2 reaction conditions, we first examined the reaction of cyclohexanone (1.5 equiv) and nitromethane (1.5 equiv) competing for 4-nitrobenzaldehyde (1.0 equiv) in the presence of catalyst **1** (2.5 mol%). Complete aldol chemoselectivity with high aldol product yield was observed. Based on that result, we increased the equiv of the Henry nucleophile such that the aldol nucleophile/Henry nucleophile/aldehyde electrophile equiv ratio was changed to 1.5:3.0:1.0 equiv for the first seven competition reactions of Table 2 (entries 1–7). Without exception, excellent aldol product profiles were observed, and no Henry product was formed (<5% yield).

Complete aldol chemoselectivity was noted for cyclohexanone, so we next examined a slow-reacting cyclic ketone, i.e., 4-methylcyclohexanone (Table 2, entry 8), and one electronically modified cyclic ketone: a dioxanone (Table 2, entry 9). For entries 8 and 9, equal equivalents of the ketone and 1-nitropropane were employed with an elevated catalyst loading (5 mol%); again, high aldol/Henry chemoselectivity (>19:1) was observed for those two substrates. Finally, we evaluated a different nitroalkane, methyl 4-nitrobutanoate (Table 2, entry 10), and observed the same high aldol chemoselectivity as noted for nitromethane and 1-nitropropane (Table 2, entries 1–9).

All Table 2 aldol products have been previously synthesized using an organocatalyst, and references for the specific products [43,44,45,46,47] are found with the entry number in Table 2. From those products, only the aldol product **3a** has been synthesized using catalyst **1** [41]. Thus, Hayashi reported reacting 4-nitrobenzaldehyde (1.0 equiv) with cyclohexanone (2.0 equiv) using catalyst **1** (1 mol%) in the presence of water (2.0 equiv), providing **3a** (t = 42 h, 89% yield, 20:1 *dr*, 97% *ee*). For our competition reactions producing **3a** (Table 2, entries 1, 5, and 10), we used fewer equivalents of cyclohexanone (1.5 equiv) and observed very similar product profiles with shorter reaction times, presumably because we used a higher catalyst loading (2.5 mol%) than Hayashi reported (1 mol%). We justified our use of elevated catalyst loadings to minimize potential off-cycle sequestration of catalyst **1**, e.g., nitroalkane deprotonation. In summary, the Table 2 data, which spans three cyclic ketones and a range of *o*-, *m*-, and *p*-substituted aldehydes, provides sufficient steric and electronic diversity to establish that high aldol chemoselectivity is maintained in the presence of simple nitroalkanes (alkyl-CH_2_NO_2_). Despite this unequivocal finding, it was not a foregone conclusion before this study was undertaken, and especially so given the Henry reaction conditions summarized in Table 1.

Table 2 evaluates nitroalkanes (RCH_2_NO_2_, R = H or alkyl) that have pK_a_ values of 8–10 [48,49,50]; however, can high aldol chemoselectivity be maintained when the pK_a_ is decreased, e.g., when R is alkenyl, phenyl, or carbonyl-based To test that, a series of nitro compounds was either synthesized (**2a**–**e**) or purchased (**2f** and **2g**) and examined in equal molar quantities against the aldol competitor cyclohexanone (Table 3). For this, 3-chlorobenzaldehyde and 4-(trifluoromethyl)benzaldehyde were chosen as the common electrophiles for examination because they are liquids and, in most instances, facilitated full dissolution of the solid nitro compounds in the concentrated organic layer.

Allylic nitro compounds **2a** and **2b** were investigated first and intriguingly resulted in high aldol/Henry chemoselectivity (>19:1) with good aldol product profiles (Table 3, entries 1–4). However, when the allylic nitro group was further conjugated to an aromatic ring, as in **2c** (Table 3, entries 5 and 6), a pK_a_ threshold was crossed, activating the Henry pathway and lowering the aldol/Henry chemoselectivity to 6.4:1 for 3-chlorobenzaldehyde and 6.8:1 for 4-(trifluoromethyl)benzaldehyde. Despite this, the aldol product yields remained acceptable, at 76% (**3e**, entry 5) and 74% (**3h**, entry 6), respectively.

Examination of **2d**, a 7-bromo derivative of **2c**, unfortunately resulted in an unfair competition wherein ~15% of **2d**, by visual inspection at 1 h, remained undissolved in the concentrated organic layer, i.e., the cyclohexanone and 3-chlorobenzaldehyde (Table 3, entry 7). To address this solubility limitation, we (i) switched to the 4-(trifluoromethyl) benzaldehyde electrophile (entry 8) and (ii) examined allylic nitro compound **2e**, a 6-bromo derivative of **2b**, with 3-chlorobenzaldehyde (entry 9). Unfortunately, in both instances, the allylic nitro compound was significantly less soluble compared to the solubility noted for **2d** in the entry 7 competition reaction. Accordingly, the aldol product data are not shown for entries 8 and 9 (Table 3). Those failures prompted us to investigate **2d** under mechanochemical methods. In the event, ball-milling reactions with 3-chlorobenzaldehyde and 4-(trifluoromethyl)benzaldehyde provided mediocre chemoselectivity for aldol products **3e** and **3h**, with respective yields of 11% and 23% (data not in table; see Section 3 (Materials and Methods) for reaction conditions).

The impractical outcomes for substrates **2d** and **2e** arise from solubility constraints under in-water conditions and do not undermine the chemoselectivity concept advanced in this study. For example, one anticipated outcome of this study is its application to molecules bearing both ketone and nitroalkane moieties, wherein the ketone undergoes an intermolecular reaction with an electrophile while the nitroalkane remains unreacted. While that substrate category does not remove potential solubility limitations, it does remove unfair competition reactions because both nucleophilic competitors are equally restricted if solubility is a limiting factor.

Building on our investigation of nitro-based compounds with lower acidity, (nitromethyl)benzene (**2f**), which has a reported pK_a_ value of 6.77 [50], was examined next. Because **2f** is a liquid, we flexibly changed to the use of 4-nitrobenzaldehyde, a solid, as the electrophile for this competition reaction. In the event, an aldol/Henry chemoselectivity of 5.4:1 was observed, resulting in a reduced but respectable 75% yield for *anti*-aldol product **3a** (Table 3, entry 10). This reaction was also carried out under ball-milling conditions; however, under an optimal reaction time of 9 h, it proved to be less efficient than our standard method using a stir bar (Table 3, entry 10).

Finally, a practical yield boundary was encountered when exploring ethyl nitroacetate (**2g**), pK_a_ = 5.67 [51], with 3-chlorobenzaldehyde. Even so, it was remarkable that a 60% yield of *anti*-aldol product **3e** (Table 3, entry 11) was obtained. In this instance, the chemoselectivity could not be assessed from the crude ^1^H NMR due to multiple indeterminate resonances from possible Henry and/or Henry condensation products and unknown products; for a discussion, see the Supplementary Materials Section S8.

### 2.2. Henry Product Reference Standards

**Figure 2:**
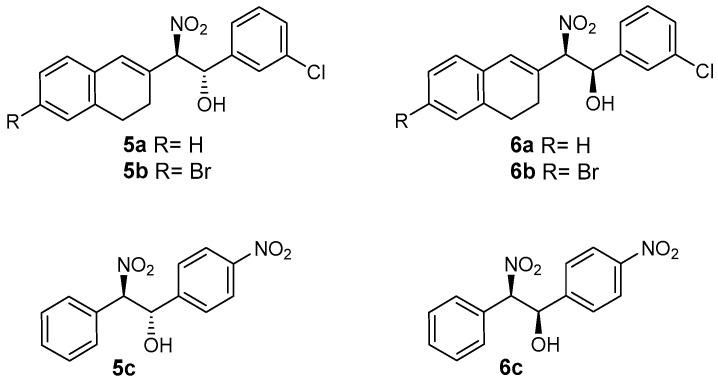
Henry product reference standards. Relative stereochemistry is provided; the enantiopurity was not determined.

The Table 3 aldol/Henry chemoselectivity ratios were determined using crude product ^1^H NMR analysis. To ensure the trustworthiness of that data, the *anti*-Henry (**5**) and *syn*-Henry (**6**) products of nitro compounds **2c** and **2d** with 3-chlorobenzaldehyde and **2f** with 4-nitrobenzaldehyde (Figure 2) were synthesized and characterized. For example, **5a**/**6a** were isolated as an inseparable mixture, albeit pure, from several screening reactions, whereas Henry products **5b**/**6b** and **5c**/**6c** were intentionally synthesized, each isolated in pure form, and characterized. These Henry reference samples were prepared using competition reaction conditions analogous to those described, but in the absence of cyclohexanone and with extended reaction times; see Supplementary Materials Section S5. Those efforts unequivocally establish the reported chemoselectivity ratios for entries 5, 7, and 10 (Table 3). However, for the corresponding Henry product with 4-(trifluoromethyl)benzaldehyde (Table 3, entry 6), no reference standard was synthesized. Instead, we used the ^1^H NMR chemical shift trends obtained from the 3-chlorobenzaldehyde Henry products (**5a**–**c and 6a**–**c**) to guide our ^1^H NMR identification of those Henry products.

### 2.3. Solvent Studies: Uniqueness of Water-Based Solvent Systems

The above examples established the viability of aldol chemoselectivity in the presence of a variety of nitroalkanes. However, given the Henry literature (Scheme 1, Table 1), any prediction of our results prior to their experimental confirmation (Table 2 and Table 3) would have been speculative. Accordingly, this prompted us to evaluate the role, if any, of the aqueous phase in determining chemoselectivity. To probe that topic, we performed competition reactions in several common Henry reaction solvents: DMSO, THF, EtOH, and H_2_O, as noted in Table 1.

**Scheme 3:**
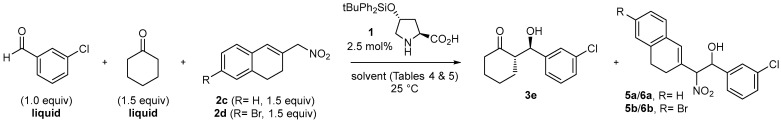
Competition reaction solvent study using allylic nitro compound **2c** or **2d**.

Table 4 provides an overview of the competition reactions between nitro compound **2d** and cyclohexanone (Scheme 3) in a variety of solvents. Four reactions were deemed unfair due to a lack of full solubility of **2d** even at t = 1 h (Table 4, entries 1, 2, 4, and 7). By contrast, anhydrous DMSO (entry 3) provided a homogenous solution, but an unknown as the major product, resulting in an estimated total yield of 45% for the unknown product, aldol products, and Henry products in an approximate ratio of 4:3:1. The unidentified compound appears to incorporate two molecules of **2d** and one molecule of 3-chlorobenzaldehyde; see Supplementary Materials Section S10 for ^1^H and ^13^C NMR and HRMS data. Use of EtOH (0.5 M) resulted in homogeneous reactions, but even with elevated catalyst loadings of 20 or 5 mol% (Table 4, entries 5 and 6), significant quantities of the limiting reactant, 3-chlorobenzaldehyde, remained unreacted, and the *dr* and *ee* were suppressed.

The Table 4 data yielded limited progress but indicated that DMSO should be avoided, that EtOH and MeOH warrant focus, and that a competition experiment between cyclohexanone and nitro compound **2c** is preferable, as **2c** is a low-melting solid without solubility limitations. We began by establishing that the in-water reaction time could not be reduced without a yield penalty (Table 5, entry 2) and then examined neat reaction conditions, which interestingly provided high chemoselectivity but intolerably poor *dr* and reduced *ee* (entry 3). Next, we investigated EtOH and noted a superior product profile when reducing the catalyst loadings from 20 to 5 to 2.5 mol% (Table 5, entries 4, 5, and 6). Switching to an MeOH solvent, albeit maintaining the best EtOH reaction conditions, further improved the overall result (entry 7). However, even the MeOH-based outcome did not surpass the in-water reaction conditions (entry 1). Finally, inspired by Lombardo and Quintavalla [52], we employed a mixture of MeOH/H_2_O (3:2 equiv), resulting in a slightly superior result as compared to the in-water reaction conditions (Table 5, compare entries 1 to 8), albeit at the expense of reaction time, which doubled (32 to 60 h).

**Scheme 4:**
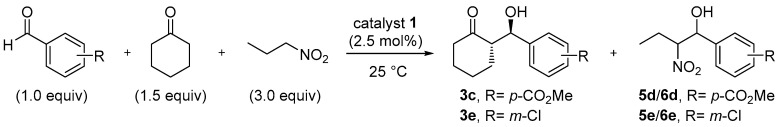
Competition reaction solvent study using methyl-4-formylbenzoate and 3-chlorobenzaldehyde.

To determine whether the Table 5 H_2_O (15 equiv) versus MeOH/H_2_O (3:2 equiv) conditions reflected a trend, we re-examined two prior competition reactions: (i) 1-nitropropane/cyclohexanone/methyl 4-formylbenzoate (Table 2, entry 6) and (ii) 1-nitropropane/cyclohexanone/3-chlorobenzaldehyde (Table 2, entry 7), albeit now using the MeOH/H_2_O (3:2 equiv) solvent system (Scheme 4 and Table 6). Using the H_2_O (15 equiv) reaction times, the MeOH/H_2_O (3:2 equiv) conditions provided aldol products **3c** (Table 6, compare entries 1 and 2) and **3e** (Table 6, compare entries 3 and 5) in slightly reduced yield, with the same *ee* but with significantly higher *dr*. The lower yield outcome is consistent with our Table 5 observations, but the increased *dr* was not anticipated, changing from 10:1 to >19:1 for **3c** and 13:1 to >19:1 for **3e**. In conclusion, the MeOH/H_2_O (3:2 equiv) conditions should be considered when longer times are acceptable and/or when the *dr* is not sufficient.

## 3. Materials and Methods

*Representative Competition Experimental Procedure*: (S)-2-((R)-hydroxy(4-nitrophenyl)methyl)cyclohexan-1-one (**3a**). To a clean, screw-cap, V-shaped reaction vessel (5.0 mL) equipped with a small pyramidal stir bar, mortar and pestle, ground 4-nitrobenzaldehyde (MW = 151.12 g/mol, 1.00 equiv, 1.50 mmol, 226.7 mg), cyclohexanone (MW = 98.14 g/mol, 1.50 equiv, 2.25 mmol, 220.84 mg, 233 µL, density 0.948 g/mL), and nitromethane (MW = 61.04 g/mol, 3.00 equiv, 4.5 mmol, 274.68 mg, 243 µL, density 1.132 g/mL) were added. The liquid reactants (cyclohexanone and nitromethane) were used to rinse the solid 4-nitrobenzaldehyde off the walls as needed. The heterogeneous mixture was allowed to gently stir for <5 min, but visual inspection showed little or no dissolution of the 4-nitrobenzaldehyde. Next, the stirring was terminated, and *trans*-4-(tert-butyldiphenylsilyloxy)-L-proline (MW = 369.54 g/mol, 2.5 mol%, 0.0375 mmol, 13.9 mg) was added. Distilled deoxygenated water (MW = 18.02 g/mol, 15.00 equiv, 22.5 mmol, 405.45 mg, 406 µL) was added within 30 s. The resulting heterogeneous solution was gently stirred such that the contents of the vessel did not splash against the vessel walls. Undissolved solids remained until about 6 h into the reaction, at which point both layers became transparent. The rate of stirring only gently agitated the phase boundary, and the reaction was quenched at 24 h. See Section S2 of the Supplementary Materials for the work-up procedure.

*Representative Ball Milling Competition Experimental Procedure:* (S)-2-((R)-hydroxy(4-nitrophenyl)methyl)cyclohexan-1-one (**3a**). To a clean plastic Eppendorf (2 mL) equipped with three 3 mm stainless steel balls, 4-nitrobenzaldehyde (MW = 151.12 g/mol, 1.00 equiv, 0.12 mmol, 18.0 mg), cyclohexanone (MW = 98.14 g/mol, 1.50 equiv, 0.18 mmol, 19.2 µL, density = 0.947 g/mL), (nitromethyl)benzene (MW = 137.14 g/mol, 1.50 equiv, 0.18 mmol, 15 µL, density = 1.158 g/mL), and *trans*-4-(tert-butyldiphenylsilyloxy)-L-proline (MW = 369.54 g/mol, 2.50 mol%, 0.003 mmol, 1.1 mg) were added. The snap-cap Eppendorf was closed, and the reaction was mixed in a ball mill (Retsch vibrating mill MM 200, Retsch GmbH, Haan, Germany) for 9 h at a frequency of 16 Hz under air and room temperature conditions. During mixing, the shaking was periodically stopped every 90 min for 2–3 min to prevent excessive heating. See Section S2 of the Supplementary Materials for the work-up procedure.

## 4. Conclusions

The purpose of this competition study was to assess the feasibility of performing aldol chemoselective reactions at the expense of Henry reactions. Using in-water reaction conditions, we established that typical nitroalkanes, with pK_a_ values of 8–10, pose no chemoselective challenge during enantioselective aldol reactions catalyzed by secondary amine catalyst **1**. That outcome was also true for allylic nitro compound (**2b**). However, an allylic nitro compound conjugated to a phenyl group (**2c**) and nitro compounds with electron-withdrawing α-substituents, e.g., -Ph (**2f**) or -CO_2_Et (**2g**), form Henry products with increasingly higher yield as the pK_a_ value of the nitro compound decreases. For example, (nitromethyl)benzene (**2f**), pK_a_ 6.77, provided a greater aldol yield (Table 3, entry 10, 75% yield) than ethyl nitroacetate (**2g**), pK_a_ 5.67 (Table 3, entry 11, 60% yield). It can be inferred that aldol chemoselectivity, under enamine catalysis promoted by catalyst **1**, begins to erode when the nitro compound has an estimated pK_a_ value of 7.0 or lower. In conclusion, this study has begun to define the boundary conditions that govern this previously undescribed chemoselectivity and, in doing so, has established a proof of concept.

Finally, the reactions outlined here, i.e., competition reactions, hold conceptual value; however, the practical value will only be evident when one molecule containing two of the three functional groups (a nitro compound with an α-proton, a ketone, and an aldehyde) is reacted with another molecule containing the remaining functional group. Future investigations of that kind may allow a tactical advantage to be identified and thus permit more efficient complex molecule synthesis.

## Figures and Tables

**Table 1:** Henry literature reports detailing mild reaction conditions for nitromethane addition to benzaldehyde (Scheme 1) ^a^.

Entry	MeNO_2_ (Equiv)	Additive	Solvent (Molarity)	Time	Yield ^b^ (%)
1 [25]	5.0	4 Å MS ^c^	DMSO (0.15)	24 h	91
2 [26]	3.0	phosphate buffer (pH = 7.0)	H_2_O (0.25)	18 h	78
3 [27]	1.4	NaOAc (33 mol%)	EtOH (1.35)	18 h	64
4 [28]	3.0	imidazole (25 mol%)	H_2_O (0.50)	15 min	90
5 [29] ^d^	4.0	DABCO (50 mol%)	CH_3_CN ^e^	24 h	95
6 [30]	2.0	cyclen (5 mol%)	THF (1.0)	24 h	80

^a^ Room temperature reactions. ^b^ Isolated yield data after column chromatography. ^c^ 200 mg of 4 Å molecular sieves per 1 mmol of PhC(O)H (limiting reactant). ^d^ Reaction performed at 0 °C. ^e^ Molarity was not provided.

**Table 2:** Aldol versus Henry competition reactions (Scheme 2): nitroalkane (Henry nucleophile) and aldol product structure and profile ^a,b^.

Entry ^c^	Henry Nucleophile	Major Competition Product	Time (h)	*anti*-Aldol (3) Yield (%) ^d^	*dr* ^e^	*ee* ^f^
1 [43]	MeNO_2_	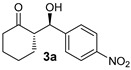	24	91	>19:1	99
2 [44]	MeNO_2_	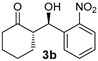	36	81	>19:1	99
3 [44]	MeNO_2_	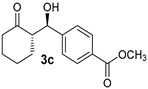	40	89	14:1	98
4 [45]	MeNO_2_	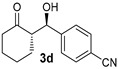	40	89	>19:1	99
5 [43]	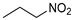	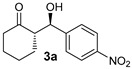	24	91	14:1	99
6 [44]	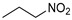	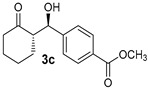	40	84	10:1	99
7 [43]	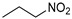	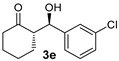	49	86	13:1	99
8 [46] ^g^	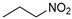	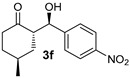	24	65	13:1 >19:1 ^h^	99
9 [47] ^g,i^	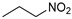	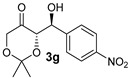	24	80	>19:1	99
10 [43] ^j^	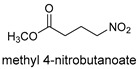	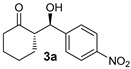	16	86	>19:1	99

^a^ Standard reaction conditions: ketone nucleophile (1.5 equiv), Henry nucleophile (3.0 equiv), aldehyde electrophile (1.0 equiv, 1.0 mmol), 2.5 mol% of catalyst **1** (Figure 1), and H_2_O (15 equiv) at room temperature. ^b^ Based on crude ^1^H NMR analysis, all Table 2 entries had aldol/Henry chemoselectivity >19:1. ^c^ The references provided with the entry number give characterization data for the indicated aldol product. ^d^ Isolated yield of *anti*-aldol product (single diastereomer) after silica gel chromatography. Note: product **3a** readily epimerizes and always contains some *syn*-aldol product **4** (Scheme 2); see the Supplementary Materials Section S7 for further details. ^e^ Based on crude ^1^H NMR analysis: ratio of the *anti*-/*syn*-aldol products. ^f^ Enantiomeric excess of *anti*-aldol product (Chiralcel OD-H HPLC column). ^g^ 2.0 equiv of both the ketone and nitroalkane were used with catalyst **1** (5.0 mol%). ^h^ Ratio of the *anti*-major/*anti*-minor; see Supplementary Materials Section S7 for structural details. ^i^ Brine (15 equiv) was used and superior to H_2_O (15 equiv). ^j^ Only 1.5 equiv of the nitroalkane was used.

**Table 3:** Competition reactions between more acidic nitroalkanes (**2a**–**g**) and cyclohexanone for 3-chlorobenzaldehyde or 4-(trifluoromethyl)benzaldehyde (Scheme 2) ^a^.

Entry	Henry Nucleophile	Major Competition Product ^b^	Chemoselectivity Aldol/ Henry ^c^	Time (h)	*Anti*-Aldol Yield (%) ^d^	*dr* ^c^	*ee* ^e^
1	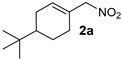	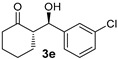	>19:1	32	84	13:1	97
2	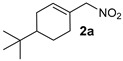	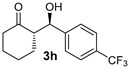	>19:1	32	82	14:1	98
3	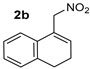	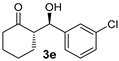	>19:1	32	68	16:1	98
4	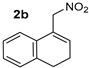	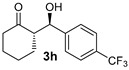	>19:1	32	74	13:1	99
5	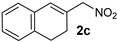	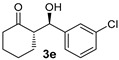	6.4:1	32	76	13:1	98
6	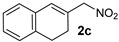	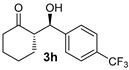	6.8:1	32	74	11:1	99
7 ^f^	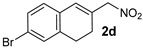	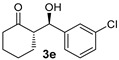	4.9:1	32	66	14:1	99
8 ^g^	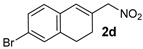	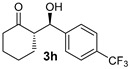	-	-	-	-	-
9 ^g^	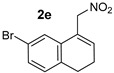	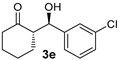	-	-	-	-	-
10	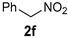	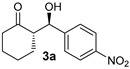	5.4:1 5.0:1 ^i^	24 9 ^i^	75 ^h^ 43 ^i^	14:1 7:1 ^i^	99 99 ^i^
11	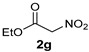	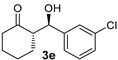	- ^j^	32	60	>11:1 ^k^	99

^a^ Standard reaction conditions: ketone nucleophile (1.5 equiv), Henry nucleophile (1.5 equiv), aldehyde electrophile (1.0 equiv, 0.70 mmol), 2.5 mol% of catalyst **1** (Figure 1), H_2_O (15 equiv) at room temperature. ^b^ For characterization data for **3a**, **3e**, and **3h** see [43]. ^c^ Based on crude ^1^H NMR analysis. ^d^ Isolated yield of *anti*-aldol product (single diastereomer) after silica gel chromatography. ^e^ Enantiomeric excess of *anti*-aldol product (Chiralcel OD-H HPLC column). ^f^ The data in this entry cannot be considered fair because the allylic nitro compound **2d** did not fully dissolve in the concentrated organic layer (see text). ^g^ The solubility of **2d** (entry 8) and **2e** (entry 9) in the concentrated organic layer was significantly less than that noted for **2d** in the entry 7 competition reaction. ^h^ Product **3a** readily epimerizes and always contains *syn*-aldol product **4** (Scheme 2); see Supplementary Materials Section S8 for further details. ^i^ Represents ball-milling data (see Supplementary Materials, Section S9). ^j^ Could not be determined; for details, see Supplementary Materials Section S8. ^k^ Due to overlapping resonance patterns, only a minimum *dr* could be established; see Supplementary Materials Section S8.

**Table 4:** Solvent chemoselectivity study: cyclohexanone versus nitro compound **2d** (Scheme 3) ^a^.

Entry	Description	Catalyst (mol%)	Solvent	Molarity (Equiv)	Time (h)	Chemoselectivity ^b^	3e (Yield %) ^c^	*dr* ^b^	*ee* ^d^
1	unfair ^e^	2.5	water	3.7 (15)	32	4.9:1	67	14:1	98
2	unfair ^e^	2.5	neat	-	32	5.62:1	- ^f^	5.6:1	- ^f^
3	homogeneous ^g^	25	DMSO	0.5	8	2.83:1	45 ^h,i^	1.9:1	- ^j^
4	unfair ^k^	15	THF	0.5	-	-	-	-	-
5	homogenous ^g^	20	EtOH	0.5	21	3.4:1	54 ^h^	2.4:1	86
6	homogenous ^g^	5	EtOH	0.5	32	5.54:1	36 ^h^	4.5:1	94
7	unfair ^e^	2.5	EtOH	1.14 (15)	32	6.1:1	- ^f^	4.5:1	- ^f^

^a^ Reaction parameters: cyclohexanone (1.5 equiv), Henry nucleophile **2d** (1.5 equiv), and 3-chlorobenzaldehyde electrophile (1.0 equiv, 0.47 mmol). For entry 1, 0.70 mmol of the limiting reactant (3-chlorobenzaldehyde) was used. ^b^ Determined by crude ^1^H NMR. ^c^ Isolated yield of *anti*-aldol (major) product. ^d^ Determined by chiral HPLC (Chiralcel OD-H column). ^e^ “Unfair” means unfair competition reaction because **2d** was not fully soluble. ^f^ Chromatography was not performed due to an unfair competition reaction. ^g^ Reaction solution was monophasic and homogeneous. ^h^ 15–25% 3-chlorobenzaldehyde remained (TLC). ^i^ The major product was not an aldol or Henry product and remains unidentified; see Supplementary Materials Section S10. ^j^ Not recorded. ^k^ At t = 0 to 2 min, the reaction was homogeneous; at 2 min, a large amount of solids precipitated. Arbitrary work-up at 4 h showed only reactants (^1^H NMR).

**Table 5:** Solvent chemoselectivity study: cyclohexanone versus nitro compound **2c** (Scheme 3) ^a^.

Entry	Solvent	Molarity (Equiv)	Time (h)	Chemoselectivity ^b^	3e (Yield %) ^c^	*dr* ^b^	*ee* ^d^
1 ^e^	water	3.7 (15)	32	6.8:1	76	13:1	98
2	water	3.7 (15)	24	5.7:1	68 ^f^	11:1	99
3	neat	-	32	13:1	75	5.3:1	93
4 ^g^	EtOH	0.5 (34)	22	6.1:1	55 ^f^	3.5:1	87
5 ^h^	EtOH	1.0 (17)	40	8.1:1	40 ^f^	4.8:1	94
6	EtOH	1.14 (15)	72	6.2:1	62 ^i^	7.7:1	98
7	MeOH	1.65 (15)	72	6.2:1	69 ^i^	9.1:1	99
8	MeOH/H_2_O	(3:2)	60	7.7:1	77	13:1	99
9	MeOH/H_2_O	(3:2)	32	7.7:1	55 ^f^	13:1	98

^a^ Reaction parameters: cyclohexanone (1.5 equiv), Henry nucleophile 2c (1.5 equiv), and 3-chlorobenzaldehyde electrophile (1.0 equiv, 0.47 mmol), with 2.5 mol% of **1**. Entries 1, 2, 8, and 9 were biphasic and had homogeneous concentrated organic layers at t = 0; all other reactions were monophasic and homogeneous. ^b^ Determined by crude ^1^H NMR. ^c^ Isolated yield of anti (major) aldol product. ^d^ Determined by chiral HPLC (Chiralcel OD-H column). ^e^ Table 3, entry 5. ^f^ 15% to 25% 3-chlorobenzaldehyde remained by TLC. ^g^ 20 mol% catalyst loading. ^h^ 5 mol% catalyst loading. ^i^ 10% 3-chlorobenzaldehyde remained by TLC.

**Table 6:** Water (15 equiv) versus MeOH/H_2_O (3:2 equiv) competition study of cyclohexanone versus 1-nitropropane for electrophiles methyl 4-formylbenzoate or 3-chlorobenzaldehyde (Scheme 4) ^a^.

Entry	Solvent (Equiv)	Time (h)	Chemoselectivity ^b^	(Yield %) ^c^	*dr* ^b^	*ee* 4 ^d^
1	Water (15 equiv)	40	>19:1	**3c**, 84	**3c**, 10:1	**3c**, 99
2	MeOH/H_2_O (3:2 equiv)	40	>19:1	**3c**, 76	**3c**, >19:1	**3c**, 99
3	Water (15 equiv)	49	>19:1	**3e**, 86	**3e**, 13:1	**3e**, 99
4	Water (15 equiv)	32	>19:1	**3e**, 74	**3e**, 13:1	**3e**, 98
5	MeOH/H_2_O (3.2 equiv)	49	>19:1	**3e**, 82	**3e**, >19:1	**3e**, 98

^a^ Reaction parameters: cyclohexanone (1.5 equiv), 1-nitropropane (3.0 equiv), aldehyde electrophile (1.0 equiv, 1.0 mmol). ^b^ Determined by crude ^1^H NMR. ^c^ Isolated yield of *anti* (major) aldol products **3c** or **3e** (Scheme 4). ^d^ Determined by chiral HPLC (Chiralcel OD-H column).

## Data Availability

The data presented in this study are available in the article and Supplementary Materials.

## Supplementary Materials

The following supporting information can be downloaded at https://www.mdpi.com/article/10.3390/molecules30244688/s1. It contains ten sections covering one hundred and forty-seven pages of detailed supportive information in the form of experimental descriptions, spectra, and chromatograms. For inquiries about Sections S1–S8 and S10, contact T.C.N. For inquiries about Section S9, contact D.A.A. References [53,54,55,56,57,58,59,60,61,62,63,64,65,66,67,68,69,70,71,72,73,74,75] are referenced in the Supplementary Materials.
