# Supplementary material for: Defining Aldol Chemoselectivity in the Presence of Henry Nucleophiles (Nitroalkanes)
Source: Molecules. 2025 Dec 7;30(24):4688. doi: 10.3390/molecules30244688 (PMC12735628; doi:10.3390/molecules30244688)
Supplement: Supplementary file 1 [file molecules-30-04688-s001.zip › molecules-4013250-supplementary.pdf]

Defining Aldol Chemoselectivity in the Presence of Henry Nucleophiles (Nitroalkanes)

Kritika B. Dwivedi[1], Patrick Knäbe[1], Nilesh N. Shitole[1], Aida H. Lakew[1], Ruslan Levochkin[1], Luis Paredes-Soler[2], Sofiia-Stefaniia Zhylinska[1], Diana Kochubei[1], Gabriela Guillena[2], Rafael Chinchilla[2], Diego A. Alonso[2,*], Thomas C. Nugent[1,*]

[1]School of Science, Constructor University, Campus Ring 1, 28759 Bremen, Germany.
[2]Department of Organic Chemistry and Institute of Organic Synthesis (ISO), University of Alicante, P.O. Box 99, 03080 Alicante, Spain.

SUPPLEMENTARY MATERIALS

Table of Contents:

Section S1. General information....S2

Section S2. General competition reaction information (in-water conditions)....S2

Section S3. Water solubility data for aldol and Henry nucleophiles....S4

Section S4. Allylic nitro compound synthesis **2a**, **b**, **c**, **d**, **e**....S5

Section S5. Henry product reference standard synthesis and characterization **5a**, **b**, **c**, & **6a**, **b**, **c**....S17

Section S6. Aldol racemate formation....S38

Section S7. Table 2 entries 1-10 experimental descriptions and characterization of *anti*-aldol products **3a**, **b**, **c**, **d**, **e**, **f**, **g**....S40

Section S8. Table 3 entries 1-7, 10, 11 experimental descriptions and characterization of *anti*-aldol product **3a**, **e**, **h**....S93

Section S9. Ball-milling experiment (Table 3, entry 10, footnote i)....S142

Section S10. Unidentified product: Table 4, entry 3....S144

Section 1. General information

Commercial reagents were used as received, unless otherwise stated, from their commercial source, usually Sigma-Aldrich. Routine monitoring of reactions was performed by thin-layer chromatography (TLC) using precoated plates of silica gel 60 F254 and visualized under ultraviolet irradiation (254 nm) and CAM (ceric ammonium molybdate) staining solution. Column chromatography was performed using silica gel 60 (0.040-0.063 mm), and a common mobile phase of EtOAc and petroleum ether (pet ether). The latter having a boiling point range of 40-60 °C. JEOL NMR (ECX) 400 MHz was used for the NMR analysis operating at 400 MHz (1H) and 100 MHz (13C) respectively. Chemical shifts (δ) were reported in parts per million (ppm) relative to $CHCl_3$ (7.26 ppm) for ^1H NMR and relative to $CHCl_3$ (77.16 ppm) for ^{13}C NMR. Multiplicities are abbreviated as (s = singlet, d = doublet, t = triplet, q = quartet, bs = broad singlet, m = multiplet). The enantiomeric excess, *ee*, was determined using a Chiralcel OD-H chiral HPLC column (6 x 250 mm, 5 µm particle size) with mixtures of n-hexane (Hex) and i-propanol (IPA) as eluents. Solvents were delivered at a flow rate of 1.0 mL/min, the column temperature was set to room temperature, and the UV detection wavelength was 254 nm unless otherwise stated.

Section 2. General competition reaction information (in-water conditions)

Standard reaction conditions: For all reactions, we used V shaped (conical) vials with matching pyramidal stir bars. When the limiting reactant (aldehyde) was used at the 1.0 or 1.5 mmol scale a 5.0 mL vial was used. When the limiting reactant (aldehyde) was used at the 0.70 mmol scale a 2.0 mL vial was used. Ketone (aldol) nucleophile (1.5 equiv), Henry nucleophile (1.5 or 3.0 equiv), aldehyde (1.0 mmol, 1.0 equiv, limiting reactant), catalyst (2.5 mol% of catalyst **1** (*trans*-4-(*tert*-butyldiphenylsilyloxy)-L-proline (see Scheme 2 for structure within manuscript), and distilled deoxygenated water (15 equiv). A small number of reactions deviate from the standard conditions and are recorded in the manuscript Table footnotes and the experimental descriptions as noted in Sections 7 and 8 of this document. Note: all solid reactants were mortar and pestle ground before weighing. Regarding reactant addition order, all reactions started by combining the ketone (aldol) nucleophile with the Henry nucleophile. From that point, the order of addition can be varied regarding the aldehyde, catalyst and water. However, the addition of water always adhered to one of the following: (i) after catalyst addition the water was added within 1 min, or alternatively (ii) water was added before the catalyst.

Reaction monitoring (**reaction progress, chemoselectivity, and *dr***): Both nucleophilic competitors were always fully dissolved in the concentrated organic layer at t= 0, unless otherwise stated. At times, solids accumulated after several hours and were assumed to be the product. Reaction progress could not be accurately monitored by TLC due to the heterogenous nature of these reactions. Instead, screening reactions were worked-up at set times and crude ^1H NMR data was used to determine reaction progress, aldol *versus* Henry chemoselectivity, and *anti-*/*syn*-aldol product ratios. Crude reaction products were generally high vacuum dried overnight to remove volatile reactants before collection of the ^1H NMR spectrum. *Note that ^1H NMR data of the crude product, after work-up and drying, of the reaction between cyclohexanone and 4-trifluoromethylbenzaldehyde was problematic. Each sample from the semi-solid product provided a different chemoselectivity. For that reason, the entire crude product was dissolved in $CDCl_3$ and only then did we record the ^1H NMR data as noted in Tables 2-3 of the manuscript and the spectrums within the experimental descriptions within this document.*

Work-up: The competition reaction (Table 2 and 3) was transferred to a separatory funnel already containing water (50 mL) and CH_2Cl_2 (15 mL). The reaction vial was further rinsed with CH_2Cl_2 (3 x 3 mL) and added to the separatory funnel. The CH_2Cl_2 was removed, and the aqueous phase was further extracted with CH_2Cl_2 (3 x 15 mL). The combined organic phases were dried over Na_2SO_4, filtered, and concentrated under rotary evaporation. *It is vital that the bath temperature remains <30 °C to suppress epimerization of the aldol product.*

Epimerization of aldol product 3a: Aldol product diastereomers of the structural category produced in this study are prone to epimerization on heating or on exposure to silica gel. Consequently, crude aldol product ^{1}H NMRs can show higher *dr* values than ^{1}H NMRs of the chromatographed aldol products. This, in large part, is why most researchers in this area decided long ago to evaluate the aldol diastereoselectivity using crude ^{1}H NMR spectrums. Furthermore, most researchers isolate the *anti*-/*syn*-aldol products together and report the total yield. For this study, we decided to isolate the *anti*-aldol products and record their yield, and that was possible for all products except *anti*-aldol product **3a**. The *anti*-aldol product **3a** epimerizes during chromatography to an extent that prohibits its isolation free of the *syn*-aldol product **4a**. For related matters, see page S4 within the Supporting Information of: Nugent, T.C.; Umar, M. N.; Bibi, A. Picolylamine as an Organocatalyst Template for Highly Diastereo- and Enantioselective Aqueous Aldol Reactions. *Org. Biomol. Chem.* **2010**, *8*, 4085-4089.

Aldehyde purity/purification: Solid aldehydes (2-nitrobenzaldehyde, 4-nitrobenzaldehyde, methyl 4-formylbenzoate, and 4-cyanobenzaldehyde) were used as purchased (no purification) but were always mortar and pestle ground. The liquid aldehydes (3-chlorobenzaldehyde and 4-(trifluoromethyl)benzaldehyde) were purchased and prone to oxidation on storage and were always purified immediately before use. Although wasteful, we found the following procedure useful for the purification of our liquid aldehydes. Under pressurized nitrogen, about 1.5 to 2.0 mL of the neat aldehyde was passed through a disposable glass pipet (diameter ≈ 6 mm) containing a small tightly packed cotton plug, sand (0.5 cm), and silica gel (1.7 to 2.0 cm). The first fraction (≈ 0.5 mL) was discarded, from the next fraction (≈ 0.75 mL) was immediately taken the required volume of aldehyde and added to the reaction without delay. During our initial experiments, ^{13}C NMR examination of the second fraction showed no carboxylic acid was present. After those positive results, we did not examine future fractions by ^{13}C NMR even though the structure of the liquid aldehyde changed. Because of the ease of aldehyde oxidation, we strongly recommend the use of *deoxygenated* distilled water for these reactions.

Water (solvent): The reaction conditions represent “in-water” reaction conditions (Kobayashi definition, see: Kitanosono, T.; Kobayashi, S. Reactions in Water Involving the “On-Water” Mechanism. *Chem. Eur. J.* **2020**, *26*, 9408–9429.) which can be described as a concentrated organic phase and a water phase coexisting. *15 equiv of distilled deoxygenated water were used, deoxygenation provided increased yield reproducibility.* These reactions were performed in screw capped vials (5.0 mL, when using 1.0 mmol (Table 3) or 1.5 mmol (Table 2) of the aldehyde limiting reactant) with a headspace containing atmospheric air. Despite this, the water was deoxygenated to reduce the aldehyde to carboxylic acid oxidation. Before each reaction, distilled water (250 mL) was deoxygenated using pressurized nitrogen via a fritted glass bubbling unit. The brine employed for Table 2, entry 9 (manuscript) was prepared with distilled deoxygenated water.

Section 3. Water solubility data for aldol and Henry nucleophiles

Table S1. Available water solubility data for the aldol and Henry nucleophiles.

**Substrate**	**CRC**[1]	**HASD**[2]	**ILO ICSC**[3]	**(ACS/Labs)**[4]	**Merck**[5]	**TGSC**[6]
Cyclohexanone	soluble	88.09 g/L (25 °C)	87 g/L (20 °C)	-	86 g/L (20 °C)	
4-(methyl) cyclohexanone	insoluble	no data	no data	7 g/L (25 °C)	no data	7.1 g/L (25 °C)
2,2-dimethyl-1,3-dioxan-5-one	soluble	no data	no data	131 g/L (25 °C)	no data	
nitromethane	soluble	99.34 g/L (20 °C) 110.0 g/L (25 °C)	no data	105 g/L (25 °C)	no data	
1-nitropropane	slightly soluble	13.81 g/L (20 °C)	14 g/L[7]	-	no data	
methyl 4-nitro-butyrate	no data	no data	no data	103 g/L (25 °C)	no data	
(nitromethyl)-benzene	no data	no data	no data	1 g/L (25 °C)	no data	

[1] Haynes, W.M. (Ed.) CRC Handbook of Chemistry and Physics, 95th ed.; CRC Press: Boca Raton, FL, USA, 2014.
[2] Yalkowsky, S.H.; He, Y.; Jain, P. Handbook of Aqueous Solubility Data, 2nd ed.; CRC Press: Boca Raton, FL, USA, 2010.
[3] ILC International Chemical Safety Cards (ICSC): www.ilo.org/dyn/icsc/showcard.home
[4] Predicted values found within "Substance Details" for structures in SciFinder, which use (ACS/Labs) Software.
[5] Merck Safety Data Sheet.
[6] Software predicted value, see: https://www.thegoodscentscompany.com/data/rw1108151.html
[7] No temperature provided.

Section S4. Allylic nitro compound synthesis **2a, b, c, d, e**.

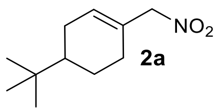

4-(tert-butyl)-1-(nitromethyl)cyclohex-1-ene (**2a**):

An adaption of the following procedure was used, see: Barton, D. H. R., Fernandez, I., Richard, C. S., Zard, S. Z. A Mild Procedure for the Reduction of Aliphatic Nitro Compounds to Oximes *Tetrahedron* **1987**, *43*, 551-558. To a clean round bottom flask (50 mL), equipped with an oval stirring bar and reflux condenser, flushed and maintained under N_2 was charged 4-(tert-butyl)cyclohexan-1-one (MW = 154.25 g/mol, 1.00 equiv, 19.45 mmol, 3.00 g), nitromethane (15.6 mL, 1.25 M), followed by ethylenediamine (MW = 60.10 g/mol, 5.0 mol%, 0.973 mmol, 58.5 mg, 65 µL, density = 0.90 g/mL) resulting in an orange colored homogenous solution. After 3 h under stirring at a gentle reflux, the reaction was cooled to room temperature and without further work-up was rotary evaporated to dryness, providing a brown colored viscous oil (4.12 g) as the crude product. Reaction progress was monitored by TLC (product R_f = 0.63, starting material R_f = 0.43 in EtOAc/petroleum ether (1:9)) and approximately 5% of the ketone remained. Note: 4-(tert-butyl)cyclohexan-1-one is UV inactive, but CAM stain active and the product is both UV and CAM stain active. The reaction changed from orange to brown to an intense brown color at 3 h.

Purification and yield: Silica gel chromatography (18 cm in height, 3.5 cm in diameter) using only petroleum ether elution provided the product as a colorless liquid (MW = 197.28 g/mol, 2.96 g, 15.00 mmol, 77% yield).

R_f: 0.63 (EtOAc/petroleum ether, 1:9).

^{1}H NMR (400 MHz, $CDCl_3$) (ppm) (Figure S1): δ 5.97-5.91 (m, 1H), 4.81 (s, 2H), 2.22-2.08 (m, 3H), 1.94-1.82 (m, 2H), 1.34-1.14 (m, 2H), 0.87 (s, 9H).

^{13}C NMR (100 MHz, $CDCl_3$) (ppm) (Figure S2): δ 133.8, 128.4, 82.5, 43.4, 32.3, 28.1, 27.3, 23.8.

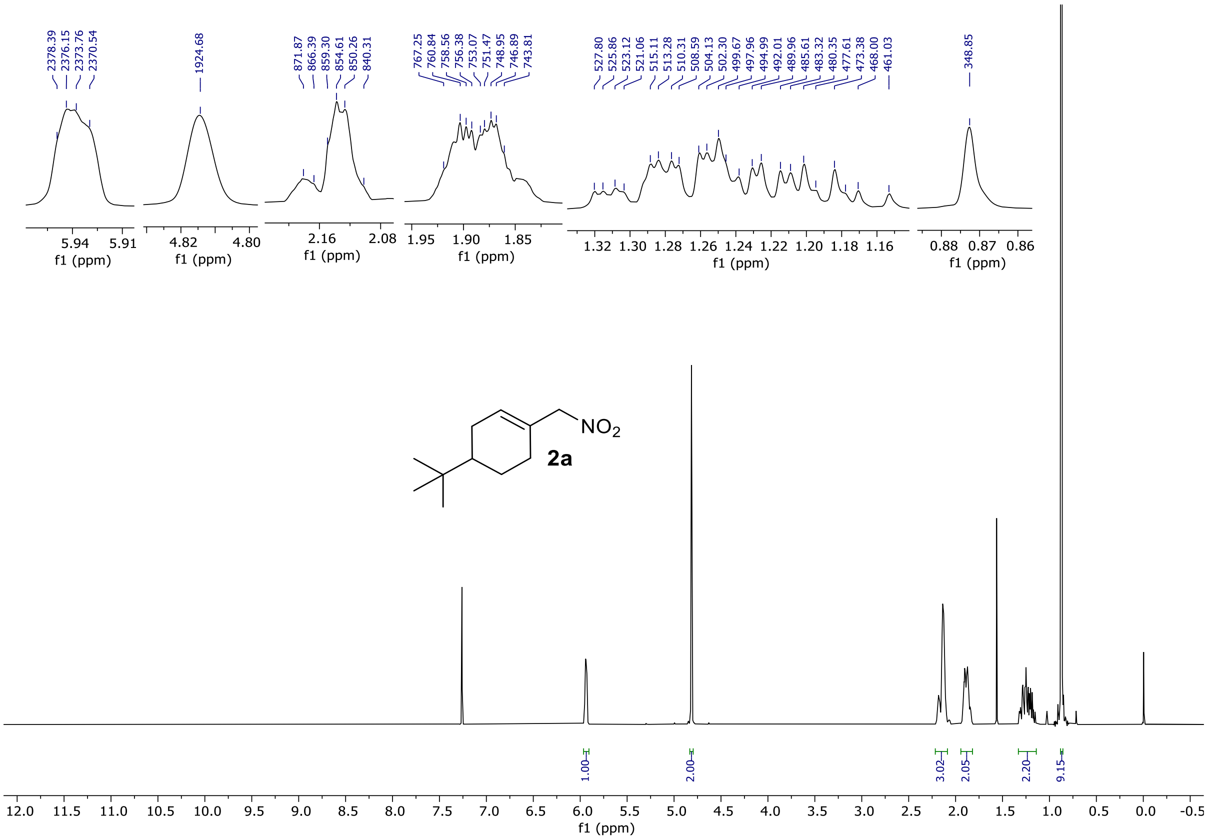

Figure S1. ^{1}H NMR spectrum of the purified product **2a**.

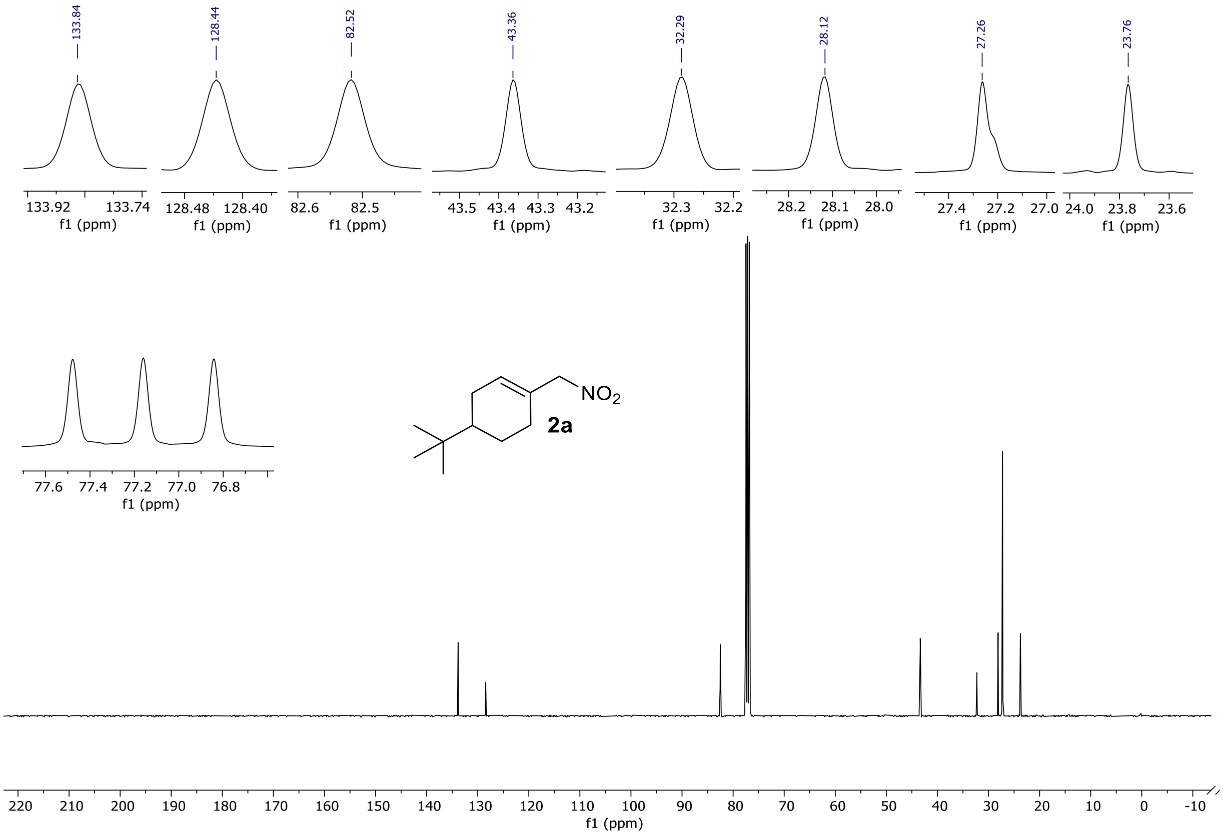

Figure S2. ^{13}C NMR spectrum of the purified product **2a**.

2b

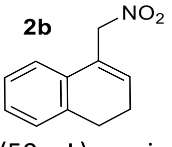

4-(nitromethyl)-1,2-dihydronaphthalene (**2b**):

An adaption of the following procedure was used, see: Barton, D. H. R.; Fernandez, I.; Richard, C. S.; Zard, S. Z. A Mild Procedure for the Reduction of Aliphatic Nitro Compounds to Oximes *Tetrahedron* **1987**, *43*, 551-558. To a clean round bottom flask (50 mL), equipped with an oval stirring bar and reflux condenser, flushed and maintained under N_2 was charged 1-tetralone (MW = 146.19 g/mol, 1.00 equiv, 36.00 mmol, 5.26 g). Nitromethane (28.9 mL, 1.25 M) was added, followed by ethylenediamine (MW = 60.10 g/mol, 5.0 mol%, 1.80 mmol, 108 mg, 120 µL, density = 0.90 g/mL) resulting in an off-white, clear solution. After 24 h under stirring at a gentle reflux, the reaction was cooled to room temperature and without further work-up was rotary evaporated to dryness, providing a brown but transparent solution (no weight taken) as the crude product. Reaction progress was monitored by TLC (product R_f = 0.25, 1-tetralone R_f = 0.09; petroleum ether/dichloromethane, 80:20). During the reaction, the solution changed from off-white (t= 0 h) to yellow (0.5 h) to slightly orange (1.5 h) to intense orange (7 h) and finally to an intense brown color (24 h).

This compound has been previously synthesized and characterized, see: Barco, A.; Benetti, S.; De Risi C.; Morelli, C. F.; Pollini, G. P.; Zanirato, V. Generation and Cycloaddition Reactions of Substituted 2-Nitro-1,3-dienes. *Tetrahedron* **1996**, *52*, 9275-9288.

Purification and yield: Silica gel chromatography (18 cm silica gel height, 5.5 cm column outer diameter) using isocratic elution (30 vol% CH_2Cl_2 in petroleum ether) allowed isolation of the product as an off-white waxy solid (5.36 g, MW = 189.21 g/mol, 28.32 mmol, 79% yield). Note: A second reaction, albeit on a smaller scale (7.50 mmol of 1-tetralone) and less concentrated (CH_3NO_2, 0.66 M), lead to a higher yield of **2b** (87%).

R_f: 0.25 (petroleum ether/dichloromethane, 80:20).

^{1}H NMR (400 MHz, $CDCl_3$) (ppm) (Figure S3): δ 7.25-7.14 (m, 4H), 6.32 (t, 1H, J = 4.6 Hz), 5.29 (s, 2H), 2.84 (t, 2H, J = 8.1 Hz), 2.46-2.39 (m, 2H).

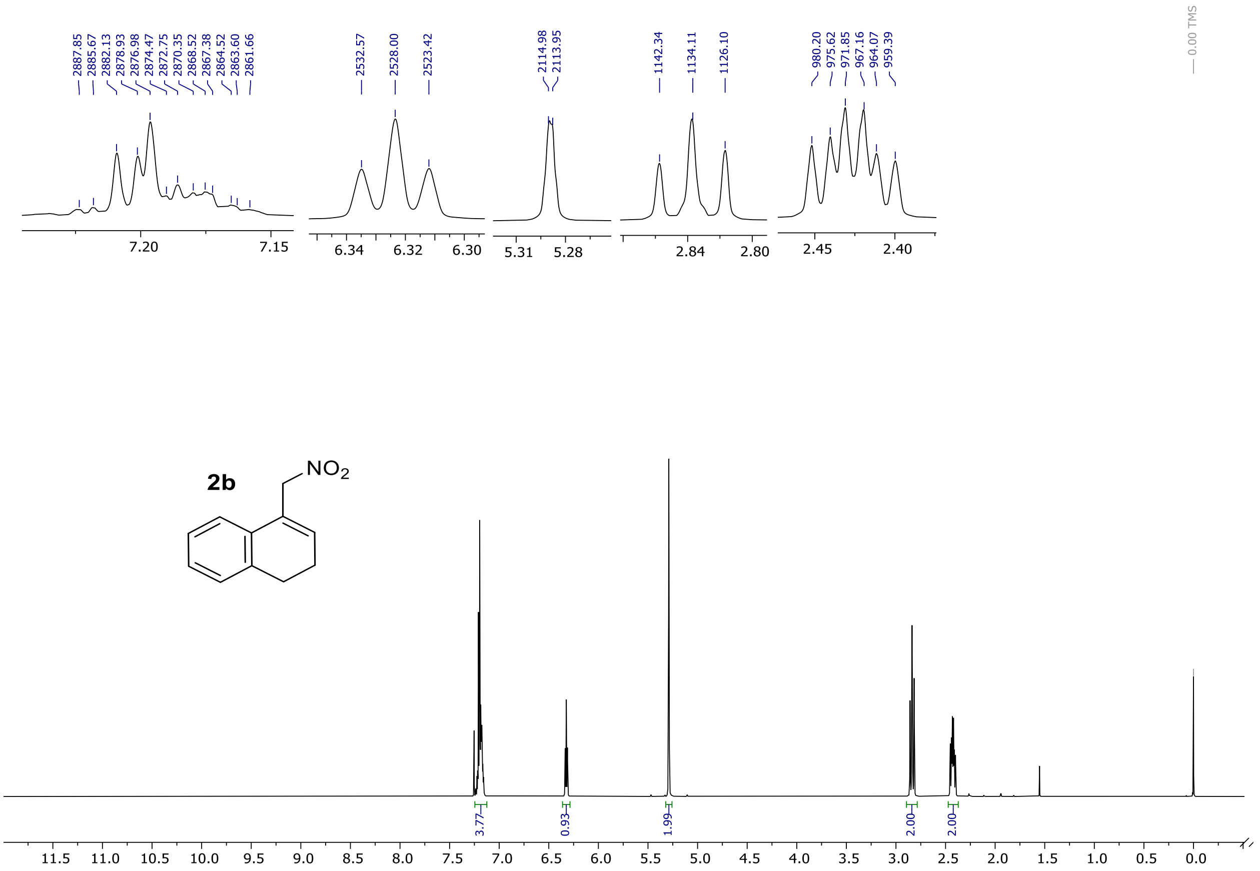

Figure S3. ^{1}H NMR spectrum of purified product **2b**.

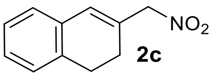

2c

3-(nitromethyl)-1,2-dihydronaphthalene (**2c**):

The following procedure was used, see: Barton, D. H. R.; Motherwell, W. B. B.; Zard, S. Z. Nouveau réactif de nitrométhylation, son application à la preparation de composes nitrométhylénés et de certains de leurs dérivés et les composes nouveaux obtenus. EP (European Patent) 0087359 A2, 31 August 1983, specifically page 14. To a clean round bottom flask (50 mL), equipped with an oval stirring bar and reflux condenser, flushed and maintained under N_2 was charged 2-tetralone (MW = 146.2 g/mol, 1.00 equiv, 20.52 mmol, 3.00 g, 2.712 mL, d = 1.106 g/mL), nitromethane (16.4 mL, 1.25 M), followed by ethylenediamine (MW = 60.10 g/mol, 5.0 mol%, 1.026 mmol, 61.66 mg, 69 µL, density = 0.90 g/mL) resulting in an orange colored solution. After 24 h under stirring at a gentle reflux, the reaction (now an intense brown color) was cooled to room temperature and without further work-up was rotary evaporated to dryness, providing a brown colored viscous oil (4.35 g) as the crude product. Reaction progress was monitored by TLC.

Purification and yield: Silica gel chromatography (16 cm in height, 4 cm in diameter) under gradient elution (10% → 20% CH_2Cl_2 in petroleum ether) allowed isolation of the product as a yellow liquid (MW = 189.1 g/mol, 1.55 g, 8.20 mmol, 40% yield). TLC (UV and CAM stain) shows one spot, but ^{1}H NMR shows what we approximate to be a 6% impurity (singlet at ~5.0 ppm). Note: We do not know what the impurity is, so the stated percentage is only an estimate (see Figure S4 for ^{1}H NMR integrations).

R_f: 0.35, CH_2Cl_2/petroleum ether (20:80).

^{1}H NMR (400 MHz, $CDCl_3$) (ppm) (Figure S4)**:** δ 7.24-7.06 (m, 4H), 6.62 (s, 1H), 5.05 (s, 2H), 2.91 (t, 2H, J = 8.2 Hz), 2.44 (t, 2H, J = 8.2 Hz).

^{13}C NMR (100 MHz, $CDCl_3$) (ppm) (Figure S5)**:** δ 135, 132.6, 132, 128.9, 128.4, 127.5, 127, 126.7, 81.6, 27.6, 25.4.

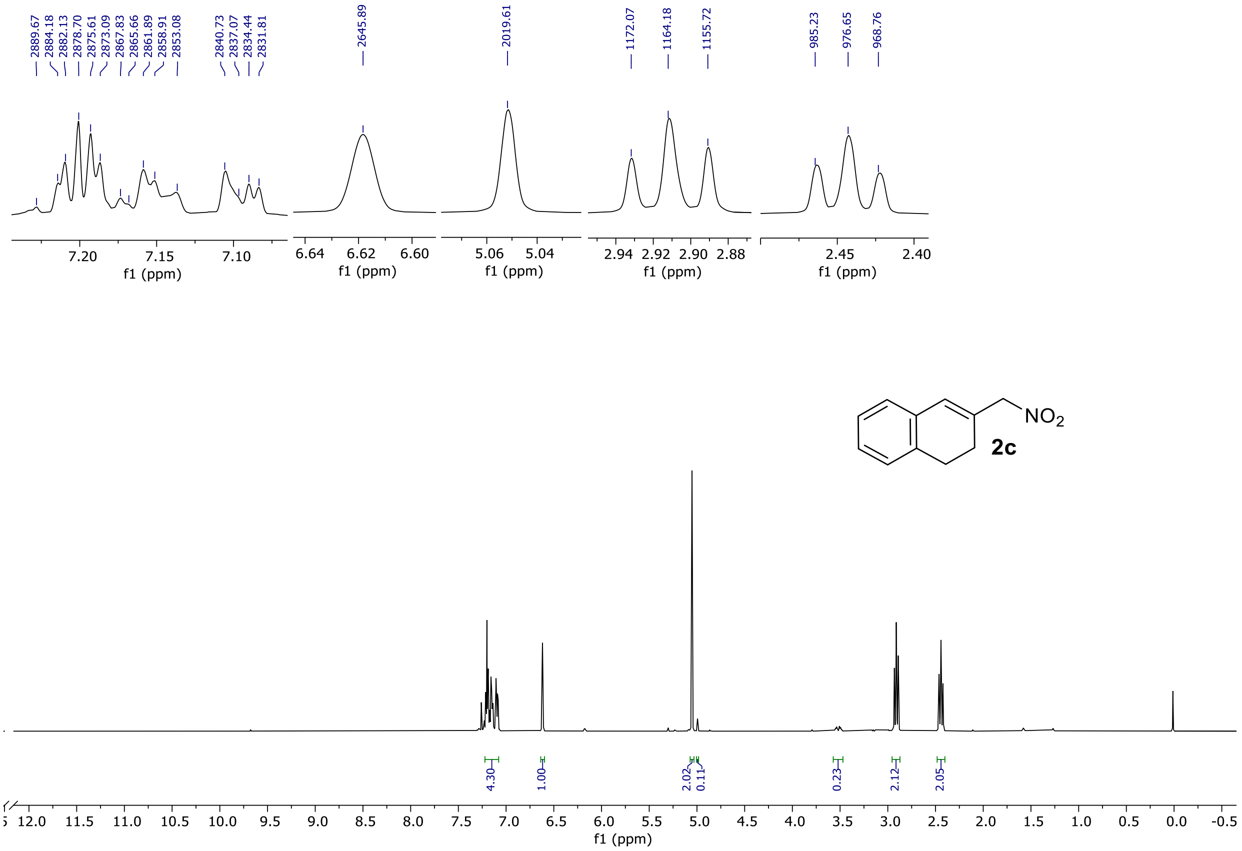

Figure S4. ^{1}H NMR of purified product **2c**.

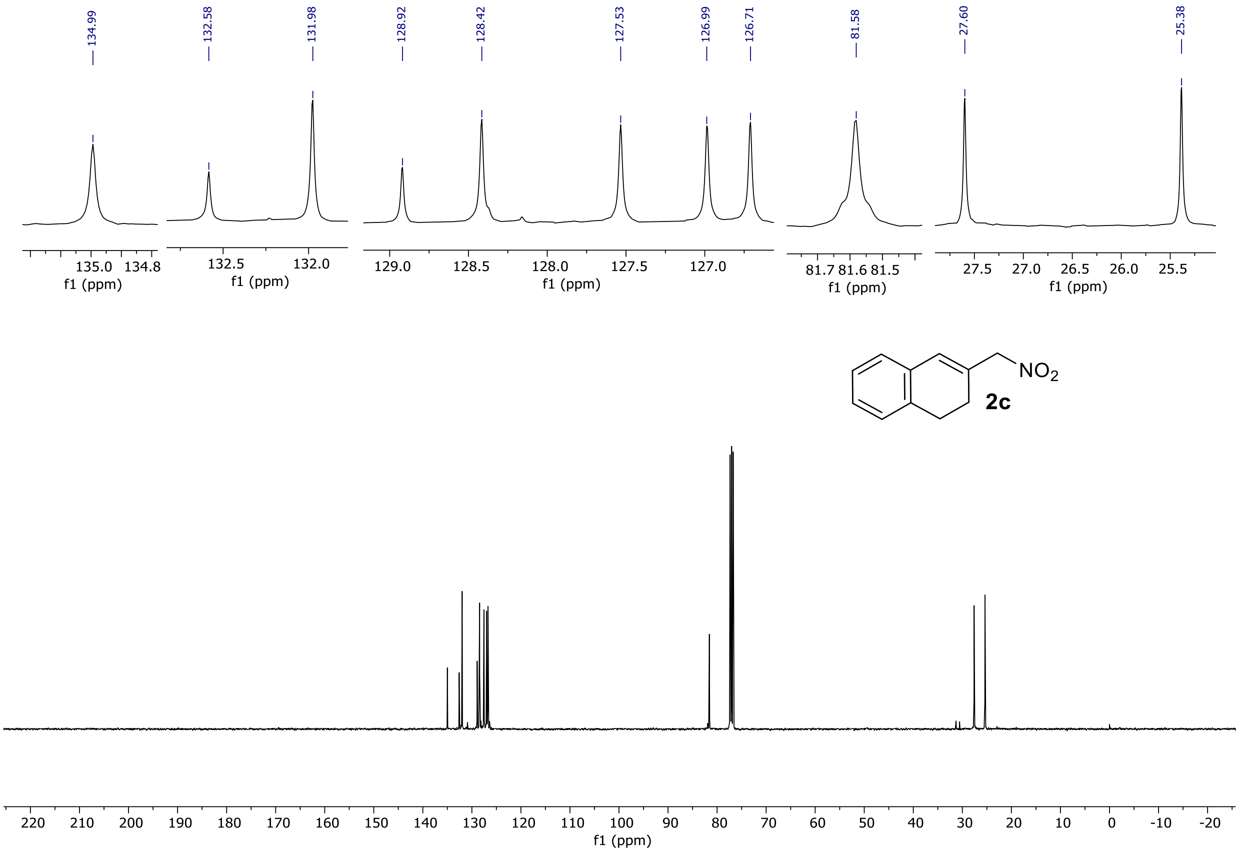

Figure S5. ^{13}C NMR of purified product **2c**.

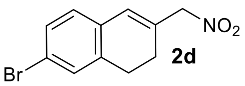

7-bromo-3-(nitromethyl)-1,2-dihydronaphthalene (**2d**):

An adaption of the following procedure was used, see: Barton, D. H. R.; Fernandez, I.; Richard, C. S.; Zard, S. Z. A Mild Procedure for the Reduction of Aliphatic Nitro Compounds to Oximes. *Tetrahedron* **1987**, *43*, 551-558. To a clean round bottom flask (50 mL), equipped with an oval stirring bar and reflux condenser, flushed and maintained under N_2 was charged 6-bromo-2-tetralone (MW = 225.09 g/mol, 1.00 equiv, 35.00 mmol, 7.88 g), nitromethane (28.0 mL, 1.25 M), followed by ethylenediamine (MW = 60.10 g/mol, 5.0 mol%, 1.75 mmol, 105 mg, 117 µL, density = 0.90 g/mL). After 24 h under stirring at a gentle reflux, the reaction was cooled to room temperature and without further work-up was rotary evaporated to dryness, providing a dark solid with liquid (no weight taken) which was purified by column chromatography. Reaction progress was monitored by TLC (product R_f = 0.35, 6-bromo-2-tetralone R_f = 0.11; hexanes/dichloromethane, 60:40) at different points in time. During the reaction the solution changed its color from amber (t = 0 h) to intense dark brown (t = 24 h), and dark brownish solids were observed.

Purification and yield: Silica gel chromatography (20 cm in height, 8 cm in diameter) using isocratic elution (40 vol% CH_2Cl_2 in hexanes) allowing isolation of the product as a yellowish fluffy solid (6.35 g, MW = 268.11 g/mol, 23.77 mmol, 68% yield). Note: A second reaction, albeit on a smaller scale (2.50 mmol of 6-bromo-2-tetralone) and less concentrated (CH_3NO_2, 0.66 M), lead to a reduced yield of **2d** (52%).

R_f: 0.35 (hexanes/dichloromethane, 60:40).

^{1}H NMR (400 MHz, $CDCl_3$) (ppm) (Figure S6): δ 7.33-7.28 (m, 2H), 6.95 (d, 1H, J = 7.8 Hz), 6.57 (s, 1H), 5.04 (s, 2H), 2.88 (t, 2H, J = 8.3 Hz), 2.42 (t, 2H, J = 8.2 Hz).

^{13}C NMR (100 MHz, $CDCl_3$) (ppm) (Figure S7): δ 137.1, 131.6, 131.1, 130.7, 129.8, 129.5, 128.4, 122.1, 81.5, 27.5, 25.2.

HRMS (ESI-QTOF) m/z (Figure S8): [M-H$^-$] Calculated for $C_{11}H_9BrNO_2$: 265.984390; Found: 265.982214, Error: -8.2 ppm.

IR (ATR, cm^{-1}) (Figure S9): ν = 2602, 2229, 2028, 1545, 1367, 821.

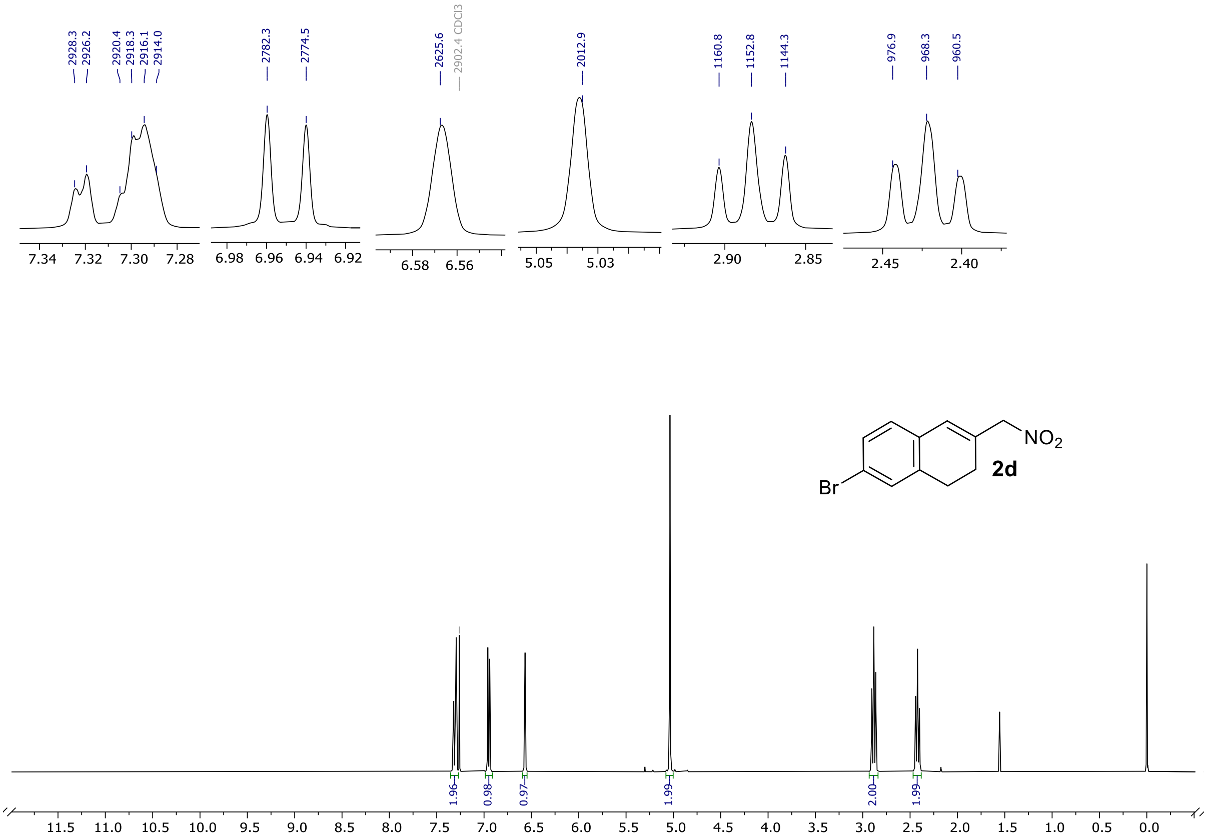

Figure S6. ^{1}H NMR spectrum of purified product **2d**.

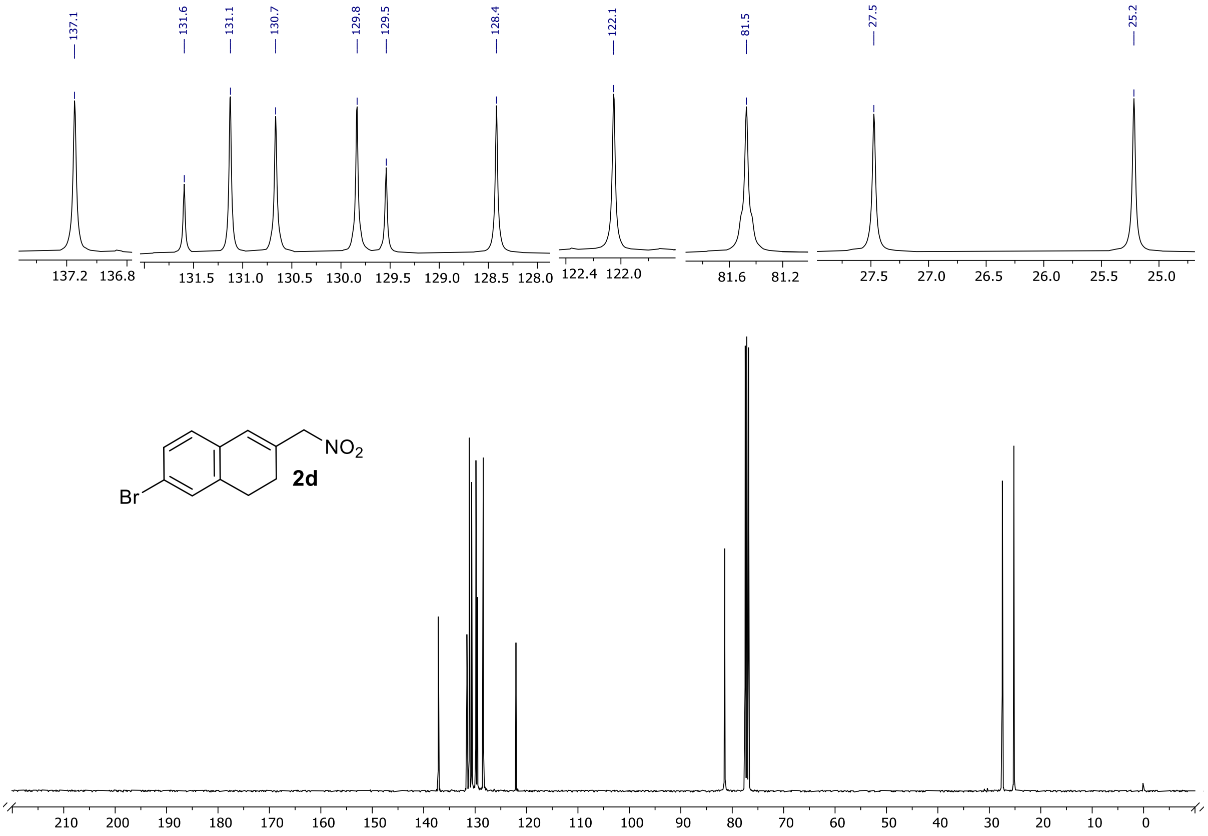

Figure S7. ^{13}C NMR spectrum of purified product **2d**.

Compound Spectrum SmartFormula Report

Analysis Info

Acquisition Date 10.03.2025 12:17:08

Analysis Name	C:\Users\jhoelscher\OneDrive - Constructor University\Nugent\MS data\Patrick\Mar 25\PK-024_10ppm_neg.d		
Method	Tune_neg_Standard.m	Operator	BDAL@DE
Sample Name	PK-024_10ppm_neg	Instrument	impact HD 1819696.00180
Comment			

Acquisition Parameter

Source Type	ESI	Ion Polarity	Negative	Set Nebulizer	0.3 Bar
Focus	Not active	Set Capillary	4500 V	Set Dry Heater	200 °C
Scan Begin	50 m/z	Set End Plate Offset	-500 V	Set Dry Gas	4.0 l/min
Scan End	2000 m/z	Set Charging Voltage	2000 V	Set Divert Valve	Source
		Set Corona	0 nA	Set APCI Heater	0 °C

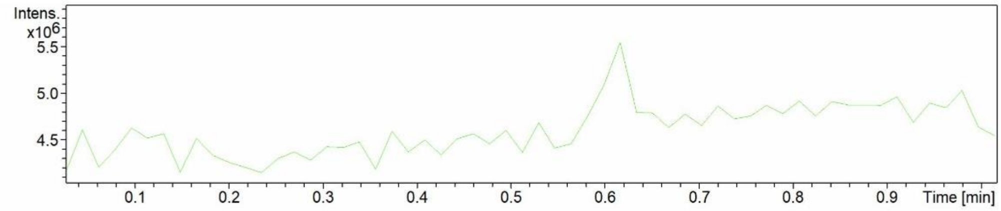

-MS, 0.4min #21

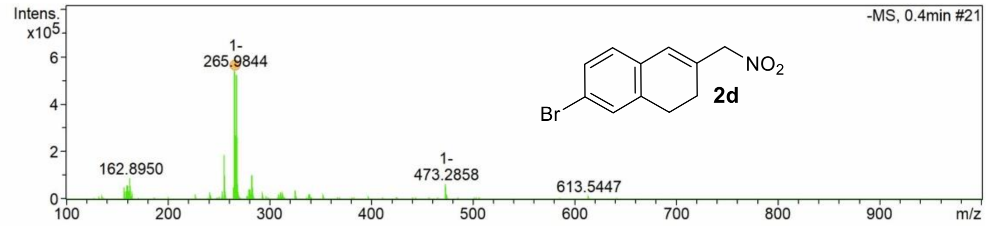

Meas. m/z	#	Ion Formula	Score	m/z	err [mDa]	err [ppm]	mSigma	rdb	e⁻ Conf	N-Rule	Adduct
265.984390	1	C11H9BrNO2	100.00	265.982214	-2.2	-8.2	6.8	7.5	even	ok	M-H

Figure S8. HRMS spectrum of purified product **2d**.

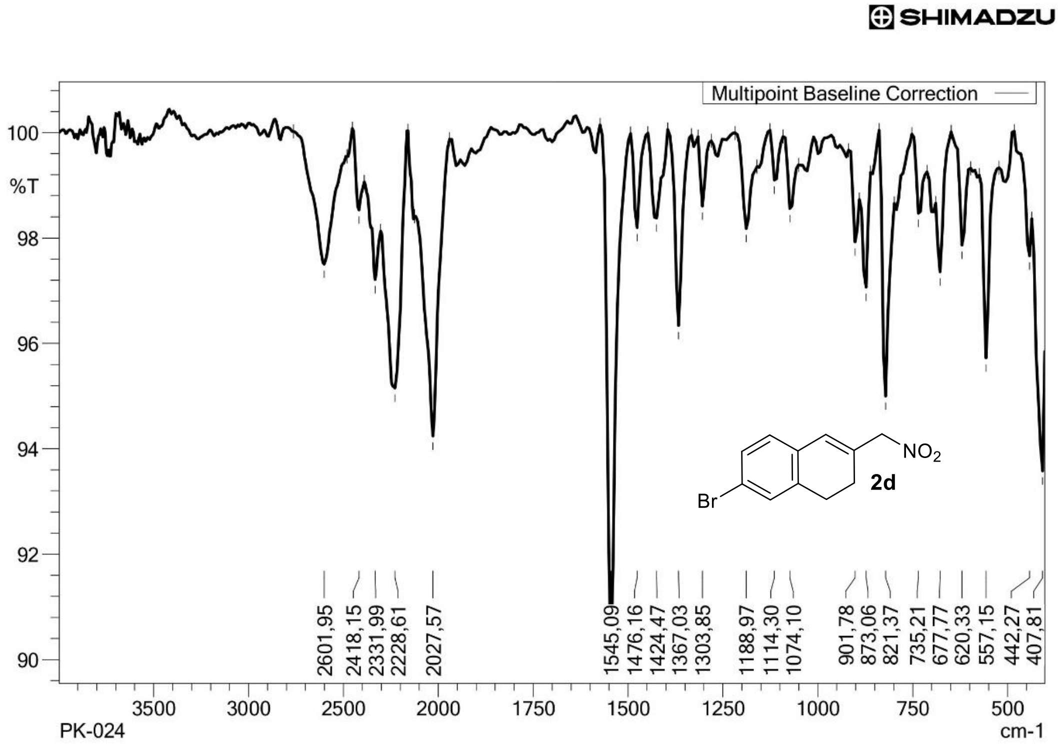

Figure S9. IR spectrum of purified product **2d**.

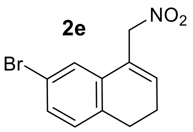
2e

6-bromo-4-(nitromethyl)-1,2-dihydronaphthalene (**2e**):

An adaption of the following procedure was used, see: Barton, D. H. R.; Fernandez, I.; Richard, C. S.; Zard, S. Z. A Mild Procedure for the Reduction of Aliphatic Nitro Compounds to Oximes. *Tetrahedron* **1987**, *43*, 551-558. To a clean round bottom flask (250 mL), equipped with an oval stirring bar and reflux condenser, flushed and maintained under N_2 was charged 7-bromo-1-tetralone (MW = 225.09 g/mol, 1.00 equiv, 30.00 mmol, 6.76 g), nitromethane (24.0 mL, 1.25 M), followed by ethylenediamine (MW = 60.10 g/mol, 5.0 mol%, 1.50 mmol, 90 mg, 100 µL, density = 0.90 g/mL). After 24 h under stirring at a gentle reflux, the reaction was cooled to room temperature and without further work-up was rotary evaporated to dryness, providing dark brown needle shaped crystals (no weight taken) which was purified by column chromatography. Reaction progress was monitored by TLC (product R_f = 0.38, 7-Br-1-tetralone R_f = 0.23; petroleum ether/dichloromethane, 65:35).

Purification and yield: Silica gel chromatography (21 cm in height, 5.5 cm in diameter) using isocratic elution (35 vol% CH_2Cl_2 in hexanes) allowed product isolation as an off-white solid (5.92 g, MW = 268.11 g/mol, 22.07 mmol, 74% yield). Note: A second reaction, albeit on a smaller scale (6.63 mmol of 7-Br-1-tetralone starting material) and less concentrated (CH_3NO_2, 0.66 M), lead to a 78% yield of **2e**.

R_f: 0.38 (petroleum ether/dichloromethane, 65:35).

^{1}H NMR (400 MHz, CDCl$_3$) (ppm) (Figure S10): δ 7.34-7.29 (m, 2H), 7.05 (d, 1H, J = 7.7 Hz), 6.38 (t, 1H, J = 4.6 Hz), 5.26 (s, 2H), 2.78 (t, 2H, J = 8.2 Hz), 2.43 (m, 2H).

^{13}C NMR (100 MHz, CDCl$_3$) (ppm) (Figure S11): δ 137.6, 134.8, 133.9, 130.7, 129.6, 126.8, 125.2, 120.3, 77.6, 26.7, 23.1.

HRMS (ESI-QTOF) m/z: Attempts to obtain an error measurement < 10 ppm failed.

IR (ATR, cm^{-1}) (Figure S12): *ν* = 3745, 2321, 2229, 2028, 1551, 1424, 821.

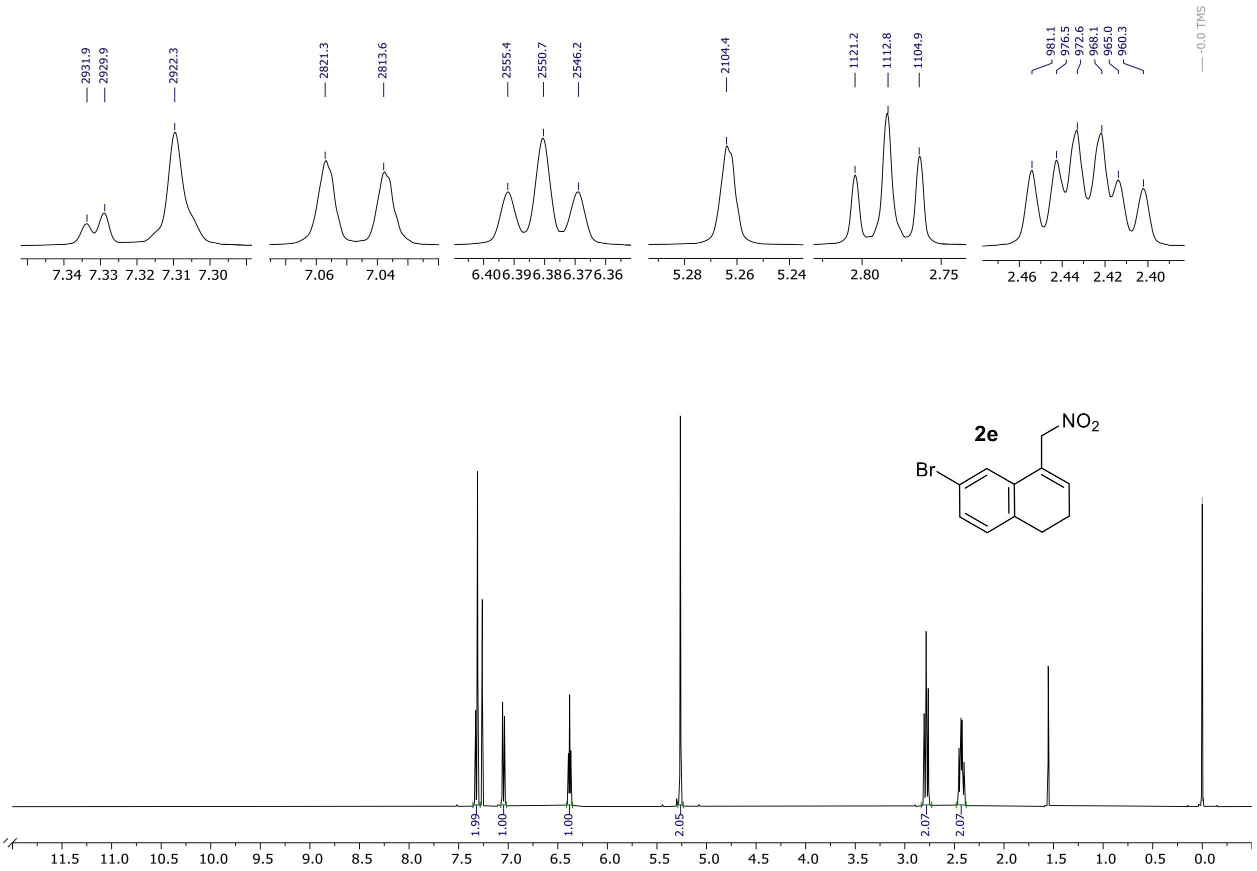

Figure S10. ^{1}H NMR spectrum of purified product **2e**.

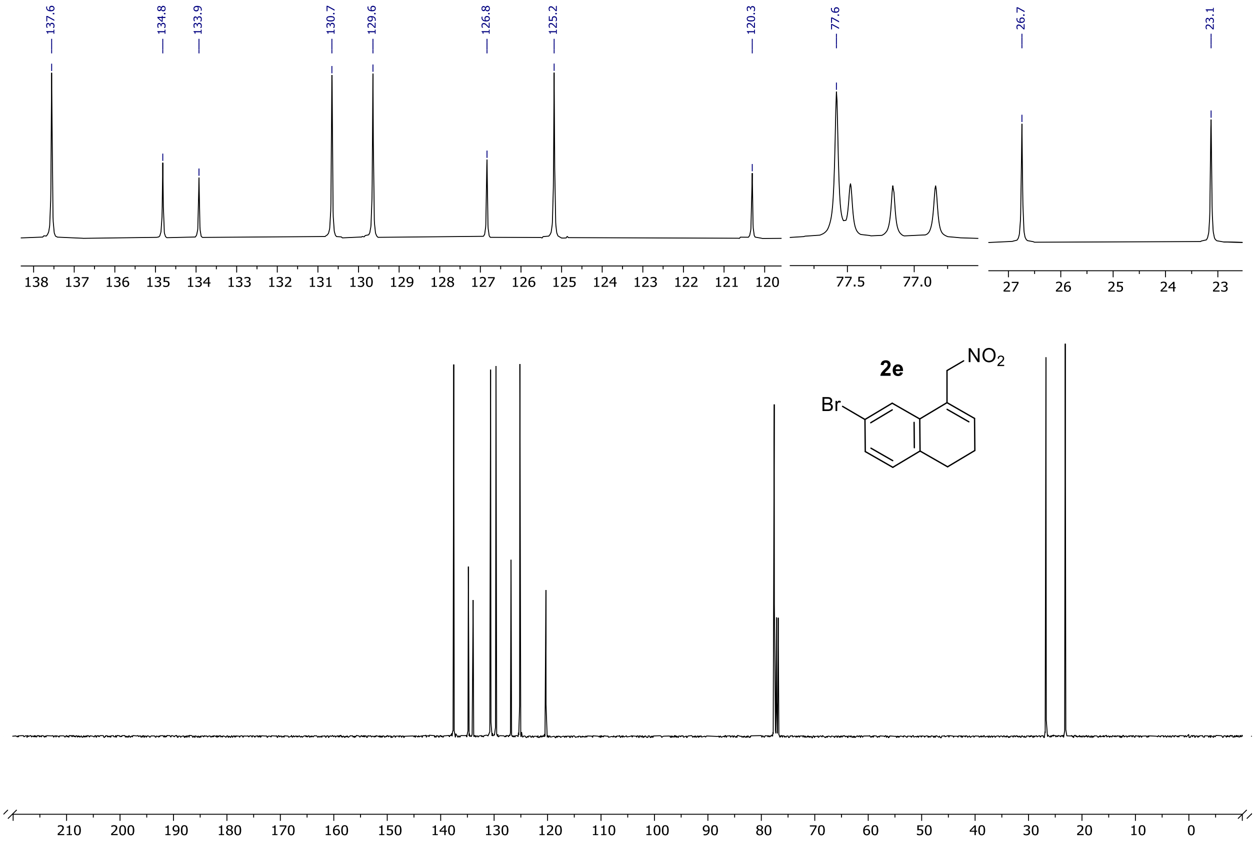

Figure S11. ^{13}C NMR spectrum of purified product **2e**.

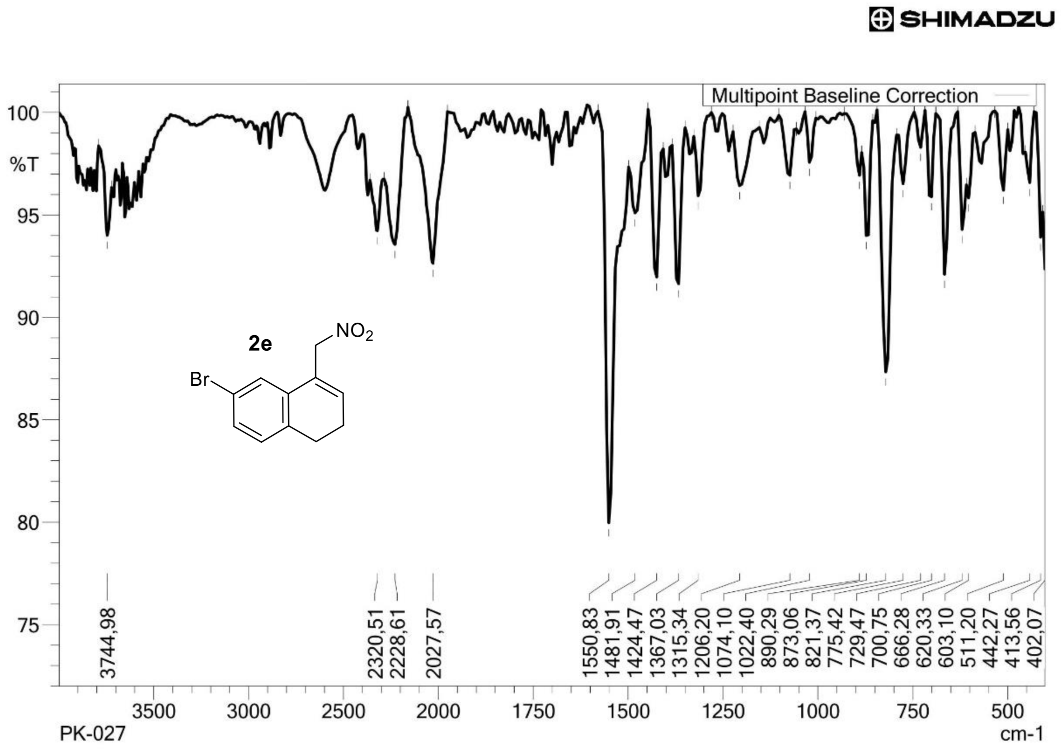

Figure S12. IR spectrum of purified product **2e**.

Section 5. Henry product reference standard synthesis and characterization **5a**, **b**, **c**, & **6a**, **b**, **c**

The *anti*- and *syn*-Henry products, respectively **5** and **6**, were either collected from multiple competition reactions (**5a**/**6a**) or intentionally synthesized (**5b**,**c**/**6b**,**c**), purified, and used as reference standards to confirm our crude ^{1}H NMR chemoselectivity assignments. These reactions were not optimized for yield or diastereoselectivity. Furthermore, we assume products **5** and **6** are racemic, however the possibility exists that they are enantioenriched because the catalyst that formed them is enantiopure. It is not a goal of this study to assess the enantiopurity of the Henry products noted here. The literature examples noted on this page show the *syn*-Henry product benzylic protons with larger coupling constants than the *anti*-Henry product benzylic protons and we have used this trend to assign the relative stereochemistry of our Henry products **5a**,**b**,**c** and **6a**,**b**,**c** as respectively *syn* or *anti*.

For examples of *syn*- *versus anti*-Henry product ^{1}H NMR coupling constant trends, -C**H**(NO_2)-C**H**(OH)Ar, see:

(i) Table 1 for compounds *erythro*-**Id** and *threo*-**Id** (*erythro* = *anti*, *threo* = *syn*) within: H. Lund, N. H. Nilsson, Electroorganic Preparations. XXXVII. Electrosynthesis of Cinnolines by Reductive Ring Closure of Dinitrocompounds, *Acta Chem. Scand. B* **1976**, *30*, 5-11. Therein the *syn*-Henry product benzylic coupling constant is assigned 9.5 Hz, while the *anti*-Henry product benzylic proton is assigned a 4.2 Hz coupling constant.

(ii) page S39 compound **2p**, the Henry product of nitroethane and 4-(trifluoromethyl)benzaldehyde, within the Supporting Information of: Mei, H.; Xiao, X.; Zhao, X.; Fang, B.; Liu, X.; Lin, L.; Feng, X. Catalytic Asymmetric Henry Reaction of Nitroalkanes and Aldehydes Catalyzed by a Chiral N,N′-Dioxide/Cu(I) Complex. *J. Org. Chem.* **2015**, *80*, 2272−2280. There the *anti*-Henry product benzylic proton of **2p** is assigned a 1.6 Hz coupling constant, while the *syn*-Henry product benzylic proton is assigned an 8.4 Hz coupling constant. Again, the *syn*-Henry product coupling constant is larger than the *anti*-Henry product.

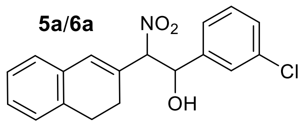

1-(3-chlorophenyl)-2-(3,4-dihydronaphthalen-2-yl)-2-nitroethan-1-ol (**5a** and **6a**):
Chromatographically isolated from several competition reactions.

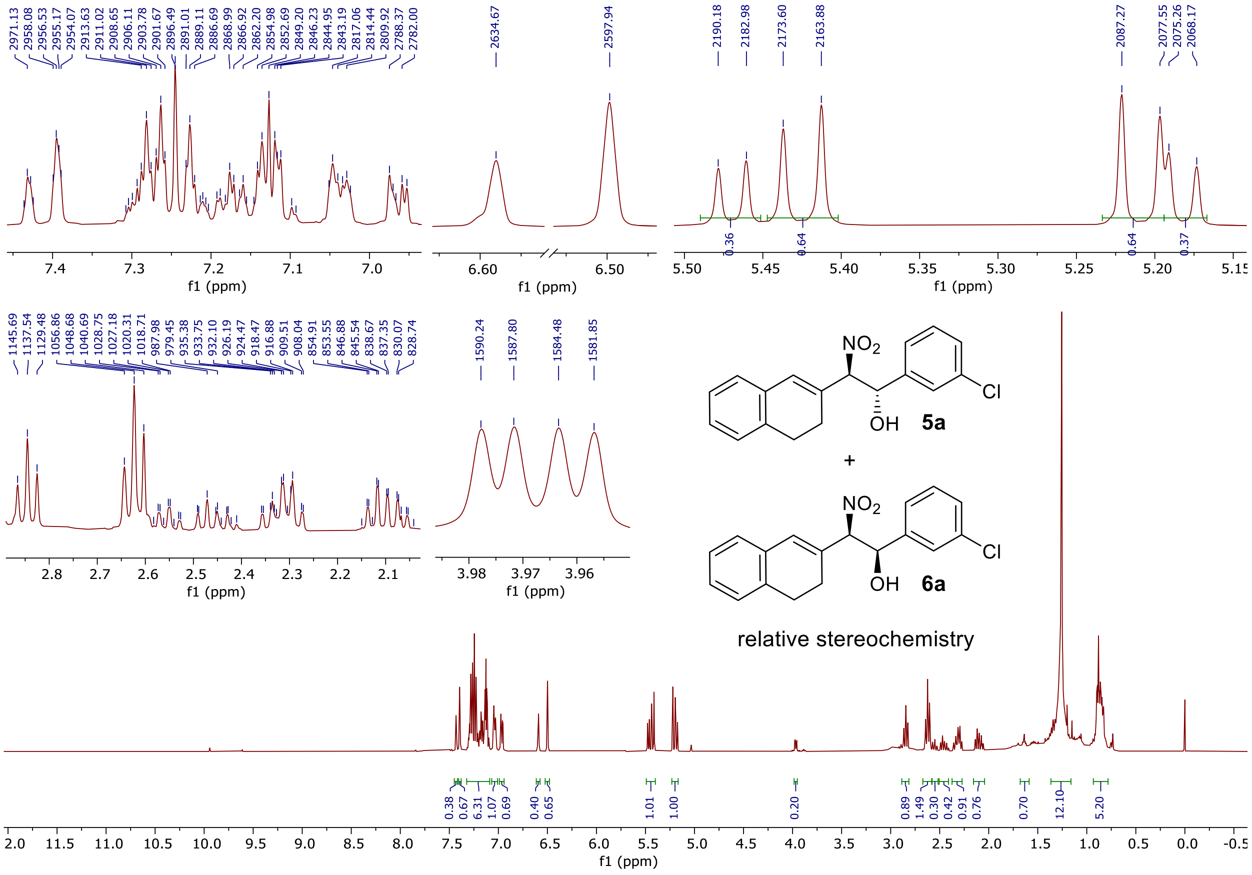

Figure S13. ^{1}H NMR of a mixture of **5a** and **6a**. These compounds were chromatographically inseparable using mixtures of EtOAc/petroleum.

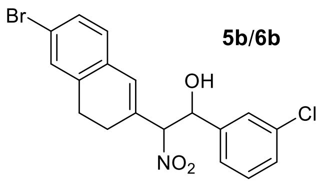

5b/6b

2-(6-bromo-3,4-dihydronaphthalen-2-yl)-1-(3-chlorophenyl)-2-nitro-ethan-1-ol (**5b** and **6b**):

To a clean, screw cap, V-shaped reaction vessel (2.0 mL) equipped with a small pyramidal stir bar, 7-bromo-3-(nitromethyl)-1,2-dihydronaphthalene (MW = 268.11 g/mol, 1.00 equiv, 0.7 mmol, 187.7 mg), 3-chloro-benzaldehyde (MW = 140.57 g/mol, 2.00 equiv, 1.4 mmol, 196.8 mg, 159 µL, density 1.241 g/mL) were added together. Stirring (5 min) provided a yellowish suspension and approximately 50% of the allylic nitro compound dissolved (visual inspection). *trans*-4-(*tert*-Butyldiphenylsilyloxy)-L-proline (MW = 369.54 g/mol, 5 mol%, 0.035 mmol, 13 mg) was added, followed within 30 sec the addition of deoxygenated distilled water (MW = 18.02 g/mol, 15.00 equiv, 10.54 mmol, 190 mg, 190 µL). The water was added such that minimal disruption of the concentrated organic layer occurred. The mixture was stirred such that the phase boundary was always gently agitated but at no point did the stirring allow any material to splash up and against the vessel walls. For observational purposes, the reaction was stopped after 5 min and two phases formed. The aqueous phase was colorless and clear, whereas the yellowish organic layer still contained ~50% of the undissolved allylic nitro compound. At 1 h no solids remained, but the organic phase appeared as a suspension when not stirred. The aqueous layer remained clear and colorless. TLC monitoring was performed at 3, 6, 24, 48, 72, and 96 h, but no TLC (UV-254 nm) change was noted for the limiting reactant (allylic nitro compound) during the last two observations so work-up was performed at 96 h.

Work-up: The reaction was transferred to a separatory funnel already containing water (50 mL) and CH_2Cl_2 (15 mL) using several portions of CH_2Cl_2 (3 x 3.0 mL). The CH_2Cl_2 was removed, and the aqueous phase was further extracted with CH_2Cl_2 (3 x 15 mL). The combined organic phases were dried over Na_2SO_4, filtered, concentrated (rotary evaporation), and high vacuum dried provided the crude product (326 mg).

Purification: The crude product was loaded onto a silica gel (230-400 mesh) column (16 cm in height, 2 cm in diameter) pre-wetted with CH_2Cl_2/petroleum ether (1:9) doped with acetic acid (1.0 vol%). Note: Chromatography without acetic acid resulted in partial reverse Henry reaction on the column. Mobile phase elution began with CH_2Cl_2/petroleum ether (1:9) doped with acetic acid (0.5 vol%). This solvent ratio was maintained until the starting materials were removed from the column. The *anti*-Henry product eluted after using CH_2Cl_2/petroleum ether (1:4) doped with acetic acid (0.5%) while the *syn* product eluted next using CH_2Cl_2/petroleum ether (1:3) doped with acetic acid (0.5%). Concentration of the pure fractions provided 30 mg (MW= 408.68 g/mol, 0.073 mmol, 10% yield) of the *anti*-Henry product as yellow liquid and 124 mg (MW= 408.68 g/mol, 124 mg, 0.303 mmol, 43% yield) of the *syn*-Henry product as white solid. Note: Instead of relying on rotary evaporation and high vacuum drying to remove the acetic acid, the combined column fractions can instead be washed with H_2O (2 x 50 mL) before rotary evaporation and high vacuum drying.

***anti*-Henry product (5b)**:

R_f = 0.15, CH_2Cl_2/petroleum ether (20:80) doped with acetic acid.

^{1}H NMR ($CDCl_3$ + D_2O, 400 MHz) (ppm) (Figures S14 & S15) δ 7.42 (s, 1H), 7.34 – 7.26 (m, 5H), 6.89 (d, *J* = 7.7 Hz, 1H), 6.50 (s, 1H), 5.50 (d, *J* = 6.7 Hz, 1H), 5.16 (d, *J* = 6.7 Hz, 1H), 2.82 (t, *J* = 8.1 Hz, 2H), 2.61 - 2.50 (m, 1H), 2.48 – 2.37 (m, 1H).

^{13}C NMR ($CDCl_3$, 100 MHz) (ppm) (Figure S16) δ 140.3, 137.5, 134.9, 131.8, 131.4, 130.7, 130.6, 130.2, 129.8, 129.3, 128.5, 126.9, 124.9, 122.1, 96.4, 72.4, 27.6, 24.

HRMS (ESI-QTOF) m/z: Attempts to obtain an error measurement < 10 ppm failed.

IR (ATR, cm^{-1}) (Figure S17) ν = 3435, 1551, 689.

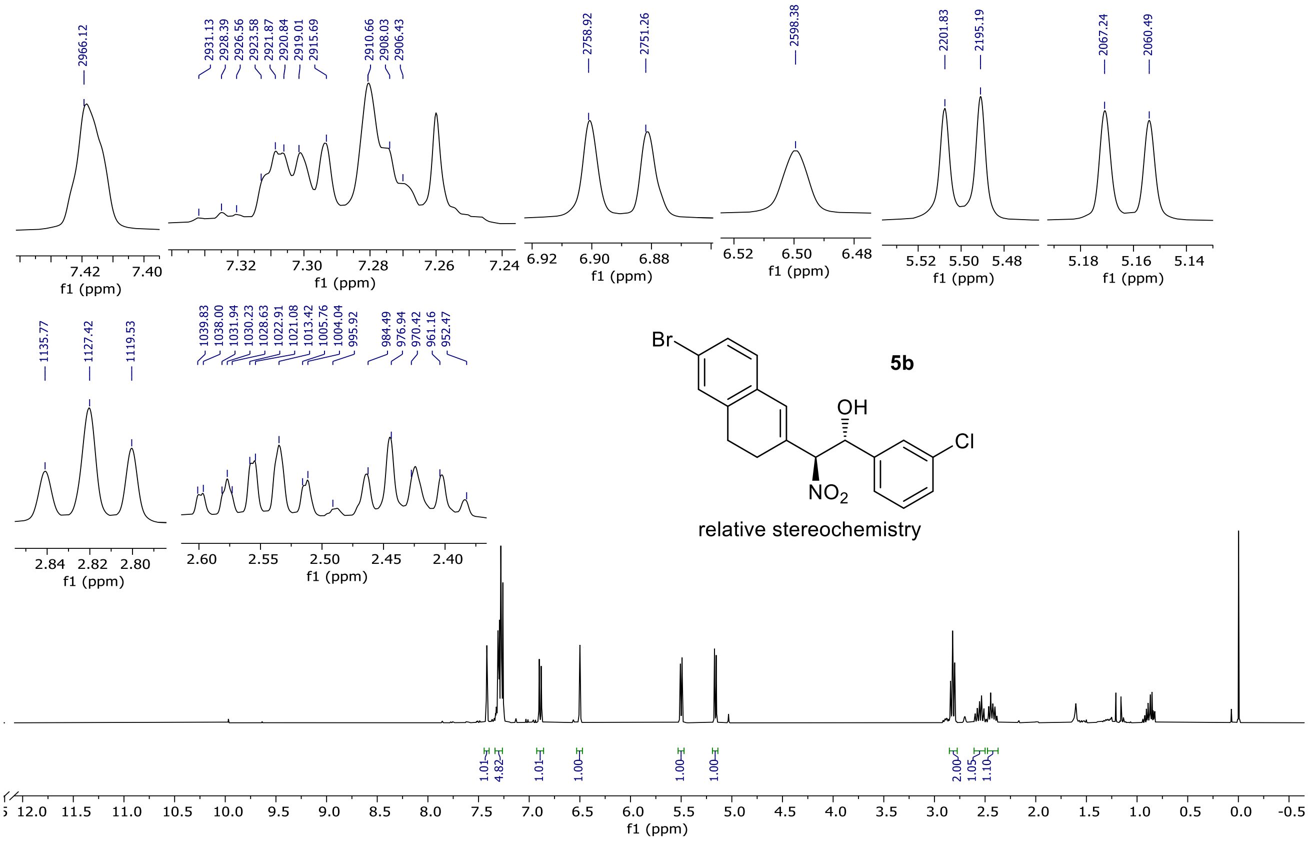

Figure S14. ^{1}H NMR spectrum of purified *anti*-Henry product **5b**: $CDCl_3$ *with* a drop of D_2O.

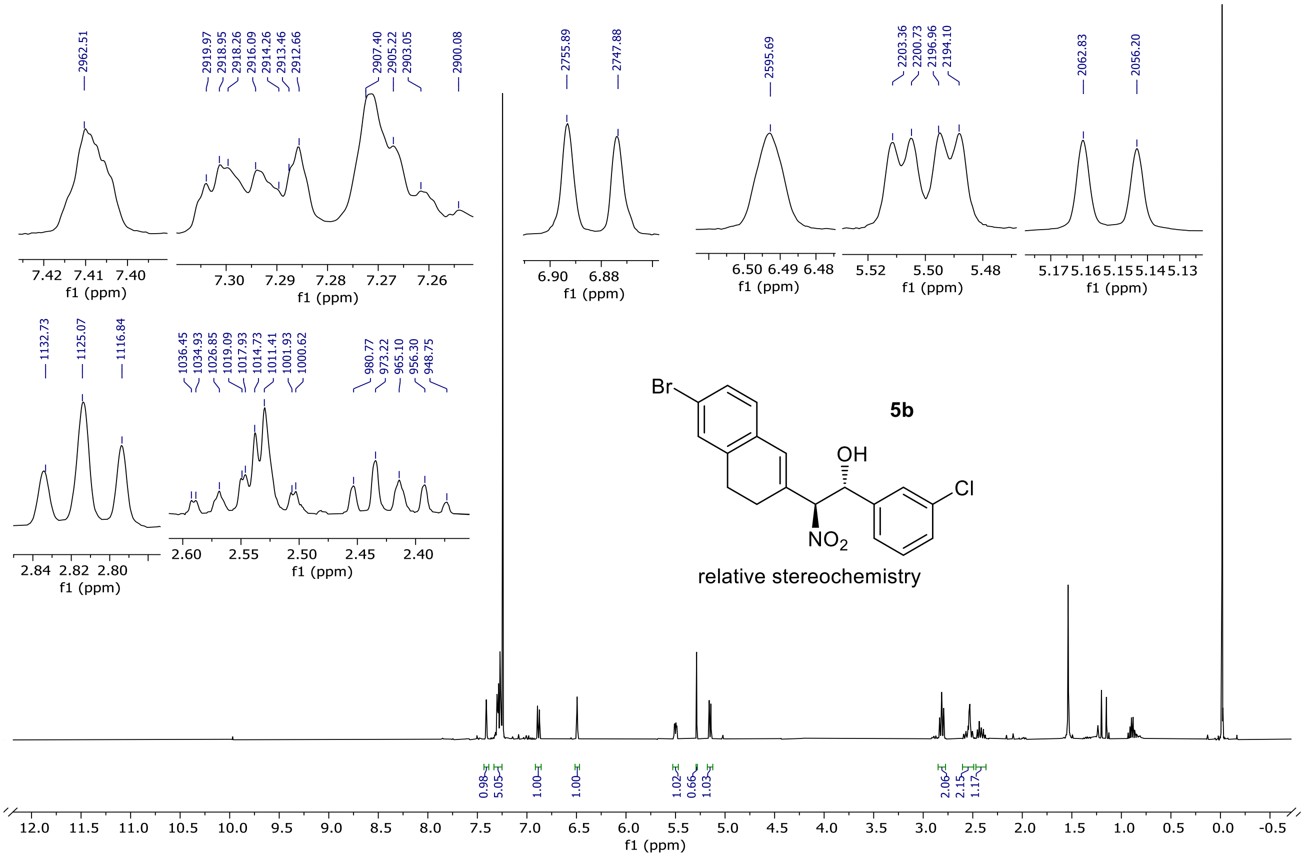

Figure S15. ^{1}H NMR spectrum of purified *anti*-Henry product **5b**: $CDCl_3$ *without* a drop of D_2O.

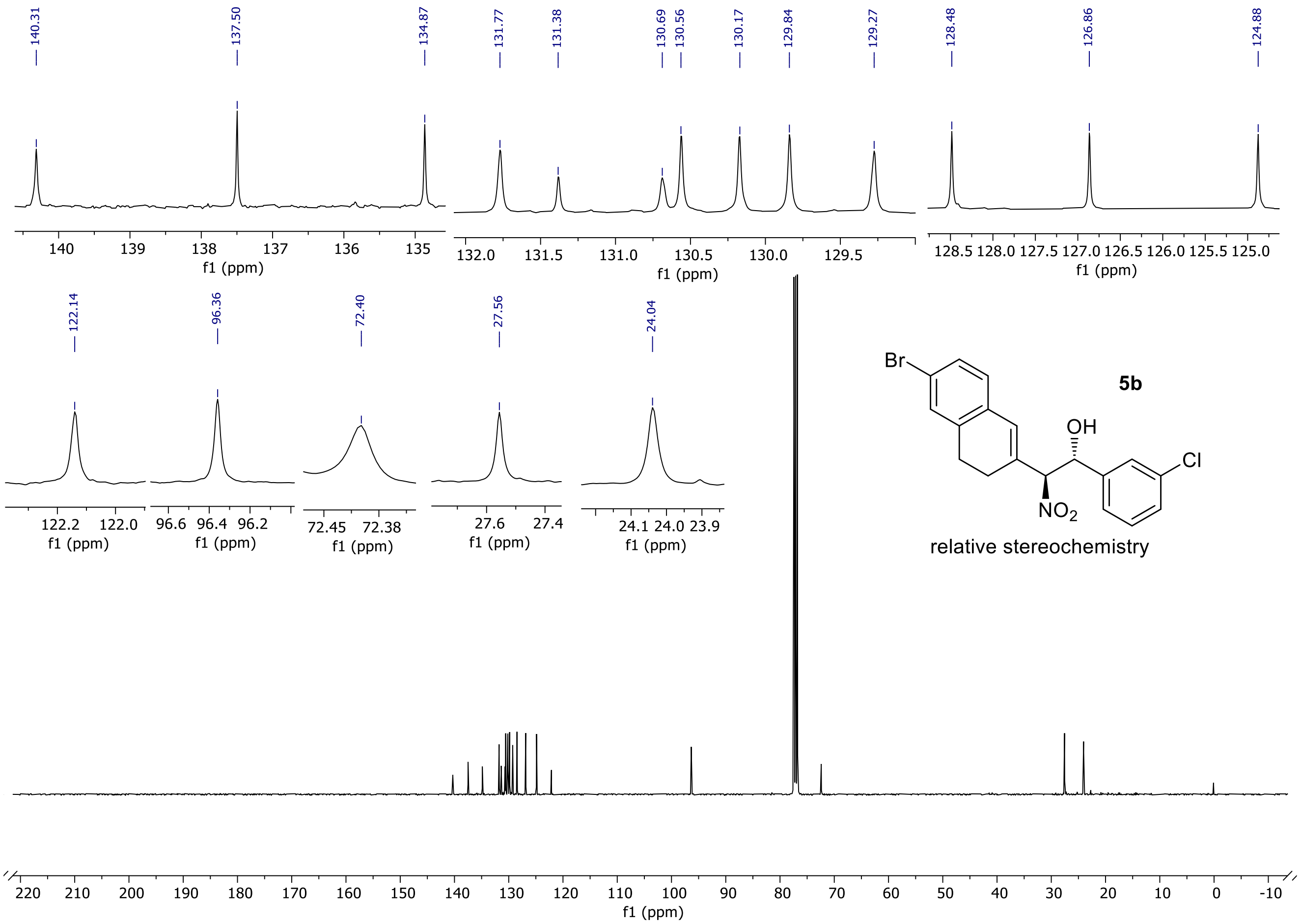

Figure S16. ^{13}C NMR spectrum of purified *anti*-Henry product **5b**: $CDCl_3$ *without* a drop of D_2O.

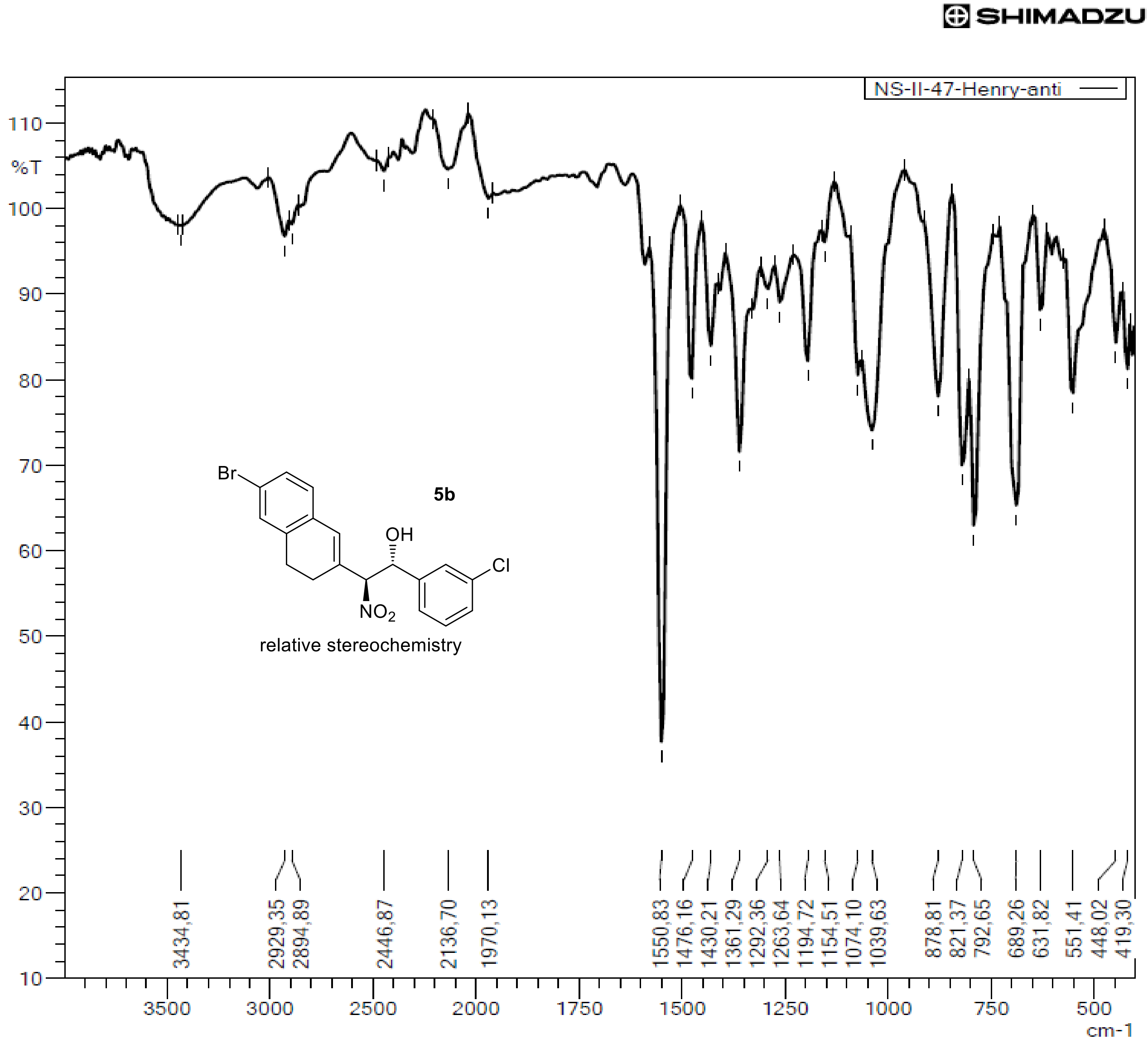

Figure S17. IR spectrum of purified *anti*-Henry product **5b**.

***syn*-Henry product** (**6b**):

R_f = 0.10, CH_2Cl_2/petroleum ether (20:80) doped with acetic acid.

Melting point: 58-60 °C

^{1}H NMR ($CDCl_3$ + D_2O, 400 MHz) (ppm) (Figures S18 & S19) δ 7.40 (s, 1H), 7.32 – 7.26 (m, 2H), 7.25 – 7.20 (m, 2H), 7.19 (s, 1H), 6.83 (d, *J* = 8.0 Hz, 1H), 6.46 (s, 1H), 5.42 (d, *J* = 9.6 Hz, 1H), 5.20 (d, *J* = 9.6 Hz, 1H), 2.61 (t, *J* = 8.1 Hz, 2H), 2.37 – 2.24 (m, 1H), 2.13 – 2.00 (m, 1H).

^{13}C NMR ($CDCl_3$, 100 MHz) (ppm) (Figure S20) δ 139.5, 136.9, 134.8, 131, 130.5, 130.4, 130.2, 130.1, 129.8, 129.4, 128.4, 127.2, 125.2, 122.1, 97.5, 73.6, 27.2, 24.2.

HRMS (ESI-QTOF) m/z: Attempts to obtain an error measurement < 10 ppm failed.

IR (ATR, cm^{-1}) (Figure S21) **ν =** 3486, 1591, 689.

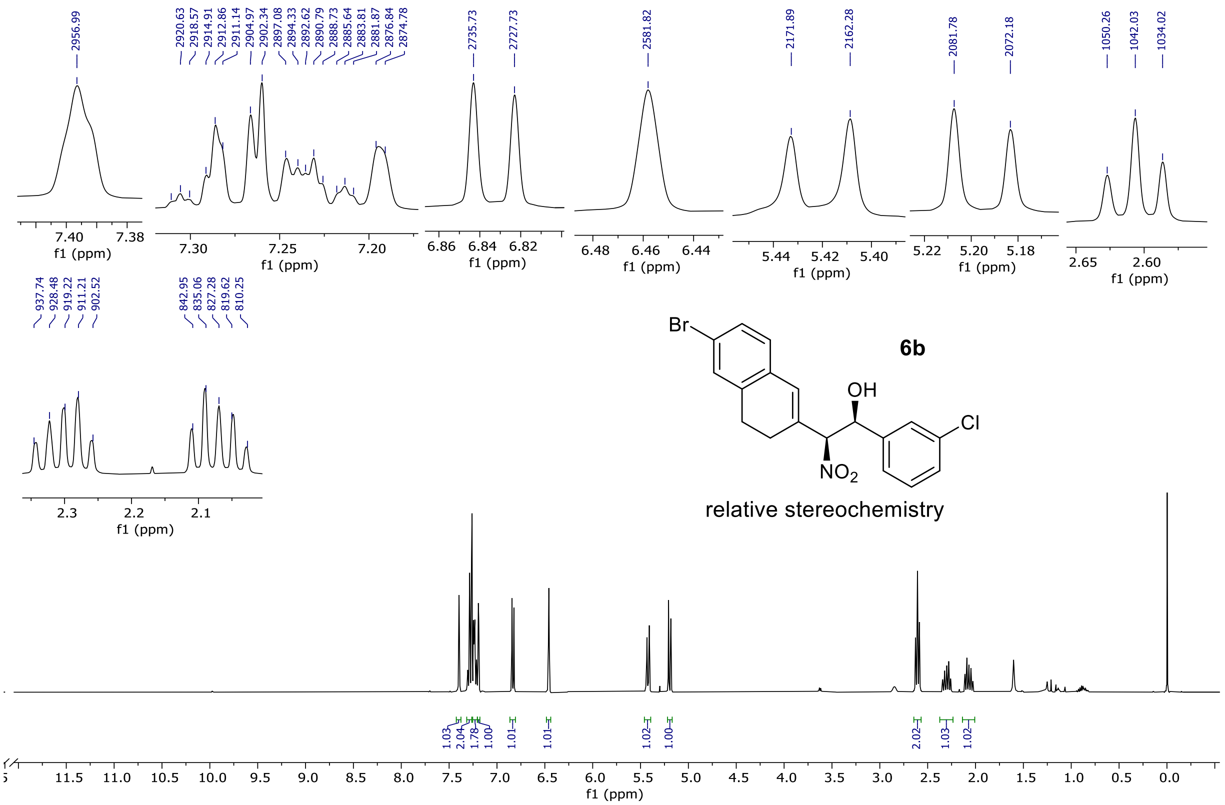

Figure S18. ^{1}H NMR spectrum of purified *syn*-Henry product **6b**: $CDCl_3$ *with* a drop of D_2O.

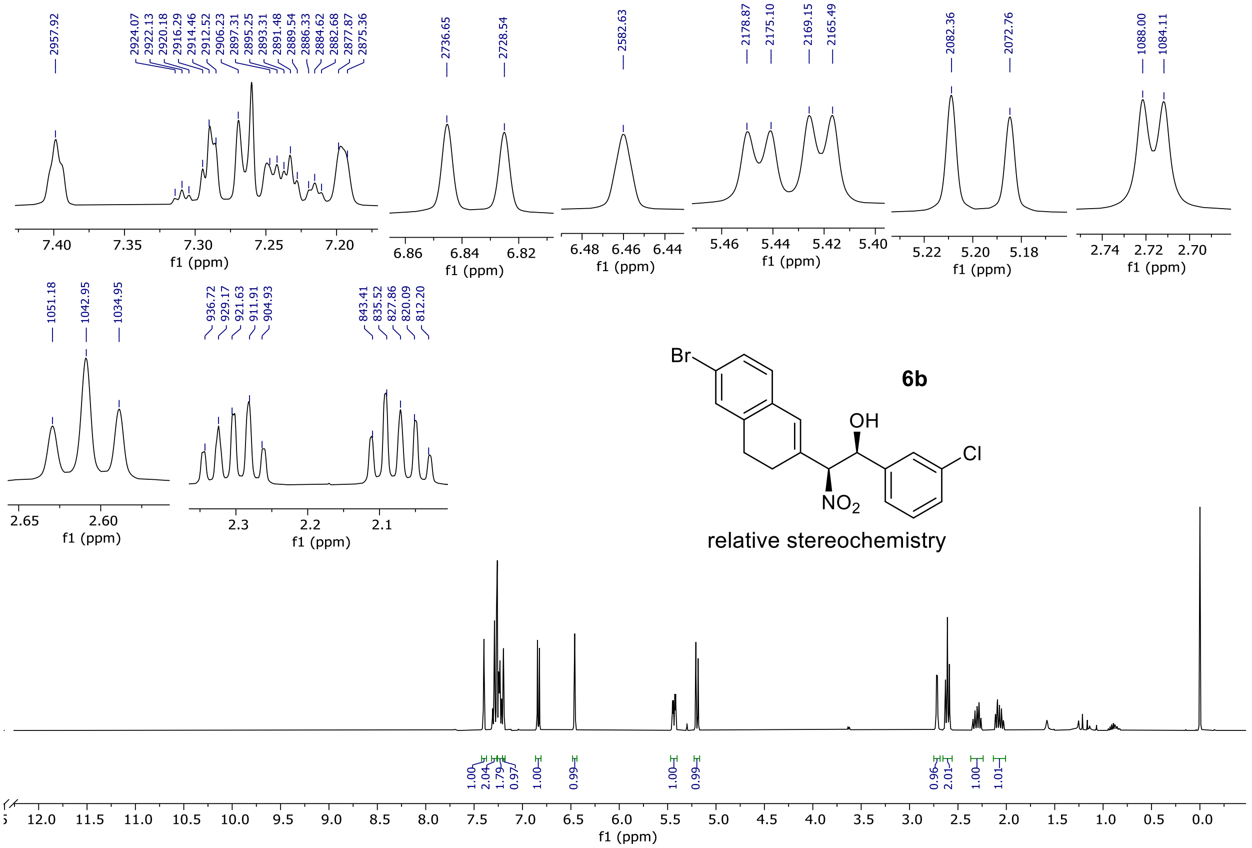

Figure S19. ^{1}H NMR spectrum of purified *syn*-Henry product **6b**: $CDCl_3$ *without* a drop of D_2O.

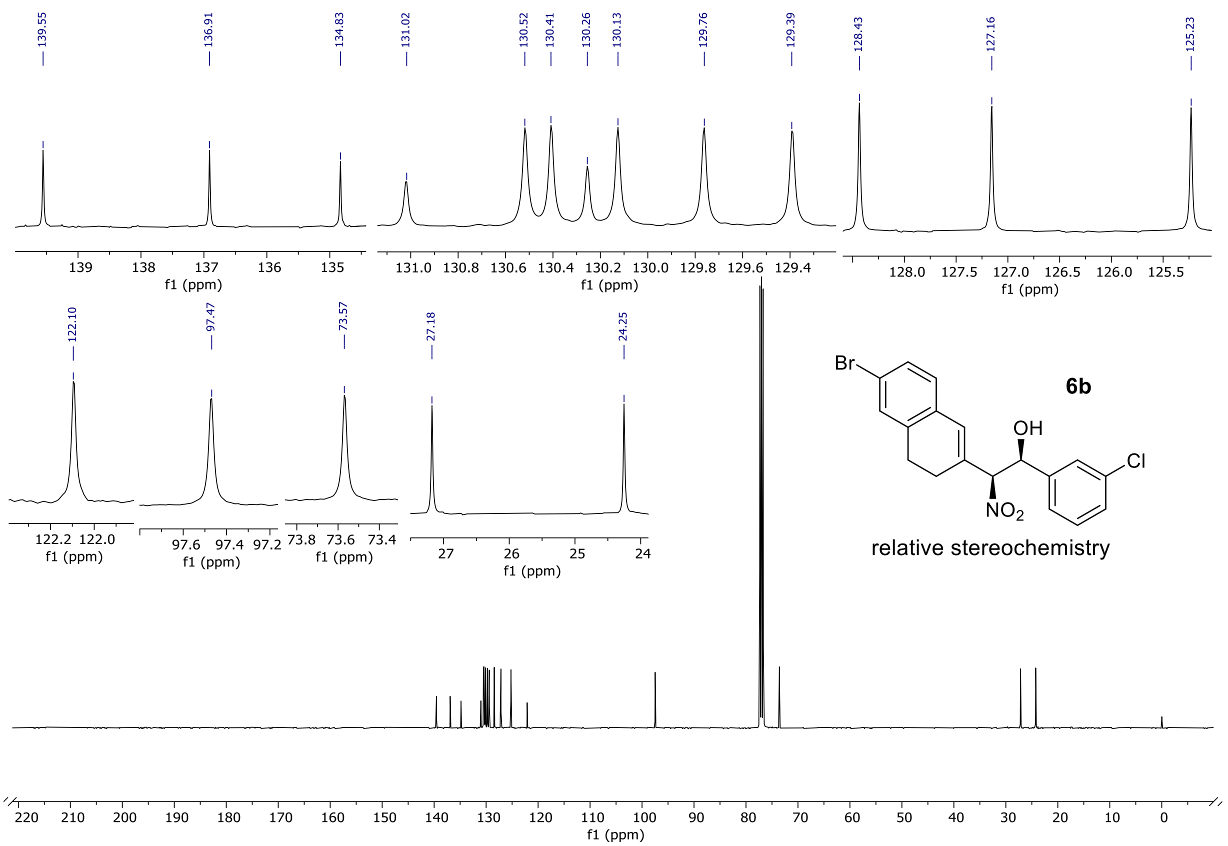

Figure S20. ^{13}C NMR spectrum of purified *syn*-Henry product **6b**: $CDCl_3$ *without* a drop of D_2O.

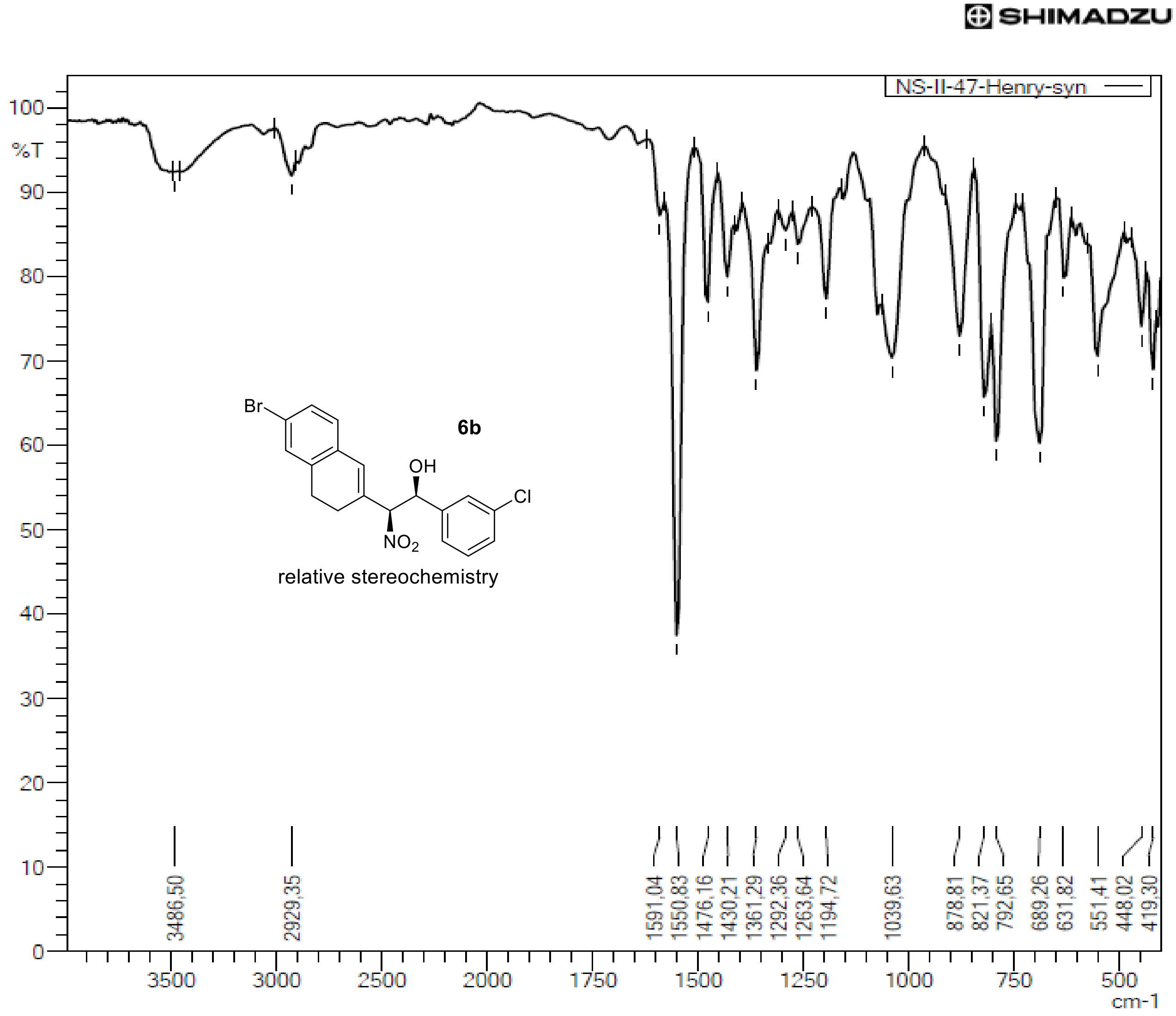

Figure S21. IR spectrum of purified *syn*-Henry product **6b**.

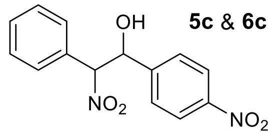

5c & **6c**

2-nitro-1-(4-nitrophenyl)-2-phenylethan-1-ol:

To a clean, screw cap, V-shaped reaction vessel (2.0 mL) equipped with a small pyramidal stir bar, mortar and pestle ground 4-nitrobenzaldehyde (MW = 151.12 g/mol, 1.00 equiv, 1.50 mmol, 226.7 mg), and (nitromethyl)benzene (MW = 137.14 g/mol, 1.97 equiv, 2.96 mmol, 405.2 mg, 350 µL, density 1.16 g/mL) were added. The heterogeneous mixture was allowed to gently stir for 5 min and visual inspection showed approximately 50% dissolution of the 4-nitrobenzaldehyde. Stirring was terminated and *trans*-4-(tert-butyldiphenylsilyloxy)-L-proline (MW = 369.54 g/mol, 5.00 mol%, 0.075 mmol, 27.7 mg) was added and followed by immediatel addition of deoxygenated distilled water (MW = 18.02 g/mol, 15.0 equiv, 22.5 mmol, 405.5 mg, 405.5 µL). The resulting heterogeneous solution was stirred such that the contents of the vessel did not splash against the vessel walls but allowed gentle phase boundary agitation. After 30 min the stirring was stopped, two phases formed, and visual inspection showed approximately 25% of the solid material continued to remain undissolved. After 1 h, the concentrated organic phase was a paste and remained so until work-up. Reaction monitoring by TLC (UV-254 nm) was performed at 4, 16, 24, 48, and 72 h, but the last two observations showed no significant change to the limiting reactant (4-nitrobenzaldehyde) so the work-up was performed at 72 h.

Work-up: The reaction was transferred to a separatory funnel already containing water (30 mL) and CH_2Cl_2 (15 mL) using several portions of CH_2Cl_2 (3 x 3 mL). The CH_2Cl_2 phase was removed from the separatory funnel, and the aqueous phase was further extracted with CH_2Cl_2 (3 x 20 mL). The combined organic phases were dried over Na_2SO_4, filtered, and concentrated under rotary evaporation. High vacuum drying provided the crude product (490 mg).

Purification: Silica gel chromatography (16 cm in height, 2 cm in diameter) was performed with a pre-wetted column in EtOAc/petroleum ether (1:19) doped with acetic acid (1.0 vol%). Elution began with EtOAc/petroleum ether (1:19) doped with acetic acid (0.5 vol%). This solvent ratio was maintained until the starting materials were removed from the column. The solvent polarity was then increased to EtOAc/petroleum ether (1:9) doped with acetic acid (0.5 vol%) at which point the *syn*-Henry product eluted out first followed by the *anti*-Henry product. Concentration, followed by high vacuum drying of the pure fractions provided 226.1 mg (MW= 288.26 g/mol, 0.784 mmol, 52% yield) of the *syn*-Henry product as yellow solid and 146 mg (MW= 288.26 g/mol, 0.506 mmol, 34% yield) of the *anti*-Henry product as pale yellow solid.

***anti*-Henry product (5c)**:

R_f = 0.21 EtOAc/petroleum ether (1:3) doped with acetic acid.

Melting point: 122-124 °C

^{1}H NMR (CDCl$_3$, 400 MHz) (ppm) (Figure S22) δ 8.19 (d, *J* = 8.86 Hz, 2H), 7.57 – 7.48 (m, 2H), 7.48 – 7.39 (m, 5H), 5.75 (d, *J* = 6.51 Hz, 1H), 5.54 (d, *J* = 6.52 Hz, 1H), 2.78 (br s, 1H).

^{13}C NMR (CDCl$_3$, 100 MHz) (ppm) (Figure S23) δ148.2, 144.9, 130.7, 130.1, 129.3, 129.1, 127.9, 123.9, 94.8, 73.7.

HRMS (ESI-QTOF) m/z: Attempts to obtain an error measurement < 10 ppm failed.

IR (ATR, cm^{-1}) (Figure S24) **ν =** 3418, 1550, 1522, 1344.

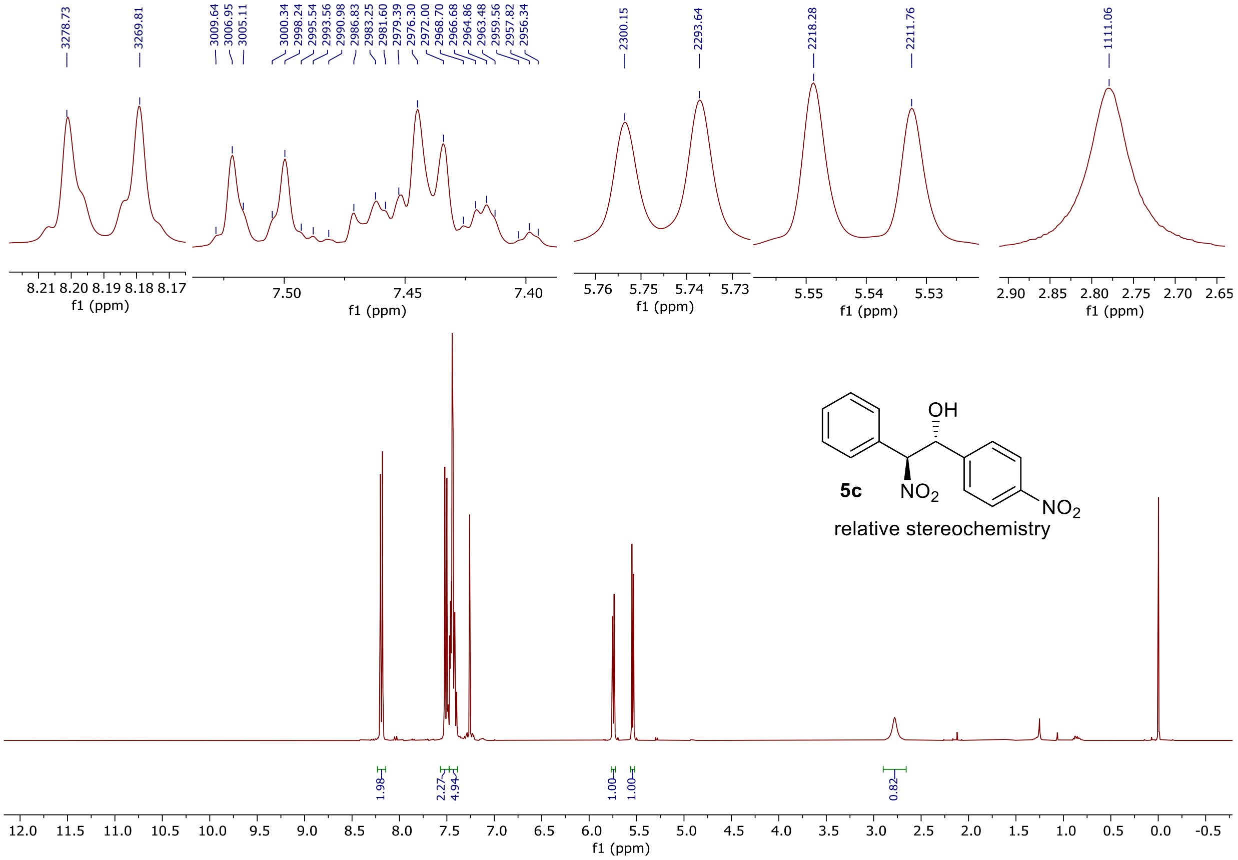

Figure S22. ^{1}H NMR spectrum of purified *anti*-Henry product **5c**.

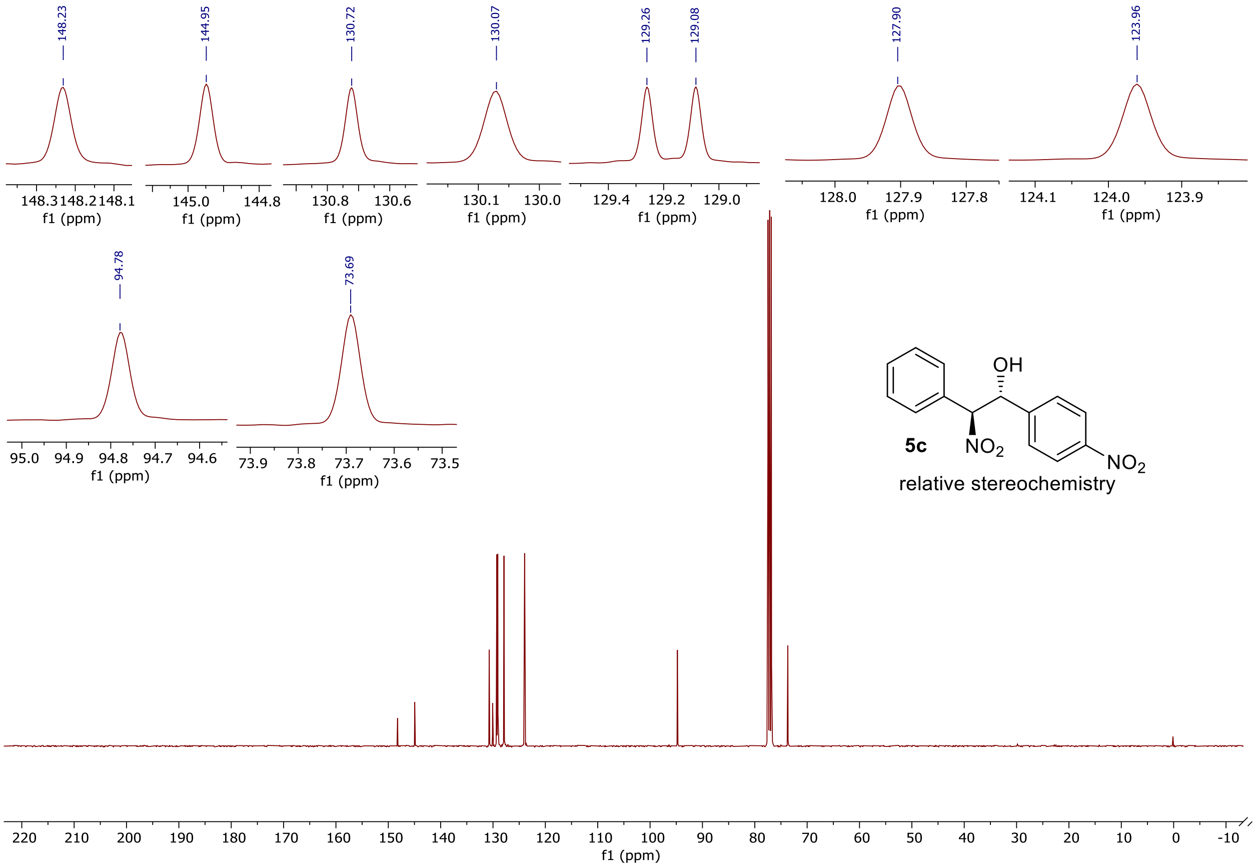

Figure S23. ^{13}C NMR spectrum of purified *anti*-Henry product **5c**.

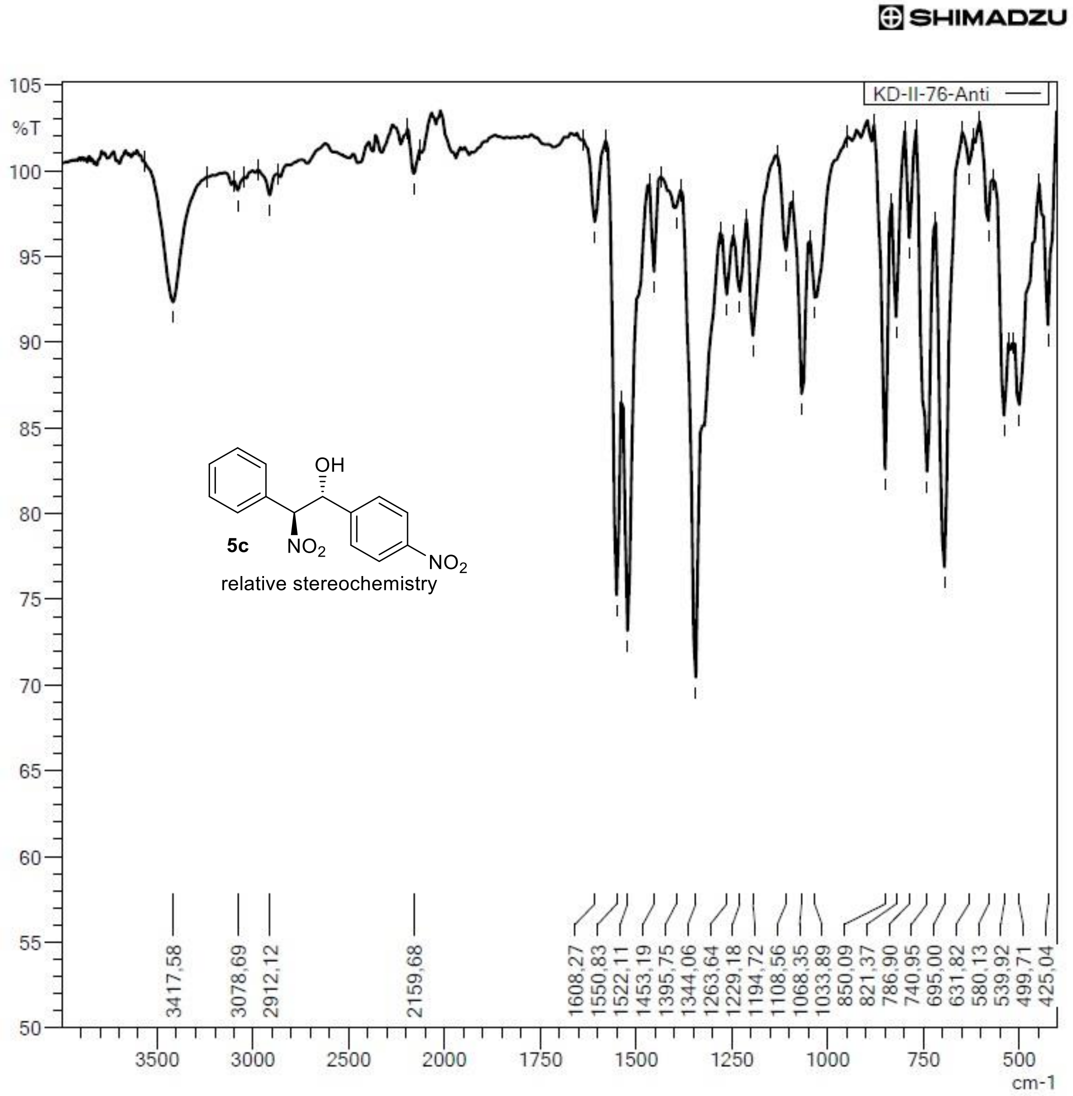

Figure S24. IR spectrum of purified *anti*-Henry product **5c**.

***syn*-Henry product** (**6c**):

R_f = 0.24 EtOAc/petroleum ether (1:3) doped with acetic acid.

Melting point: 56-58 °C

^{1}H NMR ($CDCl_3$, 400 MHz) (ppm) (Figure S25) δ 7.99 (d, *J* = 8.8 Hz, 2H), 7.38 – 7.22 (m, 7H), 5.73 (d, *J* = 9.76 Hz, 1H), 5.55 (d, *J* = 10.02 Hz, 1H), 3.43 (br s, 1H)

^{13}C NMR ($CDCl_3$, 100 MHz) (ppm) (Figure S26) δ 147.8, 144.8, 130.6, 130.5, 129.3, 128.2, 128.1, 123.6, 96.3, 74.9

HRMS (ESI-QTOF) m/z: Attempts to obtain an error measurement < 10 ppm failed.

IR (ATR, cm^{-1}) (Figure S27) **ν** = 3521, 1551, 1516, 1344

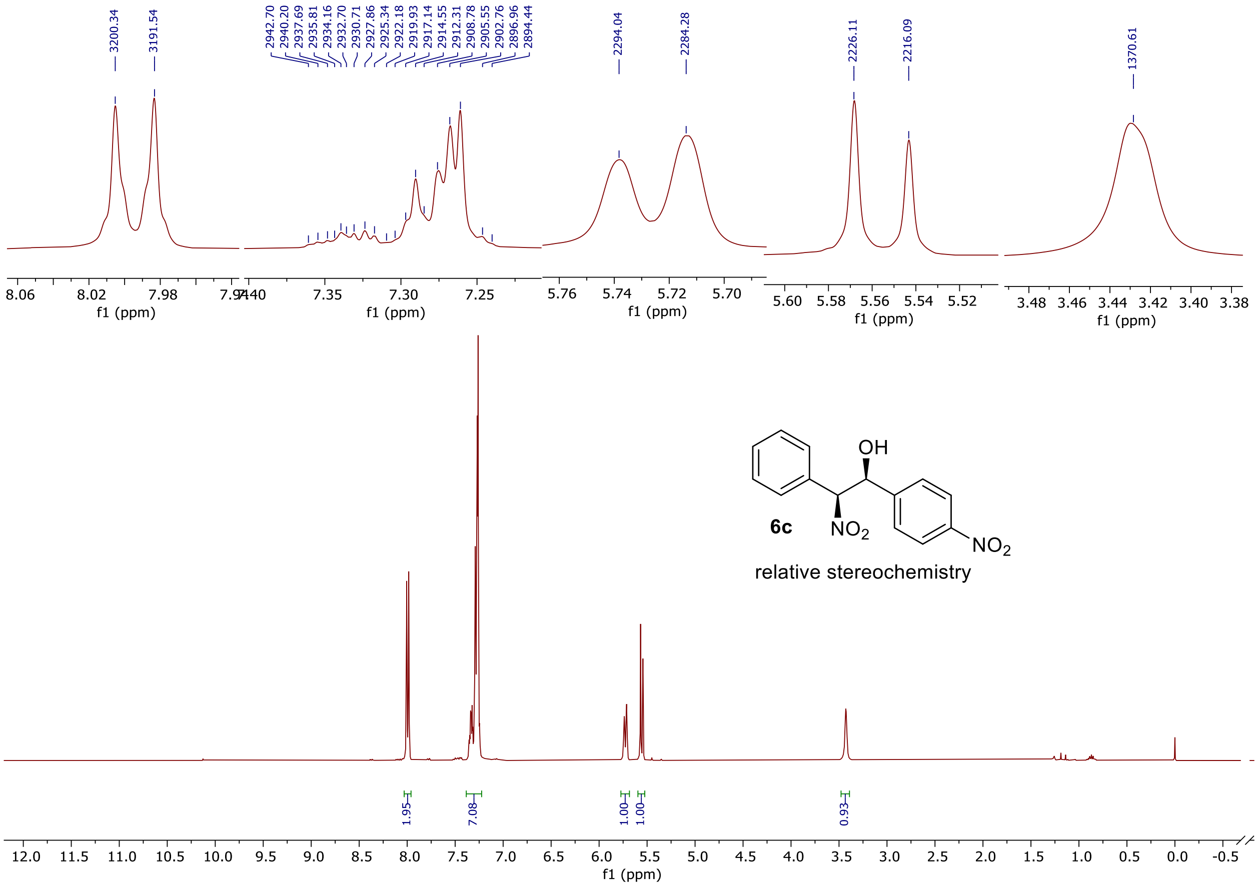

Figure S25. ^{1}H NMR spectrum of purified *syn*-Henry product **6c**.

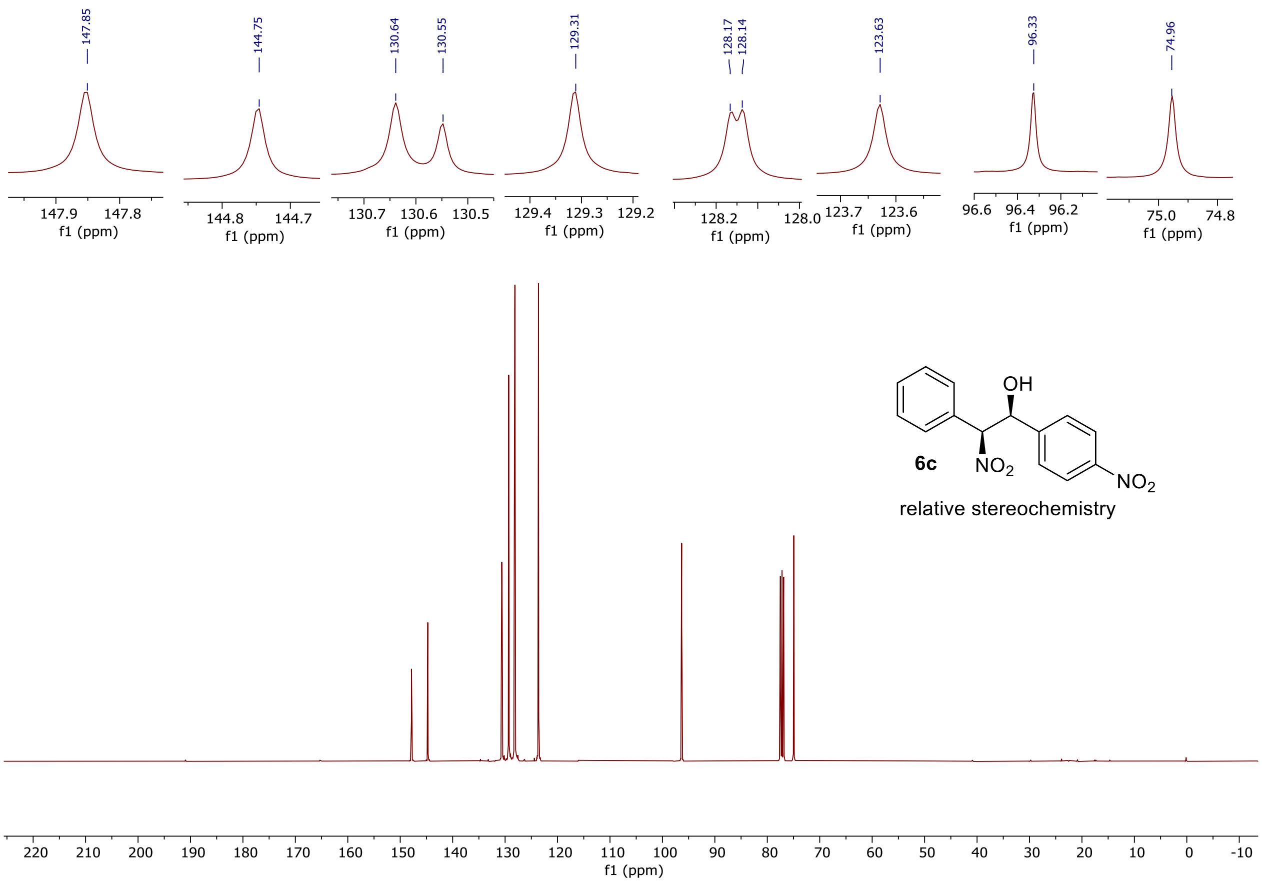

Figure S26. ^{13}C NMR spectrum of purified *syn*-Henry product **6c**.

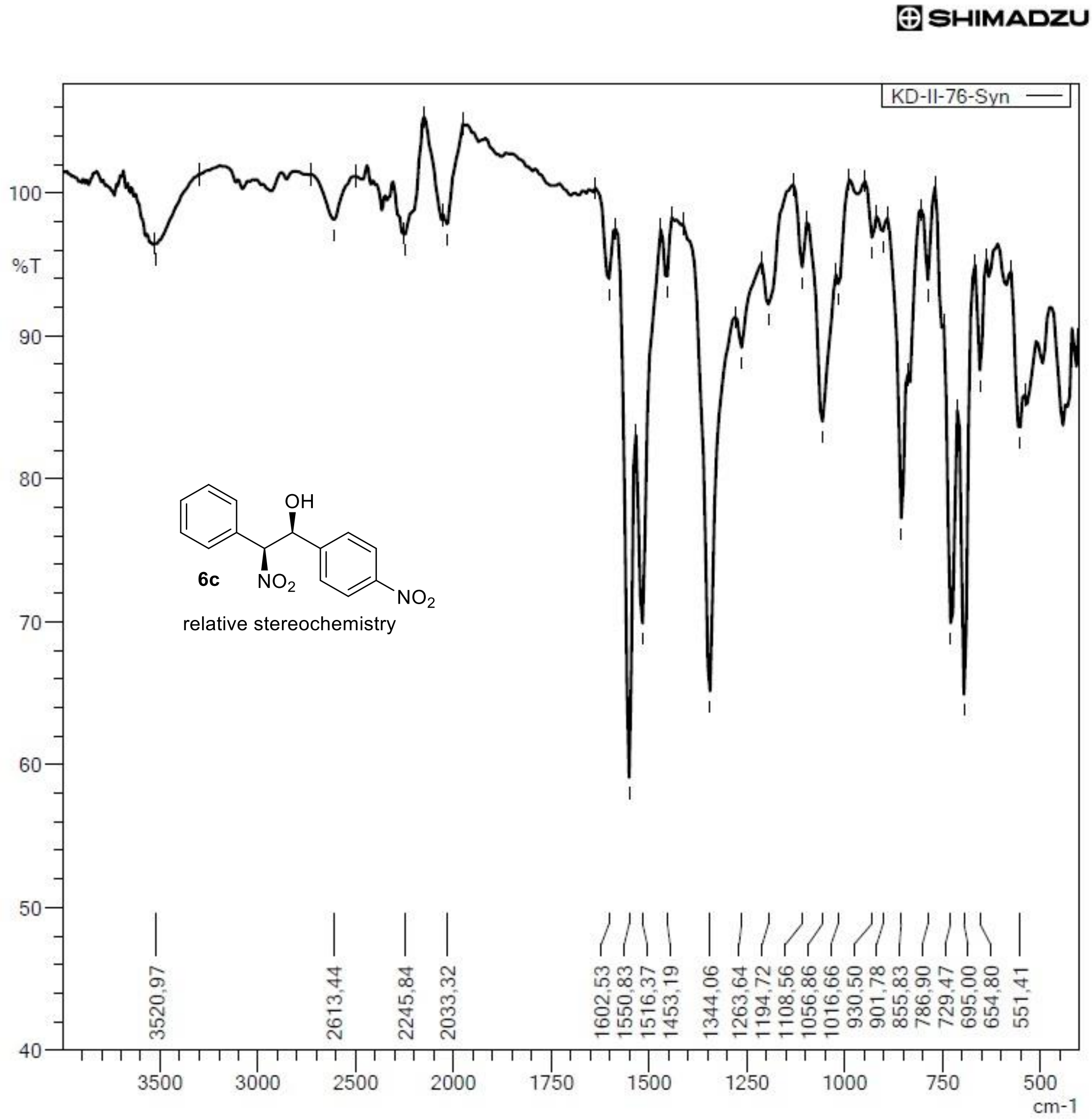

Figure S27. IR spectrum of purified *syn*-Henry product **6c**.

Section 6. Aldol racemate formation

The cobalt dichloride inspired methods came from the work of Reiser, see: Karmakar, A.; Maji, T.; Wittmann, S.; Reiser, O. L-Proline/$CoCl_2$-Catalyzed Highly Diastereo- and Enantioselective Direct Aldol Reactions. *Chem. Eur. J.* **2011**, *17*, 11024–11029. Note that while he calls for the use anhydrous $CoCl_2$ and MeOH, we did not always rigorously exclude moisture. Despite this we did perform these reactions under N_2. Our point, these reactions are moisture sensitive and while they tolerate some moisture, too much does lead to failed reactions.

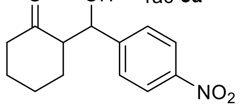

rac-**3a**

2-Picolyamine (MW= 108.14, 20 mol%, 0.53 mmol, 57.3 mg, density= 1.049 g/mL, 54.6 uL), 4-nitrobenzaldehyde (MW= 151.12, 1 equiv, 2.65 mmol, 400.5 mg), and cyclohexanone (MW= 98.14, 3 equiv, 7.95 mmol, 780.2 mg, density= 0.947 g/mL, 823.9 uL) were added to a mixture of MeOH (2.7 mL) and H_2O (2.7 mL). Time = 20 h, no yield recorded.

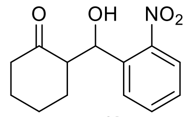

rac-**3b**

2-Picolyamine (MW= 108.14, 40 mol%, 1.20 mmol, 129.77 mg, density = 1.049 g/mL, 123.7 uL), 2-nitrobenzaldehyde (MW= 151.12, 1 equiv, 3.0 mmol, 453.36 mg), and cyclohexanone (MW= 98.14, 3.0 equiv, 9.0 mmol, 883.26 mg, density = 0.947 g/mL, 933.7 uL) were added to a mixture of MeOH (4.0 mL) and H_2O (0.5 mL). Time = 42 h, no yield recorded.

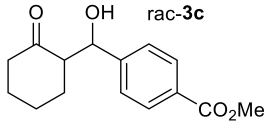

rac-**3c**

Anhydrous cobalt dichloride (MW = 129.84 g/mol, 10 mol%, 0.1 mmol, 12.9 mg), L-proline (MW = 115.13, 0.1 equiv, 0.1 mmol, 11.5 mg), D-proline (MW = 115.13, 0.1 equiv, 0.1 mmol, 11.5 mg), were added to methanol (100 µL), followed by cyclohexanone (MW = 98.14, 3.0 equiv, 3.0 mmol, density = 0.947 g/mL, 311 µL), methyl 4-formylbenzoate (MW = 164.16, 1.0 equiv, 1.0 mmol, 164.2 mg). Time = 46 h, yield = 48%.

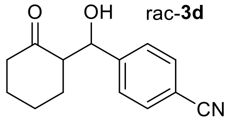

rac-**3d**

2-Picolyamine (MW= 108.14, 40 mol%, 1.2 mmol, 129.77 mg, density= 1.049 g/mL, 124 uL), 4-formylbenzonitrile (MW= 131.13, 1 equiv, 3.00 mmol, 393.39 mg), and cyclohexanone (MW= 98.14, 3 equiv, 9.00 mmol, 883.35 mg, density= 0.947 g/mL, 933uL) were added to a mixture of MeOH (4.0 mL) and H_2O (0.5 mL). Time = 43 h, no yield recorded

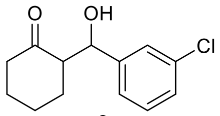

rac-**3e**

2-Picolyamine (MW= 108.14, 40 mol%, 1.2 mmol, 129.77 mg, density= 1.049 g/mL, 124 uL), 3-chlorobenzaldehyde (MW= 140.57, 1 equiv, 3.00 mmol, 421.71 mg, density=1.241 g/mL, 340 uL), and cyclohexanone (MW= 98.14, 3 equiv, 9.00 mmol, 883.35 mg, density= 0.947 g/mL, 933 uL) were added to a mixture of MeOH (3.0 mL) and H_2O (3.0 mL). Time = 22 h, no yield recorded

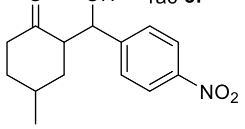

rac-**3f**

Anhydrous cobalt dichloride (MW = 129.84, 10 mol%, 0.1 mmol, 12.9 mg), L-proline (MW = 115.13, 10 mol%, 0.1 mmol, 11.5 mg), D-proline (MW = 115.13, 10 mol%, 0.1 mmol, 11.5 mg), methanol (101.1 µL), followed by 4-methylcyclohexanone (MW= 112.17, 3.0 equiv, 3.0 mmol, 336.5 mg, density= 0.914 g/mL, 368.2 µL) and 4-nitrobenzaldehyde (MW = 151.12, 1.0 equiv, 1.0 mmol, 151.1 mg) . Time = 24 h, 48% yield.

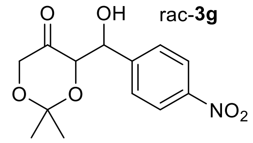

rac-**3g**

Anhydrous cobalt dichloride (MW = 129.84, 10 mol%, 0.1 mmol, 12.8 mg), L-proline (MW = 115.13, 0.1 mmol, 11.5 mg), D-proline (115.13, 0.1 mmol, 11.5 mg), and methanol (101 µL), followed by 2,2-dimethyl-1,3-dioxan-5-one (MW = 130.14, 3.0 mmol, 358 µL) and 4-nitrobenzaldehyde (MW = 151.12, 1.00 mmol, 151.12 mg). Time = 96 h, yield = 51%.

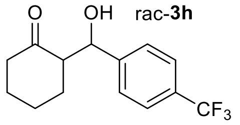

rac-**3h**

Anhydrous cobalt dichloride (MW = 129.84, 10 mol%, 0.3 mmol, 38.95 mg), L-proline (MW = 115.13, 10 mol%, 0.3 mmol, 34.54 mg), D-proline (MW = 115.13, 10 mol%, 0.3 mmol, 34.54 mg), and methanol (101.1 µL), followed by cyclohexanone (MW= 98.14, 3.0 equiv, 9.0 mmol, 883.3 mg, density= 0.947 g/mL, 932.7 µL) and 4-(trifluoromethyl)benzaldehyde (MW = 261.18, 1.0 equiv, 3.0 mmol, 783.5 mg, density= 1.275 g/mL, 614.5 µL). Time = 45 h, no yield recorded.

Section 7. Table 2 entries 1-10 experimental descriptions and characterization of *anti*-aldol products **3a**, **b**, **c**, **d**, **e**, **f**, **g**

Characterization: All aldol products synthesized in this study have been previously characterized and often by many different researchers. The references we cite here are ones where clear experimental data was provided and included an HPLC chromatogram and often a ^{1}H NMR spectrum.

Table 2, entry 1: Competition reaction between cyclohexanone and nitromethane for the limiting reactant 4-nitrobenzaldehyde

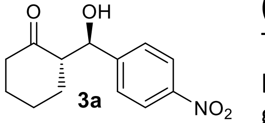

(*S*)-2-((*R*)-hydroxy(4-nitrophenyl)methyl)cyclohexan-1-one (**3a**):
To a clean, screw cap, V-shaped reaction vessel (5.0 mL) equipped with a small pyramidal stir bar, mortar and pestle ground 4-nitrobenzaldehyde (MW = 151.12 g/mol, 1.00 equiv, 1.50 mmol, 226.7 mg), cyclohexanone (MW = 98.14 g/mol, 1.50 equiv, 2.25 mmol, 220.84 mg, 233 µL, density 0.948 g/mL), and nitromethane (MW = 61.04 g/mol, 3.00 equiv, 4.5 mmol, 274.68 mg, 243 µL, density 1.132 g/mL) were added. The liquid reactants (cyclohexanone and nitromethane) were used to rinse the solid 4-nitrobenzaldehyde off the walls as needed. The heterogeneous mixture was allowed to gently stir for < 5 min, but visual inspection showed little or no dissolution of the 4-nitrobenzaldehyde. Next, the stirring was terminated and *trans*-4-(tert-butyldiphenylsilyloxy)-*L*-proline (MW = 369.54 g/mol, 2.5 mol%, 0.0375 mmol, 13.9 mg) was added. Distilled deoxygenated water (MW = 18.02 g/mol, 15.00 equiv, 22.5 mmol, 405.45 mg, 406 µL) was added within 30 sec. The resulting heterogeneous solution was gently stirred such that the contents of the vessel did not splash against the vessel walls. Undissolved solids remained until about 6 h into the reaction, at which point both layers became transparent. The rate of stirring only gently agitated the phase boundary and the reaction was quenched at 24 h. See Section 2 for the work-up procedure.

This compound was previously synthesized and characterized, see below.

Crude product ^{1}H NMR analysis (Figure S28, see below) allowed determination of the diastereo- and chemoselectivity ratios.

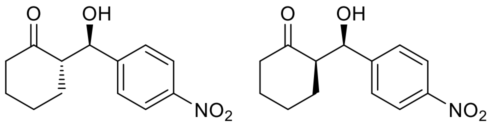

anti-aldol **3a** (major) *syn*-aldol **4a** (minor)

Diastereoselectivity: The *anti-/syn*-aldol ratio was determined as >19:1 from the benzylic proton resonances at 4.89 ppm (d, *anti*-aldol product) and 5.48 ppm (d, *syn*-aldol product) in the crude ^{1}H NMR spectrum (Figure S28). For the literature chemical shift values, see page S2 (product 3a) of the Supporting Information within: Mase, N.; Nakai, Y.; Ohara, N.; Yoda, H.; Takabe, K.; Tanaka, F.; Barbas, C. F., III. Organocatalytic Direct Asymmetric Aldol Reactions in Water. *J. Am. Chem. Soc.* **2006**, *128*, 734–735.

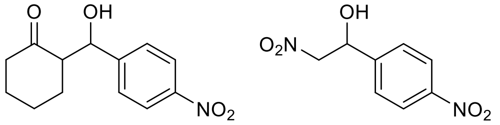

aldol products **3a**/**4a** Henry products

Chemoselectivity: The *anti*- and *syn*-aldol/Henry product ratio was determined to be >19:1, based on a lack of observable resonance patterns for the Henry product at 5.60 ppm (dd, benzylic proton) or at 4.59 ppm (dd, CH_2NO_2), see Figure S28. For related Henry product chemical shift values see page 9854 within: Pandey, S.; Bansal, D.; Gupta, R. A Metalloligand Appended with Benzimidazole Rings: Tetranuclear [$CoZn_3$] and [$CoCd_3$] Complexes and Their Catalytic Applications. *New J. Chem.* **2018**, *42*, 9847–9856.

Purification and yield: Silica gel chromatography (36 mm column outer diameter, 18 cm silica bed height) was performed using gradient elution (10 to 15 vol% EtOAc in petroleum ether). The crude product was loaded onto the column in a minimum volume of CH_2Cl_2. Gradient elution was used (10 to 15 vol% EtOAc in petroleum ether) and the products were collected when using 15 vol% EtOAc in petroleum ether. After concentration (rotary evaporation) and high vacuum drying, a yellow solid weighing 340.0 mg (MW = 249.27 g/mol, 1.364 mmol, 91% yield) of the *anti*- and *syn*-aldol diastereomers was isolated.

TLC: *anti*-aldol product R_f = 0.28; *syn*-aldol product R_f = 0.36 EtOAc/petroleum ether (3:7).

99% *ee*: Chiralcel OD-H chiral HPLC column, *i*PrOH/n-hexane (7:93), flow rate = 1.0 mL/min, λ = 254 nm, injection volume = 20 μL, the sample was dissolved in 10 vol% *i*PrOH/n-hexane with a concentration of ≈ 1 mg/mL; *anti*-aldol product t_{major} = 21.4 min, t_{minor} = 32.1 min retention times were observed (Figure S30 and S31).

^{1}H NMR (400 MHz, $CDCl_3$) (ppm) *anti*-aldol product **3a** (Figure S29): δ 8.20 (d, 2H, J = 8.8 Hz), 7.50 (d, 2H, J = 8.6 Hz), 4.89 (dd, 1H, J = 8.4, 3.0 Hz), 4.09 (d, 1H, J = 3.2 Hz), 2.63-2.54 (m, 1H), 2.53-2.45 (m, 1H), 2.41-2.31 (m, 1H), 2.15-2.07 (m, 1H), 1.86-1.78 (m, 1H), 1.75-1.48 (m, 4H), 1.43-1.32 (m, 1H).

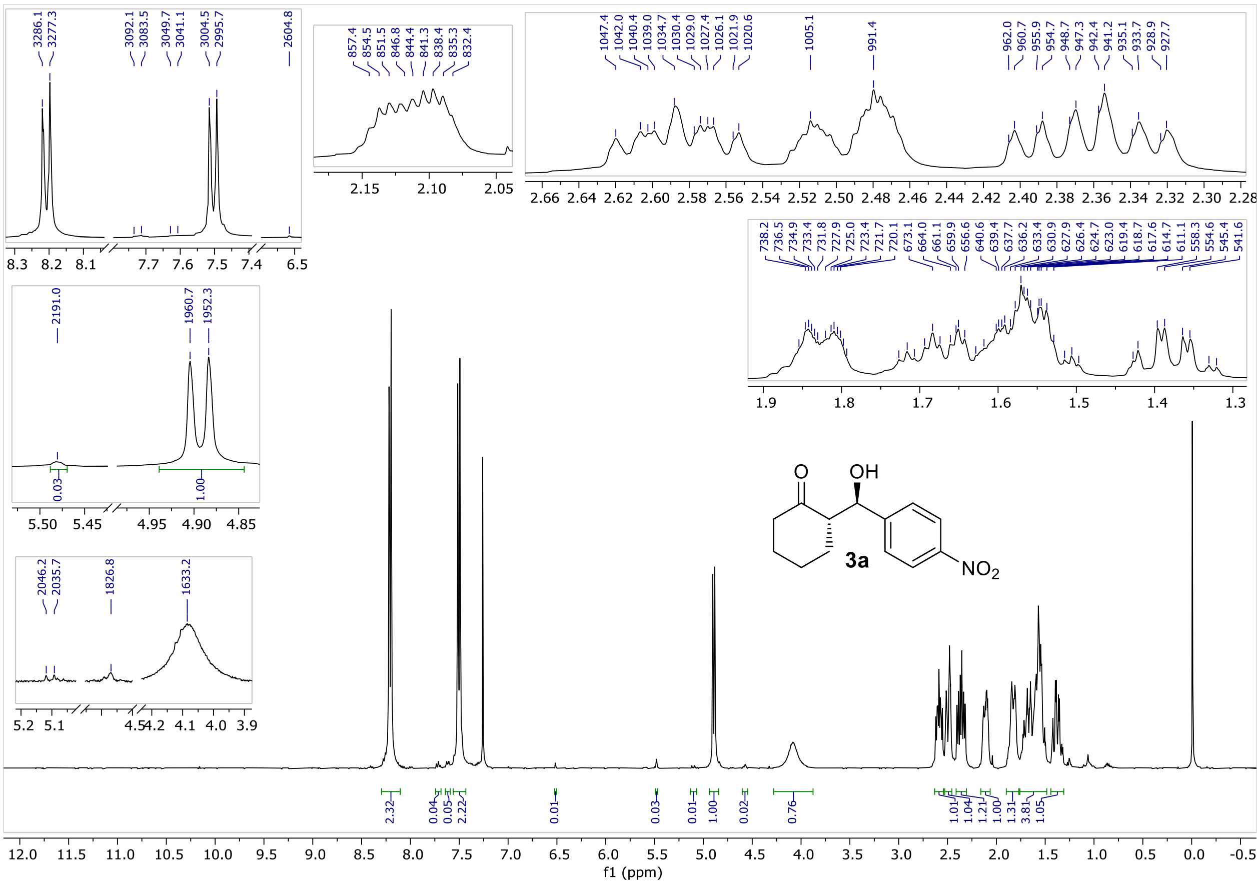

Figure S28. Crude ^{1}H NMR spectrum after high vacuum drying of **3a** (above).

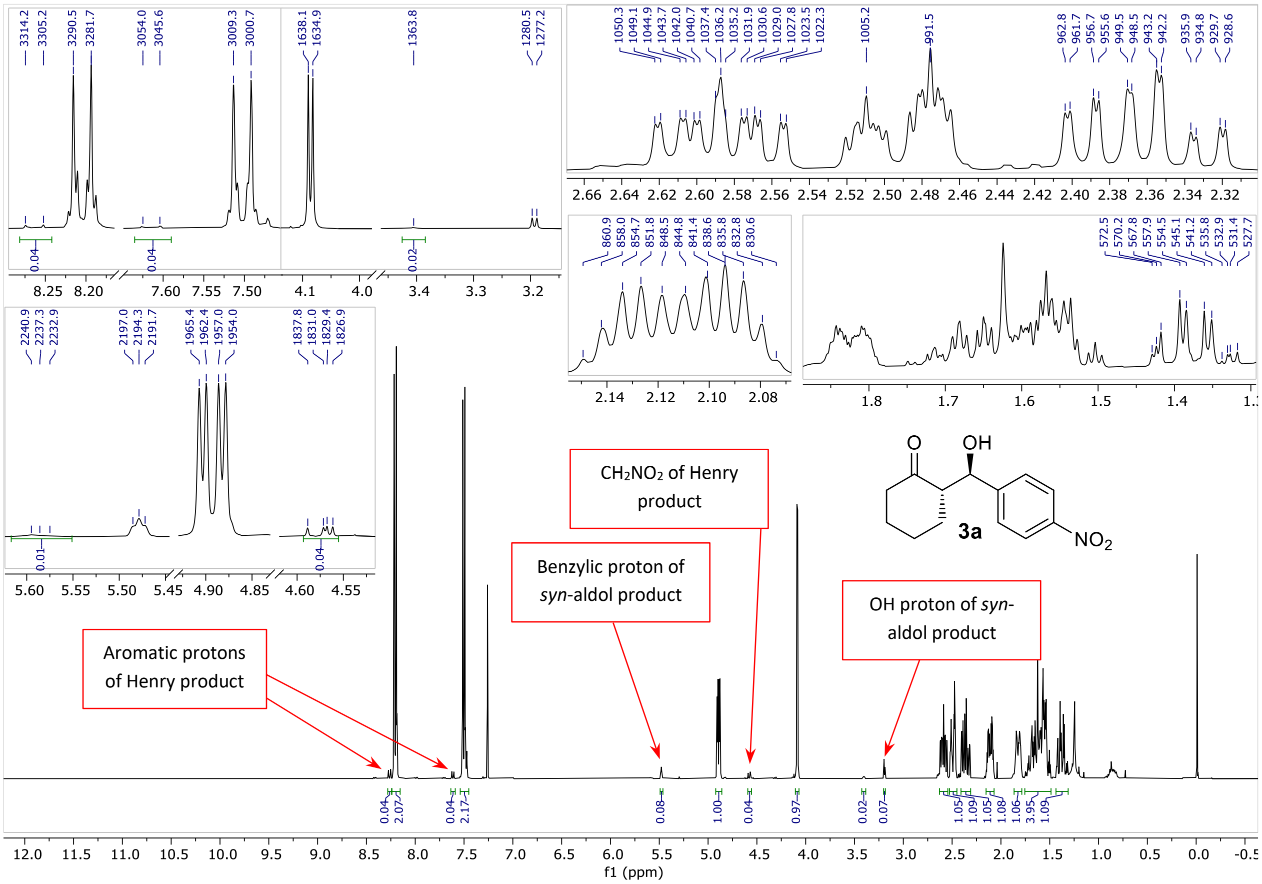

Figure S29. ¹H NMR spectrum of purified *anti*-(major) **3a** and *syn*-(minor) **4a** aldol products. The red arrows indicate the *syn*-aldol product.

Epimerization of aldol product 3a: Aldol product diastereomers of the structural category produced in this study are prone to epimerization on heating or on exposure to silica gel. Consequently, crude aldol product ¹H NMRs can show higher *dr* values than ¹H NMRs of the chromatographed aldol products. This, in large part, is why most researchers in this area decided long ago to evaluate the aldol diastereoselectivity using crude ¹H NMR spectrums. Furthermore, most researchers isolate the *anti-*/*syn*-aldol products together and report the total yield. For this study, we decided to isolate the *anti*-aldol products and record their yield, and that was possible for all products except *anti*-aldol product **3a**. The *anti*-aldol product **3a** epimerizes during chromatography to an extent that prohibits its isolation free of the *syn*-aldol product **4a**. For related matters, see page S4 within the Supporting Information of: Nugent, T.C.; Umar, M. N.; Bibi, A. Picolylamine as an Organocatalyst Template for Highly Diastereo- and Enantioselective Aqueous Aldol Reactions. *Org. Biomol. Chem.* **2010**, *8*, 4085-4089.

SHIMADZU LabSolutions **Analysis Report**

ADV-A45-7% IPA/nHex 5uL 1mL/min 12May2022

Sample Name	: A46 (RACE)7% IPAnHex 5uL1mLmin 26May 2		
Sample ID	:		
Data Filename	: A46 (RACE)7% IPAnHex 5uL1mLmin 26May 2.lcd		
Method Filename	: trial.lcm		
Batch Filename	:		
Vial #	: 1-1	Sample Type	: Unknown
Injection Volume	: 20 uL		
Date Acquired	: 5/26/2022 2:38:31 PM	Acquired by	: System Administrator
Date Processed	: 5/26/2022 3:28:36 PM	Processed by	: System Administrator

ADV-A46-7% IPA/nHex 5uL 1mL/min 12May2022

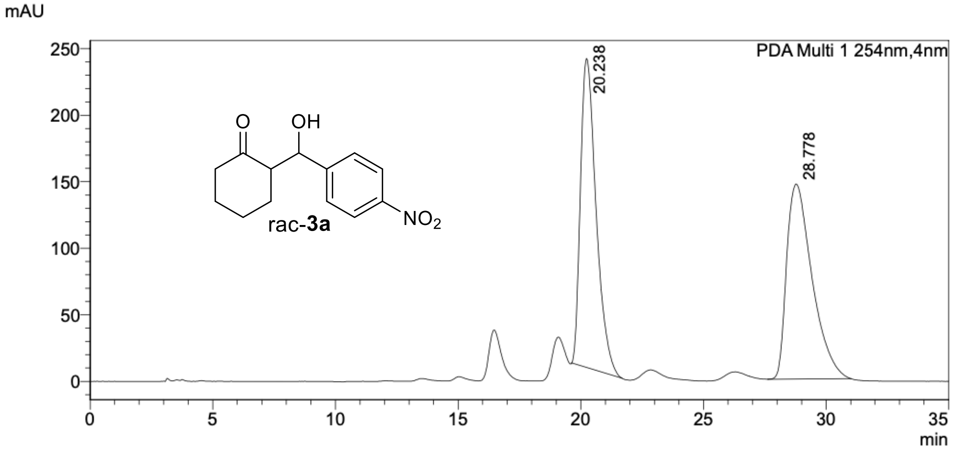

ADV-A45-7% IPA/nHex 5uL 1mL/min 12May2022

PDA Ch1 254nm

Peak#	Ret. Time	Area	Height	Area%
1	20.238	10283216	232276	49.316
2	28.778	10568658	146536	50.684
Total		20851874	378812	100.000

C:\Users\Shimadzu\Desktop\ADV\Competition aldol samples\A46 (RACE)7% IPAnHex 5uL1mLmin 26May 2.lcd

Figure S30. HPLC chromatogram of the racemic *anti*-aldol product **3a** (above).

SHIMADZU LabSolutions

Analysis Report

<Sample Information>

Sample Name	: DK1-B		
Data Filename	: DK1-B.lcd		
Method Filename	: RL1-7%IPANHEX-1000PPM-SAM[10%IPANHEX].lcm		
Vial #	: 1-6	Sample Type	: Unknown
Injection Volume	: 20 uL		
Date Acquired	: 9/20/2024 11:57:24 AM	Acquired by	: System Administrator
Date Processed	: 9/20/2024 12:37:26 PM	Processed by	: System Administrator

<Chromatogram>

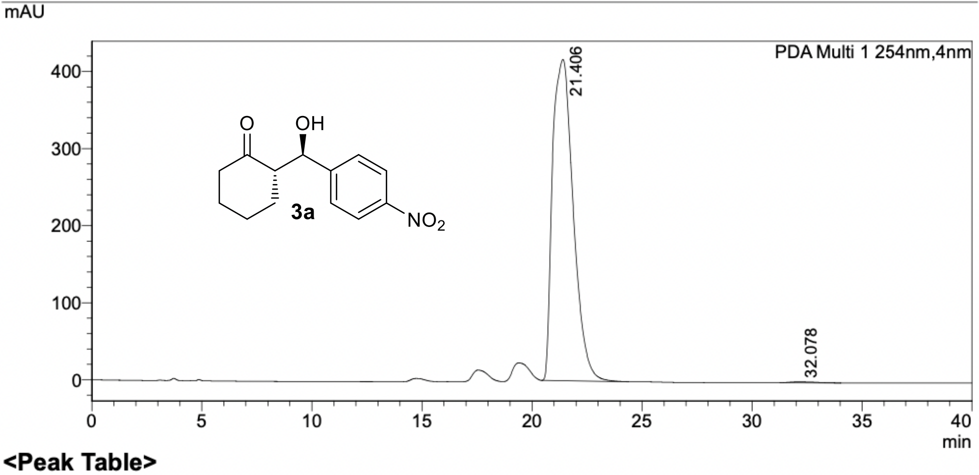

<Peak Table>

PDA Ch1 254nm

Peak#	Ret. Time	Area	Height	Area%
1	21.406	27352617	416920	99.615
2	32.078	105799	1273	0.385
Total		27458416	418193	100.000

C:\LabSolutions\Data\Project1\Data\Diana\DK1-B.lcd

Figure S31. HPLC chromatogram of the enantioenriched *anti*-aldol (major) product **3a** (above).

Table 2, entry 2: Competition reaction between cyclohexanone and nitromethane for the limiting reactant 2-nitrobenzaldehyde

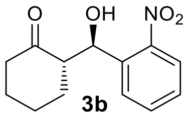

(*S*)-2-((*R*)-hydroxy(2-nitrophenyl)methyl)cyclohexan-1-one (**3b**):
To a clean, screw cap, V-shaped reaction vessel (5.0 mL) equipped with a small pyramidal stir bar, mortar and pestle ground 2-nitrobenzaldehyde (MW = 151.12 g/mol, 1.00 equiv, 1.0 mmol, 151.12 mg), cyclohexanone (MW = 98.15 g/mol, 1.50 equiv, 1.5 mmol, 147.21 mg, 155 µL, density 0.948 g/mL), and nitromethane (MW = 61.04 g/mol, 3.00 equiv, 3.0 mmol, 183.12 mg, 162 µL, density 1.132 g/mL) were added. The liquid reactants (cyclohexanone and nitromethane) were used to rinse the solid 2-nitrobenzaldehyde off the walls as needed. The heterogeneous mixture was allowed to gently stir for < 1 min, visual inspection showed full dissolution of the 2-nitrobenzaldehyde. Next, the stirring was terminated and *trans*-4-(tert-butyldiphenylsilyloxy)-*L*-proline (MW = 369.54 g/mol, 2.5 mol%, 0.025 mmol, 9.22 mg) was added. Distilled deoxygenated water (MW = 18.02 g/mol, 15.00 equiv, 15.0 mmol, 270.30 mg, 270 µL) was added within 30 sec. The resulting heterogeneous solution was gently stirred such that the contents of the vessel did not splash against the vessel walls but allowed the phase boundary to be gently agitated. Both the aqueous and organic layers were transparent. The reaction was quenched at 36 h. See Section 2 for the work-up procedure.

This compound was previously synthesized and characterized, see below.

Crude product ^{1}H NMR analysis (Figure S32, see below) allowed determination of the diastereo- and chemoselectivity ratios.

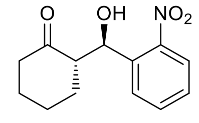

anti-aldol **3b** (major)

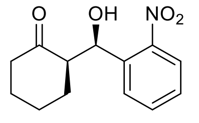

syn-aldol **4b** (minor)

Diastereoselectivity: The *anti-/syn*-aldol ratio was determined as >19:1 from the benzylic proton resonances at 5.44 ppm (d, *anti*-aldol product) and 5.96 ppm (d, *syn*-aldol product) in the crude ^{1}H NMR spectrum (Figure S32). For the literature chemical shift values, see page S2 (product **3i**) of the Supporting Information within: Mase, N.; Nakai, Y.; Ohara, N.; Yoda, H.; Takabe, K.; Tanaka, F.; Barbas, C. F., III. Organocatalytic Direct Asymmetric Aldol Reactions in Water. *J. Am. Chem. Soc.* **2006**, *128*, 734–735.

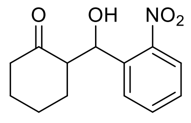

aldol products **3b**/**4b**

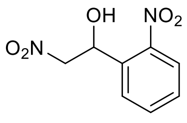

Henry products

Chemoselectivity: The *anti*- and *syn*-aldol/Henry product ratio was determined to be >19:1, based on a lack of resonance patterns for the Henry product at 4.87 ppm (dd, benzylic proton) or at 4.55 ppm (dd, CH_2NO_2), see Figure S32. For related Henry product chemical shift values see: Pandey, S.; Bansal, D.; Gupta, R. A Metalloligand Appended with Benzimidazole Rings: Tetranuclear [$CoZn_3$] and [$CoCd_3$] Complexes and Their Catalytic Applications. *New J. Chem.* **2018**, *42*, 9847–9856. An alternative reference is: Khromova, O. V.; Yashkina, L. V.; Stoletova, N. V.; Maleev, V. I.; Belokon, Y. N.; Larionov, V. A. Selectivity Control in Nitroaldol (Henry) Reaction by Changing the Basic Anion in a Chiral Copper(II) Complex Based on (S)-2-Aminomethylpyrrolidine and 3,5-Di-tert-butylsalicylaldehyde. *Molecules* **2024**, *29*, 5207. https://doi.org/10.3390/molecules29215207

Purification and yield: Silica gel chromatography (25 mm column outer diameter, 16 cm silica bed height) was performed using gradient elution (6 to 11 vol% EtOAc in petroleum ether). The crude product was loaded onto the column in a minimum volume of CH_2Cl_2. The product fractions were collected when using 11 vol% EtOAc in petroleum ether. After concentration (rotary evaporation) and high vacuum drying, a yellow solid weighing 201.3 mg (MW = 249.27 g/mol, 0.808 mmol, 81% yield) of the *anti*-aldol diastereomer was isolated.

TLC: *anti*-aldol product R_f = 0.31 EtOAc/petroleum ether (3:7).

99% *ee*: Chiralcel OD-H chiral HPLC column, *i*PrOH/n-hexane (10:90), flow rate = 1.0 mL/min, λ = 254 nm, injection volume = 20 μL, the sample was dissolved in 10 vol% *i*PrOH/n-hexane with a concentration of ≈ 1 mg/mL; *anti*-aldol product t_{major} = 9.6 min, t_{minor} = 11.7 min retention times were observed. (Figures S34 & S35).

^{1}H NMR (400 MHz, $CDCl_3$) (ppm) *anti*-aldol product **3b** (Figure S33): δ 7.85 (dd, 1H, J = 8.2, 1.3 Hz), 7.77 (dd, 1H, J = 7.9, 1.3 Hz), 7.65 (td, 1H, J = 7.4, 1.3 Hz), 7.46-7.41 (m, 1H), 5.47-5.43 (d, 1H, J = 6.6 Hz), 4.18 (br s, 1H), 2.79-2.72 (m, 1H), 2.48-2.42 (m, 1H), 2.38-2.28 (m, 1H), 2.14-2.05 (m, 1H), 1.86-1.51 (m, 5H).

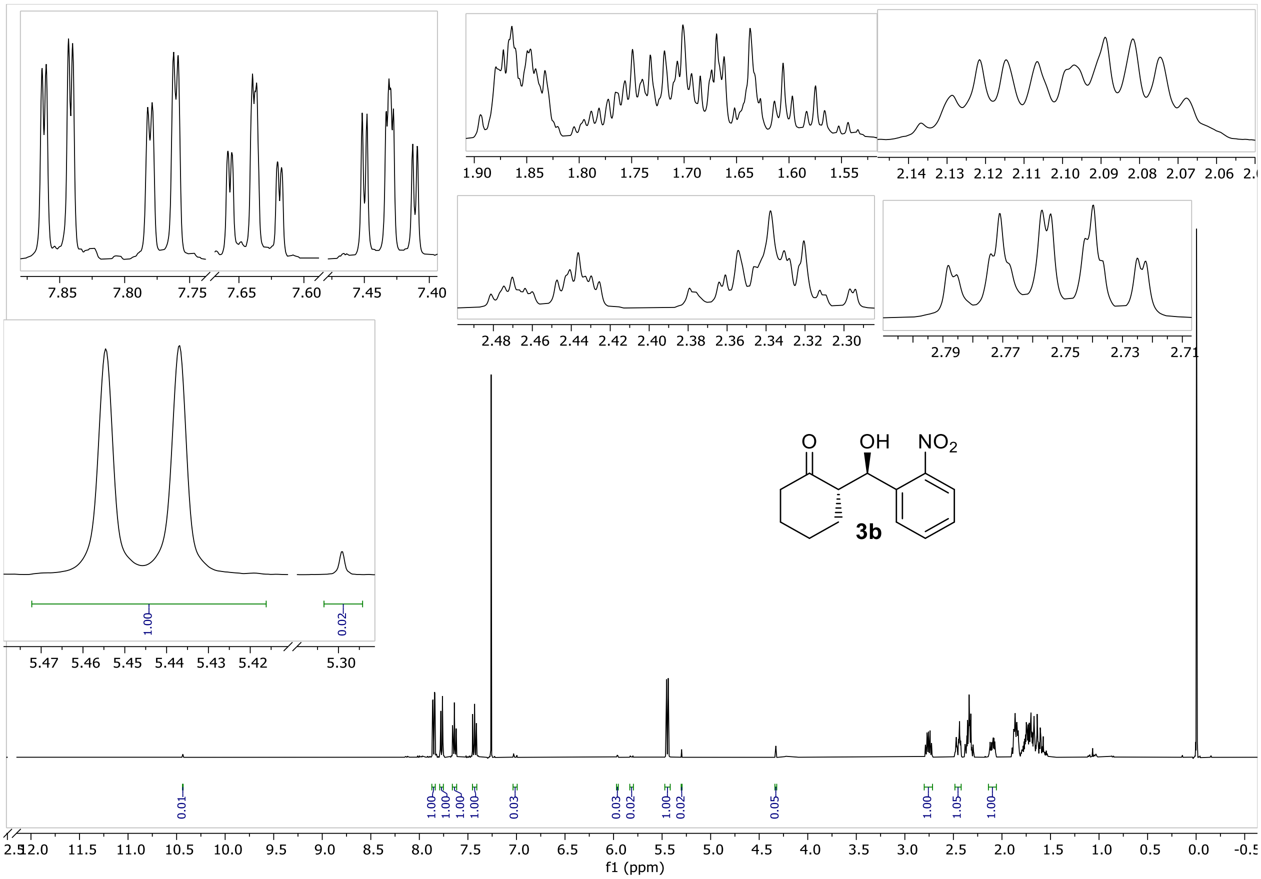

Figure S32. Crude ^{1}H NMR spectrum after high vacuum drying **3b** (above).

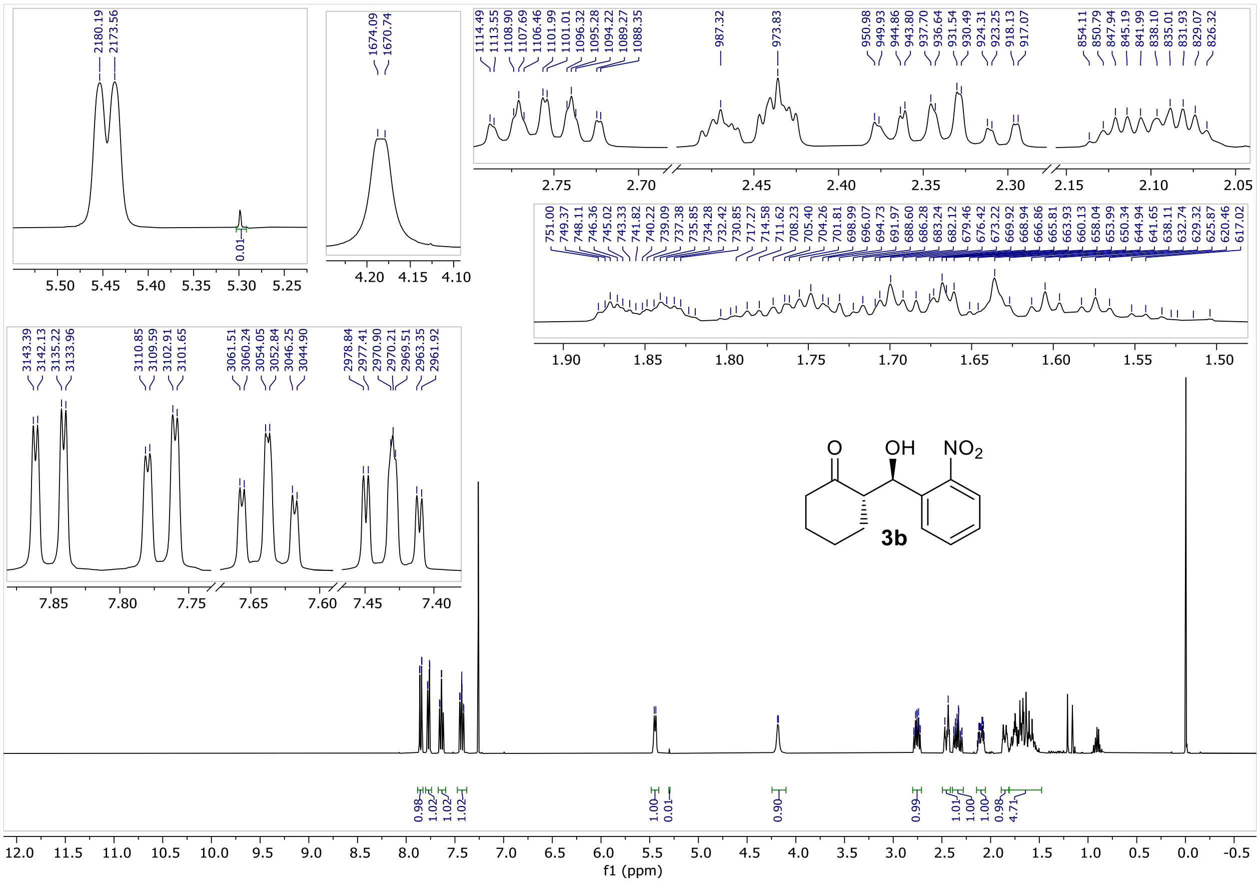

Figure S33. ^{1}H NMR spectrum of the purified *anti*-aldol (major) product **3b** (above).

Data File D:\DATA\NUGENT\AL41 RACE 10%IPAINHEX 04MAR25.D
Sample Name: AL41 RACE 10%IPAinHEX 04MAR25

===

Acq. Operator : Seq. Line : 3
Acq. Instrument : Instrument 1 Location : P1-A-03
Injection Date : 3/4/2025 12:39:18 PM Inj : 1
Inj Volume : 20 µl
Acq. Method : D:\Methods\Nugentlab_Patrick\AIDA 10IPA 254NM.m
Last changed : 3/4/2025 12:51:00 PM
Analysis Method : D:\Methods\Nugentlab_Patrick\AIDA 15IPA 254NM.m
Last changed : 3/3/2025 4:17:17 PM
(modified after loading)

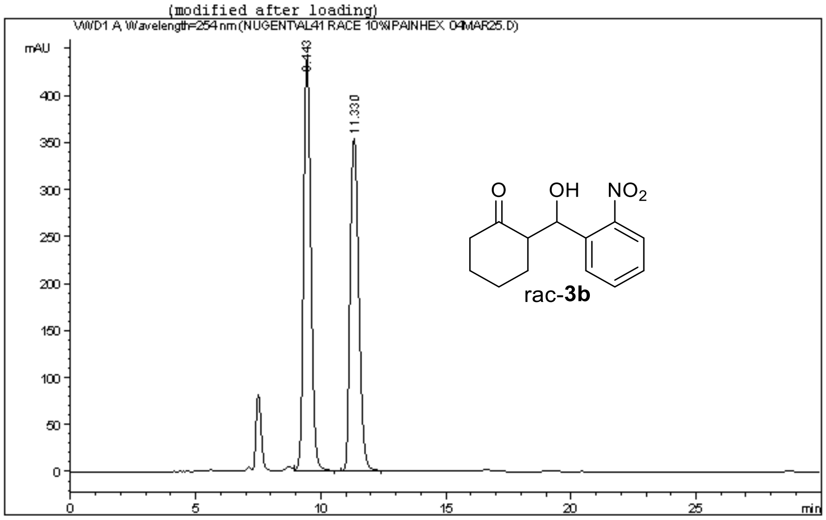

===

Area Percent Report

===

Sorted By : Signal
Multiplier : 1.0000
Dilution : 1.0000
Use Multiplier & Dilution Factor with ISTDs

Signal 1: VWD1 A, Wavelength=254 nm

Peak #	RetTime [min]	Type	Width [min]	Area mAU *s	Height [mAU]	Area %
1	9.443	VB	0.3240	8978.00586	437.18134	51.3284
2	11.330	BB	0.3840	8513.29590	353.96155	48.6716

Totals : 1.74913e4 791.14288

===

*** End of Report ***

Instrument 1 3/4/2025 1:12:40 PM Page 1 of 1

Figure S34. HPLC chromatogram of the racemic *anti*-aldol product **3b** (above).

Data File D:\DATA\NUGENT\DK2E ENANTIOEN 10%IPAINHEX 16APR25.D
Sample Name: DK2E ENANTIOEN 10%IPAINHEX 16APR25

===

Acq. Operator : Seq. Line : 2
Acq. Instrument : Instrument 1 Location : P1-A-02
Injection Date : 4/16/2025 1:16:23 PM Inj : 1
Inj Volume : 20 µl
Acq. Method : D:\Methods\Nugentlab_Patrick\AIDA 10IPA 254NM.m
Last changed : 4/16/2025 1:15:25 PM
Analysis Method : D:\Methods\Nugentlab_Patrick\AIDA 10IPA 254NM.m
Last changed : 4/16/2025 1:50:45 PM
(modified after loading)

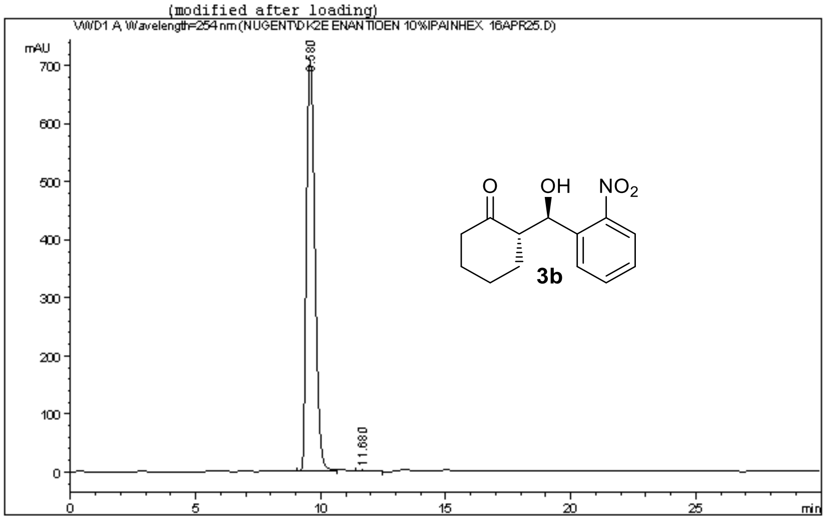

===

Area Percent Report

===

Sorted By : Signal
Multiplier : 1.0000
Dilution : 1.0000
Use Multiplier & Dilution Factor with ISTDs

Signal 1: VWD1 A, Wavelength=254 nm

Peak #	RetTime [min]	Type	Width [min]	Area mAU *s	Height [mAU]	Area %
1	9.580	BB	0.3760	1.67529e4	709.30896	99.5477
2	11.680	VB	0.4583	76.11137	2.41253	0.4523

Totals : 1.68290e4 711.72149

===

*** End of Report ***

Instrument 1 4/16/2025 1:50:55 PM Page 1 of 1

Figure S35. HPLC chromatogram of the enantioenriched *anti*-aldol (major) product **3b** (above).

Table 2, entry 3: Competition reaction between cyclohexanone and nitromethane for the limiting reagent methyl 4-formylbenzoate

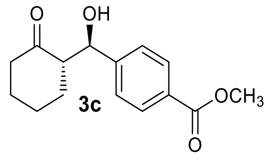

3c

Methyl 4-((*R*)-hydroxy((*S*)-2-oxocyclohexyl)methyl)benzoate (**3c**):
To a clean, screw cap, V-shaped reaction vessel (5.0 mL) equipped with a small pyramidal stir bar mortar and pestle ground methyl 4-formylbenzoate (MW = 164.16 g/mol, 1.0 equiv, 1.0 mmol, 164.16 mg), cyclohexanone (MW = 98.15 g/mol, 1.50 equiv, 1.5 mmol, 147.2 mg, 155 µL, density 0.947 g/mL), and nitromethane (MW = 61.04 g/mol, 3.0 equiv, 3.0 mmol, 162 µL, density 1.132 g/mL) were added. The liquid reactants (cyclohexanone and nitromethane) were used to rinse the solid methyl 4-formylbenzoate off the walls as needed. Stirring was initiated, and within 30 sec a transparent homogeneous mixture resulted. Next, the stirring was terminated, and *trans*-4-(tert-butyldiphenylsilyloxy)-L-proline (MW = 369.54 g/mol, 2.5 mol%, 0.025 mmol, 9.2 mg) was added. Distilled deoxygenated water (MW = 18.02 g/mol, 15.0 equiv., 15.0 mmol, 270.3 mg, 270 µL) was added within 30 sec (the concentrated organic phase immediately turned and remained milky for ≈ 30 min). The resulting biphasic solution was intentionally stirred such that the contents of the vessel did not splash against the vessel wall, but such that the phase boundary was gently agitated. The concentrated organic phase became transparent after 30 min and the reaction was stirred for 40 h. See Section 2 for the work-up procedure.

This compound was previously synthesized and characterized, see below.

Crude product ^{1}H NMR analysis (Figure S36, see below) allowed determination of the diastereo- and chemoselectivity ratios.

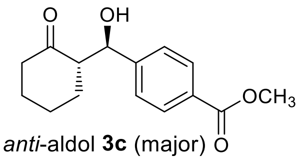

anti-aldol **3c** (major)

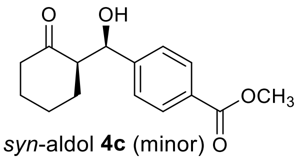

syn-aldol **4c** (minor)

Diastereoselectivity: The *anti-/syn*-aldol ratio was determined as 14.3:1 from the benzylic proton resonances at 4.84 ppm (d, *anti*-aldol product) and 5.44 ppm (d, *syn*-aldol product) in the crude ^{1}H NMR spectrum (Figure S36). For the literature provided chemical shift values, see page S2 (product 3c) of the Supporting Information within: Mase, N.; Nakai, Y.; Ohara, N.; Yoda, H.; Takabe, K.; Tanaka, F.; Barbas, C. F., III. Organocatalytic Direct Asymmetric Aldol Reactions in Water. *J. Am. Chem. Soc.* **2006**, *128*, 734–735.

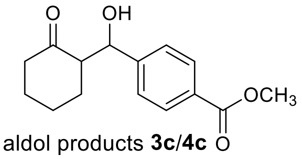

aldol products **3c**/**4c**

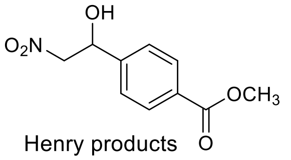

Henry products

Chemoselectivity: The *anti*- and *syn*-aldol/Henry product ratio was determined to be >19:1, based on the lack of a Henry product resonance at 4.59 ppm (dd, benzylic proton), see Figure S36. For literature regarding the Henry product chemical shifts, see page 239 (compound 3c) within the Experimental Section of: Zhang, S.; Xiangrui, L.; Wenxin, L.; Weidong, R.; Danhua, G.; Zhiliang, S.; Xueqiang, C. Iron(0)-Mediated Henry-Type Reaction of Bromonitromethane with Aldehydes for the Efficient Synthesis of 2-Nitro-alkan-1-ols. *Chin. J. Org. Chem.* **2022**, *42*, 235-241.

Purification and yield: Silica gel chromatography (25 mm column outer diameter, 18 cm silica bed height) was performed using gradient elution (10 to 16 vol% EtOAc in petroleum ether). The crude product was loaded onto the column in a minimum volume of CH_2Cl_2. The *anti*-aldol product, a white solid, was isolated as a single diastereomer (232.2 mg, MW = 262.31 g/mol, 0.885 mmol, 89% yield).

TLC: *anti*-aldol product R_f = 0.23, *syn*-aldol product R_f = 0.31 (EtOAc/petroleum ether, 1:4).

98% *ee*: Chiralcel OD-H chiral HPLC column, iPrOH/n-hexane (5:95), flow rate = 1.0 mL/min, λ = 254 nm, injection volume = 20 μL, the sample was dissolved in 5 vol% *i*PrOH/n-hexane with a concentration of ≈1 mg/mL; *anti*-aldol product retention times: t_{minor}= 18.9 min, t_{major}= 21.2 min (Figures S38 & S39).

^{1}H NMR (400 MHz, $CDCl_3$) (ppm) *anti*-aldol product **3c** (Figure S37): δ 8.02 (d, 2H, J = 8.3 Hz), 7.39 (d, 2H, J = 8.3 Hz), 4.84 (dd, 1H, J = 8.7 Hz, 3.0 Hz), 4.02 (d, 1H, J = 2.9 Hz), 3.91 (s, 3H), 2.63-2.55 (m, 1H), 2.51-2.44 (m, 1H), 2.41-2.30 (m, 1H), 2.13-2.05 (m, 1H), 1.83-1.75 (m, 1H), 1.72-1.47 (m, 3H), 1.39-1.21 (m, 2H).

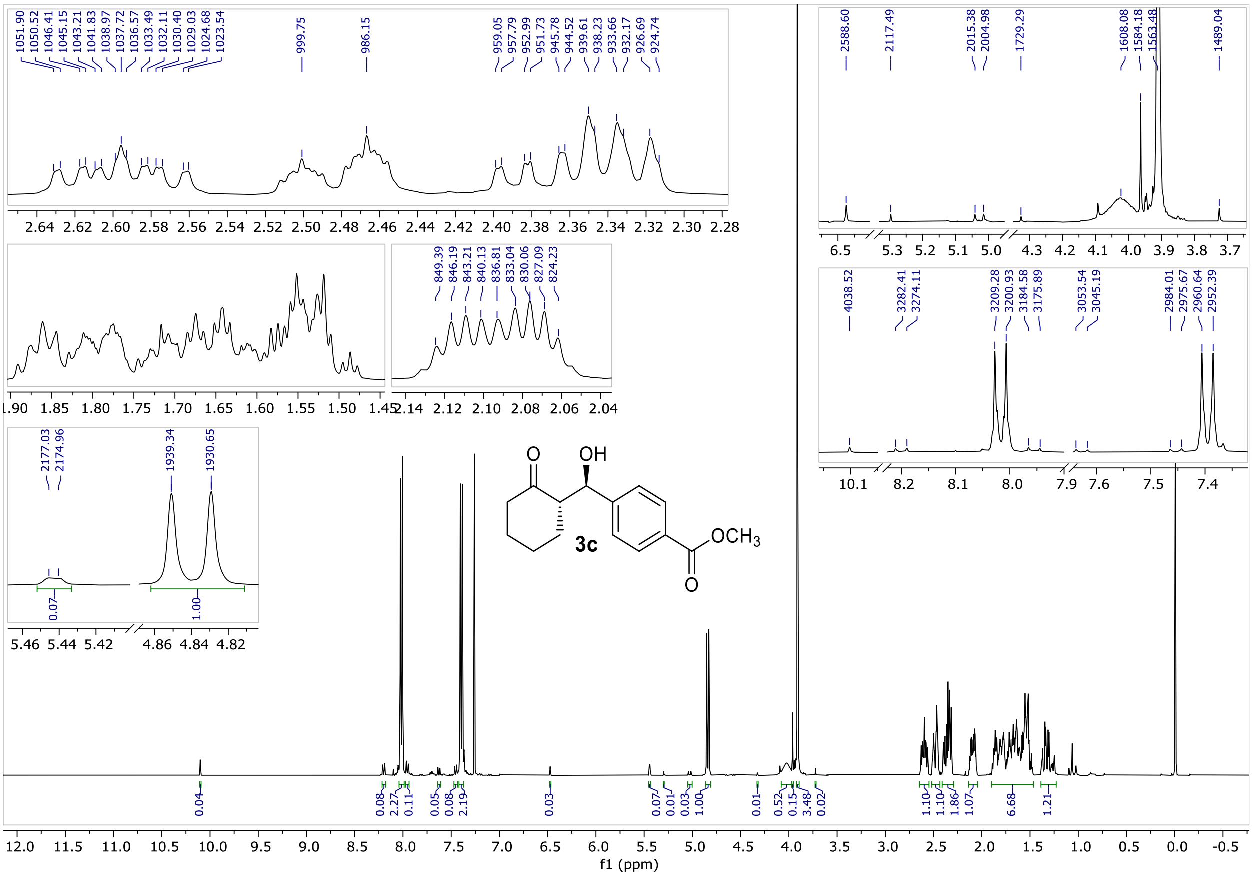

Figure S36. Crude ^{1}H NMR spectrum after high vacuum drying **3c** (above).

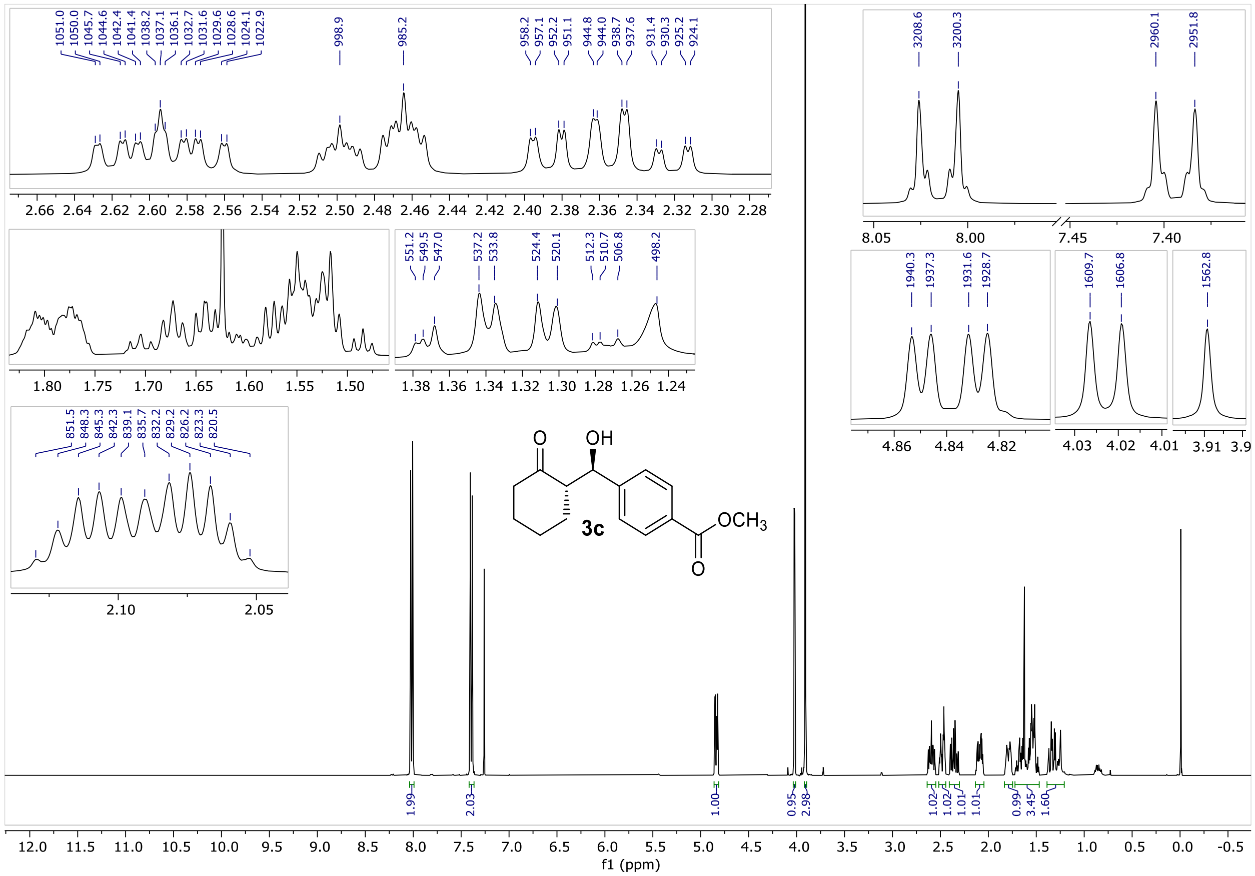

Figure S37. ^{1}H NMR spectrum of the purified *anti*-aldol (major) product **3c** (above).

SHIMADZU LabSolutions **Analysis Report**

<Sample Information>

Sample Name	: SSZ02 RACE 2mgmL 5%IPAnHex 20uL 1mLmin 17nov22		
Sample ID	:		
Data Filename	: SSZ02 RACE 2mgmL 5%IPAnHex 20uL 1mLmin 17nov22.lcd		
Method Filename	: trial.lcm		
Batch Filename	:		
Vial #	: 1-4	Sample Type	: Unknown
Injection Volume	: 20 uL		
Date Acquired	: 11/17/2022 12:56:08 PM	Acquired by	: System Administrator
Date Processed	: 11/17/2022 1:34:19 PM	Processed by	: System Administrator

<Chromatogram>

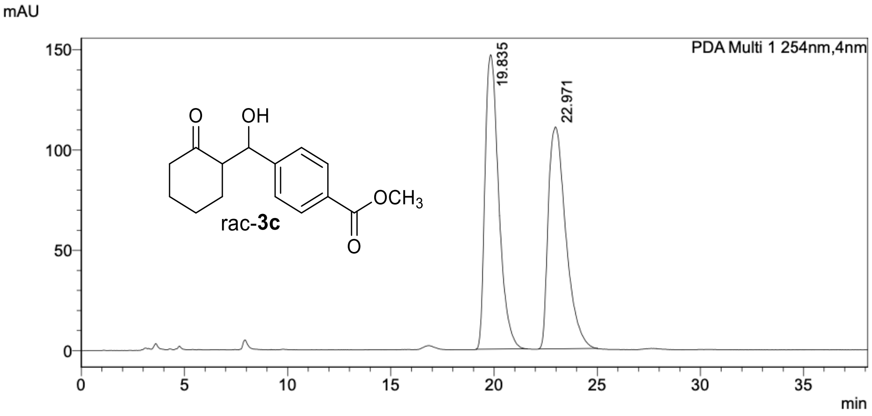

<Peak Table>

PDA Ch1 254nm

Peak#	Ret. Time	Area	Height	Area%
1	19.835	6780885	146718	50.893
2	22.971	6542886	110549	49.107
Total		13323772	257267	100.000

C:\Users\Shimadzu\Desktop\SZ\HPLC\Runs\SSZ02 RACE 2mgmL 5%IPAnHex 20uL 1mLmin 17nov22.lcd

Figure S38. HPLC chromatogram of the racemic *anti*-aldol (major) product **3c** (above).

SHIMADZU LabSolutions **Analysis Report**

<Sample Information>

Sample Name	: RL5-IV-5%IPANHEX-1000PPM-SAM[5%IPANHEX]		
Data Filename	: RL5-IV-5%IPANHEX-1000PPM-SAM[5%IPANHEX].lcd		
Method Filename	: RL5-5%IPANHEX-1000PPM-SAM[5%IPANHEX].lcm		
Vial #	: 1-5	Sample Type	: Unknown
Injection Volume	: 20 uL		
Date Acquired	: 8/30/2024 4:01:38 PM	Acquired by	: System Administrator
Date Processed	: 8/30/2024 4:31:40 PM	Processed by	: System Administrator

<Chromatogram>

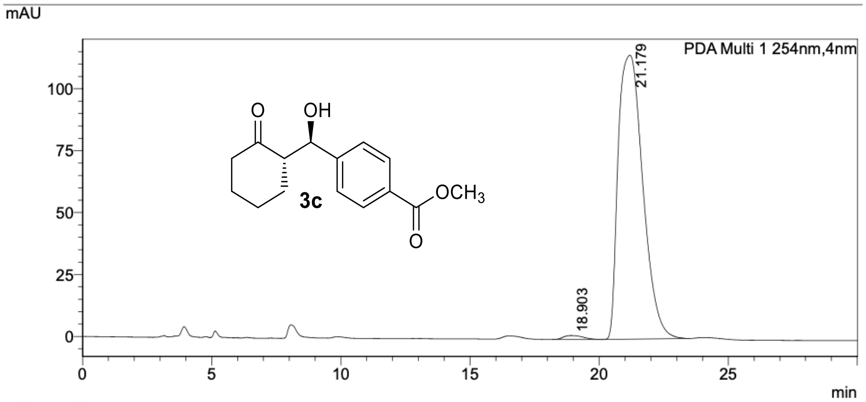

<Peak Table>

PDA Ch1 254nm

Peak#	Ret. Time	Area	Height	Area%
1	18.903	86879	1616	1.128
2	21.179	7616723	114692	98.872
Total		7703602	116307	100.000

C:\LabSolutions\Data\Project1\Data\RUSLAN\RL5-IV-5%IPANHEX-1000PPM-SAM[5%IPANHEX].lcd

Figure S39. HPLC chromatogram of the enantioenriched *anti*-aldol (major) product **3c** (above).

Table 2, entry 4: Competition reaction between cyclohexanone and nitromethane for the limiting reagent 4-formylbenzonitrile

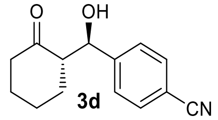

4-((*R*)-hydroxy((*S*)-2-oxocyclohexyl)methyl)benzonitrile (**3d**):
To a clean, screw cap, V-shaped reaction vessel (5.0 mL) equipped with a small pyramidal stir bar mortar and pestle ground 4-formylbenzonitrile (MW = 131.13 g/mol, 1.0 equiv, 1.0 mmol, 131.13 mg), cyclohexanone (MW = 98.15 g/mol, 1.5 equiv, 1.5 mmol, 146.79 mg, 155 µL, density = 0.947 g/mL) and nitromethane (MW = 61.04 g/mol, 2.97 equiv, 2.97 mmol, 181.12 mg, 160 µL, density = 1.132 g/mL) were added. The liquid reactants (cyclohexanone and 1-nitropropane) were used to rinse the solid 4-formylbenzonitrile off the walls as needed. Stirring was initiated, and within a minute a fully dissolved, transparent and colorless mixture resulted. Next, the stirring was terminated, and *trans*-4-(tert-butyldiphenylsilyloxy)-*L*-proline (MW = 369.54 g/mol, 2.5 mol%, 0.025 mmol, 9.2 mg) was added. Within 30 sec, distilled deoxygenated water (MW = 18.02 g/mol, 15.0 equiv., 15.0 mmol, 270 mg, 270 µL) was carefully added to ensure minimal disruption of the concentrated organic layer. Within minutes, the concentrated organic phase appeared transparent and had a very light-yellow color. Appearance stayed the same at time of quenching, 40 h. Note: Stirring was maintained such that the contents of the vessel did not splash against the vessel wall, but the phase boundary was gently agitated. The reaction time has not been optimized. See Section 2 for the work-up procedure.

This compound was previously synthesized and characterized, see below.

Crude product ^{1}H NMR analysis (Figure S40, see below) allowed determination of the diastereo- and chemoselectivity ratios.

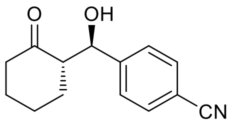

anti-aldol **3d** (major)

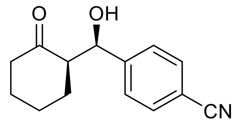

syn-aldol **4d** (minor)

Diastereoselectivity: The *anti-/syn*-aldol ratio was determined as >19:1 based on the benzylic proton resonances at 4.84 ppm (d, *anti*-aldol product) and 5.43 ppm (d, *syn*-aldol product) in the crude ^{1}H NMR spectrum (Figure S40). For the chemical shift literature values, see page 9 (product 18g) of the Supporting Information within: Agarwal, J.; Peddinti, R. Glucosamine-based primary amines as organocatalysts for the asymmetric aldol reaction. *J. Org. Chem*. **2011**, *76*, 9, 3502–3505.

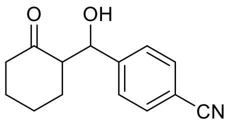

aldol products **3d**/**4d**

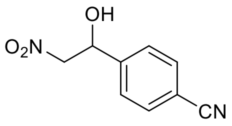

Henry products

Chemoselectivity: The *anti*- and *syn*-aldol/Henry product ratio is >19:1, based on the integration sums for the *anti*-aldol product resonance at resonance at 4.84 ppm (d, representing the benzylic proton) and the *syn*-aldol product resonance at 5.43 ppm (d, representing the benzylic proton) *versus* the Henry product resonances at 5.53 ppm (dd, benzylic proton) and 4.55 ppm (m, C$\underline{H}_2$NO$_2$) in the crude ^{1}H NMR spectrum (Figure S40). For the chemical shift literature values, see page 6 (product 3i) of the Supporting Information within: Gao, N.; Chen, Y. -L.; He, Y. -H.; Guan, Z. Highly efficient and large-scalable glucoamylase-catalyzed Henry reactions. *RSC Adv.* **2013**, *3*, 16850–16856.

Purification and yield: Silica gel chromatography (25 mm column outer diameter, 20 cm silica bed height) was performed using isocratic elution (20 vol% EtOAc in petroleum ether). The crude product was loaded onto the column with minimum volume CH_2Cl_2. The *anti*-aldol product, a white solid, was isolated as a single diastereomer (202.8 mg, MW = 229.28 g/mol, 0.885 mmol, 88.5 % yield).

TLC: *anti*-aldol product R_f = 0.22, *syn*-aldol product R_f = 0.31 (EtOAc/petroleum ether, 3:10).

99% *ee*: Chiralcel OD-H chiral HPLC column, iPrOH/n-hexane (30:70), flow rate = 0.5 mL/min, λ = 216 nm, injection volume = 20 μL, the sample was dissolved in 30 vol% *i*PrOH/n-hexane with a concentration of ≈1 mg/mL; *anti*-aldol product retention times: t_{minor}= 22.0, t_{major}= 15.7 (Figures S42 and S43).

^{1}H NMR (400 MHz, $CDCl_3$) (ppm) *anti*-aldol product **3d** (Figure S41): δ 7.65 (d, 2H, J = 8.4 Hz), 7.45 (d, 2H, J = 8.1 Hz), 4.84 (dd, 1H, J = 8.5, 3.0 Hz), 4.06 (d, 1H, J = 3.1 Hz), 2.61-2.46 (m, 2H), 2.41-2.31 (m, 1H), 2.16-2.07 (m, 1H), 1.88-1.78 (m, 1H), 1.74-1.48 (m, 3H), 1.42-1.29 (m, 1H).

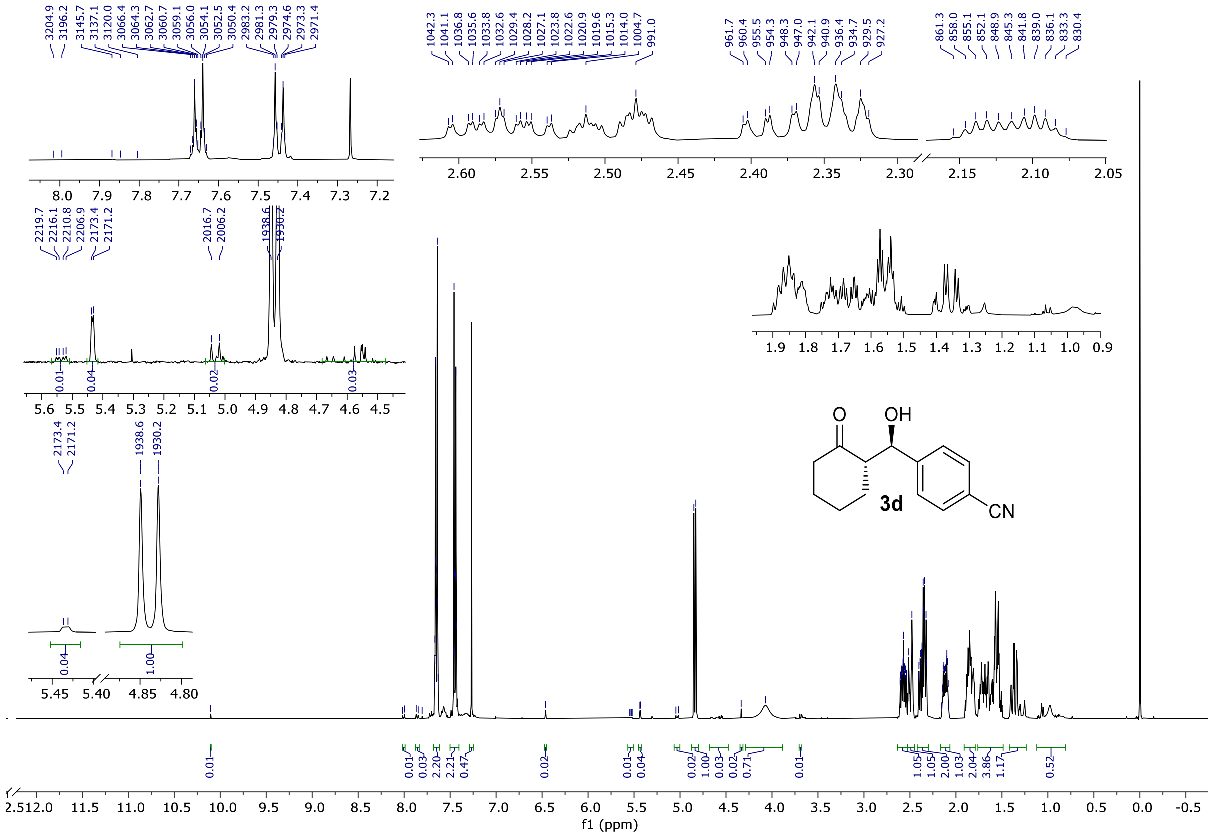

Figure S40. Crude ^{1}H NMR spectrum after high vacuum drying **3d** (above).

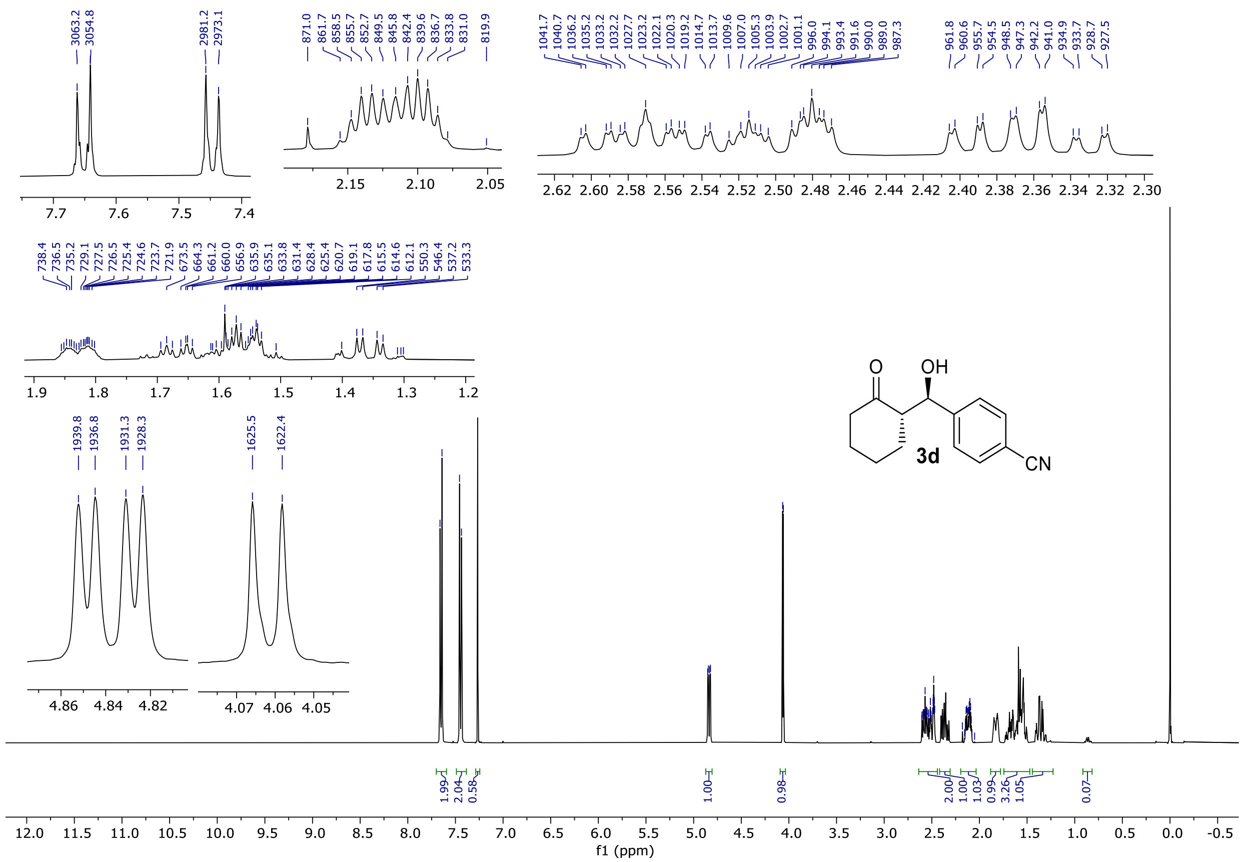

Figure S41. ^{1}H NMR spectrum of purified *anti*-aldol (major) product **3d** (above).

```
Data File D:\DATA\NUGENT\AL 34 RAC 30% IPAINHEX 14FEB25.D
Sample Name: AL 34 RAC 30% IPAinHEX 14FEB25
=====================================================================
    Acq. Operator   :                                  Seq. Line :   7
    Acq. Instrument : Instrument 1                      Location : P1-A-07
    Injection Date  : 2/14/2025 3:37:25 PM                   Inj :   1
                                                      Inj Volume : 20 µl
    Acq. Method     : D:\Methods\Nugentlab_Patrick\AIDA.m
    Last changed    : 2/14/2025 3:36:05 PM
    Analysis Method : D:\Methods\Nugentlab_Patrick\AIDA.m
    Last changed    : 2/5/2025 12:02:12 PM
=====================================================================
```

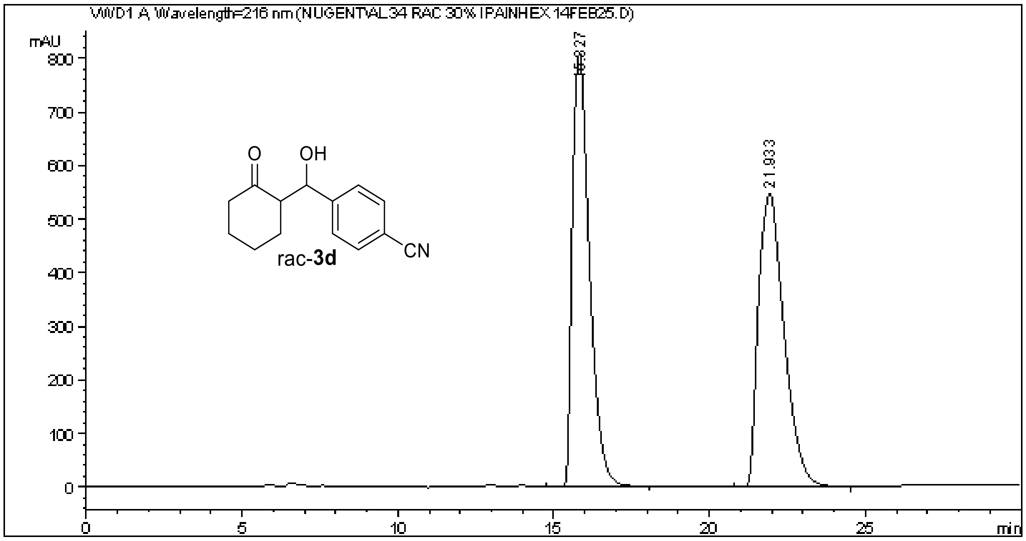


```
=====================================================================
                          Area Percent Report
=====================================================================

Sorted By             :      Signal
Multiplier            :      1.0000
Dilution              :      1.0000
Use Multiplier & Dilution Factor with ISTDs


Signal 1: VWD1 A, Wavelength=216 nm

Peak RetTime Type  Width     Area      Height     Area
  #   [min]        [min]  mAU   *s   [mAU   ]      %
----|-------|----|-------|----------|----------|--------|
   1  15.827 VB    0.5969 3.12011e4  811.76617  49.9797
   2  21.933 BB    0.8812 3.12265e4  548.48279  50.0203

Totals :                  6.24276e4  1360.24896


=====================================================================
                          *** End of Report ***
```


Figure S42. HPLC chromatogram of the racemic *anti*-aldol product **3d** (above).

Data File D:\DATA\NUGENT\AL 28 ENANTIOENR 30% IPAINHEX 14FEB25.D
Sample Name: AL 28 ENANTIOENR 30% IPAinHEX 14FEB25

===

Acq. Operator	:	Seq. Line :	6
Acq. Instrument	: Instrument 1	Location :	P1-A-06
Injection Date	: 2/14/2025 3:06:14 PM	Inj :	1
		Inj Volume :	20 µl
Acq. Method	: D:\Methods\Nugentlab_Patrick\AIDA.m		
Last changed	: 2/14/2025 3:05:11 PM		
Analysis Method	: D:\Methods\Nugentlab_Patrick\AIDA.m		
Last changed	: 2/5/2025 12:02:12 PM		

===

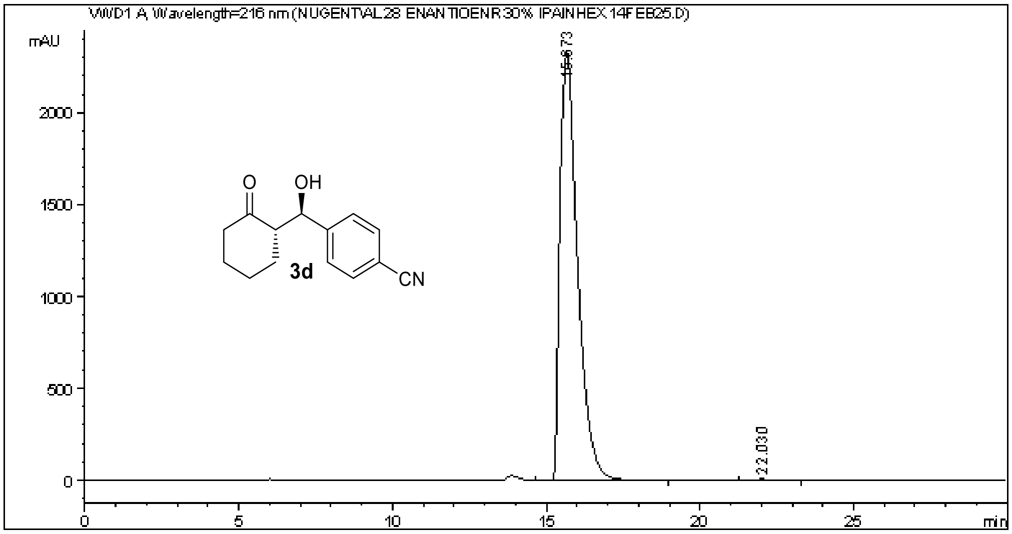

===
Area Percent Report
===

Sorted By : Signal
Multiplier : 1.0000
Dilution : 1.0000
Use Multiplier & Dilution Factor with ISTDs

Signal 1: VWD1 A, Wavelength=216 nm

Peak #	RetTime [min]	Type	Width [min]	Area mAU *s	Height [mAU]	Area %
1	15.673	VB	0.6120	9.33536e4	2334.93652	99.6303
2	22.030	BB	0.7078	346.38644	7.70644	0.3697
Totals :				9.37000e4	2342.64297	

===
*** End of Report ***

Instrument 1 2/14/2025 4:26:01 PM

Page 1 of 1

Figure S43. HPLC chromatogram of the enantioenriched *anti*-aldol (major) product **3d** (above).

Table 2, entry 5: Competition reaction between cyclohexanone and 1-nitropropane for the limiting reactant 4-nitrobenzaldehyde

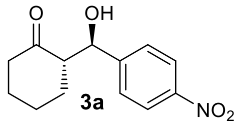

(*S*)-2-((*R*)-hydroxy(4-nitrophenyl)methyl)cyclohexan-1-one (**3a**):
To a clean, screw cap, V-shaped reaction vessel (5.0 mL) equipped with a small pyramidal stir bar, mortar and pestle ground 4-nitrobenzaldehyde (MW = 151.12 g/mol, 1.00 equiv, 1.00 mmol, 151.1 mg), cyclohexanone (MW = 98.14 g/mol, 1.50 equiv, 1.50 mmol, 147.2 mg, 155 µL, density 0.947 g/mL), and 1-nitropropane (MW = 89.09 g/mol, 3.00 equiv, 3.00 mmol, 267.3 mg, 268 µL, density 0.998 g/mL) were added. The liquid reactants (cyclohexanone and 1-nitropropane) were used to rinse the solid 4-nitrobenzaldehyde off the walls as needed. The heterogeneous mixture was allowed to gently stir for < 5 min, to encourage dissolution of the 4-nitrobenzaldehyde, but visual inspection showed little or no dissolution. Next, the stirring was terminated and *trans*-4-(tert-butyldiphenylsilyloxy)-L-proline (MW = 369.54 g/mol, 2.5 mol%, 0.0250 mmol, 9.2 mg) was added. Distilled deoxygenated water (MW = 18.02 g/mol, 15.0 equiv, 15.0 mmol, 270.3 mg, 270 µL) was added within 30 sec. The resulting heterogeneous solution was stirred such that the contents of the vessel did not splash against the vessel walls but allowed gentle phase boundary agitation. Note that a clear phase boundary was always evident. Within approximately 2 h, the undissolved solid material fully dissolved in the concentrated organic phase. Work-up was performed at 24 h. See Section 2 for the work-up procedure.

This compound was previously synthesized and characterized, see below.

Crude product ^{1}H NMR analysis (Figure S44, see below) allowed determination of the diastereo- and chemoselectivity ratios.

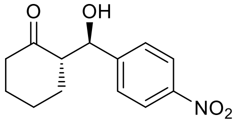

anti-aldol **3a** (major)

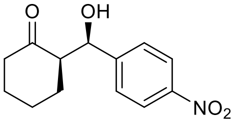

syn-aldol **4a** (minor)

Diastereoselectivity: The *anti-/syn*-aldol ratio was determined as 14.3:1 from the benzylic proton resonances at 4.89 ppm (d, *anti*-aldol product) and 5.48 ppm (d, *syn*-aldol product) in the crude ^{1}H NMR spectrum (Figure S44). For the chemical shift literature values, see page 5 (product 3e) of the Supporting Information within: Martínez-Castañeda, Á.; Rodríguez-Solla, H.; Concellón, C.; del Amo, V. TBD/Al_2O_3: a Novel Catalytic System for Dynamic Intermolecular Aldol Reactions that Exhibit Complex System Behaviour. *Org. Biomol. Chem.* **2012**, *10*, 1976–1981.

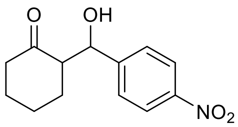

aldol products **3a**/**4a**

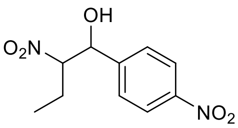

Henry products

Chemoselectivity: The *anti*- and *syn*-aldol/*anti*- and *syn*-Henry product ratio was determined to be >19:1, based on a lack of observable resonance patterns for the *anti*-Henry product at 5.33 ppm (d, benzylic proton) and *syn*-Henry product at 5.18 ppm (d, benzylic proton), see Figure S44. For literature regarding Henry product chemical shifts, see pages 19-20 (compound **11l**), within the Supporting Information of Blay, G.; Domingo, L. R.; Hernández-Olmos, V.; Pedro, J. R. New Highly Asymmetric Henry Reaction Catalyzed by Cu^{II} and a C_1-Symmetric Aminopyridine Ligand, and Its Application to the Synthesis of Miconazole. *Chem. Eur. J.* **2008**, *14*, 4725-4730.

Purification and yield: Silica gel chromatography (25 mm column outer diameter, 18 cm silica bed height) was performed using gradient elution (10 to 25 vol% EtOAc in petroleum ether). The crude product was loaded onto the column in a minimum volume of 50 vol% EtOAc in petroleum ether. A light-yellow solid weighing 226.1 mg (MW = 249.27 g/mol, 0.907 mmol, 91% yield) of the *anti*- and *syn*-aldol diastereomers was isolated.

TLC: *anti*-aldol product R_f = 0.23; *syn*-aldol product R_f = 0.30 (EtOAc/petroleum ether, 3:7).

99% *ee*: Chiralcel OD-H chiral HPLC column, *i*PrOH/n-hexane (7:93), flow rate = 1.0 mL/min, λ = 254 nm, injection volume = 20 μL, the sample was dissolved in 10 vol% *i*PrOH/n-hexane with a concentration of ≈ 1 mg/mL; *anti*-aldol product retention times: t_{major} = 21.1 min, t_{minor} = 32.3 min (Figure S46 & S47).

^{1}H NMR (400 MHz, $CDCl_3$) (ppm) *anti*-aldol product **3a** (Figure S45): δ 8.20 (d, 2H, J = 8.8 Hz), 7.50 (d, 2H, J = 8.8 Hz), 4.89 (dd, 1H, J = 8.4, 3.1 Hz), 4.09 (d, 1H, J = 3.1 Hz), 2.63-2.55 (m, 1H), 2.52-2.46 (m, 1H), 2.41-2.31 (m, 1H), 2.15-2.07 (m, 1H), 1.86-1.78 (m, 1H), 1.75-1.48 (m, 4H), 1.44-1.30 (m, 1H).

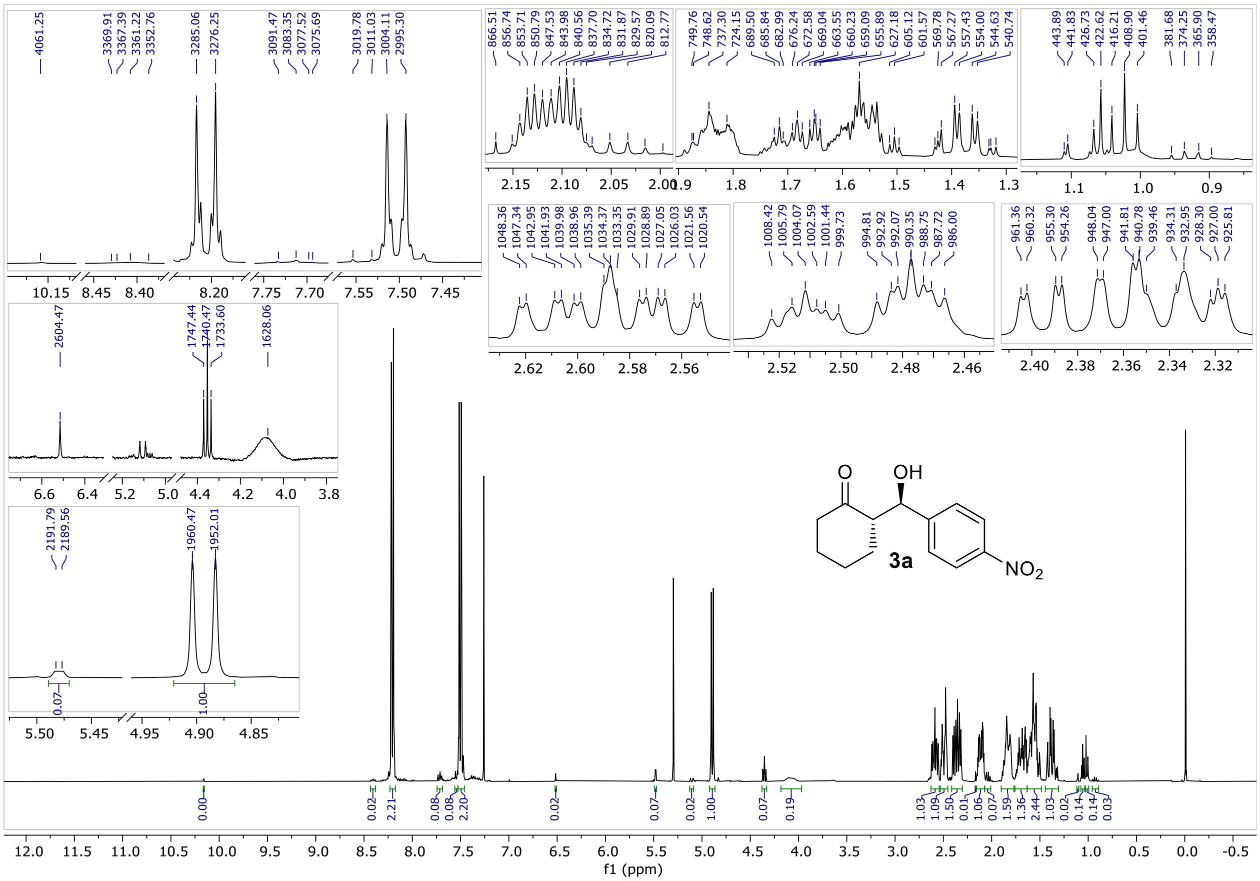

Figure S44. Crude ^{1}H NMR spectrum after high vacuum drying **3a** (above).

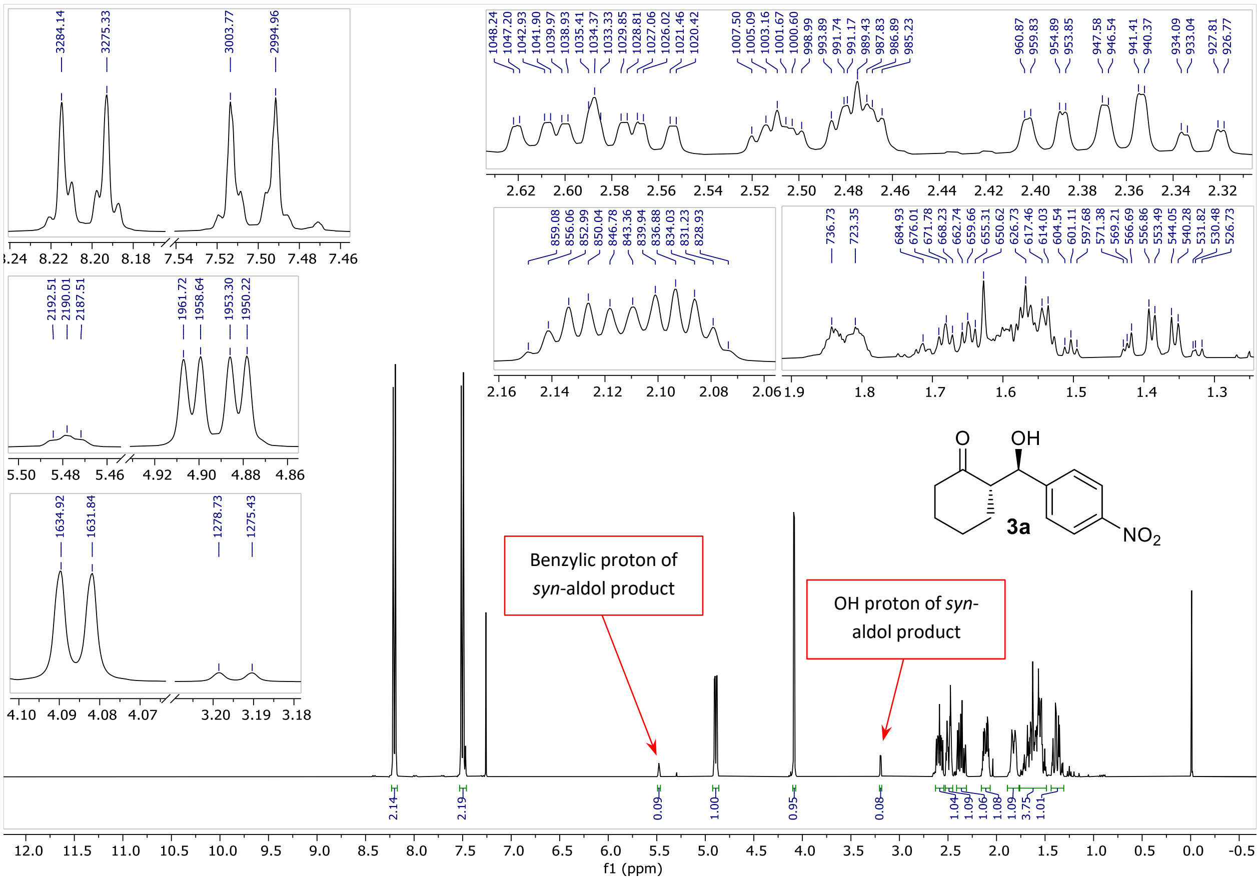

Figure S45. ^{1}H NMR spectrum of purified *anti*-(major) **3a** and *syn*-(minor) **4a** aldol products (above). The red arrows indicate the *syn*-aldol product.

Epimerization of aldol product 3a: Aldol product diastereomers of the structural category produced in this study are prone to epimerization on heating or on exposure to silica gel. Consequently, crude aldol product ^{1}H NMRs can show higher *dr* values than ^{1}H NMRs of the chromatographed aldol products. This, in large part, is why most researchers in this area decided long ago to evaluate the aldol diastereoselectivity using crude ^{1}H NMR spectrums. Furthermore, most researchers isolate the *anti-*/*syn*-aldol products together and report the total yield. For this study, we decided to isolate the *anti*-aldol products and record their yield, and that was possible for all products except *anti*-aldol product **3a**. The *anti*-aldol product **3a** epimerizes during chromatography to an extent that prohibits its isolation free of the *syn*-aldol product **4a**. For related matters, see page S4 within the Supporting Information of: Nugent, T.C.; Umar, M. N.; Bibi, A. Picolylamine as an Organocatalyst Template for Highly Diastereo- and Enantioselective Aqueous Aldol Reactions. *Org. Biomol. Chem.* **2010**, *8*, 4085-4089.

SHIMADZU LabSolutions **Analysis Report**

ADV-A45-7% IPA/nHex 5uL 1mL/min 12May2022

Sample Name	: A46 (RACE)7% IPAnHex 5uL1mLmin 26May 2		
Sample ID	:		
Data Filename	: A46 (RACE)7% IPAnHex 5uL1mLmin 26May 2.lcd		
Method Filename	: trial.lcm		
Batch Filename	:		
Vial #	: 1-1	Sample Type	: Unknown
Injection Volume	: 20 uL		
Date Acquired	: 5/26/2022 2:38:31 PM	Acquired by	: System Administrator
Date Processed	: 5/26/2022 3:28:36 PM	Processed by	: System Administrator

ADV-A46-7% IPA/nHex 5uL 1mL/min 12May2022

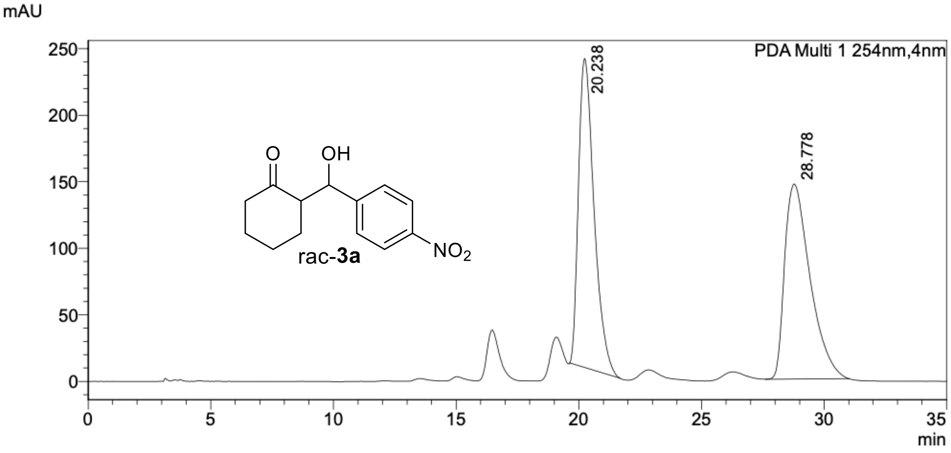

ADV-A45-7% IPA/nHex 5uL 1mL/min 12May2022

PDA Ch1 254nm

Peak#	Ret. Time	Area	Height	Area%
1	20.238	10283216	232276	49.316
2	28.778	10568658	146536	50.684
Total		20851874	378812	100.000

C:\Users\Shimadzu\Desktop\ADV\Competition aldol samples\A46 (RACE)7% IPAnHex 5uL1mLmin 26May 2.lcd

Figure S46. HPLC chromatogram of the racemic *anti*-aldol product **3a** (above).

Analysis Report

SHIMADZU LabSolutions

<Sample Information>

Sample Name	: RL1-VI-7%IPANHEX-1000PPM-SAM[10%IPANHEX]		
Data Filename	: RL1-VI-7%IPANHEX-1000PPM-SAM[10%IPANHEX].lcd		
Method Filename	: RL1-7%IPANHEX-1000PPM-SAM[10%IPANHEX].lcm		
Vial #	: 1-11	Sample Type	: Unknown
Injection Volume	: 20 uL		
Date Acquired	: 10/26/2024 12:44:09 PM	Acquired by	: System Administrator
Date Processed	: 10/26/2024 1:24:12 PM	Processed by	: System Administrator

<Chromatogram>

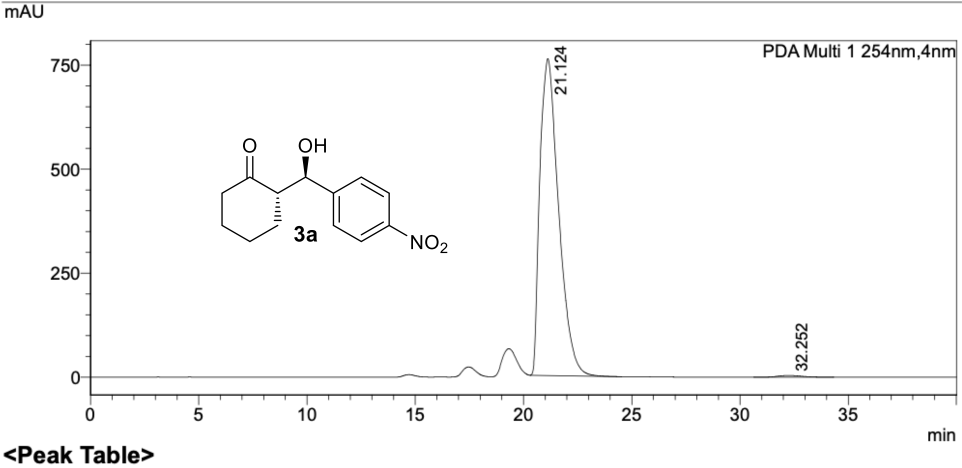

<Peak Table>

PDA Ch1 254nm

Peak#	Ret. Time	Area	Height	Area%
1	21.124	45884735	761729	99.281
2	32.252	332337	4186	0.719
Total		46217072	765915	100.000

C:\LabSolutions\Data\Project1\Data\RUSLAN\RL1-VI-7%IPANHEX-1000PPM-SAM[10%IPANHEX].lcd

Figure S47. HPLC chromatogram of the enantioenriched *anti*-aldol (major) product **3a** (above).

Table 2, entry 6: Competition reaction between cyclohexanone and 1-nitropropane for the limiting reagent methyl 4-formylbenzoate

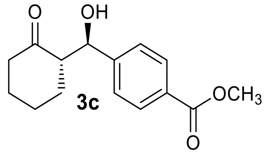

Methyl 4-((R)-hydroxy((S)-2-oxocyclohexyl)methyl)benzoate (3c):
To a clean, screw cap, V-shaped reaction vessel (5.0 mL) equipped with a small pyramidal stir bar mortar and pestle ground methyl 4-formylbenzoate (MW = 164.16 g/mol, 1.0 equiv, 1.0 mmol, 164.2 mg), cyclohexanone (MW = 98.15 g/mol, 1.5 equiv, 1.5 mmol, 147.2 mg, 155 µL, density = 0.947 g/mL) and 1-nitropropane (MW = 89.09 g/mol, 3.02 equiv, 3.02 mmol, 269.5 mg, 270 µL, density = 0.998 g/mL) were added. The liquid reactants (cyclohexanone and 1-nitropropane) were used to rinse the solid methyl 4-formylbenzoate off the walls as needed. Stirring was initiated, and within 30 sec a fully dissolved and transparent mixture resulted. Next, the stirring was terminated, and *trans*-4-(tert-butyldiphenylsilyloxy)-L-proline (MW = 369.54 g/mol, 2.5 mol%, 0.025 mmol, 9.2 mg) was added. Within 30 sec, distilled deoxygenated water (MW = 18.02 g/mol, 15.0 equiv., 15.0 mmol, 270.3 mg, 270 µL) was carefully added to ensure minimal disruption of the concentrated organic layer. Within minutes, the concentrated organic phase turned milky white but at the time of quenching, 40 h, the aqueous and organic phase were both transparent and colorless. Note: Stirring was maintained such that the contents of the vessel did not splash against the vessel wall, but the phase boundary was gently agitated. See Section 2 for the work-up procedure.

This compound was previously synthesized and characterized, see below.

Crude product ^{1}H NMR analysis (Figure S48, see below) allowed determination of the diastereo- and chemoselectivity ratios.

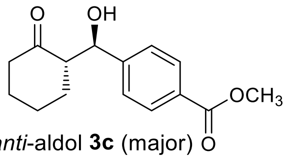

anti-aldol **3c** (major)

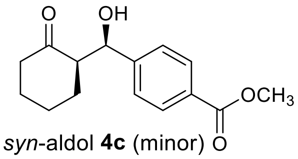

syn-aldol **4c** (minor)

Diastereoselectivity: The *anti-/syn*-aldol ratio was determined as 10:1 based on the benzylic proton resonances at 4.84 ppm (d, *anti*-aldol product) and 5.44 ppm (d, *syn*-aldol product) in the crude ^{1}H NMR spectrum (Figure S48). For the chemical shift literature values, see page S2 (product **3c**) of the Supporting Information within: Mase, N.; Nakai, Y.; Ohara, N.; Yoda, H.; Takabe, K.; Tanaka, F.; Barbas, C. F., III. Organocatalytic Direct Asymmetric Aldol Reactions in Water. *J. Am. Chem. Soc.* **2006**, *128*, 734–735.

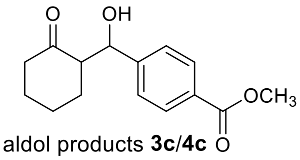

aldol products **3c**/**4c**

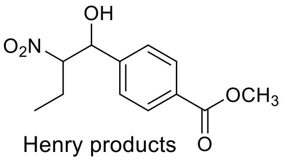

Henry products

Chemoselectivity: The *anti*- and *syn*-aldol/*anti*- and *syn*-Henry product ratio is >19:1, based on the following assumptions (Figure S48). While both Henry products have been reported on multiple occasions, the ^{1}H NMR data was never provided. This noted, related compounds have been reported, *e.g.*, the corresponding nitroethane *anti*-Henry product has been reported with a resonance at 4.69 ppm (dt, $C\underline{H}NO_2$) and 5.47 ppm (dd, benzylic proton), see page S7 within the Supporting Information of: Nitabaru, T.; Nojiri, A.; Kobayashi, M.; Kumagai, N.; Shibasaki, M. *anti*-Selective Catalytic Asymmetric Nitroaldol Reaction via a Heterobimetallic Heterogeneous Catalyst. *J. Am. Chem. Soc.* **2009**, *131*, 13860–13869. Furthermore, the 1-nitropropane Henry product of benzaldehyde has been reported with resonances at 4.95 ppm (d, *syn*-product benzylic proton) and 5.10 ppm (d, *anti*-product benzylic proton), see page S13 within the Supporting Information of: Li, Y.; Deng, P.; Zeng, Y.; Xiong, Y.; Zhou, H. *anti*-Selective Asymmetric Henry Reaction Catalyzed by a Heterobimetallic Cu–Sm–Aminophenol Sulfonamide Complex. *Org. Lett.* **2016**, *18*, 1578–1581.

Purification and yield: Silica gel chromatography (25 mm column outer diameter, 16 cm silica bed height) was performed using isocratic elution (10 vol% EtOAc in petroleum ether). The crude product was loaded onto the column in 1:1 ratio of minimum volume EtOAc and petroleum ether. The *anti*-aldol product, a white solid, was isolated as a single diastereomer (221.1 mg, MW = 262.31 g/mol, 0.842 mmol, 84% yield).

TLC: *anti*-aldol product R_f = 0.23, *syn*-aldol product R_f = 0.32 (EtOAc/petroleum ether, 1:4).

99% *ee*: Chiralcel OD-H chiral HPLC column, iPrOH/n-hexane (5:95), flow rate = 1.0 mL/min, λ = 254 nm, injection volume = 20 μL, the sample was dissolved in 5 vol% *i*PrOH/n-hexane with a concentration of ≈1 mg/mL; *anti*-aldol product retention times: t_{minor}= 18.8, t_{major}= 21.1 (Figure S50 & S51).

^{1}H NMR (400 MHz, $CDCl_3$) (ppm) *anti*-aldol product **3c** (Figure S49): δ 8.02 (d, 2H, J = 8.2 Hz), 7.39 (d, 2H, J = 8.3 Hz), 4.84 (dd, 1H, J = 8.7, 2.9 Hz), 4.02 (d, 1H, J = 2.8 Hz), 3.91 (s, 3H), 2.63-2.55 (m, 1H), 2.51-2.45 (m, 1H), 2.41-2.30 (m, 1H), 2.13-2.05 (m, 1H), 1.84-1.75 (m, 1H), 1.72-1.47 (m, 3H), 1.38-1.23 (m, 2H).

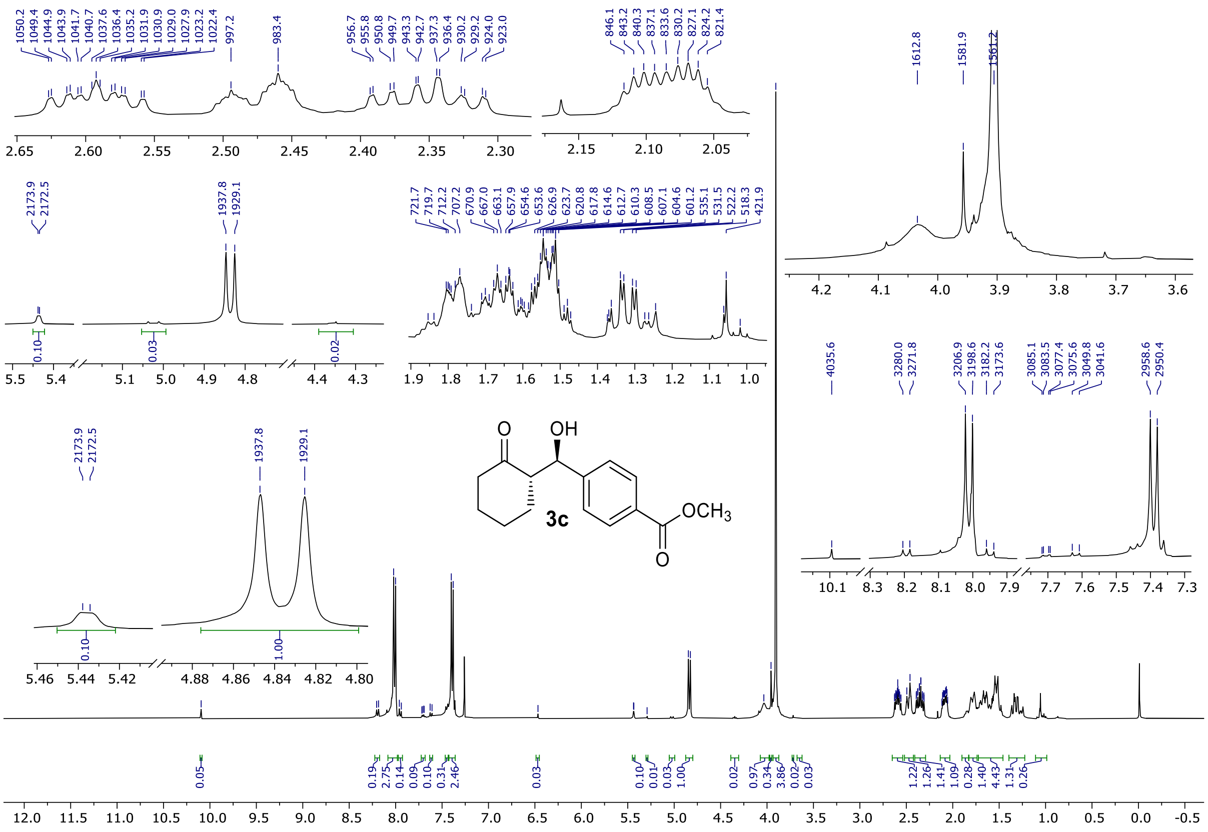

Figure S48. Crude ^{1}H NMR spectrum after high vacuum drying **3c** (above).

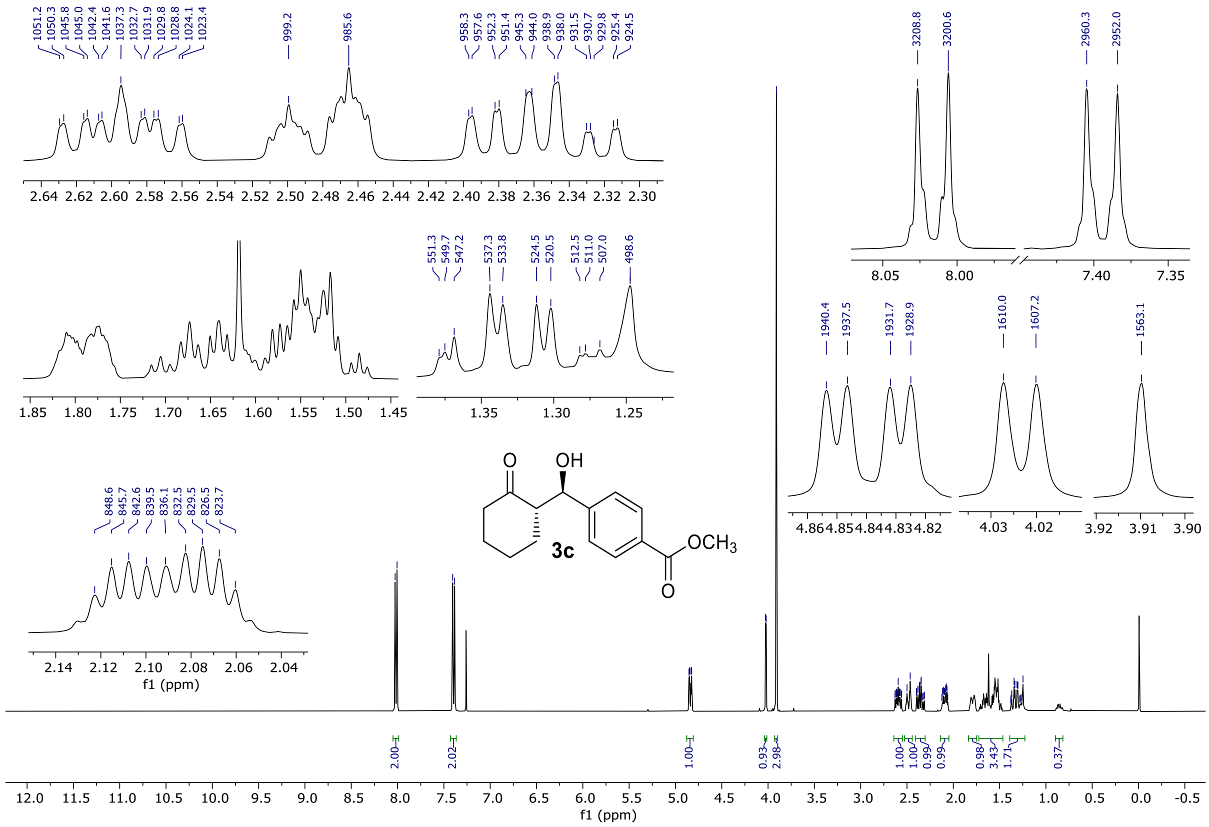

Figure S49. ^{1}H NMR spectrum of the purified *anti*-aldol (major) product **3c** (above).

SHIMADZU LabSolutions

Analysis Report

<Sample Information>

Sample Name	: SSZ02 RACE 2mgmL 5%IPAnHex 20uL 1mLmin 17nov22		
Sample ID	:		
Data Filename	: SSZ02 RACE 2mgmL 5%IPAnHex 20uL 1mLmin 17nov22.lcd		
Method Filename	: trial.lcm		
Batch Filename	:		
Vial #	: 1-4	Sample Type	: Unknown
Injection Volume	: 20 uL		
Date Acquired	: 11/17/2022 12:56:08 PM	Acquired by	: System Administrator
Date Processed	: 11/17/2022 1:34:19 PM	Processed by	: System Administrator

<Chromatogram>

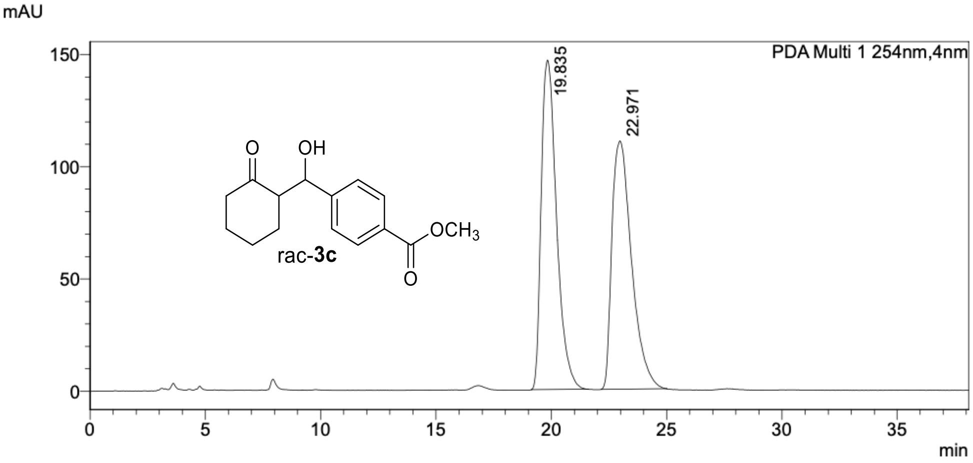

<Peak Table>

PDA Ch1 254nm

Peak#	Ret. Time	Area	Height	Area%
1	19.835	6780885	146718	50.893
2	22.971	6542886	110549	49.107
Total		13323772	257267	100.000

C:\Users\Shimadzu\Desktop\SZ\HPLC\Runs\SSZ02 RACE 2mgmL 5%IPAnHex 20uL 1mLmin 17nov22.lcd

Figure S50. HPLC chromatogram of the racemic *anti*-aldol product **3c** (above).

SHIMADZU LabSolutions **Analysis Report**

<Sample Information>

Sample Name	: AL-15-5%IPANHEX-1000PPM		
Data Filename	: AL-15-5%IPANHEX-1000PPM.lcd		
Method Filename	: RL5-5%IPANHEX-1000PPM-SAM[5%IPANHEX].lcm		
Vial #	: 1-4	Sample Type	: Unknown
Injection Volume	: 20 uL		
Date Acquired	: 9/30/2024 4:13:37 PM	Acquired by	: System Administrator
Date Processed	: 9/30/2024 4:43:39 PM	Processed by	: System Administrator

<Chromatogram>

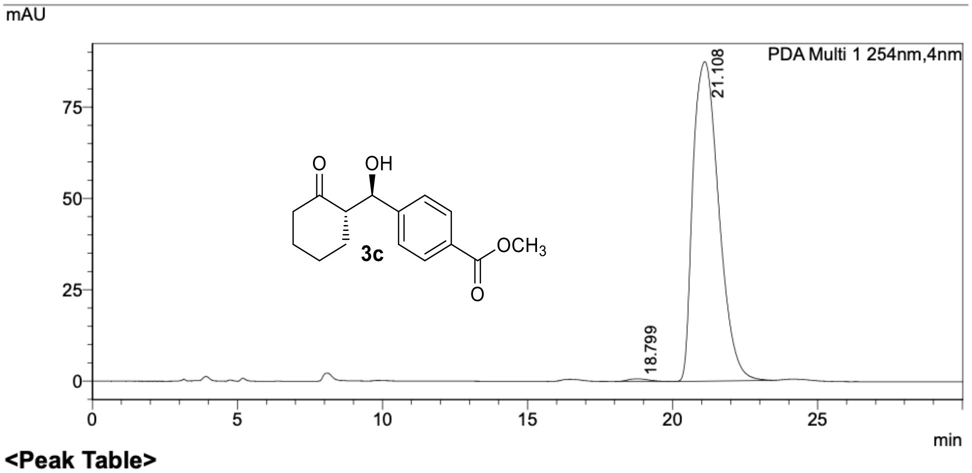

<Peak Table>

PDA Ch1 254nm

Peak#	Ret. Time	Area	Height	Area%
1	18.799	36069	719	0.661
2	21.108	5417248	87440	99.339
Total		5453317	88160	100.000

C:\LabSolutions\Data\Project1\Data\RUSLAN\AL-15-5%IPANHEX-1000PPM.lcd

Figure S51. HPLC chromatogram of the enantioenriched *anti*-aldol (major) product **3c** (above).

Table 2, entry 7: Competition reaction between cyclohexanone and 1-nitropropane for the limiting reagent 3-chlorobenzaldehyde

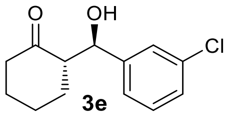

(S)-2-((R)-(3-chlorophenyl)(hydroxy)methyl)cyclohexan-1-one (**3e**):
To a clean, screw cap, V-shaped reaction vessel (5.0 mL) equipped with a small pyramidal stir bar and freshly purified (see Section 2) 3-chlorobenzaldehyde (MW = 140.57 g/mol, 1.00 equiv, 1.00 mmol, 140.23 mg, 113 µL, density = 1.241 g/mL), cyclohexanone (MW = 98.15 g/mol, 1.50 equiv, 1.50 mmol, 146.79 mg, 155 µL, density = 0.947 g/mL) and 1-nitropropane (MW = 89.09 g/mol, 3.00 equiv, 3.00 mmol, 267.46 mg, 268 µL, density = 0.998 g/mL) were added. Stirring was initiated, and the mixture was transparent. Next, the stirring was terminated, and *trans*-4-(tert-butyldiphenylsilyloxy)-L-proline (MW = 369.54 g/mol, 2.5 mol%, 0.025 mmol, 9.2 mg) was added. Within 30 sec, distilled deoxygenated water (MW = 18.02 g/mol, 15.0 equiv., 15.0 mmol, 270 mg, 270 µL) was carefully added to ensure minimal disruption of the concentrated organic layer. Within minutes, the concentrated organic phase turned milky white but at the time of quenching, 49 h, the aqueous and organic phase were both transparent and colorless. Workup after 30 and 40 h of starting the reaction showed a higher amount of the aldehyde compared to workup after 49 h. Note: Stirring was maintained such that the contents of the vessel did not splash against the vessel wall, but the phase boundary was gently agitated. See Section 2 for the work-up procedure.

This compound was previously synthesized and characterized, see below.

Crude product ^{1}H NMR analysis (Figure S52, see below) allowed determination of the diastereo- and chemoselectivity ratios.

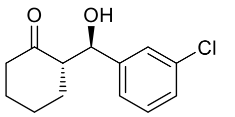

anti-aldol (major) **3e**

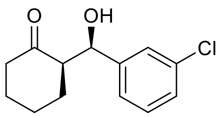

syn-aldol (minor) **4e**

Diastereoselectivity: The *anti-/syn*-aldol ratio was determined as 12.5:1 from the benzylic proton resonances at 4.76 ppm (d, *anti*-aldol product) and 5.37 ppm (d, *syn*-aldol product) in the crude ^{1}H NMR spectrum (Figure S52). For the chemical shift literature values, see page S6 (product 3f) of the Supporting Information within: Martínez-Castañeda, Á.; Rodríguez-Solla, H.; Concellón, C.; del Amo, V. TBD/Al_2O_3: A Novel Catalytic System for Dynamic Intermolecular Aldol Reactions that Exhibit Complex System Behaviour. *Org. Biomol. Chem.* **2012**, *10*, 1976-1981.

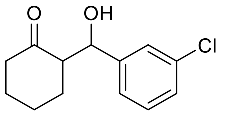

aldol products **3e**/**4e**

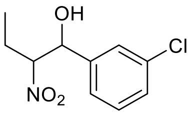

Henry products

Chemoselectivity: The *anti*- and *syn*-aldol/*anti*- and *syn*-Henry product ratio is >19:1, based on the following assumptions from our crude ^{1}H NMR (Figure S52). While both Henry products have been reported, the ^{1}H NMR data was never provided. This noted, related compounds have been reported, *e.g.*, the corresponding nitroethane *anti*-Henry product has been reported with a resonance at 4.67 ppm (dt, C<u>H</u>NO_2) and 5.39 ppm (d, benzylic proton), see page S11 within the Supporting information of: Xu, K.; Lai, G.; Zha, Z.; Pan, S.; Chen, H.; Wang, Z. A Highly *anti*-Selective Asymmetric Henry Reaction Catalyzed by a Chiral Copper Complex: Applications to the Syntheses of (+)-Spisulosine and a Pyrroloisoquinoline Derivative. *Chem. Eur. J.* **2012**, *18*, 12357–12362.
Furthermore, the 1-nitropropane Henry product of benzaldehyde has been reported with resonances at 4.95 ppm (d, *syn*-product benzylic proton) and 5.10 ppm (d, *anti*-product benzylic proton), see page S13 within the Supporting Information of: Li, Y.; Deng, P.; Zeng, Y.; Xiong, Y.; Zhou, H. *anti*-Selective Asymmetric Henry Reaction Catalyzed by a Heterobimetallic Cu−Sm−Aminophenol Sulfonamide Complex. *Org. Lett.* **2016**, *18*, 1578−1581.

Purification and yield: The crude product was loaded onto a silica gel (230-400 mesh) column (16 cm in height, 2.5 cm in diameter pre-wetted with EtOAc/petroleum ether (1:19). The crude product was loaded onto the column in a minimum volume of EtOAc. The mobile phase elution began with EtOAc/petroleum ether (1:19). This solvent ratio was maintained until the starting materials were removed from the column. The polarity of the mobile phase was then raised to EtOAc/petroleum ether (1:13) to remove the syn-aldol product. The anti-aldol product eluted soon after use of EtOAc/petroleum ether (1:9). Concentration of the pure fractions provided a white solid (MW= 238.71 g/mol, 205 mg, 0.859 mmol, 86% yield) of the *anti*-aldol product **3e**.

TLC: *anti*-aldol product R_f = 0.22, *syn*-aldol product R_f = 0.31 (EtOAc/petroleum ether, 3:20). Note: UV at 254 nm was ineffective for observation of the product so CAM staining was used.

99% *ee*: Chiralcel OD-H chiral HPLC column, iPrOH/n-hexane (5:95), flow rate = 1.0 mL/min, λ = 210 nm, injection volume = 10 μL, the sample was dissolved in 5 vol% *i*PrOH/n-hexane with a concentration of ≈1 mg/mL; *anti*-aldol product retention times: t_{minor}= 16.5, t_{major}= 11.4 (Figure S54 & S55).

Note: Column chromatography for the racemate needed to be performed twice. Initially, 15 vol% EtOAc/Hexane (isocratic) was used. However, TLC with CH_2Cl_2 revealed impurity bands that were not visible with EtOAc/Hexane. Therefore, a second purification was done using 100 vol% CH_2Cl_2.

^{1}H NMR (400 MHz, CDCl$_3$) (ppm) *anti*-aldol product **3e** (Figure S53): δ 7.35-7.15 (m, 5H), 4.76 (dd, 1H, J = 8.8, 2.9 Hz), 4.01 (d, 1H, J = 2.9 Hz), 2.63-2.53 (m, 1H), 2.52-2.43 (m, 1H), 2.42-2.32 (m, 1H), 2.15-2.06 (m, 1H), 1.85-1.49 (m, 1H), 1.73-1.50 (m, 4H), 1.37-1.25 (m, 1H).

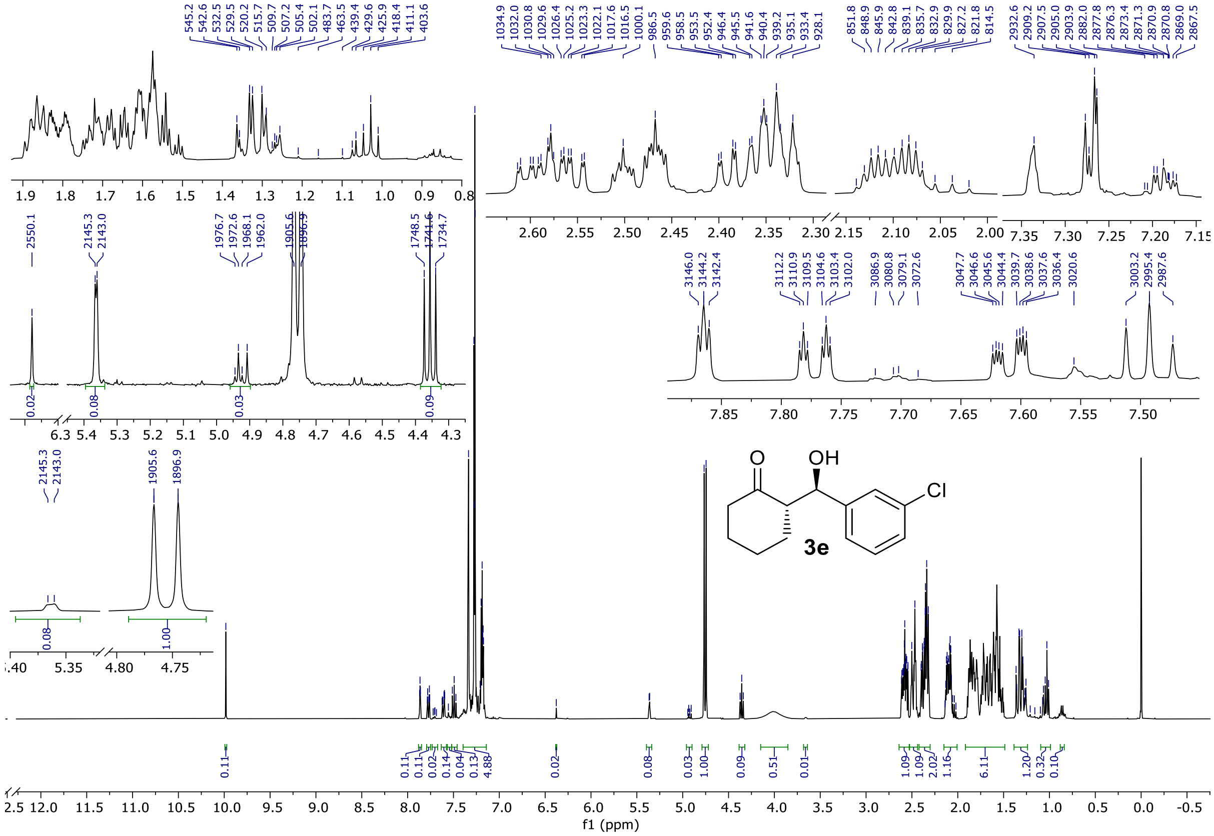

Figure S52. Crude ^{1}H NMR spectrum after high vacuum drying **3e** (above).

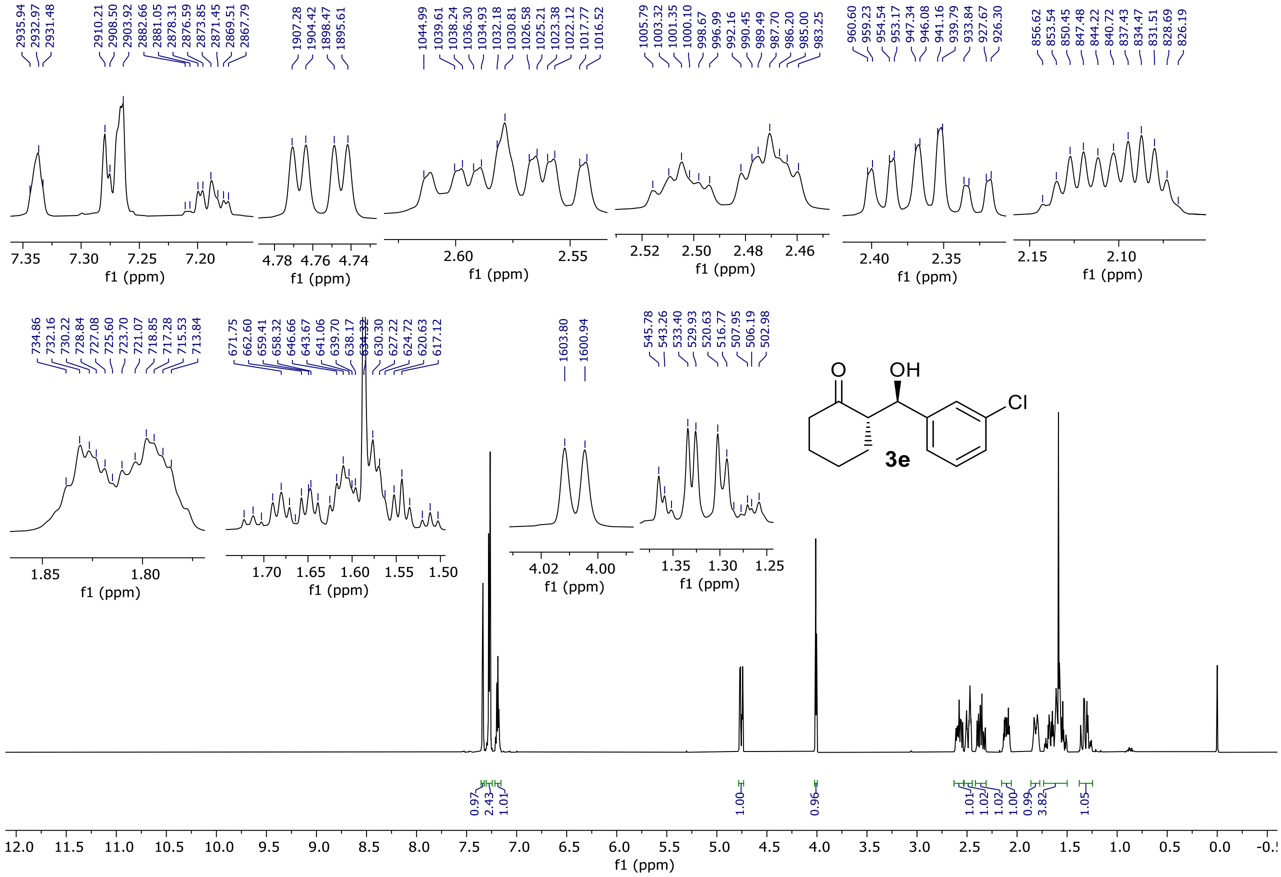

Figure S53. ^{1}H NMR spectrum of the purified *anti*-aldol (major) product **3e** (above).

Data File D:\DATA\NUGENT\AL24 RAC 5%IPANHEX 06FEB25 RE.D
Sample Name: AL24 RAC 5%IPAnHex 06feb25 re

===

Acq. Operator : Seq. Line : 2
Acq. Instrument : Instrument 1 Location : P1-A-03
Injection Date : 2/6/2025 1:35:39 PM Inj : 1
Inj Volume : 20 µl
Acq. Method : D:\Methods\Nugentlab_Patrick\AIDA.m
Last changed : 2/6/2025 1:34:31 PM
Analysis Method : D:\Methods\Nugentlab_Patrick\AIDA254NM1.m
Last changed : 2/6/2025 2:01:03 PM
(modified after loading)

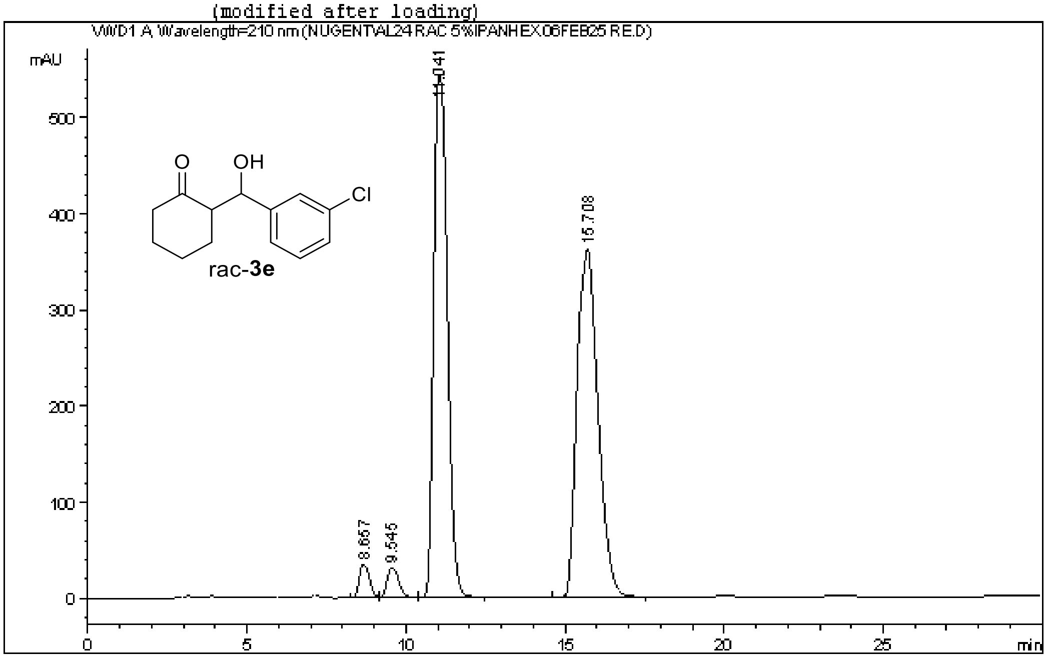

===

Area Percent Report

===

Sorted By : Signal
Multiplier : 1.0000
Dilution : 1.0000
Use Multiplier & Dilution Factor with ISTDs

Signal 1: VWD1 A, Wavelength=210 nm

Peak #	RetTime [min]	Type	Width [min]	Area mAU *s	Height [mAU]	Area %
1	8.657	BV	0.3643	785.40881	34.54132	2.2828
2	9.545	VB	0.4170	804.99719	30.80102	2.3397
3	11.041	BB	0.4747	1.62915e4	543.91724	47.3515
4	15.708	BB	0.7207	1.65235e4	361.77737	48.0260
Totals :				3.44054e4	971.03695	

===

*** End of Report ***

Instrument 1 2/6/2025 2:06:54 PM

Page 1 of 1

Figure S54. HPLC chromatogram of the racemic *anti*-aldol products **3e** (above).

SHIMADZU LabSolutions

Analysis Report

<Sample Information>

Sample Name	: kd-11-107-anti		
Data Filename	: kd-11-107-anti.lcd		
Method Filename	: RL5-5%IPANHEX-1000PPM-SAM[5%IPANHEX].lcm		
Vial #	: 1-31	Sample Type	: Unknown
Injection Volume	: 10 uL		
Date Acquired	: 6/28/2025 1:42:52 PM	Acquired by	: System Administrator
Date Processed	: 8/12/2025 9:37:52 AM	Processed by	: System Administrator

<Chromatogram>

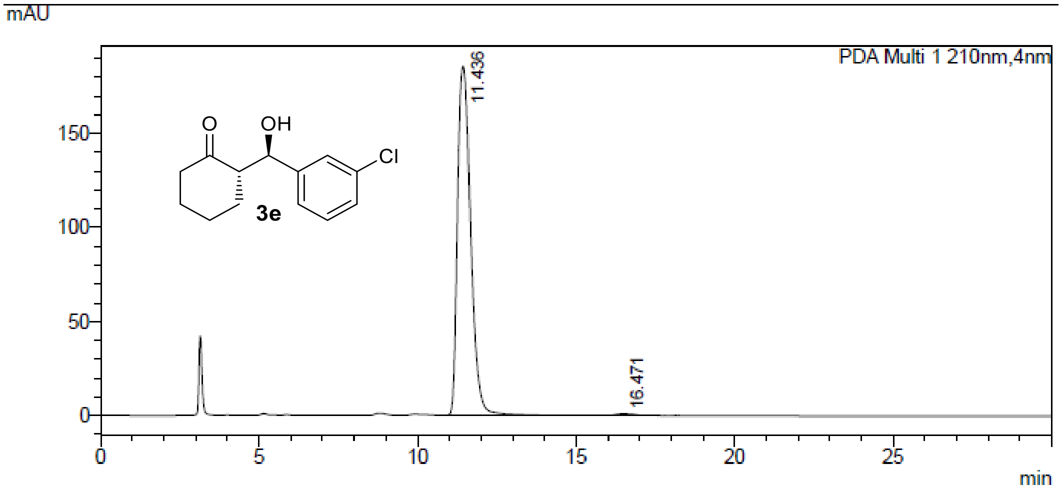

<Peak Table>

PDA Ch1 210nm

Peak#	Ret. Time	Area	Height	Area%
1	11.436	5322995	185717	99.366
2	16.471	33984	861	0.634
Total		5356979	186577	100.000

Figure S55. HPLC chromatogram of the enantioenriched *anti*-aldol (major) **3e** (above).

Table 2, entry 8: Competition reaction between 4-methylcyclohexanone and 1-nitropropane for the limiting reactant 4-nitrobenzaldehyde

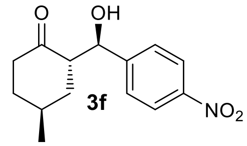

(2*S*,4*S*)-2-((*R*)-hydroxy(4-nitrophenyl)methyl)-4-methylcyclohexan-1-one (3f):
To a clean, screw cap, V-shaped reaction vessel (5.0 mL) equipped with a small pyramidal stir bar, mortar and pestle ground 4-nitrobenzaldehyde (MW = 151.12 g/mol, 1.00 equiv, 1.00 mmol, 151 mg), 4-methylcyclohexanone (MW = 112.17 g/mol, 2.00 equiv, 2.00 mmol, 224 mg, 245 µL, density 0.914 g/mL), and 1-nitropropane (MW = 89.09 g/mol, 2.02 equiv, 2.02 mmol, 180 mg, 180 µL, density 0.998 g/mL) were added. The liquid reactants (4-methylcyclohexanone and 1-nitropropane) were used to rinse the solid 4-nitrobenzaldehyde off the walls as needed. This mixture was gently stirred for 5 min resulting in a beige colored organic layer containing undissolved, finely divided aldehyde. The stirring was terminated and the *trans*-4-(*tert*-butyldiphenylsilyloxy)-L-proline catalyst (MW = 369.54 g/mol, 5.0 mol%, 0.050 mmol, 18.6 mg) was added to the reaction vessel. Within 30 sec after catalyst addition, distilled deoxygenated water (MW = 18.02 g/mol, 14.98 equiv, 14.98 mmol, 270 mg, 270 µL) was added with minimal disruption of the concentrated organic layer. The mixture was stirred such that the contents of the vessel did not splash up and against the walls, but the phase boundary was gently agitated. For observational purposes, the stirring was stopped after 5 min and two phases formed. The resulting biphasic system contained undissolved, finely divided solids in both phases which were both grey in color. This was repeated at 20 min and again a bottom phase was noted with finely divided solids in it, but now a slightly yellow colored transparent top phase appeared with solid material within it. After 24 h the stirring was terminated, revealing a biphasic system, at which both layers contained a yellow colored precipitate. See Section 2 for the work-up procedure.

This compound was previously synthesized and characterized, see below.

Crude product ^{1}H NMR analysis (Figure S56, see below) allowed determination of the following diastereo- and chemoselectivity ratios.

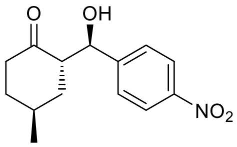

anti-aldol (major) **3f**

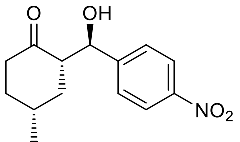

anti-aldol (minor) **3f**

Diastereoselectivity (*anti*/*anti*): The *anti*(major)-/*anti*(minor)-aldol ratio was determined as >19:1 from the benzylic proton resonances at 4.92 ppm (d, *anti*(major)-aldol product) and 4.87 ppm (d, *anti*(minor)-aldol product) in the crude ^{1}H NMR spectrum (Figure S56). For the chemical shift literature values, see the manuscript text and page 82 of the Supporting Information within: Nugent, T. C.; Spiteller, P.; Hussain, I.; Hussein, H. A. E. D.; Najafian, F. T. A Catalyst-Directed Remote Stereogenic Center Switch During the Site-Selective Aldol Desymmetrization of Cyclohexanone-Based Diketones. *Adv. Synth. Catal.* **2016**, *358*, 3706-3713.

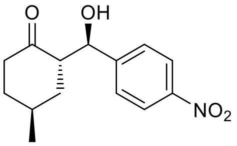

anti-aldol (major) **3f**

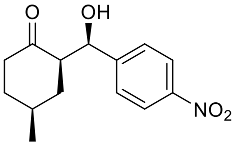

syn-aldol (minor) **4f**

Diastereoselectivity (*anti*/*syn*): The *anti*(major)-/*syn*(major)-aldol ratio was determined as 12.5:1 based on the benzylic proton resonances at 4.92 ppm (d, *anti*(major)-aldol product) and 5.49 ppm (d, *syn*(major)-aldol product) in the crude ^{1}H NMR spectrum (Figure S56). For the chemical shift literature values of *syn*-aldol product, see page 3676 of the manuscript within: Qian, Y.; Zheng, X.; Wang, Y. A Green and Efficient Asymmetric Aldol Reaction Catalyzed by a Chiral Anion Modified Ionic Liquid. *Eur. J. Org. Chem.* **2010**, 3672-3677.

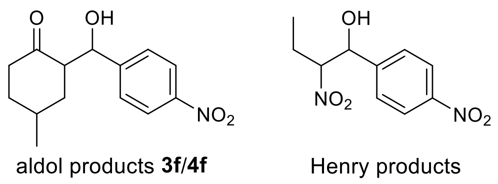

Chemoselectivity: The *anti*- and *syn*-aldol/Henry product ratio was determined to be >19:1, based on a lack of observable resonance patterns for the Henry product at 5.17 ppm (dd, *syn*-Henry product benzylic proton) and 5.32 ppm (d, *anti*-Henry product benzylic proton), see Figure S56. For the Henry product chemical shift literature values, see product 6c (specifically p 4594) and the Supporting Information within: Toussaint, A.; Pfaltz, A. Asymmetric Henry Reactions Catalyzed by Metal Complexes of Chiral Boron-Bridged Bisoxazoline (Borabox) Ligands. *Eur. J. Org. Chem.* **2008**, *27*, 4591-4597.

Purification and yield: Silica gel chromatography (25 mm column outer diameter, 18 cm silica bed height) was performed using isocratic elution (1 vol% acetone in CH_2Cl_2). The crude product was loaded onto the column in a minimum volume of CH_2Cl_2. The *anti*(major)-aldol product was isolated as an off-white solid (172 mg, MW = 263.29 g/mol, 0.653 mmol, 65% yield).

TLC: *anti*(major)-aldol product R_f = 0.26 (acetone/dichloromethane, 1:99).

99% *ee*: Chiralcel OD-H chiral HPLC column, iPrOH/n-hexane (5:95), flow rate = 1.0 mL/min, λ = 254 nm, injection volume = 20 µL, the sample was dissolved in 5 vol% iPrOH/n-hexane with a concentration of ≈ 1 mg/mL; *anti*(major)-aldol product retention times: t_{major} = 38.1 min, t_{minor} = 52.5 min. (Figure S58 & S59).

^{1}H NMR (400 MHz, $CDCl_3$) (ppm) *anti*(major)-aldol product **3f** (Figure S57): δ 8.22 (d, 2H, J = 8.8 Hz), 7.50 (d, 2H, J = 8.8 Hz), 4.92 (dd, 1H, J = 8.6, 3.2 Hz), 3.92 (d, 1H, J = 3.2 Hz), 2.70-2.78 (m, 1H), 2.50-2.59 (m, 1H), 2.35-2.43 (m, 1H), 2.04-2.13 (m, 1H), 1.89-1.99 (m, 1H), 1.75-1.84 (m, 1H), 1.55-1.64 (m, 1H), 1.27-1.35 (m, 1H), 1.06 (d, 3H, J = 7.0 Hz).

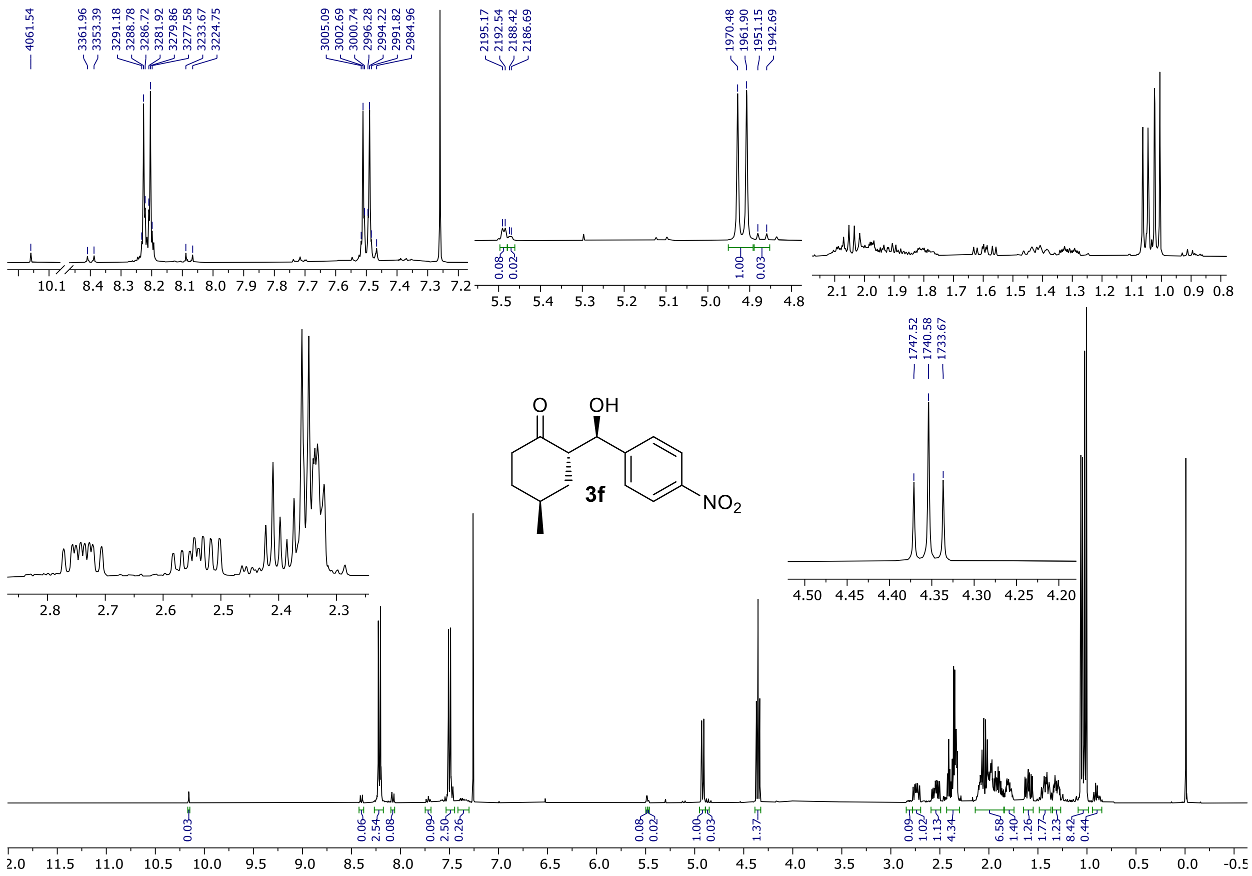

Figure S56. Crude ^{1}H NMR spectrum after short high vacuum drying **3f** (above).

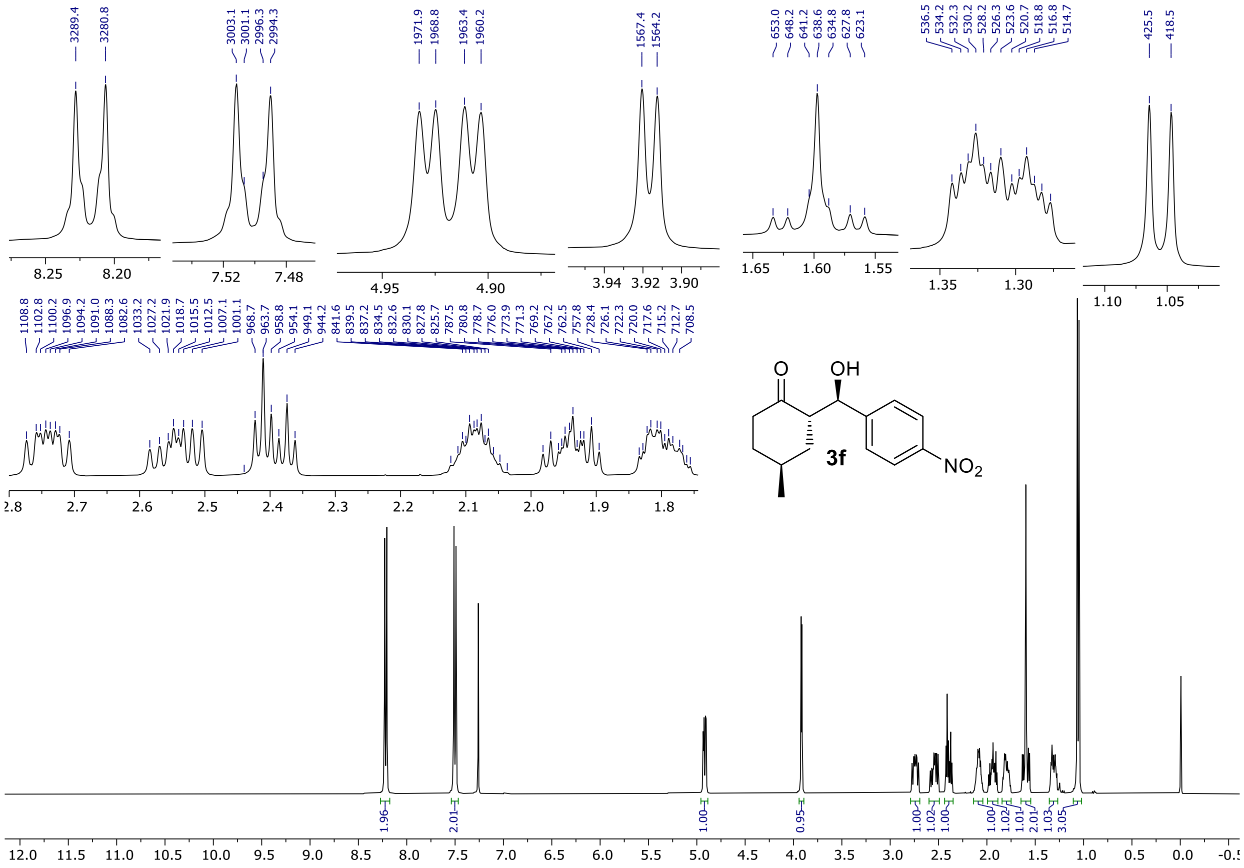

Figure S57. ^{1}H NMR spectrum of the purified *anti*-aldol (major) product **3f** (above).

SHIMADZU LabSolutions

Analysis Report

<Sample Information>

Sample Name	: HAB21 Race 5%IPAnHex 10uL 1mLmin 10Aug
Sample ID	:
Data Filename	: HAB21 Race 5%IPAnHex 10uL 1mLmin 10Aug.lcd
Method Filename	: trial.lcm
Batch Filename	:
Vial #	: 1-3
Sample Type	: Unknown
Injection Volume	: 10 uL
Date Acquired	: 8/10/2022 1:43:42 PM
Acquired by	: System Administrator
Date Processed	: 8/10/2022 2:43:47 PM
Processed by	: System Administrator

<Chromatogram>

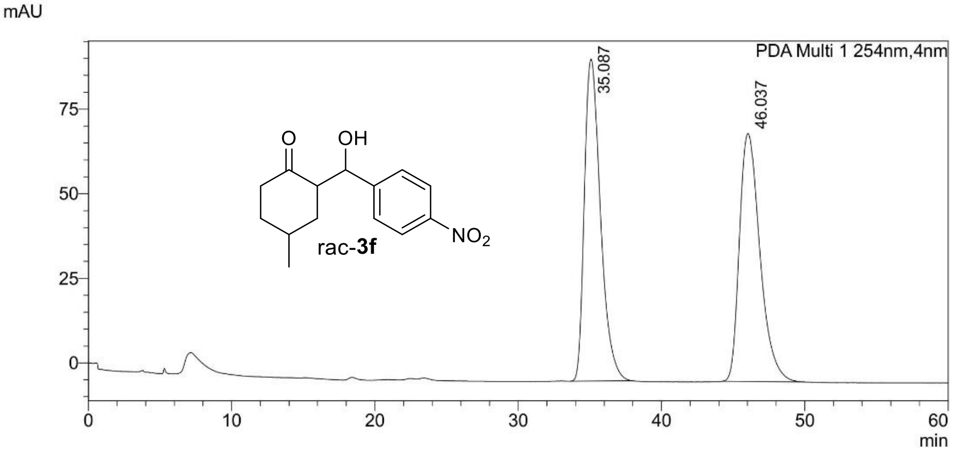

<Peak Table>

PDA Ch1 254nm

Peak#	Ret. Time	Area	Height	Area%
1	35.087	7421140	95157	50.429
2	46.037	7294940	73334	49.571
Total		14716080	168491	100.000

C:\Users\Shimadzu\Desktop\Hanaa\4methcyc4nitro Racemate\HAB21 Race 5%IPAnHex 10uL 1mLmin 10Aug.lcd

Figure S58. HPLC chromatogram of the racemic *anti*-aldol product **3f** (above).

SHIMADZU LabSolutions

Analysis Report

<Sample Information>

Sample Name	: PK-016_purified_2nd		
Data Filename	: PK-016_purified_2nd.lcd		
Method Filename	: PK016-5%_60_min_IPANHEX-1000PPM-SAM[5%IPANHEX].lcm		
Vial #	: 1-9	Sample Type	: Unknown
Injection Volume	: 20 uL		
Date Acquired	: 10/23/2024 2:10:56 PM	Acquired by	: System Administrator
Date Processed	: 10/23/2024 3:10:58 PM	Processed by	: System Administrator

<Chromatogram>

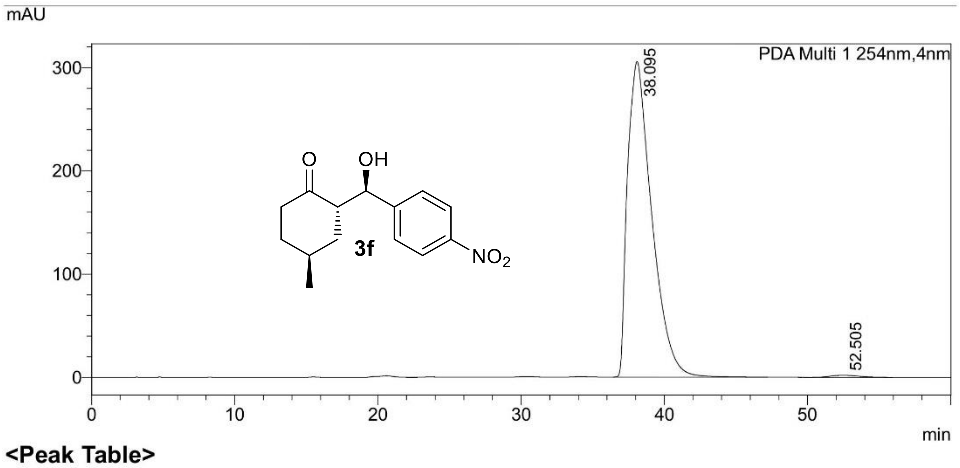

<Peak Table>

PDA Ch1 254nm

Peak#	Ret. Time	Area	Height	Area%
1	38.095	35980999	305621	99.261
2	52.505	267917	2013	0.739
Total		36248916	307634	100.000

C:\LabSolutions\Data\Project1\Data\Patrick\PK-016_purified_2nd.lcd

Figure S59. HPLC chromatogram of the enantioenriched *anti*-aldol (major) product **3f** (above).

Table 2, entry 9: Competition reaction between 2,2-dimethyl-1,3-dioxan-5-one and 1-nitropropane for the limiting reactant 4-nitrobenzaldehyde

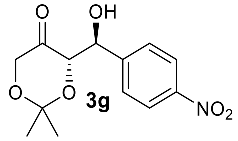

(*S*)-4-((*S*)-hydroxy(4-nitrophenyl)methyl)-2,2-dimethyl-1,3-dioxan-5-one (**3g**):
To a clean, screw cap, V-shaped reaction vessel (5.0 mL) equipped with a small pyramidal stir bar, mortar and pestle ground 4-nitrobenzaldehyde (MW = 151.12 g/mol, 1.00 equiv, 1.00 mmol, 151 mg), 2,2-dimethyl-1,3-dioxan-5-one (MW = 130.14 g/mol, 2.01 equiv, 2.01 mmol, 262 mg, 240 µL, density 1.09 g/mL), and 1-nitropropane (MW = 89.09 g/mol, 2.02 equiv, 2.02 mmol, 180 mg, 180 µL, density 0.998 g/mL) were added. The liquid reactants (2,2-dimethyl-1,3-dioxan-5-one and 1-nitropropane) were used to rinse the solid 4-nitrobenzaldehyde off the walls as needed. This mixture was gently stirred for 5 min resulting in a beige colored organic layer containing undissolved, finely divided aldehyde. The stirring was terminated and the *trans*-4-(*tert*-butyldiphenylsilyloxy)-*L*-proline catalyst (MW = 369.54 g/mol, 5.0 mol%, 0.050 mmol, 18.6 mg) was added to the reaction vessel. Within 30 sec after catalyst addition, deoxygenated brine (MW = 18.02 g/mol, 14.95 equiv of H_2O, 14.95 mmol of H_2O, 305 µL, density 1.20 g/mL, 26.4 wt% NaCl in H_2O) was added with minimal disruption of the concentrated organic layer. The mixture was stirred such that the contents of the vessel did not splash up against the walls, but the phase boundary was gently agitated. For observational purposes, the stirring was stopped after 5 min and two phases formed. The bottom layer was transparent, while the top layer contained finely divided solids and looked off-white. This was repeated at 20 min and again a transparent lower phase was noted but now an amber colored transparent top phase appeared with solid material in it. After 24 h the stirring was terminated, revealing a transparent colorless phase at the bottom and an amber colored top phase full of solids. See Section 2 for the work-up procedure.

This compound was previously synthesized and characterized, see below.

Crude product ^{1}H NMR analysis (Figure S60, see below) allowed determination of the following diastereo- and chemoselectivity ratios.

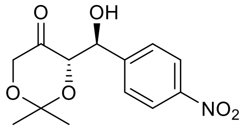

anti-aldol (major) **3g**

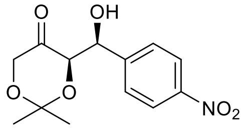

syn-aldol (minor) **4g**

Diastereoselectivity: The *anti-/syn*-aldol ratio was determined as >19:1 from the benzylic proton resonances at 4.99 ppm (d, *anti*-aldol product) and 5.32 ppm (d, *syn*-aldol product) in the crude ^{1}H NMR spectrum (Figure S60). For the chemical shift literature values, see page S9 of the Supporting Information within: Ma, G.; Bartoszewicz, A.; Ibrahem, I.; Córdova, A. Highly Enantioselective Co-Catalytic Direct Aldol Reactions by Combination of Hydrogen-Bond Donating and Acyclic Amino Acid Catalysts. *Adv. Synth. Catal.* **2011**, *353*, 3114-3122.

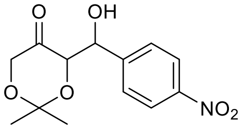

aldol products **3g**/**4g**

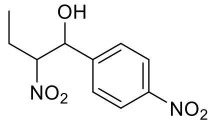

Henry products

Chemoselectivity: The *anti*- and *syn*-aldol/Henry product ratio was determined to be >19:1, based on a lack of observable resonance patterns for the Henry product at 5.17 ppm (dd, *syn*-Henry product benzylic proton) and 5.32 ppm (d, *anti*-Henry product benzylic proton), see our crude ^{1}H NMR spectrum (Figure S60). For the Henry product chemical shift literature values, see product 6c (specifically p 4594) and the Supporting Information within: Toussaint, A.; Pfaltz, A. Asymmetric Henry Reactions Catalyzed by Metal Complexes of Chiral Boron-Bridged Bisoxazoline (Borabox) Ligands. *Eur. J. Org. Chem.* **2008**, *27*, 4591-4597.

Purification and yield: Silica gel chromatography (25 mm column outer diameter, 18 cm silica bed height) was performed using isocratic elution (1 vol% acetone in CH_2Cl_2). The crude product was loaded onto the column in a minimum volume of CH_2Cl_2. The *anti*-aldol product was isolated as an off-white solid (226 mg, MW = 281.26 g/mol, 0.804 mmol, 80% yield).

TLC: *anti*-aldol product R_f = 0.34, *syn*-aldol product R_f = 0.22 (acetone/dichloromethane, 1:99).

99% *ee*: Chiralcel OD-H chiral HPLC column, iPrOH/n-hexane (5:95), flow rate = 1.0 mL/min, λ = 254 nm, injection volume = 20 µL, the sample was dissolved in 5 vol% iPrOH in n-hexane with a concentration of ≈ 1 mg/mL; *anti*-aldol product: t_{major} = 18.6 min, t_{minor} = 21.5 min. (Figures S62 & S63).

^{1}H NMR (400 MHz, $CDCl_3$) (ppm) *anti*-aldol product **3g** (Figure S61): δ 8.20 (d, 2H, J = 8.8 Hz), 7.58 (d, 2H, J = 8.6 Hz), 4.99 (dd, 1H, J = 7.9, 2.5 Hz), 4.28 (dd, 1H, J = 17.7, 1.4 Hz), 4.22 (dd, 1H, J = 7.9, 1.4 Hz), 4.08 (d, 1H, J = 17.7 Hz), 3.83 (d, 1H, J = 2.7 Hz), 1.38 (s, 3H), 1.20 (s, 3H).

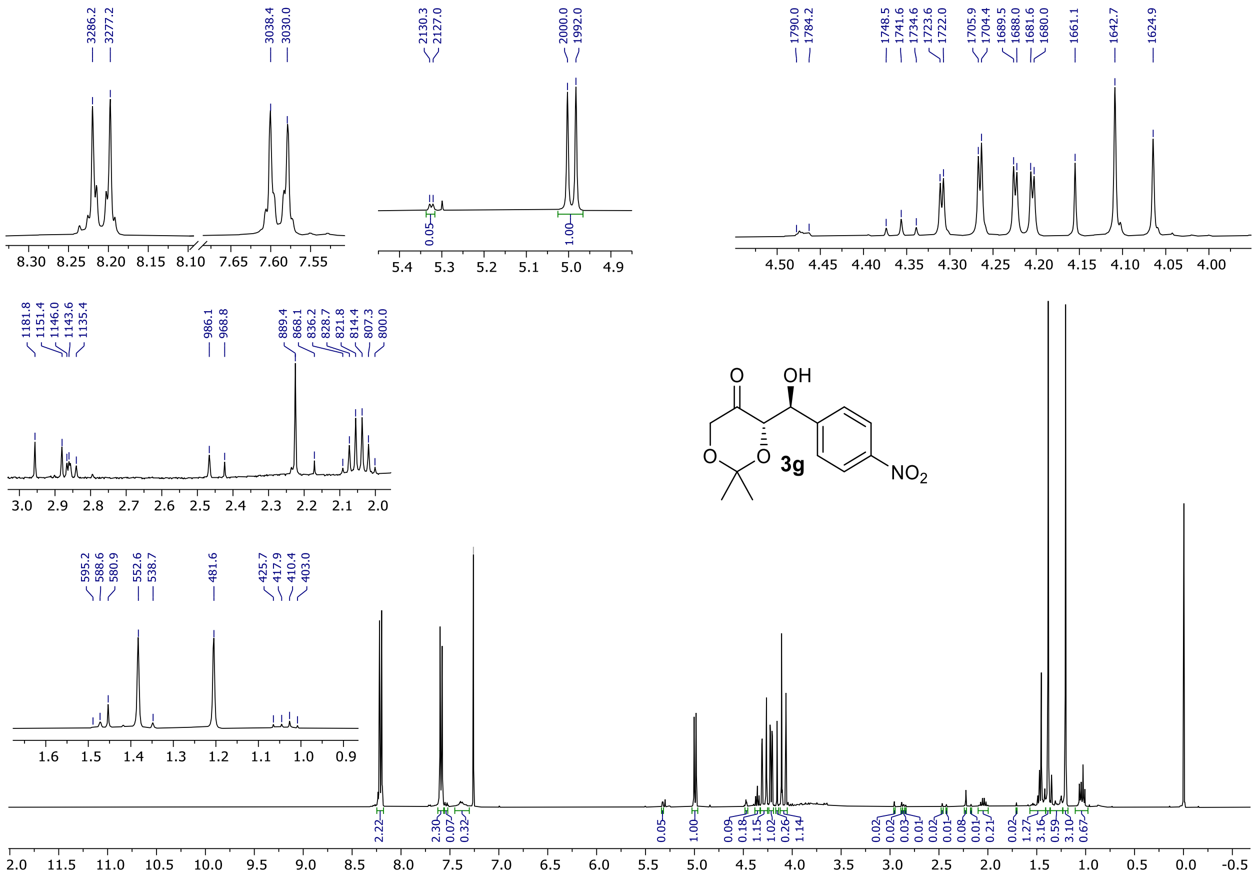

Figure S60. Crude ^{1}H NMR spectrum after high vacuum drying **3g** (above).

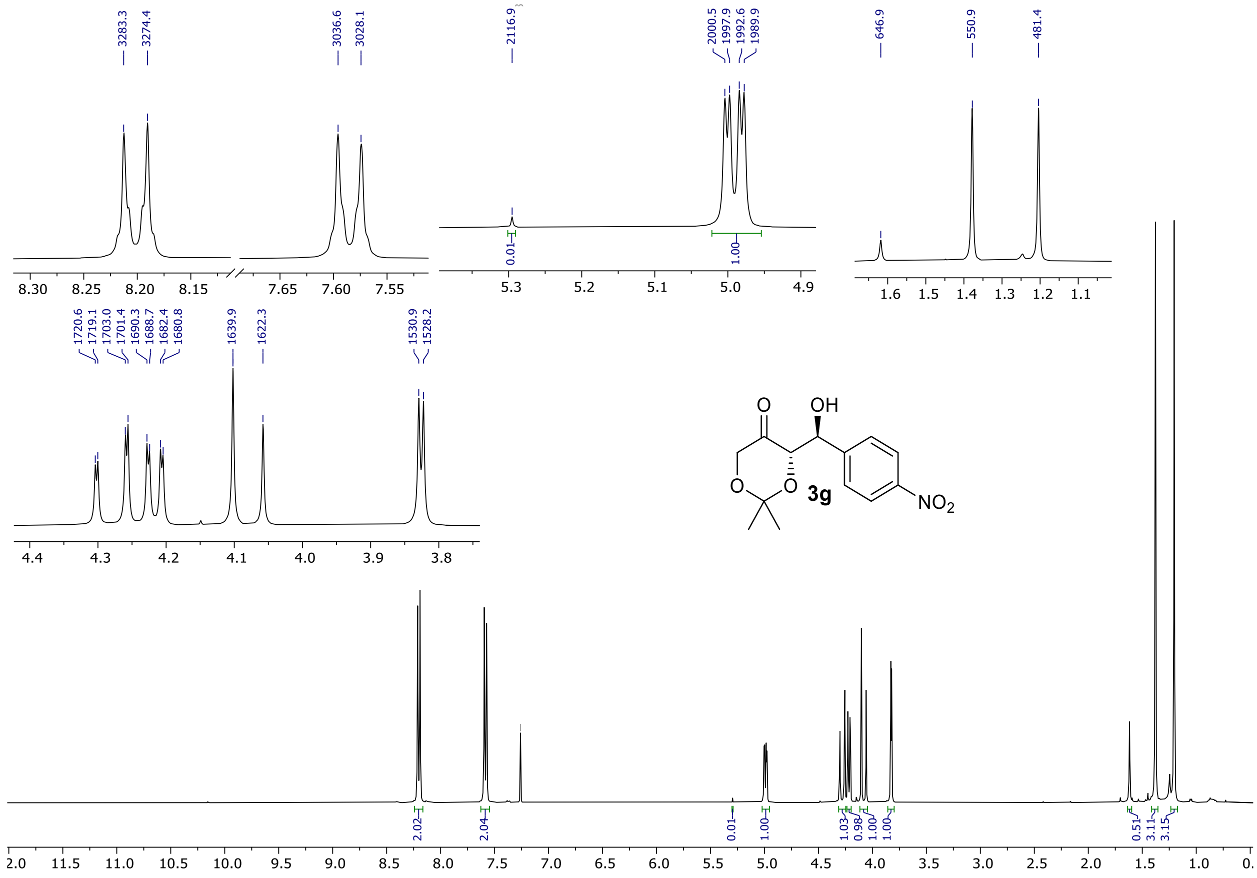

Figure S61. ^{1}H NMR spectrum of the purified *anti*-aldol (major) product **3g** (above).

SHIMADZU LabSolutions **Analysis Report**

<Sample Information>

Sample Name	: SSZ33-RACE 5%IPAnHex 1mLmin 1mgmL 10Aug23
Sample ID	:
Data Filename	: SSZ33-RACE 5%IPAnHex 1mLmin 1mgmL 20 uL 10Aug23.lcd
Method Filename	: trial.lcm
Batch Filename	:
Vial #	: 1-1
Sample Type	: Unknown
Injection Volume	: 20 uL
Date Acquired	: 8/10/2023 3:25:07 PM
Acquired by	: System Administrator
Date Processed	: 8/14/2023 4:35:17 PM
Processed by	: System Administrator

<Chromatogram>

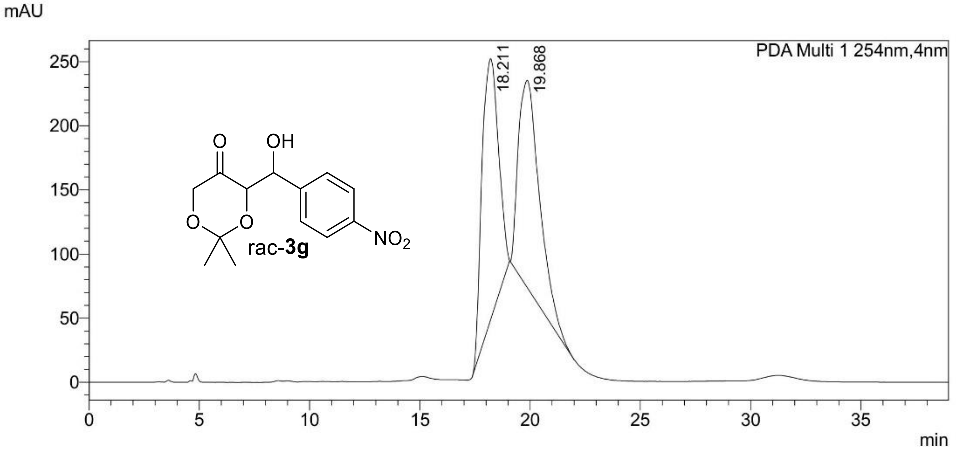

<Peak Table>

PDA Ch1 254nm

Peak#	Ret. Time	Area	Height	Area%
1	18.211	10783191	203257	50.099
2	19.868	10740403	161614	49.901
Total		21523593	364871	100.000

C:\Users\Shimadzu\Desktop\SZ\HPLC\Runs\SSZ33-RACE 5%IPAnHex 1mLmin 1mgmL 20 uL 10Aug23.lcd

Figure S62. HPLC chromatogram of the racemic *anti*-aldol product **3g** (above).

SHIMADZU LabSolutions

Analysis Report

<Sample Information>

Sample Name	: PK-015-5%IPANHEX-1mg/mL		
Data Filename	: PK-015-5%IPANHEX-1mg per mL.lcd		
Method Filename	: RL5-5%IPANHEX-1000PPM-SAM[5%IPANHEX].lcm		
Vial #	: 1-5	Sample Type	: Unknown
Injection Volume	: 20 uL		
Date Acquired	: 10/2/2024 1:15:48 PM	Acquired by	: System Administrator
Date Processed	: 10/2/2024 1:45:50 PM	Processed by	: System Administrator

<Chromatogram>

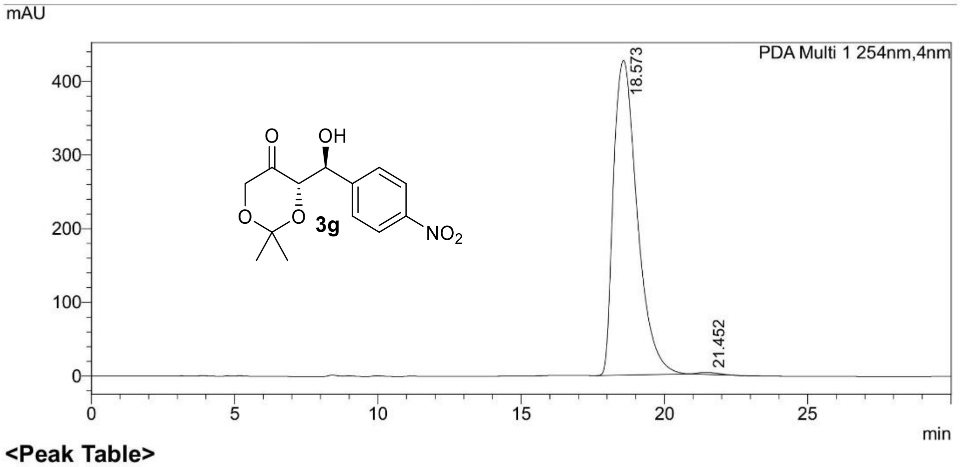

<Peak Table>

PDA Ch1 254nm

Peak#	Ret. Time	Area	Height	Area%
1	18.573	24603315	427006	99.442
2	21.452	137933	2676	0.558
Total		24741248	429682	100.000

C:\LabSolutions\Data\Project1\Data\Patrick\PK-015-5%IPANHEX-1mg per mL.lcd

Figure S63. HPLC chromatogram of the enantioenriched *anti*-aldol (major) product **3g** (above).

Table 2, entry 10: Competition reaction between cyclohexanone and methyl 4-nitrobuytrate for the limiting reactant 4-nitrobenzaldehyde

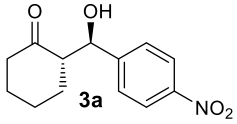

(*S*)-2-((*R*)-hydroxy(4-nitrophenyl)methyl)cyclohexan-1-one (**3a**):
To a clean, screw cap, V-shaped reaction vessel (5.0 mL) equipped with a small pyramidal stir bar, mortar and pestle ground 4-nitrobenzaldehyde (MW = 151.12 g/mol, 1.00 equiv, 1.00 mmol, 151 mg), cyclohexanone (MW = 98.15 g/mol, 1.50 equiv, 1.50 mmol, 147 mg, 155 µL, density 0.947 g/mL), and methyl 4-nitrobutyrate (MW = 147.13 g/mol, 1.50 equiv, 1.48 mmol, 218 mg, 190 µL, density 1.149 g/mL) were added. The liquid reactants (cyclohexanone and methyl 4-nitrobutyrate) were used to rinse the solid 4-nitrobenzaldehyde off the walls as needed. The mixture of compounds was gently stirred for 5 min resulting in an amber colored organic layer containing undissolved, finely divided aldehyde. The stirring was terminated and the *trans*-4-(*tert*-butyldiphenylsilyloxy)-L-proline catalyst (MW = 369.54 g/mol, 2.5 mol%, 0.025 mmol, 9.3 mg) was added to the reaction vessel. Within 30 sec after catalyst addition, distilled deoxygenated water (MW = 18.02 g/mol, 15.0 equiv, 15.0 mmol, 270 mg, 270 µL) was added with minimal disruption of the concentrated organic layer. The resulting biphasic system contained undissolved, finely divided solids in both phases which were both grey in color. This mixture was stirred such that the contents of the vessel did not splash against the walls and the phase boundary was gently agitated. After 16 h the stirring was stopped revealing a biphasic system with no undissolved solids. The bottom layer was semi-transparent and amber colored, while the upper layer was colorless. Work-up was performed at 16 h. See Section 2 for the work-up procedure.

This compound was previously synthesized and characterized, see below.

Crude product ^{1}H NMR analysis (Figure S64, see below) allowed determination of the diastereo- and chemoselectivity ratios.

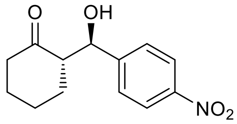

anti-aldol **3a** (major)

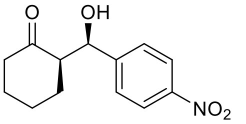

syn-aldol **4a** (minor)

Diastereoselectivity: The *anti-/syn*-aldol ratio was determined as >19:1 from the benzylic proton resonances at 4.89 ppm (d, *anti*-aldol product) and 5.48 ppm (bs, *syn*-aldol product) in the crude ^{1}H NMR spectrum (Figure S64). For the chemical shift literature values, see page S2 of the Supporting Information within: Mase, N.; Nakai, Y.; Ohara, N.; Yoda, H.; Takabe, K.; Tanaka, F.; Barbas, C. F., III. Organocatalytic Direct Asymmetric Aldol Reactions in Water. *J. Am. Chem. Soc.* **2006**, *128*, 734–735.

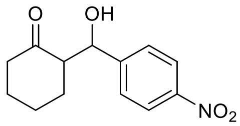

aldol products **3a**/**4a**

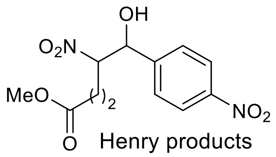

Henry products

Chemoselectivity: The *anti*- and *syn*-aldol/*anti*- and *syn*-Henry product ratio was determined to be >19:1, based on the resonance patterns for the *anti*-Henry product at 3.64 ppm (s, C$\underline{H}_3$OC(O)R) and *syn*-Henry product at 3.65 ppm (s, C$\underline{H}_3$OC(O)R) in our crude ^{1}H NMR spectrum (Figure S64). Alternative Henry product resonances were not chosen because the signal to noise ratio was not strong enough for accurate integration. For the Henry chemical shift literature values, see page S5 of the Supporting Information within: Blay, G.; Hernández-Olmos, V.; Pedro, J. R., XIII. Enantioselective Henry Addition of Methyl 4-Nitrobutyrate to Aldehydes. Chiral Building Blocks for 2-Pyrrolidinones and Other Derivatives. *Org. Lett.* **2010**, *12*, 3058-3061.

Purification and yield: Silica gel chromatography (25 mm column outer diameter, 18 cm silica bed height) was performed using isocratic elution (25 vol% EtOAc in petroleum ether). The crude product was loaded onto the column in a minimum volume of CH_2Cl_2. The *anti*- and *syn*-aldol product diastereomers were

isolated together as an off-white solid (214 mg, MW = 249.27 g/mol, 0.859 mmol, 86% yield). Note that chromatographic removal of the Henry byproduct was not possible, and the Henry product is labelled in the ^{1}H NMR spectrum of the purified product (see below).

TLC: *anti*-aldol product R_f = 0.27, *syn*-aldol product R_f = 0.33 (EtOAc/petroleum ether, 1:3).

99% *ee*: Chiralcel OD-H chiral HPLC column, iPrOH/n-hexane (7:93), flow rate = 1.0 mL/min, λ = 254 nm, injection volume = 20 μL, the sample was dissolved in 10 vol% iPrOH in n-hexane with a concentration of ≈ 1 mg/mL; *anti*-aldol product: t_{major} = 21.0 min, t_{minor} = 32.0 min. (Figure S66 & S67).

^{1}H NMR (400 MHz, $CDCl_3$) (ppm) *anti*-aldol product **3a** (Figure S65): δ 8.20 (d, 2H, J = 8.8 Hz), 7.50 (d, 2H, J = 8.5 Hz), 4.89 (dd, 1H, J = 8.3, 3.1 Hz), 4.08 (d, 1H, J = 3.2), 2.54-2.63 (m, 1H), 2.45-2.53 (m, 1H), 2.31-2.41 (m, 1H), 2.07-2.15 (m, 1H), 1.78-1.86 (m, 1H), 1.49-1.73 (m, 3H), 1.31-1.44 (m, 1H).

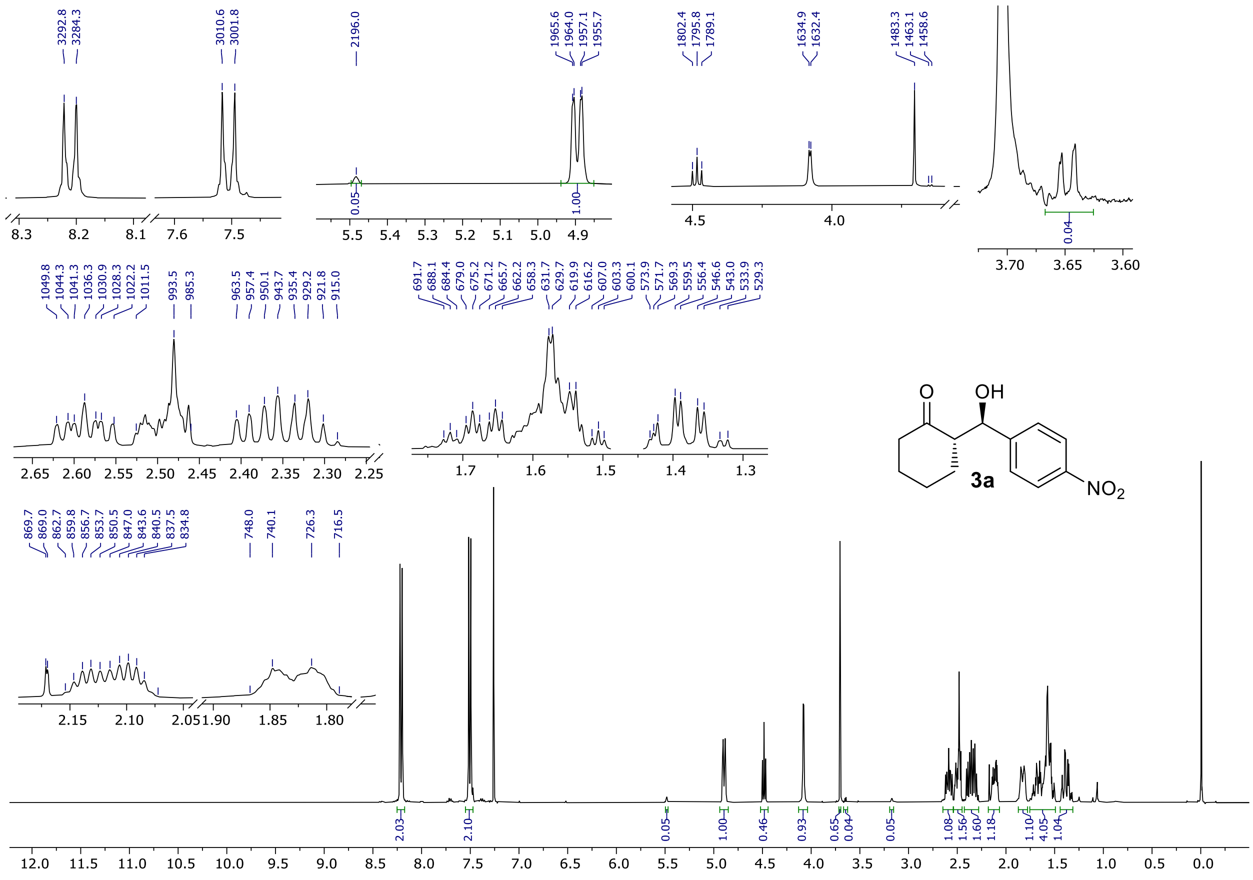

Figure S64. Crude ^{1}H NMR spectrum after high vacuum drying **3a** (above).

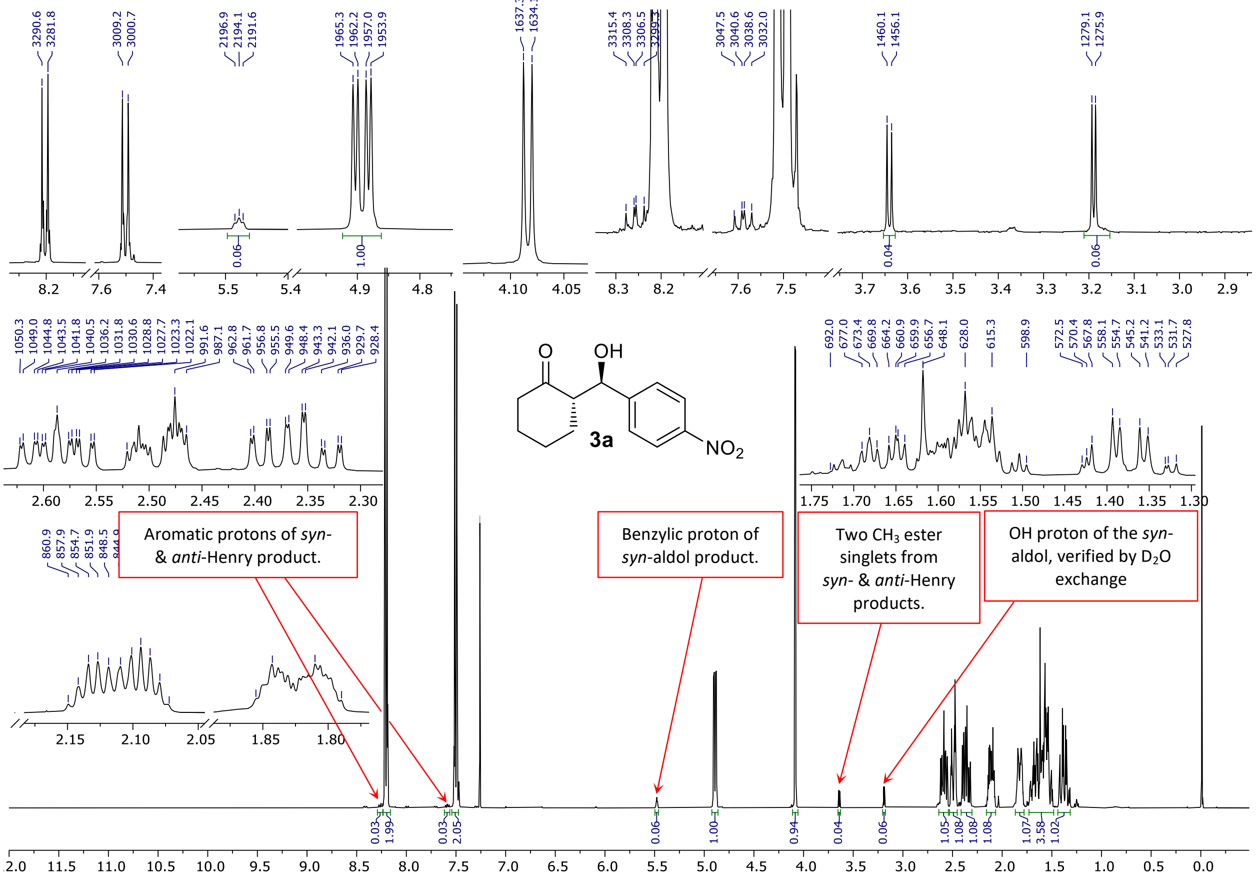

Figure S65. ^{1}H NMR spectrum of the purified *anti*-(major) **3a** and *syn*-(minor) **4a** aldol products. The red arrows indicate the *syn*-aldol product.

Epimerization of aldol product 3a: Aldol product diastereomers of the structural category produced in this study are prone to epimerization on heating or on exposure to silica gel. Consequently, crude aldol product ^{1}H NMRs can show higher *dr* values than ^{1}H NMRs of the chromatographed aldol products. This, in large part, is why most researchers in this area decided long ago to evaluate the aldol diastereoselectivity using crude ^{1}H NMR spectrums. Furthermore, most researchers isolate the *anti*-/*syn*-aldol products together and report the total yield. For this study, we decided to isolate the *anti*-aldol products and record their yield, and that was possible for all products except *anti*-aldol product **3a**. The *anti*-aldol product **3a** epimerizes during chromatography to an extent that prohibits its isolation free of the *syn*-aldol product **4a**. For related matters, see page S4 within the Supporting Information of: Nugent, T.C.; Umar, M. N.; Bibi, A. Picolylamine as an Organocatalyst Template for Highly Diastereo- and Enantioselective Aqueous Aldol Reactions. *Org. Biomol. Chem.* **2010**, *8*, 4085-4089.

SHIMADZU LabSolutions

Analysis Report

ADV-A45-7% IPA/nHex 5uL 1mL/min 12May2022

Sample Name	: A46 (RACE)7% IPAnHex 5uL1mLmin 26May 2		
Sample ID	:		
Data Filename	: A46 (RACE)7% IPAnHex 5uL1mLmin 26May 2.lcd		
Method Filename	: trial.lcm		
Batch Filename	:		
Vial #	: 1-1	Sample Type	: Unknown
Injection Volume	: 20 uL		
Date Acquired	: 5/26/2022 2:38:31 PM	Acquired by	: System Administrator
Date Processed	: 5/26/2022 3:28:36 PM	Processed by	: System Administrator

ADV-A46-7% IPA/nHex 5uL 1mL/min 12May2022

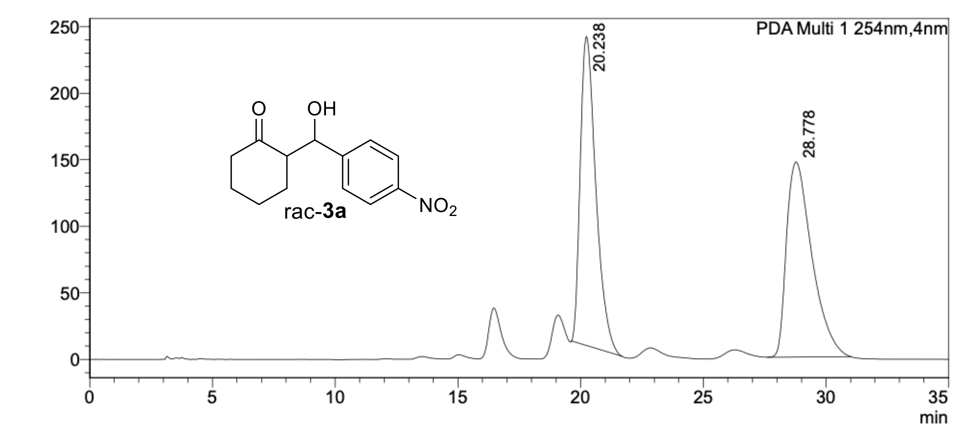

ADV-A45-7% IPA/nHex 5uL 1mL/min 12May2022

PDA Ch1 254nm

Peak#	Ret. Time	Area	Height	Area%
1	20.238	10283216	232276	49.316
2	28.778	10568658	146536	50.684
Total		20851874	378812	100.000

C:\Users\Shimadzu\Desktop\ADV\Competition aldol samples\A46 (RACE)7% IPAnHex 5uL1mLmin 26May 2.lcd

Figure S66. HPLC chromatogram of the racemic *anti*-aldol product **3a** (above).

SHIMADZU LabSolutions **Analysis Report**

<Sample Information>

Sample Name	: PK-I-18 (PK-013)		
Data Filename	: PK-I-18 (PK-013).lcd		
Method Filename	: RL1-7%IPANHEX-1000PPM-SAM[10%IPANHEX].lcm		
Vial #	: 1-5	Sample Type	: Unknown
Injection Volume	: 20 uL		
Date Acquired	: 9/19/2024 5:06:26 PM	Acquired by	: System Administrator
Date Processed	: 9/19/2024 5:46:28 PM	Processed by	: System Administrator

<Chromatogram>

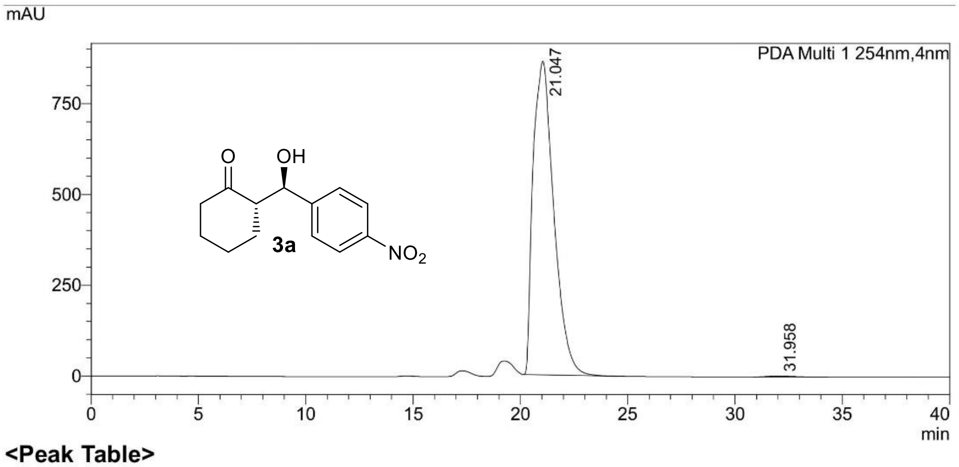

<Peak Table>

PDA Ch1 254nm

Peak#	Ret. Time	Area	Height	Area%
1	21.047	57979985	864098	99.697
2	31.958	176264	2021	0.303
Total		58156249	866120	100.000

C:\Users\Shimadzu\Desktop\Patrick\PK-I-18 (PK-013).lcd

Figure S67. HPLC chromatogram of the enantioenriched *anti*-aldol (major) product **3a** (above).

Section 8. Table 3 entries 1-7, 10, 11 experimental descriptions and characterization of *anti*-aldol product **3a**, **e**, **h**

Characterization: All aldol products formed in this study have been previously characterized and often by many different researchers. The references we cite here are ones containing experimental data including an HPLC trace and often a ^{1}H NMR spectrum.

Table 3, entry 1: Competition reaction between cyclohexanone and 4-(tert-butyl)-1-(nitromethyl)cyclohex-1-ene (2a) for the limiting reactant 3-chlorobenzaldehyde

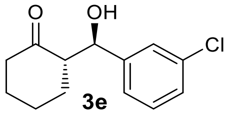

(*S*)-2-((*R*)-(3-chlorophenyl)(hydroxy)methyl)-cyclohexan-1-one (**3e**):
To a clean, screw cap, V-shaped reaction vessel (2.0 mL) equipped with a small pyramidal stir bar were added: 4-(tert-butyl)-1-(nitromethyl)cyclohex-1-ene (**2a**) (MW = 197.28 g/mol, 1.50 equiv, 1.05 mmol, 207.1 mg), cyclohexanone (MW = 98.15 g/mol, 1.50 equiv, 1.05 mmol, 103 mg, 109 µL, density = 0.947 g/mL) and freshly purified (see Section 2) 3-chlorobenzaldehyde (MW = 140.57 g/mol, 1.00 equiv, 0.70 mmol, 98 mg, 79 µL, density = 1.241 g/mL). This mixture was gently stirred for 5 min resulting in a clear colorless solution with no undissolved material. The stirring was terminated and *trans*-4-(*tert*-butyldiphenylsilyloxy)-L-proline (MW = 369.54 g/mol, 2.5 mol%, 0.0175 mmol, 6.47 mg) was added to the reaction vessel resulting in a fully dissolved colorless solution. Distilled deoxygenated water (MW = 18.02 g/mol, 15.00 equiv, 10.5 mmol, 189 mg, 189 µL) was added with minimal disruption of the concentrated organic layer. The mixture was stirred such that the contents of the vessel did not splash up and against the walls, but the phase boundary was gently agitated. Note that the concentrated organic layer became milky when the water was added and remained so until about 2 h at which point the concentrated organic layer became transparent with no undissolved solids. The reaction remained this way until work-up (32 h). See Section 2 for the work-up procedure.

This compound was previously synthesized and characterized, see below.

Crude product ^{1}H NMR analysis (Figure S68, see below) allowed determination of the following diastereo- and chemoselectivity ratios.

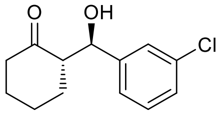

anti-aldol (major) **3e**

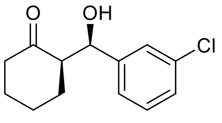

syn-aldol (minor) **4e**

Diastereoselectivity: The *anti*-aldol/*syn*-aldol ratio was determined as 12.5:1 from the benzylic proton resonances at 4.76 ppm (d, *anti*-aldol product) and 5.37 ppm (d, *syn*-aldol product) in the crude ^{1}H NMR spectrum (Figure S68). For the chemical shift literature values, see page SI 6 (product 3f) of the Supporting Information within: Martínez-Castañeda, Á.; Rodríguez-Solla, H.; Concellón, C.; del Amo, V. TBD/Al_2O_3: A Novel Catalytic System for Dynamic Intermolecular Aldol Reactions that Exhibit Complex System Behaviour. *Org. Biomol. Chem.* **2012**, *10*, 1976-1981.

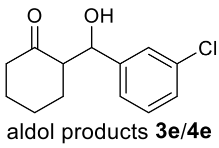

aldol products **3e**/**4e**

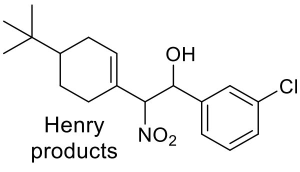

Henry products

Chemoselectivity: The *anti*- and *syn*-aldol/*anti*- and *syn*-Henry product ratio is >19:1. This Henry product is not reported in the literature, and we have not synthesized it. However, based on related Henry products we have synthesized (see Section 5 of this document), no resonances corresponding to the indicative chemical shifts, -C**H**(NO_2)-C**H**(OH)Ar, of the potential Henry products were observed in the crude ^{1}H NMR (Figure S68) of this competition reaction.

Purification and yield: The crude product was loaded onto a silica gel (230-400 mesh) column (16 cm in height, 2.5 cm in diameter pre-wetted with EtOAc/petroleum ether (1:19). The crude product was loaded onto the column in a minimum volume of EtOAc. The mobile phase elution began with EtOAc/petroleum ether (1:19). This solvent ratio was maintained until the starting materials were removed from the column. The polarity of the mobile phase was then raised to EtOAc/petroleum ether (1:13) to remove the *syn*-aldol product. The *anti*-aldol product eluted soon after use of EtOAc/petroleum ether (1:9). Concentration of the pure fractions provided a white solid (MW= 238.71 g/mol, 140 mg, 0.586 mmol, 84% yield) of the *anti*-aldol product **3e**.

R_f: 0.17 (*anti*-aldol product), EtOAc/petroleum ether (15:85). Note: UV at 254 nm was ineffective for observation of product, instead CAM stain was effective.

97% *ee*: Chiralcel OD-H chiral HPLC column, iPrOH/n-hexane (5:95), flow rate = 1.0 mL/min, λ = 210 nm, injection volume = 6 μL, the sample was dissolved in 5 vol% *i*PrOH/n-hexane with a concentration of ≈1 mg/mL; *anti*-aldol product retention times: t_{minor}= 15.6, t_{major}= 11.1 (Figures S70 and S71).

^{1}H NMR (400 MHz, $CDCl_3$) (ppm) *anti*-aldol product **3e** (Figure S69): δ 7.35-7.32 (m, 1H), 7.30-7.23 (m, 2H), 7.22-7.16 (m, 1H), 4.75 (dd, 1H, J = 8.8, 2.9 Hz), 4.01 (d, 1H, J = 2.9 Hz), 2.63-2.53 (m, 1H), 2.52-2.44 (m, 1H), 2.41-2.30 (m, 1H), 2.15-2.06 (m, 1H), 1.86-1.76 (m, 1H), 1.73-1.49 (m, 4H), 1.31 (m, 1H).

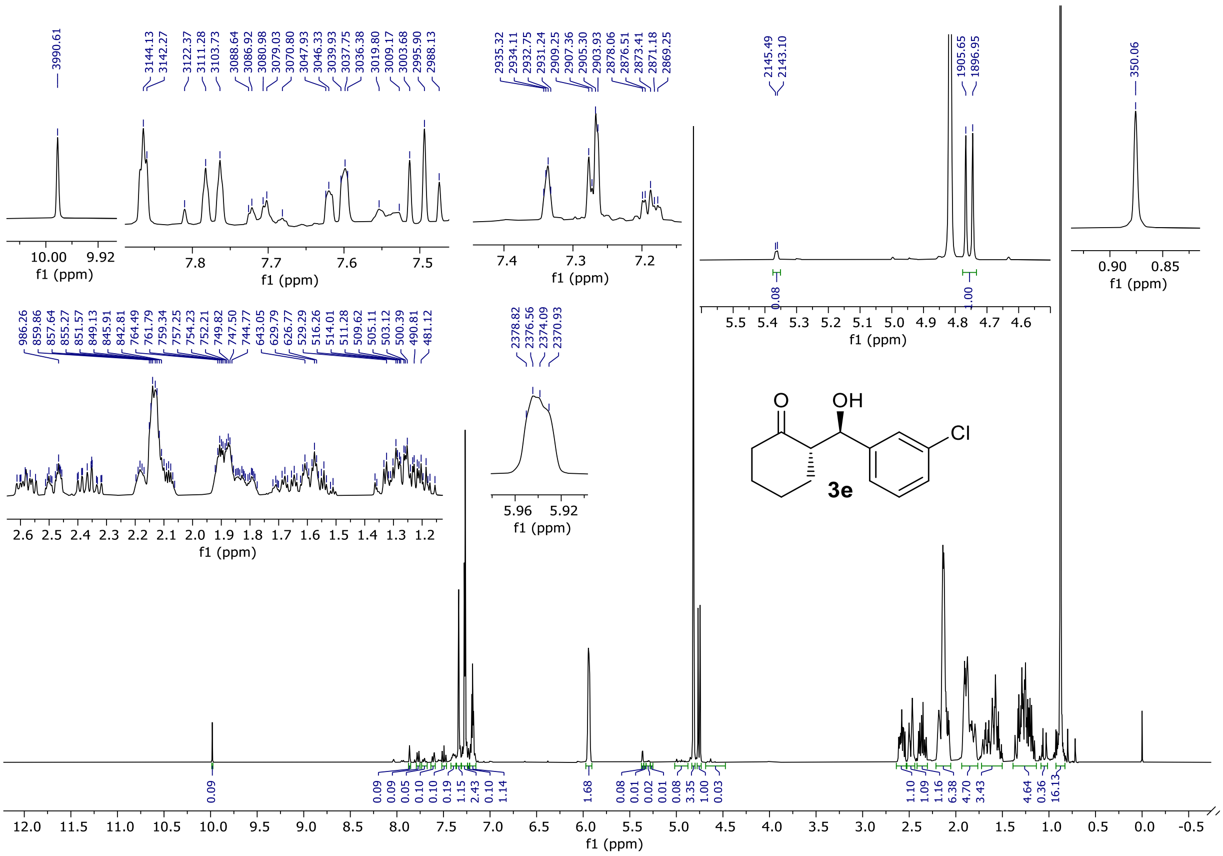

Figure S68. Crude ^{1}H NMR spectrum after high vacuum drying of **3e** (above).

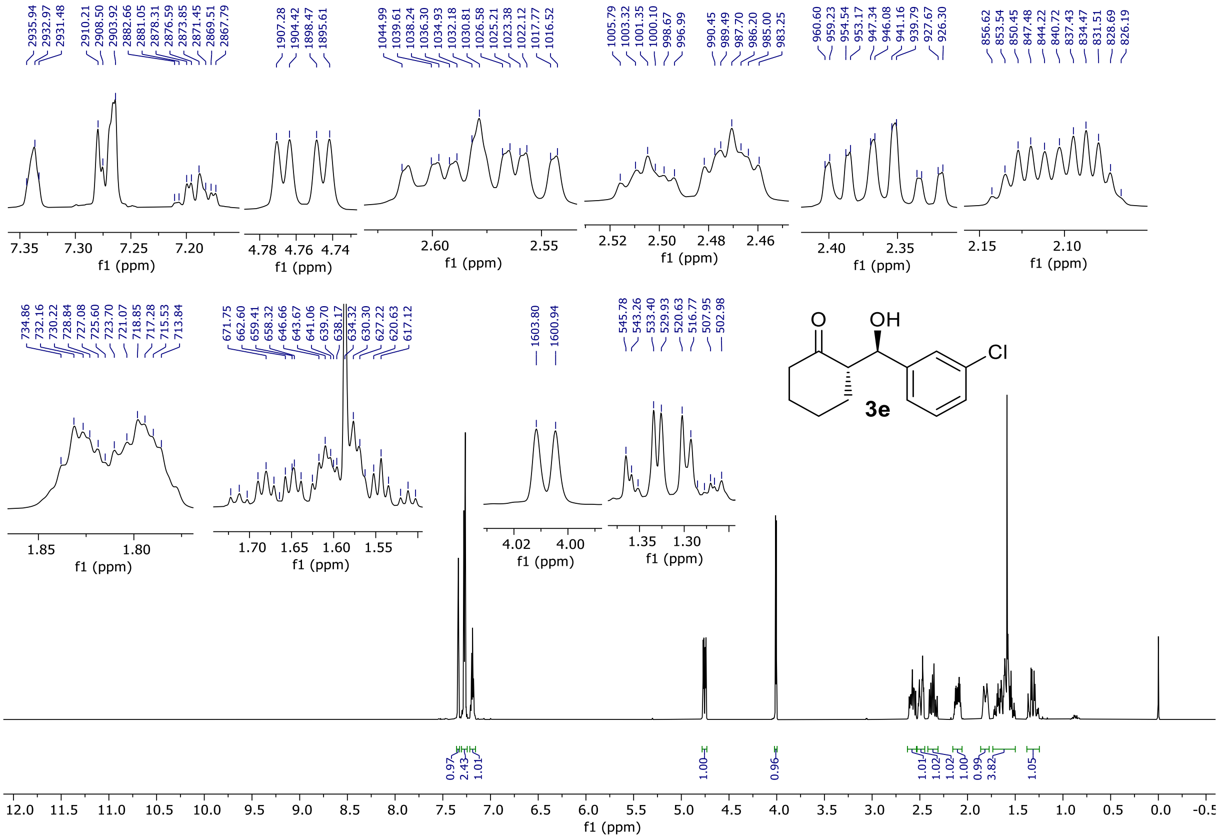

Figure S69. ^{1}H NMR spectrum of purified *anti*-aldol (major) product **3e** (above).

```
Acq. Operator   :                                    Seq. Line :    2
Acq. Instrument : Instrument 1                        Location : P1-A-03
Injection Date  : 2/6/2025 1:35:39 PM                      Inj :    1
                                                    Inj Volume : 20 µl
Acq. Method     : D:\Methods\Nugentlab_Patrick\AIDA.m
Last changed    : 2/6/2025 1:34:31 PM
Analysis Method : D:\Methods\Nugentlab_Patrick\AIDA254NM1.m
Last changed    : 2/6/2025 2:01:03 PM
                  (modified after loading)
```

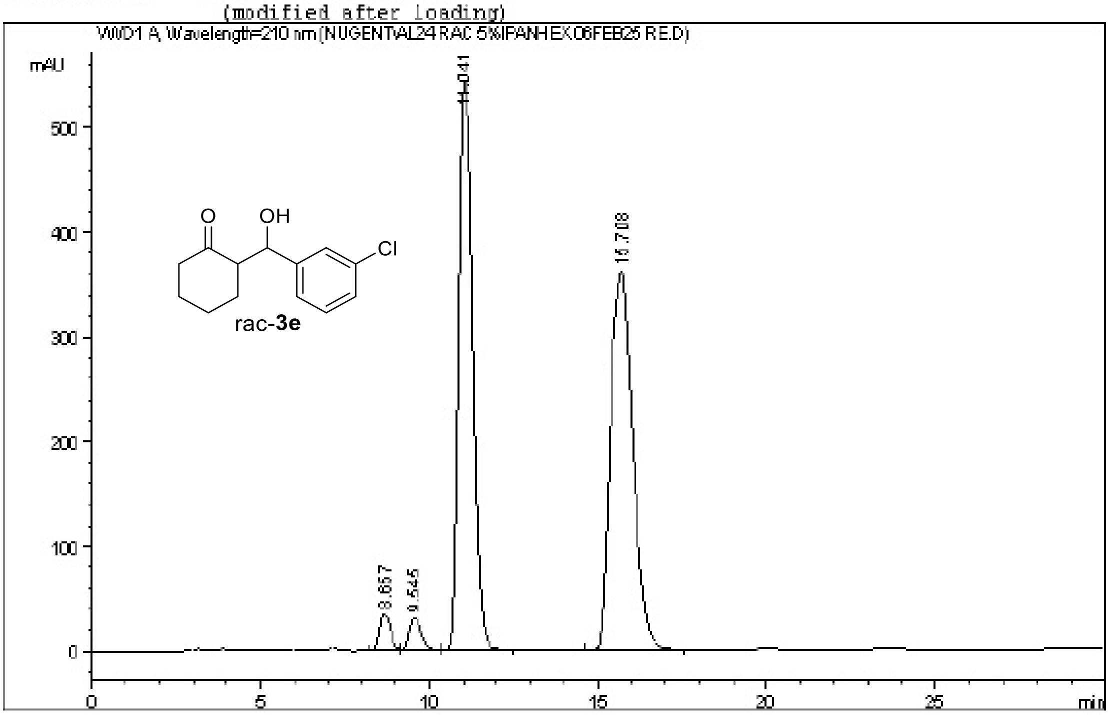

===

Area Percent Report

===

```
Sorted By             :      Signal
Multiplier            :      1.0000
Dilution              :      1.0000
Use Multiplier & Dilution Factor with ISTDs
```

Signal 1: VWD1 A, Wavelength=210 nm

Peak #	RetTime [min]	Type	Width [min]	Area mAU *s	Height [mAU]	Area %
1	8.657	BV	0.3643	785.40881	34.54132	2.2828
2	9.545	VB	0.4170	804.99719	30.80102	2.3397
3	11.041	BB	0.4747	1.62915e4	543.91724	47.3515
4	15.708	BB	0.7207	1.65235e4	361.77737	48.0260
Totals :				3.44054e4	971.03695	

Figure S70. HPLC chromatogram of the racemic *anti*-aldol product **3e** (above).

Data File D:\DATA\NUGENT\KD-II-77 ANTI 5%IPAINHEX 17MAR25.D
Sample Name: KD-II-77 ANTI 5%IPAINHEX 17MAR25

===

```
Acq. Operator   :                                Seq. Line :   1
Acq. Instrument : Instrument 1                    Location : P1-A-01
Injection Date  : 3/17/2025 11:49:33 AM                Inj :   1
                                                Inj Volume : 20 µl
Different Inj Volume from Sequence !     Actual Inj Volume : 6 µl
Acq. Method     : D:\Methods\Nugentlab Patrick\AIDA 5IPA 210NM.m
Last changed    : 3/17/2025 11:48:30 AM
Analysis Method : D:\Methods\Nugentlab_Patrick\AIDA 15IPA 254NM.m
Last changed    : 3/3/2025 4:17:17 PM
                  (modified after loading)
```

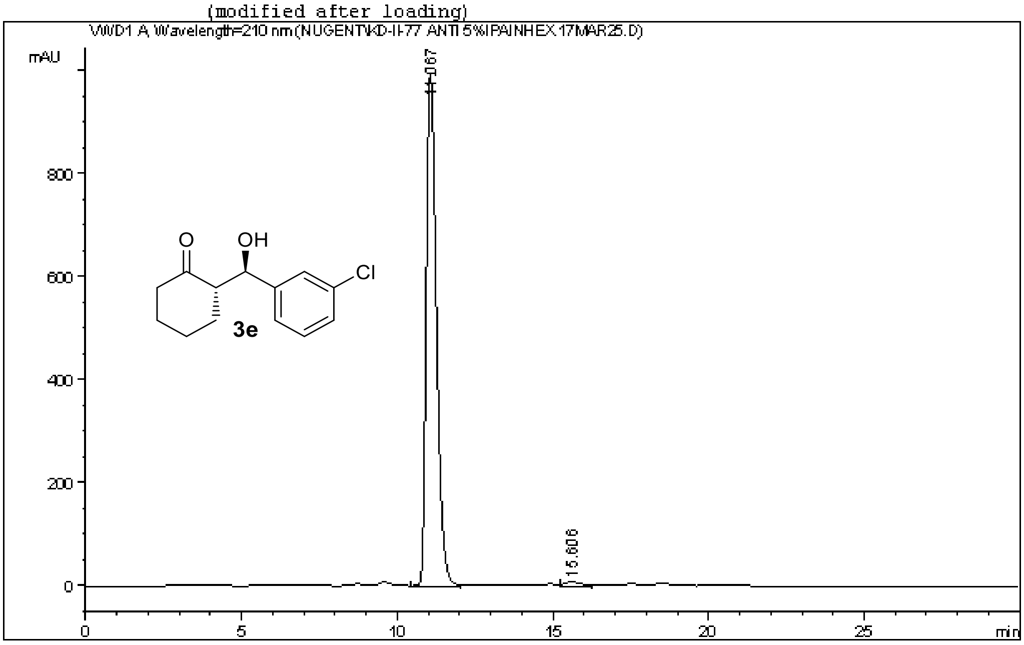

===
Area Percent Report
===

```
Sorted By             :      Signal
Multiplier            :      1.0000
Dilution              :      1.0000
Use Multiplier & Dilution Factor with ISTDs
```

Signal 1: VWD1 A, Wavelength=210 nm

Peak #	RetTime [min]	Type	Width [min]	Area mAU *s	Height [mAU]	Area %
1	11.067	VV	0.3624	2.29981e4	996.64447	98.7271
2	15.606	VV	0.5508	296.51785	8.18525	1.2729

Totals : 2.32946e4 1004.82972

===
*** End of Report ***

Instrument 1 3/17/2025 12:36:20 PM   Page 1 of 1

Figure S71. HPLC chromatogram of enantioenriched *anti*-aldol (major) product **3e** (above).

Table 3, entry 2: Competition reaction between cyclohexanone and 4-(tert-butyl)-1-(nitromethyl)cyclohex-1-ene (2a) for the limiting reactant 4-(trifluoromethyl)benzaldehyde

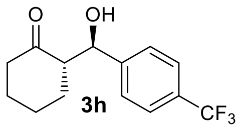

(*S*)-2-((*R*)-hydroxy(4-(trifluoromethyl)phenyl)methyl)-cyclohexan-1-one (**3h**): To a clean, screw cap, V-shaped reaction vessel (2.0 mL) equipped with a small pyramidal stir bar, 4-(tert-butyl)-1-(nitromethyl)cyclohex-1-ene (**2a**) (MW = 197.28 g/mol, 1.50 equiv, 1.05 mmol, 207.1 mg), cyclohexanone (MW = 98.15 g/mol, 1.50 equiv, 1.05 mmol, 103.1 mg, 109 µL, density 0.947 g/mL) and freshly purified (see Section 2) 4-(trifluoromethyl)benzaldehyde (MW = 174.12 g/mol, 1.00 equiv, 0.70 mmol, 122 mg, 96 µL, density= 1.275 g/mL) were added. This mixture was gently stirred for 5 min resulting in a clear colorless solution with no undissolved material. The stirring was terminated and *trans*-4-(*tert*-butyldiphenylsilyloxy)-L-proline (MW = 369.54 g/mol, 2.5 mol%, 0.0175 mmol, 6.47 mg) was added resulting in a clear colorless fully dissolved solution. Distilled deoxygenated water (MW = 18.02 g/mol, 15.00 equiv, 10.5 mmol, 189 mg, 189 µL) was added to the reaction vessel with minimal disruption of the concentrated organic layer. The mixture was stirred such that the contents of the vessel did not splash up and against the walls, but the phase boundary was gently agitated. Note that the concentrated organic layer became milky when the water was added and remained so until about 2 h at which point the concentrated organic layer became transparent with no undissolved solids. The reaction remained this way until work-up (32 h). See Section 2 for the work-up procedure.

This compound was previously synthesized and characterized, see below.

Crude product ^{1}H NMR analysis (Figure S72, see below) allowed determination of the following diastereo- and chemoselectivity ratios.

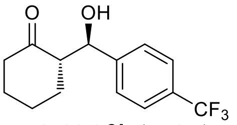

anti-aldol **3h** (major)

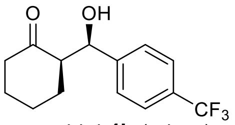

syn-aldol **4h** (minor)

Diastereoselectivity: The *anti*-aldol/*syn*-aldol product ratio was determined as 14:1 from the benzylic proton resonances at 4.85 ppm (d, *anti*-aldol product) and 5.44 ppm (s_{broad}, *syn*-aldol product) in the crude ^{1}H NMR spectrum (Figure S72). For literature regarding the *syn*-aldol product chemical shift, see page 14 of the Supporting Information within: Gao, J.; Bai, S.; Gao, Q.; Liu, Y.; Yang, Q. Acid Controlled Diastereoselectivity in Asymmetric Aldol Reaction of Cycloketones with Aldehydes using Enamine-Based Organocatalysts. *Chem. Commun.* **2011**, *47*, 6716–6718. For the chemical shift literature value for the *anti*-aldol product, see page S8 of the supporting information of: Al Beiruty, H.; Zhylinska, S.-S.; Kutateladze, N.; Cheong, H. K. T.; Ñíguez, J. A.; Burlingham, S. J.; Marset X.; Guillena, G.; Chinchilla, R.; Alonso, D. A.; Nugent, T. C. Enantioselective Catalytic Aldol Reactions in the Presence of Knoevenagel Nucleophiles: A Chemoselective Switch Optimized in Deep Eutectic Solvents Using Mechanochemistry. *Molecules* **2024**, *29*, 4. https://doi.org/10.3390/molecules29010004

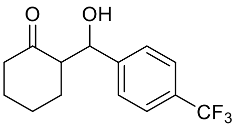

aldol products **3h**/**4h**

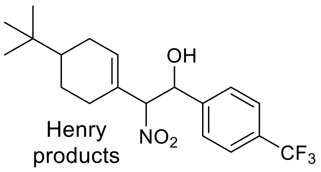

Chemoselectivity: The *anti*- and *syn*-aldol/*anti*- and *syn*-Henry product ratio is >19:1. This Henry product is not reported in the literature, and we have not synthesized it. However, based on related Henry products we have synthesized (see Section 5 of this document), no resonances corresponding to the indicative chemical shifts, -C**H**(NO_2)-C**H**(OH)Ar, of the potential Henry products were observed in the crude ^{1}H NMR (Figure S72) of this competition reaction.

Purification and yield: The crude product was loaded onto a silica gel (230-400 mesh) column (16 cm in height, 2.5 cm in diameter) pre-wetted with EtOAc/petroleum ether (1:9). The crude product was loaded onto the column in a minimum volume of EtOAc. The mobile phase elution began with EtOAc/petroleum ether (1:9). This solvent ratio was maintained until the starting materials were removed from the column. The polarity of the mobile phase was raised to EtOAc/petroleum ether (1:7) to remove the *syn*-aldol product. The *anti*-aldol product eluted soon after use of EtOAc/petroleum ether (1:5.7). Concentration of the pure fractions provided white solid (MW= 272.27 g/mol, 156.3 mg, 0.574 mmol, 82% yield) of the *anti*-aldol product **3h**.

R_f: 0.22 (*anti*-aldol product), EtOAc/petroleum ether (25:75) Note: UV at 254 nm was ineffective for observation of product, instead CAM stain was effective.

98% *ee*: Chiralcel OD-H chiral HPLC column, iPrOH/n-hexane (20:80), flow rate = 0.5 mL/min, λ = 216 nm, injection volume = 20 µL, the sample was dissolved in 20 vol% iPrOH/n-hexane with a concentration of ≈ 1 mg/mL; *anti*-aldol product retention times: t_{major} = 10.8 min, t_{minor} = 12.5 min. (Figures S74 and S75).

^{1}H NMR (400 MHz, $CDCl_3$) (ppm) *anti*-aldol product **3h** (Figure S73): δ 7.61 (d, 2H, J = 8.1 Hz), 7.44 (d, 2H, J = 8.0 Hz), 4.84 (dd, 1H, J = 8.6, 2.9 Hz), 4.04 (d, 1H, *J* = 3.0 Hz), 2.64-2.54 (m, 1H), 2.53-2.44 (m, 1H), 2.42-2.30 (m, 1H), 2.15-2.05 (m, 1H), 1.88-1.77 (m, 1H), 1.74-1.48 (m, 3H), 1.40-1.20 (m, 1H).

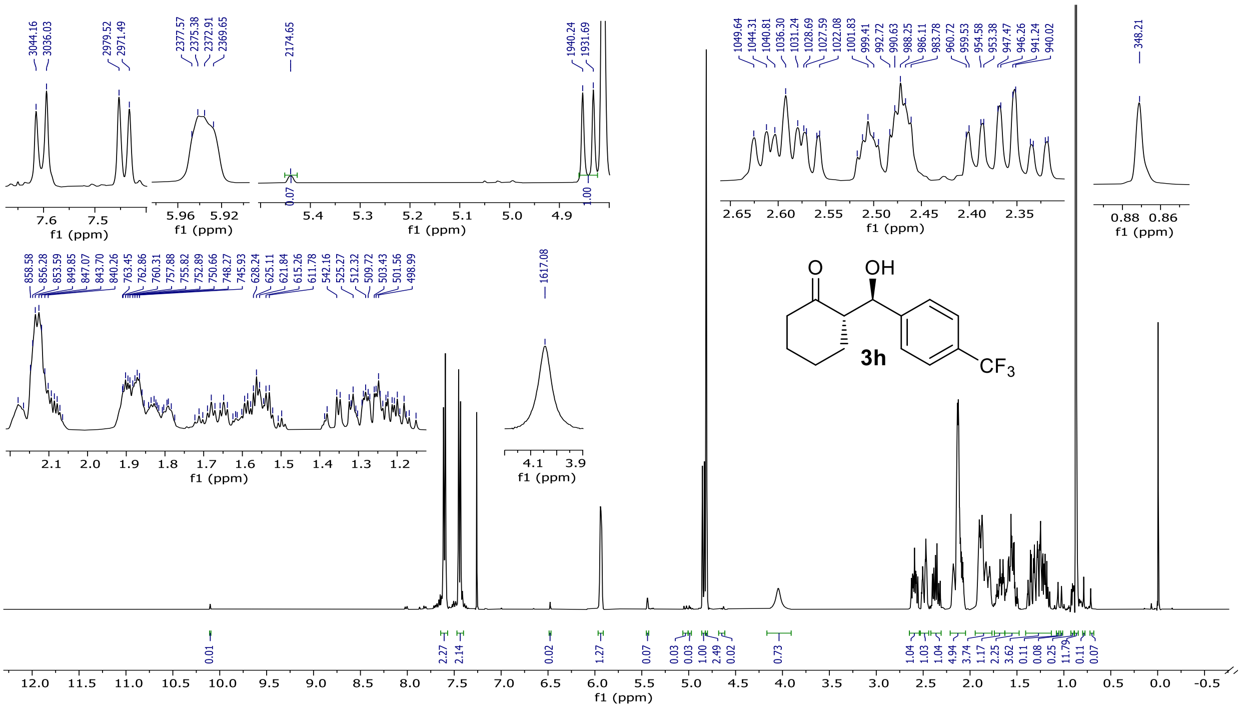

Figure S72. Crude ^{1}H NMR spectrum after high vacuum drying of **3h** (above).

Note that ^{1}H NMR data of crude product **3h**, after work-up and drying, of the reaction between cyclohexanone and 4-trifluoromethylbenzaldehyde was problematic. Each sample from the semi-solid product provided a different chemoselectivity. For that reason, the entire crude product was dissolved in $CDCl_3$ and only then did we record the ^{1}H NMR.

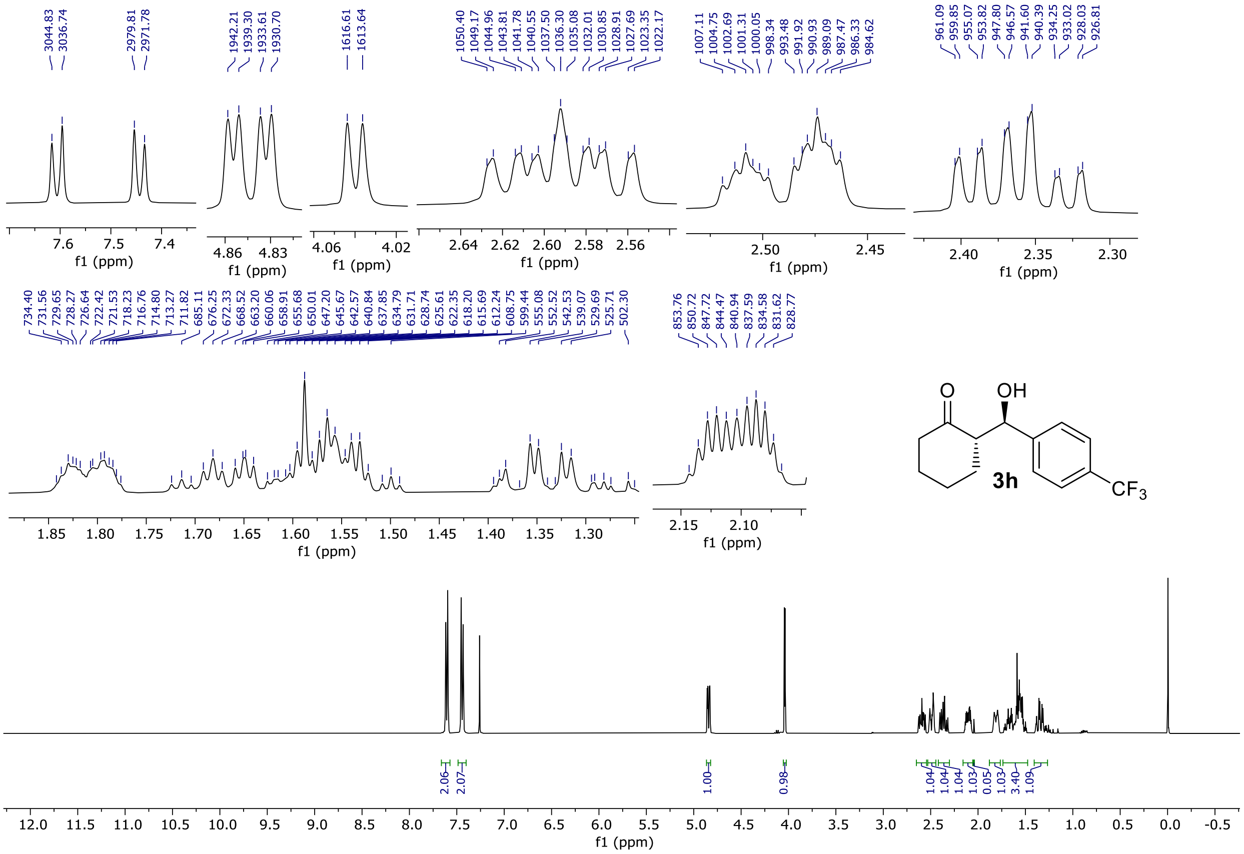

Figure S73. ^{1}H NMR spectrum of purified *anti*-aldol (major) product **3h** (above).

SHIMADZU LabSolutions **Analysis Report**

<Sample Information>

Sample Name	: HC-I-26 20%IPAnHex 20uL 0.5 mLmin 08Feb23 1mgmL		
Sample ID	:		
Data Filename	: HC-I-26 20%IPAnHex 20uL 0.5 mLmin 08Feb23 1mgmL.lcd		
Method Filename	: trial.lcm		
Batch Filename	:		
Vial #	: 1-4	Sample Type	: Unknown
Injection Volume	: 20 uL		
Date Acquired	: 2/8/2023 3:15:20 PM	Acquired by	: System Administrator
Date Processed	: 2/8/2023 3:39:35 PM	Processed by	: System Administrator

<Chromatogram>

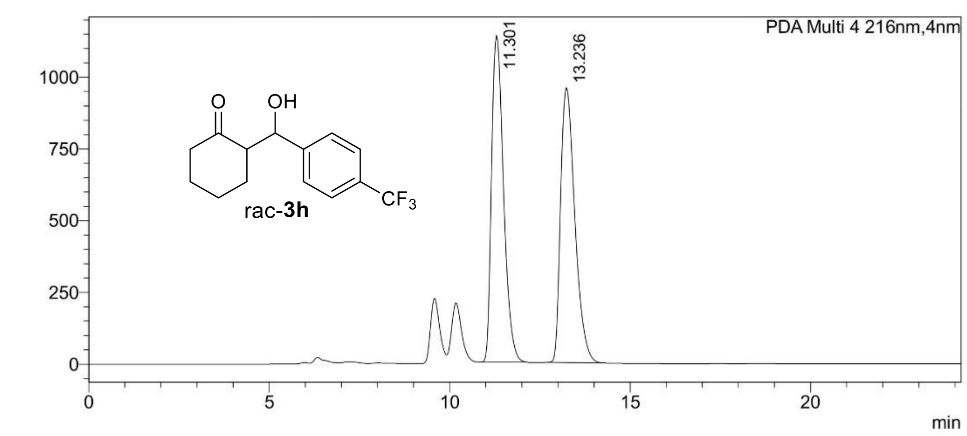

<Peak Table>

PDA Ch4 216nm

Peak#	Ret. Time	Area	Height	Area%
1	11.301	26277677	1138787	49.940
2	13.236	26341028	958736	50.060
Total		52618705	2097523	100.000

C:\Users\Shimadzu\Desktop\Hayley\HC-I-26 20%IPAnHex 20uL 0.5 mLmin 08Feb23 1mgmL.lcd

Figure S74. HPLC chromatogram of the racemic *anti*-aldol product **3h** (above).

Data File D:\DATA\NUGENT\KD-II-78 ANTI 20% IPAINHEX RERUN 18MAR25.D
Sample Name: KD-II-78 ANTI 20% IPAINHEX RERUN 18MAR25

===

Acq. Operator : Seq. Line : 1
Acq. Instrument : Instrument 1 Location : P1-A-03
Injection Date : 3/18/2025 1:11:34 PM Inj : 1
Inj Volume : 20 µl
Acq. Method : D:\Methods\Nugentlab_Patrick\PK 20IPA 216NM.m
Last changed : 3/18/2025 1:09:54 PM
Analysis Method : D:\Methods\Nugentlab_Patrick\AIDA 15IPA 254NM.m
Last changed : 3/3/2025 4:17:17 PM
(modified after loading)

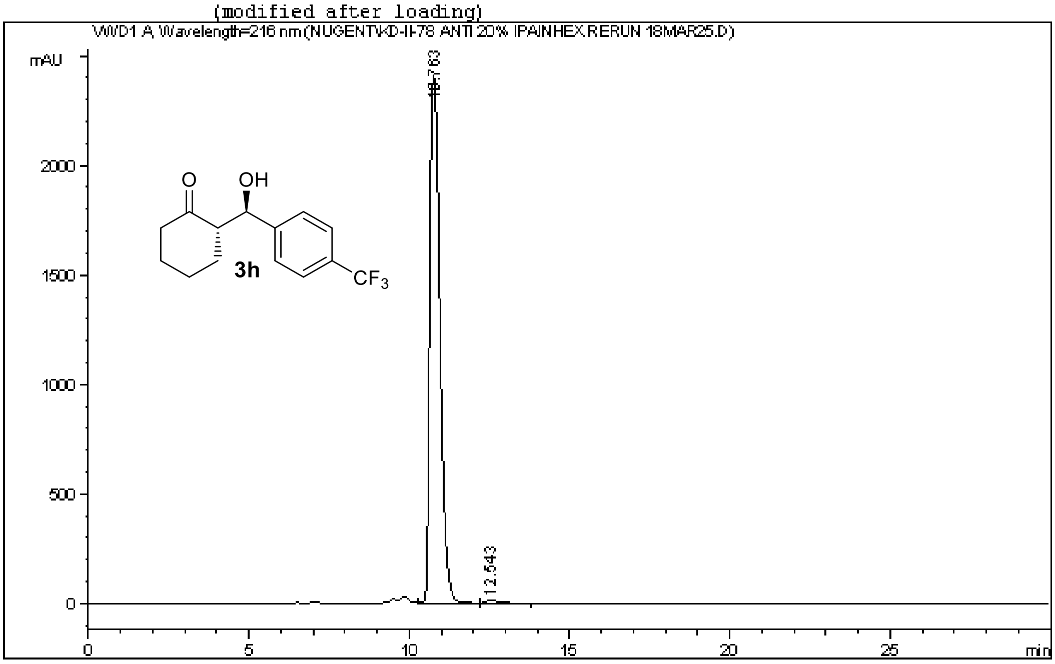

===

Area Percent Report

===

Sorted By : Signal
Multiplier : 1.0000
Dilution : 1.0000
Use Multiplier & Dilution Factor with ISTDs

Signal 1: VWD1 A, Wavelength=216 nm

Peak #	RetTime [min]	Type	Width [min]	Area mAU *s	Height [mAU]	Area %
1	10.763	VV	0.3467	5.33161e4	2411.95703	99.1136
2	12.543	VB	0.5341	476.84586	12.41183	0.8864

Totals : 5.37929e4 2424.36887

===

*** End of Report ***

Instrument 1 3/18/2025 1:48:33 PM Page 1 of 1

Figure S75. HPLC chromatogram of enantioenriched *anti*-aldol (major) product **3h** (above).

Table 3, entry 3: Competition reaction between cyclohexanone and 4-(nitromethyl)-1,2-dihydronaphthalene (2b) for the limiting reactant 3-chlorobenzaldehyde

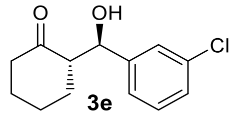

(*S*)-2-((*R*)-(3-chlorophenyl)(hydroxy)methyl)-cyclohexan-1-one (**3e**):
To a clean, screw cap, V-shaped reaction vessel (2.0 mL) equipped with a small pyramidal stir bar, 4-(nitromethyl)-1,2-dihydronaphthalene (**2b**) (MW = 189.21 g/mol, 1.50 equiv, 1.05 mmol, 199.5 mg), cyclohexanone (MW = 98.15 g/mol, 1.50 equiv, 1.05 mmol, 103 mg, 109 µL, density = 0.947 g/mL) and freshly purified (see Section 2) 3-chlorobenzaldehyde (MW = 140.57 g/mol, 1.00 equiv, 0.70 mmol, 98 mg, 79 µL, density = 1.241 g/mL) were added. This mixture was gently stirred for 30 sec resulting in a slightly yellowish solution with no undissolved material. The stirring was terminated and *trans*-4-(*tert*-butyldiphenylsilyloxy)-L-proline catalyst (MW = 369.54 g/mol, 2.5 mol%, 0.0176 mmol, 6.5 mg) was added to the reaction vessel. Within 30 sec after catalyst addition, distilled deoxygenated water (MW = 18.02 g/mol, 15.01 equiv, 10.54 mmol, 190 mg, 190 µL) was added with minimal disruption of the concentrated organic layer. The mixture was stirred such that the contents of the vessel did not splash up and against the walls, but the phase boundary was gently agitated. For observational purposes, the stirring was stopped after 5 min and two phases formed. The top phase was colorless and clear, whereas the bottom layer was pale yellowish color but transparent with no undissolved material. After 32 h, the stirring was terminated, revealing two clear phases, and the bottom layer was now more intensely yellow in color. See Section 2 for the work-up procedure.

This compound was previously synthesized and characterized, see below.

Crude product ^{1}H NMR analysis (Figure S76, see below) allowed determination of the following diastereo- and chemoselectivity ratios.

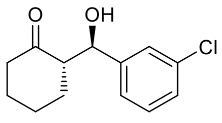

anti-aldol (major) **3e**

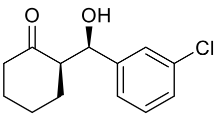

syn-aldol (minor) **4e**

Diastereoselectivity: The *anti*-aldol/*syn*-aldol product ratio was determined as 15.7:1 from the benzylic proton resonances at 4.76 ppm (d, *anti*-aldol product) and 5.37 ppm (d, *syn*-aldol product) in the crude ^{1}H NMR spectrum (Figure S76). For the chemical shift literature values of *anti*- and *syn*-aldol products, see page SI 6 of the supporting information of: Martínez-Castañeda, Á.; Rodríguez-Solla, H.; Concellón, C.; del Amo, V. TBD/Al_2O_3: A Novel Catalytic System for Dynamic Intermolecular Aldol Reactions that Exhibit Complex System Behavior. *Org. Biomol. Chem.* **2012**, *10*, 1976-1981.

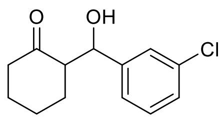

aldol products **3e**/**4e**

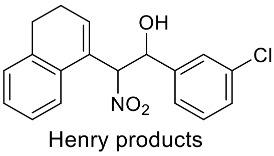

Henry products

Chemoselectivity: The *anti*- and *syn*-aldol/ *anti*- and *syn*-Henry products ratio was determined to be >19:1. This Henry product is not reported in the literature, and we have not synthesized it. However, based on related Henry products we have synthesized (see Section 5 of this document), no resonances corresponding to the indicative chemical shifts, -C**H**(NO_2)-C**H**(OH)Ar, of the potential Henry products were observed in the crude ^{1}H NMR (Figure S76) of this competition reaction.

Purification and yield: Silica gel chromatography (3.2 cm column outer diameter, 18 cm silica bed height) was performed using isocratic elution (15 vol% ethyl acetate in petroleum ether). The crude product was loaded onto the column in a minimum volume of CH_2Cl_2. The *anti*-aldol product **3e** was isolated as an off-white solid (113 mg, MW = 238.71 g/mol, 0.473 mmol, 68% yield). Note: Repeating the same reaction with an extended reaction of 49 h resulted in a 70% yield. Here we have provided the data for the 32 h reaction.

R_f: *anti*-aldol product = 0.18, *syn*-aldol product = 0.24 (EtOAc/petroleum ether, 15:85).

98% ee: Chiralcel OD-H chiral HPLC column, iPrOH/n-hexane (5:95), flow rate = 1.0 mL/min, λ = 210 nm, injection volume = 20 µL, the sample was dissolved in 5 vol% iPrOH/n-hexane with a concentration of ≈ 1 mg/mL; *anti*-aldol product retention times: t_{major} = 10.9 min, t_{minor} = 15.7 min. (Figures S78 and S79).

^{1}H NMR (400 MHz, $CDCl_3$) (ppm) *anti*-aldol product **3e** (Figure S77): δ 7.35-7.33 (m, 1H), 7.29-7.25 (m, 2H), 7.21-7.16 (m, 1H), 4.76 (dd, 1H, *J* = 8.7, 2.8 Hz), 4.01 (d, 1H, *J* = 2.9 Hz), 2.62-2.54 (m, 1H), 2.52-2.45 (m, 1H), 2.41-2.31 (m, 1H), 2.15-2.06 (m, 1H), 1.85-1.77 (m, 1H), 1.73-1.50 (m, 3H), 1.37-1.25 (m, 1H).

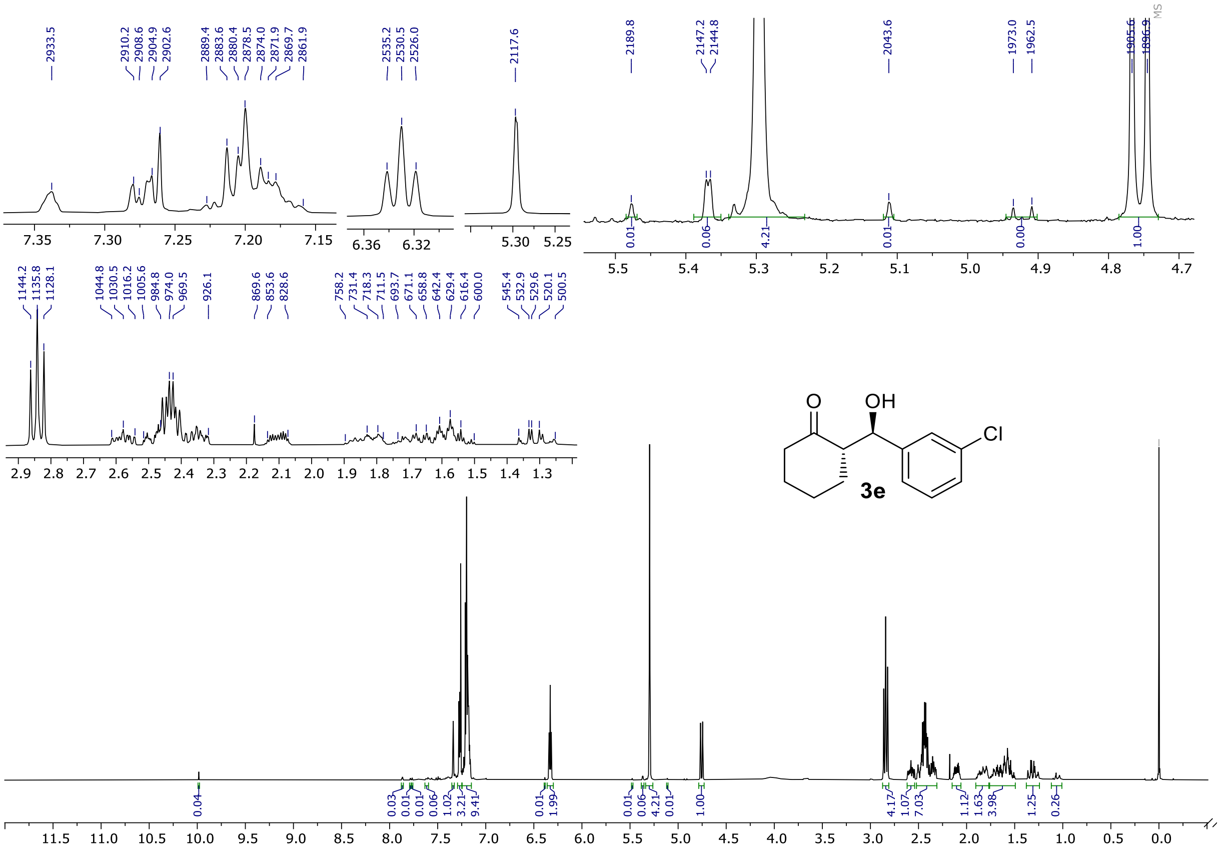

Figure S76. Crude ^{1}H NMR spectrum after high vacuum drying of **3e** (above).

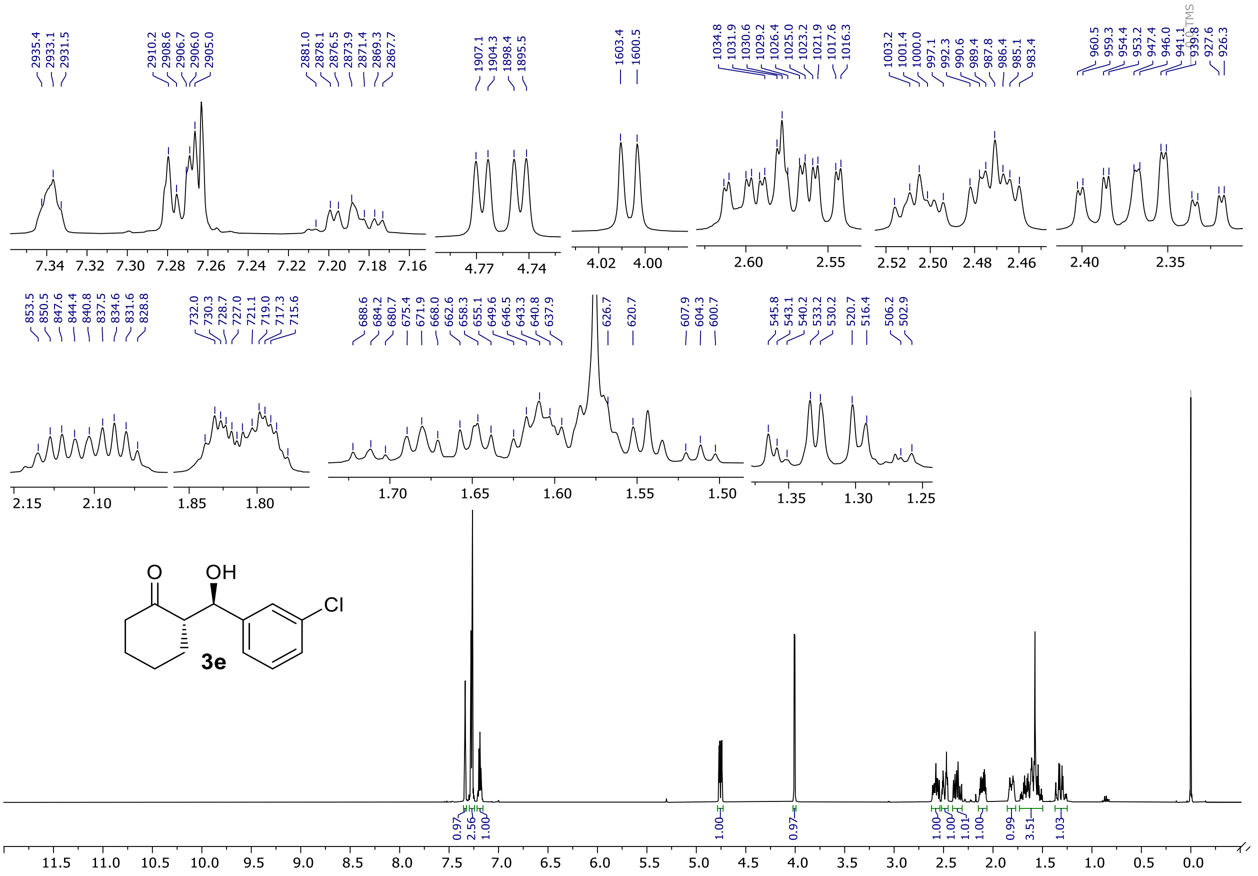

Figure S77. ^{1}H NMR spectrum of the purified *anti*-aldol (major) product **3e** (above).

Data File D:\DATA\NUGENT\AL24 RAC 5%IPANHEX 06FEB25 RE.D
Sample Name: AL24 RAC 5%IPAnHex 06feb25 re

===

```
Acq. Operator   :                                    Seq. Line :   2
Acq. Instrument : Instrument 1                        Location : P1-A-03
Injection Date  : 2/6/2025 1:35:39 PM                      Inj :   1
                                                   Inj Volume : 20 µl
Acq. Method     : D:\Methods\Nugentlab_Patrick\AIDA.m
Last changed    : 2/6/2025 1:34:31 PM
Analysis Method : D:\Methods\Nugentlab_Patrick\AIDA254NM1.m
Last changed    : 2/6/2025 2:01:03 PM
                  (modified after loading)
```

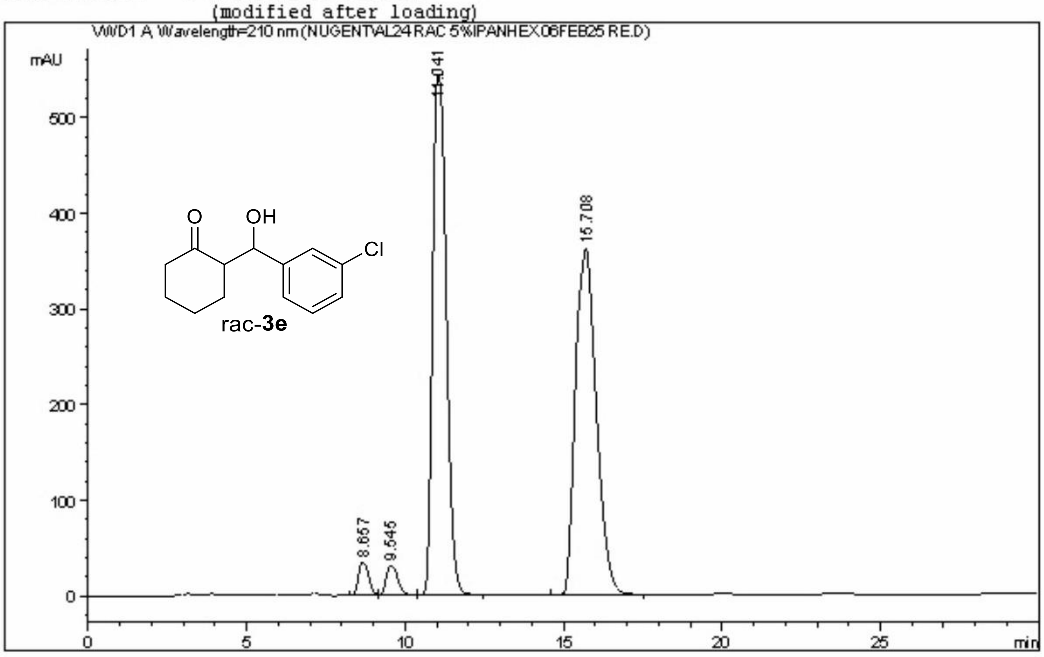

===
Area Percent Report
===

```
Sorted By             :      Signal
Multiplier            :      1.0000
Dilution              :      1.0000
Use Multiplier & Dilution Factor with ISTDs
```

Signal 1: VWD1 A, Wavelength=210 nm

Peak #	RetTime [min]	Type	Width [min]	Area mAU *s	Height [mAU]	Area %
1	8.657	BV	0.3643	785.40881	34.54132	2.2828
2	9.545	VB	0.4170	804.99719	30.80102	2.3397
3	11.041	BB	0.4747	1.62915e4	543.91724	47.3515
4	15.708	BB	0.7207	1.65235e4	361.77737	48.0260
Totals :				3.44054e4	971.03695	

===
*** End of Report ***

Instrument 1 2/6/2025 2:06:54 PM

Page 1 of 1

Figure S78. HPLC chromatogram of the racemic *anti*-aldol product **3e** (above).

Data File D:\DATA\NUGENT\PK 025 5% IPAINHEX 04MAR25.D
Sample Name: PK 025 5% IPAinHEX 04MAR25

===

Acq. Operator   :   Seq. Line :   2
Acq. Instrument : Instrument 1   Location : P1-A-05
Injection Date : 3/4/2025 2:24:36 PM   Inj :   1
                                                Inj Volume : 20 µl
Acq. Method   : D:\Methods\Nugentlab_Patrick\AIDA 5IPA 210NM.m
Last changed   : 3/4/2025 2:23:09 PM
Analysis Method : D:\Methods\Nugentlab_Patrick\AIDA 15IPA 254NM.m
Last changed   : 3/3/2025 4:17:17 PM
                  (modified after loading)

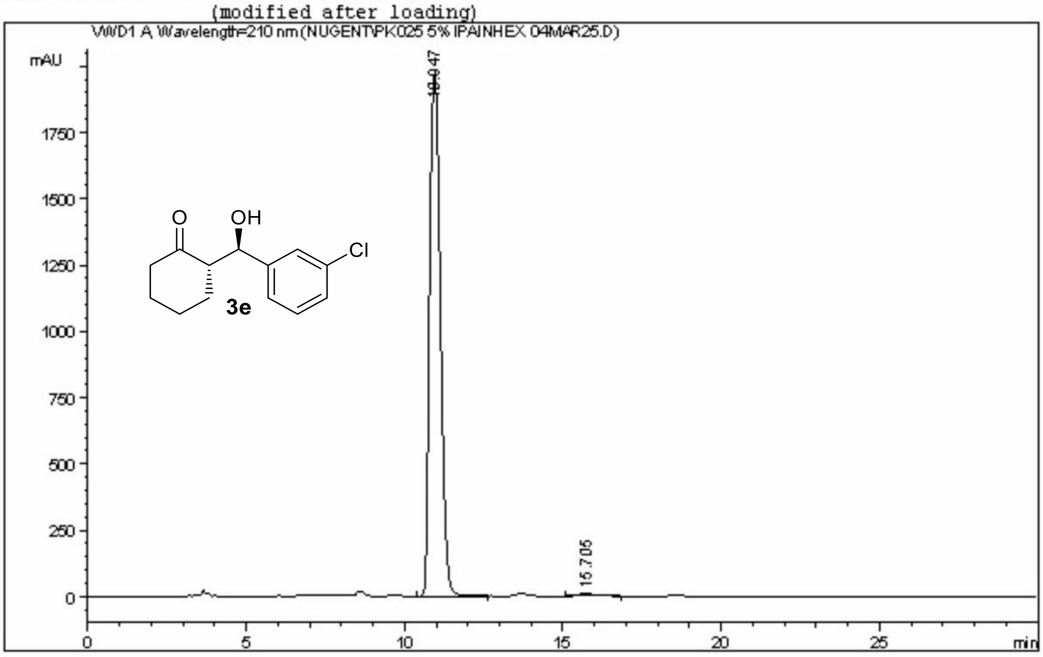

===

Area Percent Report

===

Sorted By   :   Signal
Multiplier   :   1.0000
Dilution   :   1.0000
Use Multiplier & Dilution Factor with ISTDs

Signal 1: VWD1 A, Wavelength=210 nm

Peak #	RetTime [min]	Type	Width [min]	Area mAU *s	Height [mAU]	Area %
1	10.947	BB	0.3813	4.65670e4	1965.25220	99.1778
2	15.705	BB	0.5578	386.02875	10.27357	0.8222
Totals :				4.69530e4	1975.52577	

===

*** End of Report ***

Instrument 1 3/4/2025 3:59:26 PM

Page 1 of 1

Figure S79. HPLC chromatogram of enantioenriched *anti*-aldol (major) product **3e** (above).

Table 3, entry 4: Competition reaction between cyclohexanone and 4-(nitromethyl)-1,2-dihydronaphthalene (2b) for the limiting reactant 4-(trifluoromethyl)benzaldehyde

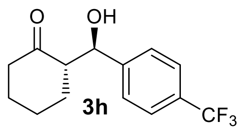

(*S*)-2-((*R*)-hydroxy(4-(trifluoromethyl)phenyl)methyl)-cyclohexan-1-one (**3h**): To a clean, screw cap, V-shaped reaction vessel (2.0 mL) equipped with a small pyramidal stir bar, 4-(nitromethyl)-1,2-dihydronaphthalene (**2b**) (MW = 189.21 g/mol, 1.50 equiv, 1.05 mmol, 199.0 mg), cyclohexanone (MW = 98.15 g/mol, 1.50 equiv, 1.05 mmol, 103 mg, 109 µL, density 0.947 g/mL) and freshly purified (see Section 2) 4-(trifluoromethyl)benzaldehyde (MW = 174.12 g/mol, 1.00 equiv, 0.70 mmol, 122 mg, 95.5 µL, density 1.275 g/mL) were added. This mixture was gently stirred for 30 sec resulting in a slightly yellowish solution with no undissolved material. The stirring was terminated and the *trans*-4-(*tert*-butyldiphenylsilyloxy)-L-proline catalyst (MW = 369.54 g/mol, 2.6 mol%, 0.0179 mmol, 6.6 mg) [6.6 mg was accidentally used, resulting in a 2.6 mol% (=2.55 mol%) catalyst loading] was added to the reaction vessel. Within 30 sec after catalyst addition, distilled deoxygenated water (MW = 18.02 g/mol, 15.01 equiv, 10.54 mmol, 190 mg, 190 µL) was added with minimal disruption of the concentrated organic layer. The mixture was stirred such that the contents of the vessel did not splash up and against the walls, but the phase boundary was gently agitated. For observational purposes, the stirring was stopped after 5 min and two phases formed. The top phase was colorless and clear, whereas the bottom layer was slightly yellowish colored with no undissolved material. Over the 32 h reaction time, the concentrated organic layer progressively became more orange in color. See Section 2 for the work-up procedure (32 h).

This compound was previously synthesized and characterized, see below.

Crude product ^{1}H NMR analysis (Figure S80, see below) allowed determination of the following diastereo- and chemoselectivity ratios.

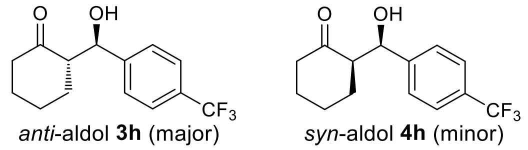

Diastereoselectivity: The *anti*-aldol/*syn*-aldol product ratio was determined as 12.5:1 from the benzylic proton resonances at 4.85 ppm (d, *anti*-aldol product) and 5.44 ppm (bs, *syn*-aldol product) in the crude ^{1}H NMR spectrum (Figure S80). For literature regarding the *syn*-aldol product chemical shift, see page 14 of the Supporting Information within: Gao, J.; Bai, S.; Gao, Q.; Liu, Y.; Yang, Q. Acid Controlled Diastereoselectivity in Asymmetric Aldol Reaction of Cycloketones with Aldehydes using Enamine-Based Organocatalysts. *Chem. Commun.* **2011**, *47*, 6716–6718. For the chemical shift literature value for the *anti*-aldol product, see page S8 of the supporting information of: Al Beiruty, H.; Zhylinska, S.-S.; Kutateladze, N.; Cheong, H. K. T.; Ñíguez, J. A.; Burlingham, S. J.; Marset X.; Guillena, G.; Chinchilla, R.; Alonso, D. A.; Nugent, T. C. Enantioselective Catalytic Aldol Reactions in the Presence of Knoevenagel Nucleophiles: A Chemoselective Switch Optimized in Deep Eutectic Solvents Using Mechanochemistry. *Molecules* **2024**, *29*, 4. https://doi.org/10.3390/molecules29010004

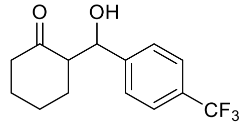

aldol products **3h**/**4h**

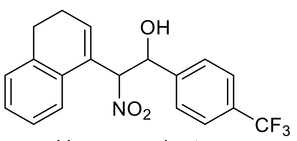

Henry products

Chemoselectivity: The *anti*- and *syn*-aldol/*anti*- and *syn*-Henry product ratio was determined to be >19:1. The Henry product is not reported in the literature, and we have not synthesized it. However, based on related Henry products we have synthesized (see Section 5 of this document), resonances corresponding to the indicative chemical shifts, -C**H**(NO_2)-C**H**(OH)Ar, of the potential Henry products were observed in the crude ^{1}H NMR (Figure S80) but had insignificant integration areas. See, the expansion from 4.80 to 5.70 ppm.

Purification and yield: Silica gel chromatography (2.5 cm column outer diameter, 20 cm silica bed height) was performed using isocratic elution (20 vol% ethyl acetate in petroleum ether). The crude product was loaded onto the column in a minimum volume of CH_2Cl_2. The *anti*-aldol product **3h** was isolated as an off-white solid (141 mg, MW = 272.27 g/mol, 0.518 mmol, 74% yield).

R_f: 0.23 (*anti*-aldol product), EtOAc/petroleum ether (1:4).

99% *ee*: Chiralcel OD-H chiral HPLC column, iPrOH/n-hexane (20:80), flow rate = 0.5 mL/min, λ = 216 nm, injection volume = 20 µL, the sample was dissolved in 20 vol% iPrOH/n-hexane with a concentration of ≈ 1 mg/mL; *anti*-aldol product retention times: t_{major} = 10.6 min, t_{minor} = 12.3 min. (Figures S82 and S83).

^{1}H NMR (400 MHz, $CDCl_3$) (ppm) *anti*-aldol product **3h** (Figure S81): δ 7.61 (d, 2H, *J* = 8.3 Hz), 7.45 (d, 2H, *J* = 8.1 Hz), 4.85 (dd, 1H, *J* = 8.6, 3.0 Hz), 4.05 (d, 1H, *J* = 3.0 Hz), 2.64-2.55 (m, 1H), 2.53-2.46 (m, 1H), 2.42-2.32 (m, 1H), 2.15-2.07 (m, 1H), 1.85-1.77 (m, 1H), 1.74-1.48 (m, 3H), 1.40-1.27 (m, 1H).

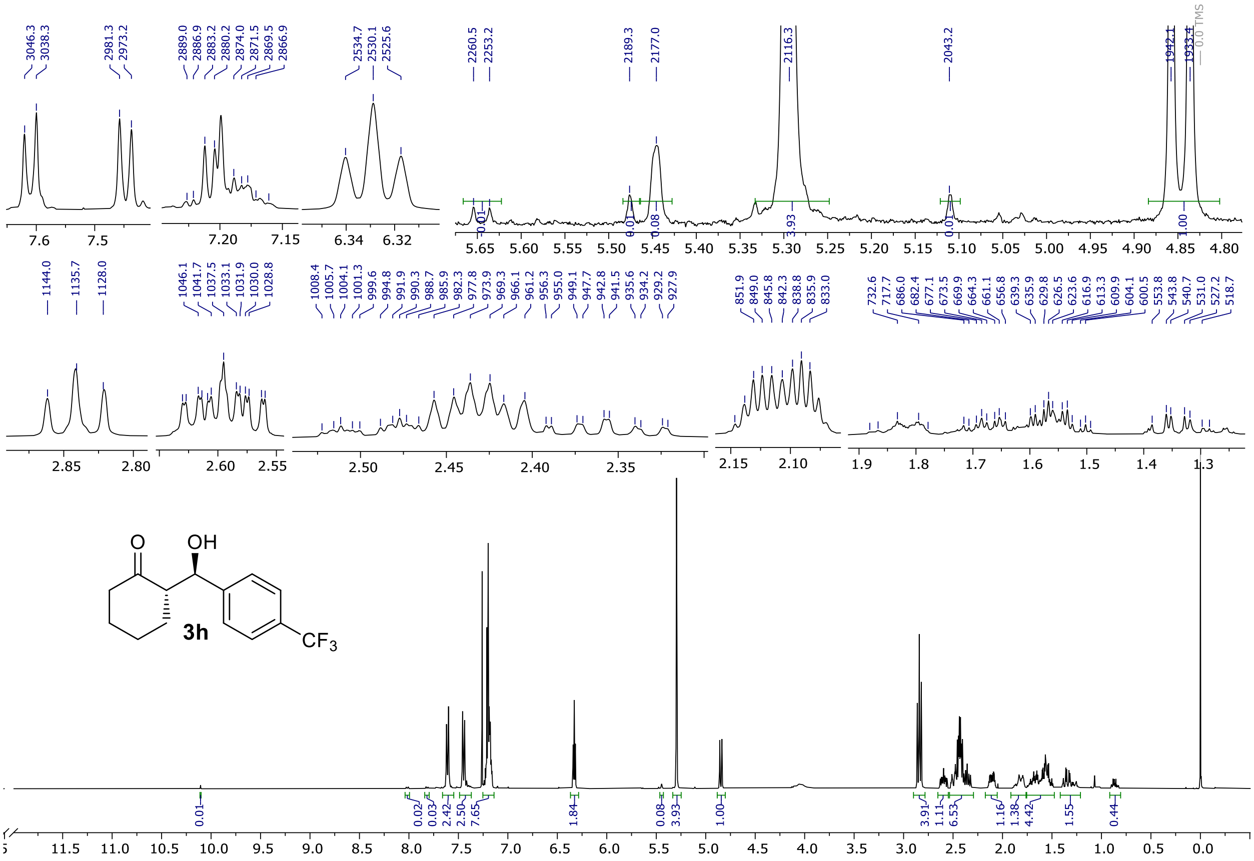

Figure S80. Crude ^{1}H NMR spectrum after high vacuum drying of **3h** (above).

Note that ^{1}H NMR data of crude product **3h**, after work-up and drying, of the reaction between cyclohexanone and 4-trifluoromethylbenzaldehyde was problematic. Each sample from the semi-solid product provided a different chemoselectivity. For that reason, the entire crude product was dissolved in $CDCl_3$ and only then did we record the ^{1}H NMR.

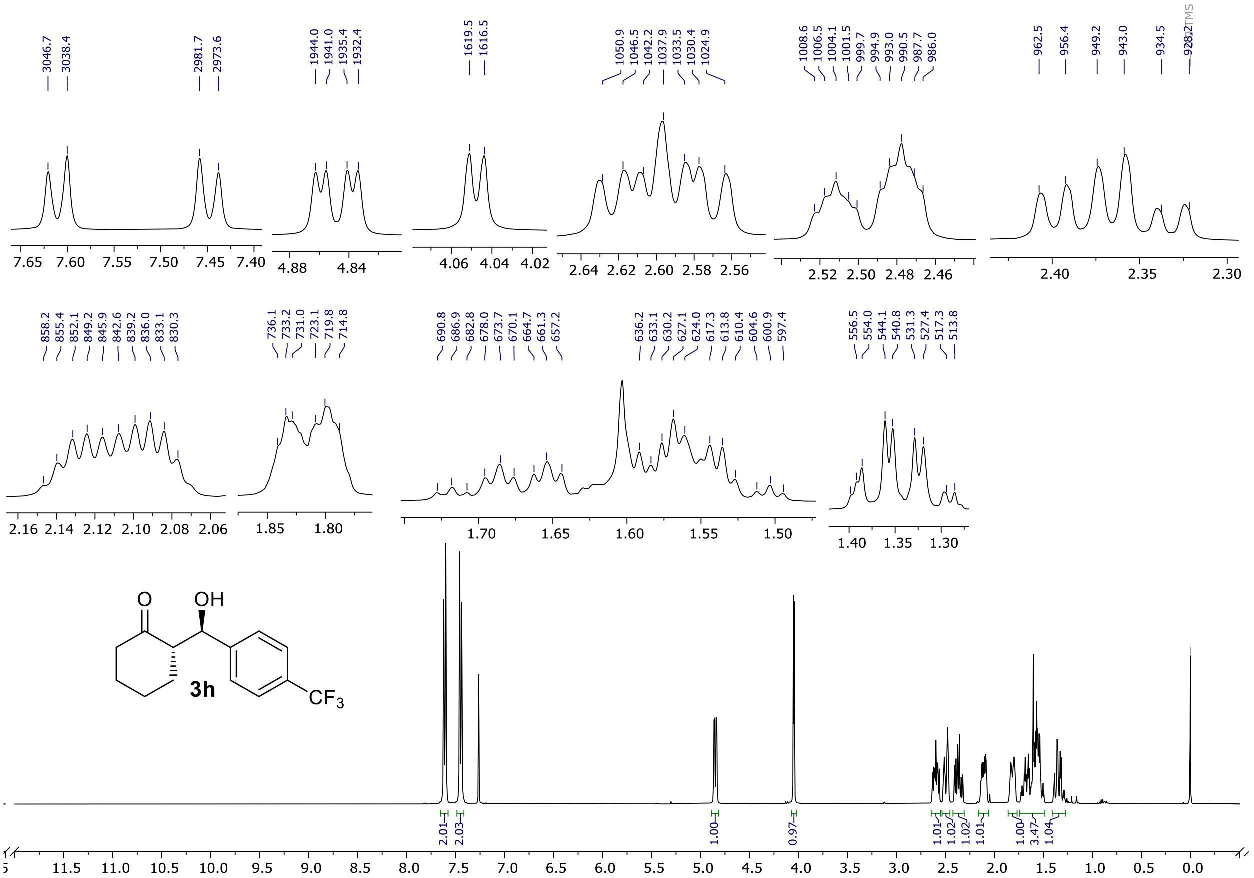

Figure S81. ^{1}H NMR spectrum of purified *anti*-aldol (major) product **3h** (above).

SHIMADZU LabSolutions **Analysis Report**

<Sample Information>

Sample Name	: HC-I-26 20%IPAnHex 20uL 0.5 mLmin 08Feb23 1mgmL
Sample ID	:
Data Filename	: HC-I-26 20%IPAnHex 20uL 0.5 mLmin 08Feb23 1mgmL.lcd
Method Filename	: trial.lcm
Batch Filename	:
Vial #	: 1-4
Sample Type	: Unknown
Injection Volume	: 20 uL
Date Acquired	: 2/8/2023 3:15:20 PM
Acquired by	: System Administrator
Date Processed	: 2/8/2023 3:39:35 PM
Processed by	: System Administrator

<Chromatogram>

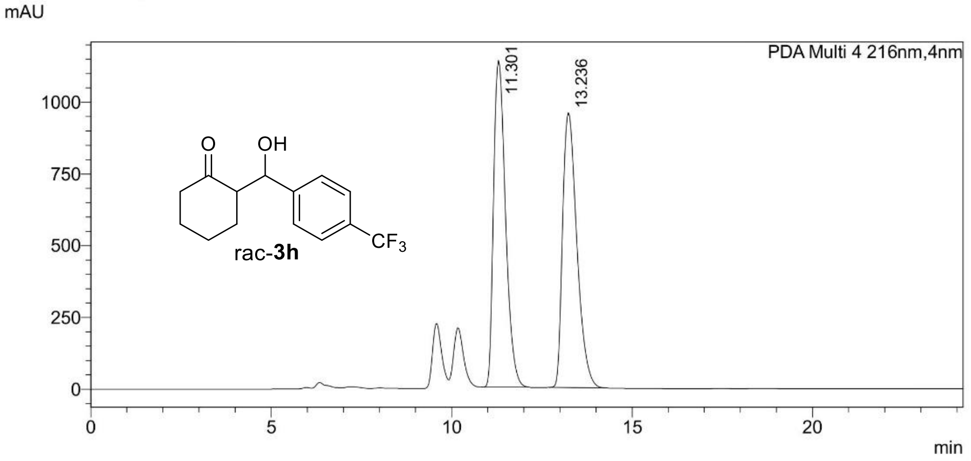

<Peak Table>

PDA Ch4 216nm

Peak#	Ret. Time	Area	Height	Area%
1	11.301	26277677	1138787	49.940
2	13.236	26341028	958736	50.060
Total		52618705	2097523	100.000

C:\Users\Shimadzu\Desktop\Hayley\HC-I-26 20%IPAnHex 20uL 0.5 mLmin 08Feb23 1mgmL.lcd

Figure S82. HPLC chromatogram of the racemic *anti*-aldol product **3h** (above).

```
Data File D:\DATA\NUGENT\PK 030 20% IPAINHEX  04MAR25.D
Sample Name: PK 030 20% IPAinHEX  04MAR25

=====================================================================
Acq. Operator   :                                   Seq. Line :   4
Acq. Instrument : Instrument 1                       Location : P1-A-07
Injection Date  : 3/4/2025 3:27:47 PM                     Inj :   1
                                                   Inj Volume : 20 µl
Acq. Method     : D:\Methods\Nugentlab_Patrick\AIDA 5IPA 210NM.m
Last changed    : 3/4/2025 3:26:37 PM
Analysis Method : D:\Methods\Nugentlab_Patrick\AIDA 15IPA 254NM.m
Last changed    : 3/3/2025 4:17:17 PM
                  (modified after loading)
```

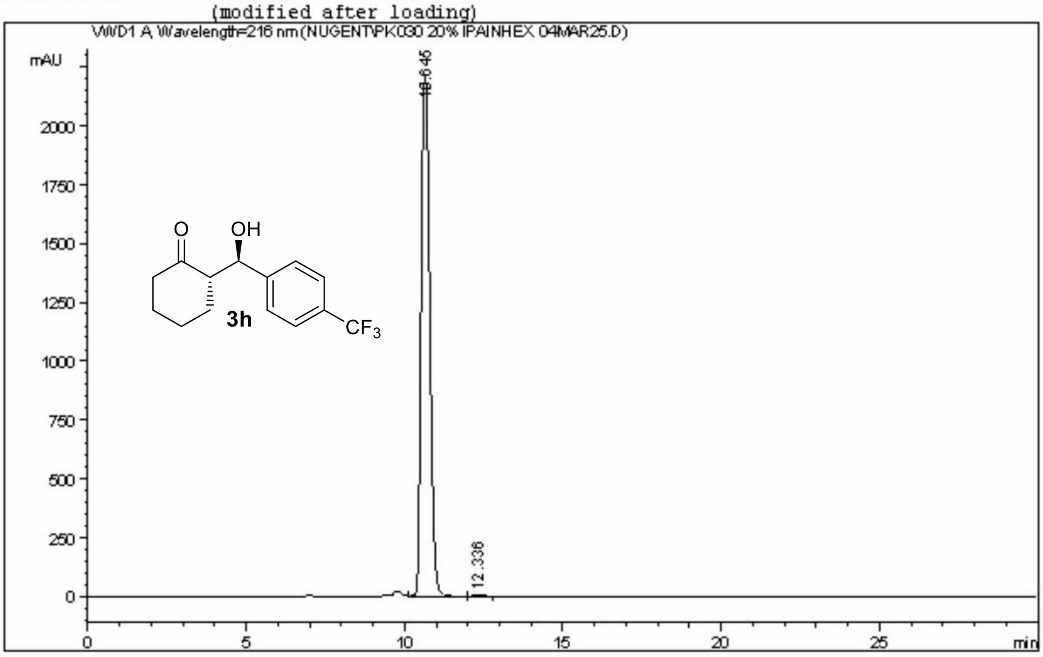


```
=====================================================================
                         Area Percent Report
=====================================================================

Sorted By             :      Signal
Multiplier            :      1.0000
Dilution              :      1.0000
Use Multiplier & Dilution Factor with ISTDs


Signal 1: VWD1 A, Wavelength=216 nm

Peak RetTime Type  Width     Area      Height     Area
  #   [min]        [min]  mAU   *s   [mAU   ]      %
----|-------|----|-------|----------|----------|--------|
   1  10.645 VB    0.3029 4.20733e4  2217.66968 99.5147
   2  12.336 BV    0.3558  205.16342    8.86836  0.4853

Totals :                  4.22784e4  2226.53804

=====================================================================
                         *** End of Report ***
```

Instrument 1 3/4/2025 4:02:14 PM

Page 1 of 1

Figure S83. HPLC chromatogram of enantioenriched *anti*-aldol (major) product **3h** (above).

Table 3, entry 5: Competition reaction between cyclohexanone and 3-(nitromethyl)-1,2-dihydronaphthalene (2c) for the limiting reactant 3-chlorobenzaldehyde

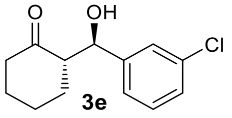

***(S)*-2-((*R*)-(3-chlorophenyl)(hydroxy)methyl)-cyclohexan-1-one** (**3e**):
To a clean, screw cap, V-shaped reaction vessel (2.0 mL) equipped with a small pyramidal stir bar, 3-(nitromethyl)-1,2-dihydronaphthalene (**2c**) (MW = 189.21 g/mol, 1.50 equiv, 1.05 mmol, 199 mg), cyclohexanone (MW = 98.15 g/mol, 1.50 equiv, 1.05 mmol, 103 mg, 109 µL, density = 0.947 g/mL) and freshly purified (see Section 2) 3-chlorobenzaldehyde (MW = 140.57 g/mol, 1.00 equiv, 0.70 mmol, 98 mg, 79 µL, density = 1.241 g/mL) were added. This mixture was gently stirred for 5 min resulting in a yellow colored clear solution with no undissolved material. The stirring was terminated and the *trans*-4-(*tert*-butyldiphenylsilyloxy)-L-proline catalyst (MW = 369.54 g/mol, 2.5 mol%, 0.0175 mmol, 6.47 mg) was added to the reaction vessel. Within 30 sec of catalyst addition, distilled deoxygenated water (MW = 18.02 g/mol, 15.01 equiv, 10.54 mmol, 190 mg, 190 µL) was added with minimal disruption to the concentrated organic layer. The mixture was stirred such that the contents of the vessel did not splash up and against the vessel walls, but the phase boundary was always gently agitated. For observational purposes, the stirring was stopped after 5 min and two phases formed. No undissolved solids were noted and the organic phase was transparent and yellow colored. After 15 min the stirring was again stopped for better observation and the organic phase was now a suspension. At 2 h, the organic phase became fully transparent with no undissolved material and remained so until the end of reaction (32 h). See Section 2 for the work-up procedure.

This compound was previously synthesized and characterized, see below.

Crude product ^{1}H NMR analysis (Figure S84, see below) allowed determination of the following diastereo- and chemoselectivity ratios.

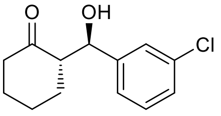

anti-aldol (major) **3e**

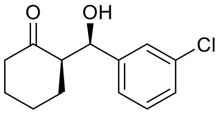

syn-aldol (minor) **4e**

Diastereoselectivity: The *anti*-aldol/*syn*-aldol product ratio was determined as 12.5:1 based on the integration of the benzylic proton resonances at 4.75 ppm (d, *anti*-aldol product) and 5.36 ppm (bs, *syn*-aldol product) in the crude ^{1}H NMR spectrum (Figure S84). For the chemical shift literature values of *anti*- and *syn*-aldol products, see page SI 6 of the supporting information of: Martínez-Castañeda, Á.; Rodríguez-Solla, H.; Concellón, C.; del Amo, V. TBD/Al_2O_3: A Novel Catalytic System for Dynamic Intermolecular Aldol Reactions that Exhibit Complex System Behavior. *Org. Biomol. Chem.* **2012**, *10*, 1976-1981.

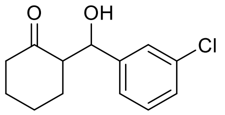

aldol products **3e**/**4e**

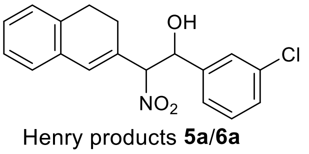

Henry products **5a**/**6a**

Chemoselectivity: The *anti*- and *syn*-aldol/*anti*- and *syn*-Henry product ratio was determined to be 6.35:1 based on the integration of the benzylic proton resonances at 4.75 ppm (d, *anti*-aldol product) and 5.36 ppm (bs, *syn*-aldol product) compared to the benzylic proton resonances at 5.45 ppm (d, C**H**(Ph)OH, *syn*-Henry product) and 5.48 ppm (d, C**H**(Ph)OH, *anti*-Henry product) in the crude ^{1}H NMR spectrum (Figure S84). We also isolated the Henry products **5a** and **6a** in pure form and their ^{1}H NMRs can be found in Section 5 of this document.

Purification and yield: The crude product was loaded onto a silica gel (230-400 mesh) column (16 cm in height, 2 cm in diameter) pre-wetted with EtOAc/petroleum ether (1:19). The crude product was loaded onto the column in a minimum volume of CH_2Cl_2. The mobile phase elution began with EtOAc/petroleum ether (1:19). This solvent ratio was maintained until the starting materials were

removed from the column. The polarity of the mobile phase was raised to EtOAc/petroleum ether (7:93) to remove the Henry products and the *syn*-aldol product. The *anti*-aldol product eluted after use of EtOAc/petroleum ether (1:9). Concentration of the pure fractions provided white solid (MW= 238.71 g/mol, 127 mg, 0.532 mmol, 76% yield) of the *anti*-aldol product **3e**.

R_f: 0.17 (*anti*-aldol product), EtOAc/petroleum ether (15:85).

98% *ee*: Chiralcel OD-H chiral HPLC column, iPrOH/n-hexane (5:95), flow rate = 1.0 mL/min, λ = 210 nm, injection volume = 6 µL, the sample was dissolved in 5 vol% iPrOH/n-hexane with a concentration of ≈ 1 mg/mL; *anti*-aldol product retention times: t_{major} = 11.2 min, t_{minor} = 16.0 min. (Figured S86 and S87).

^{1}H NMR (400 MHz, $CDCl_3$) (ppm) *anti*-aldol product **3e** (Figure S85): δ 7.34 (s, 1H), 7.30-7.23 (m, 2H), 7.19 (m, 1H), 4.75 (dd, 1H, J = 8.7, 2.9 Hz), 4.00 (d, 1H, J = 2.7 Hz), 2.62-2.53 (m, 1H), 2.52-2.44 (m, 1H), 2.41-2.30 (m, 1H), 2.10 (m, 1H), 1.86-1.76 (m, 1H), 1.74-1.48 (m, 2H), 1.31 (m, 1H).

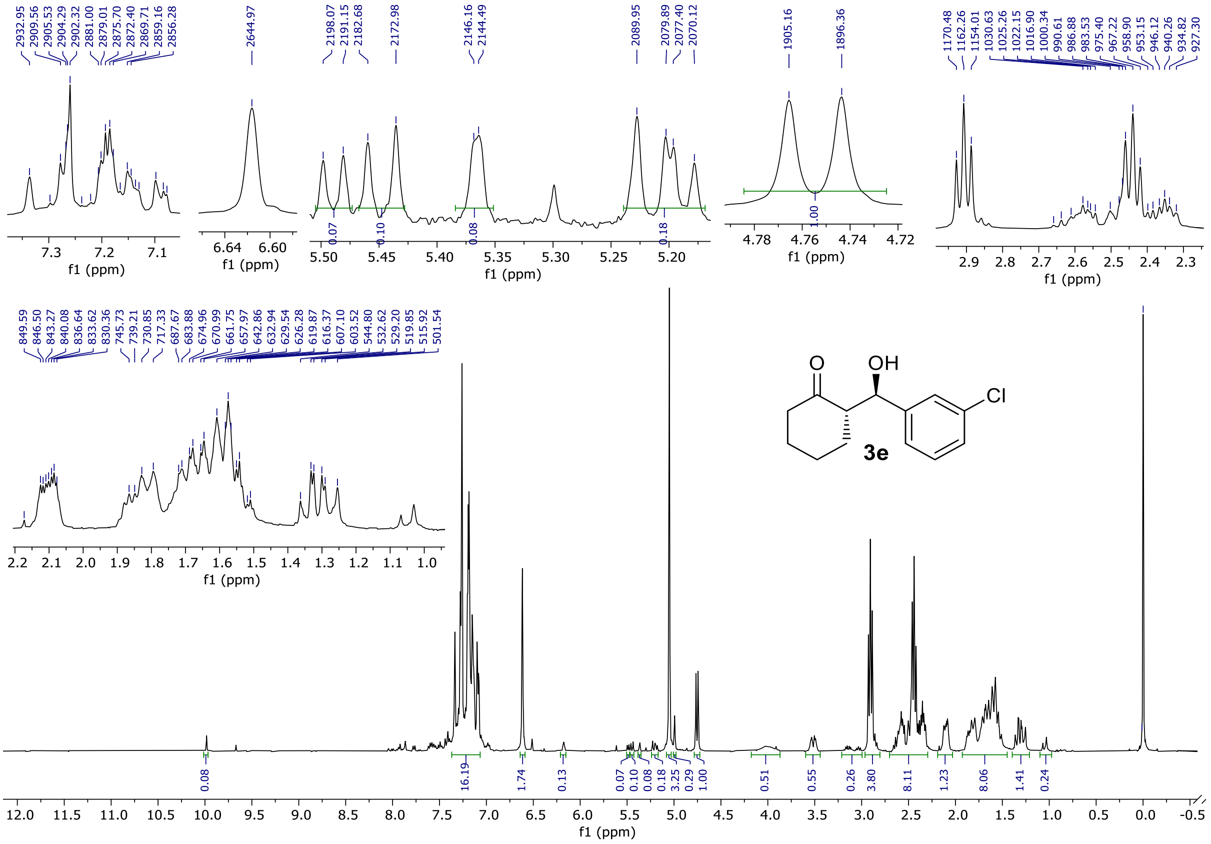

Figure S84. Crude ^{1}H NMR spectrum after high vacuum drying of **3e** (above).

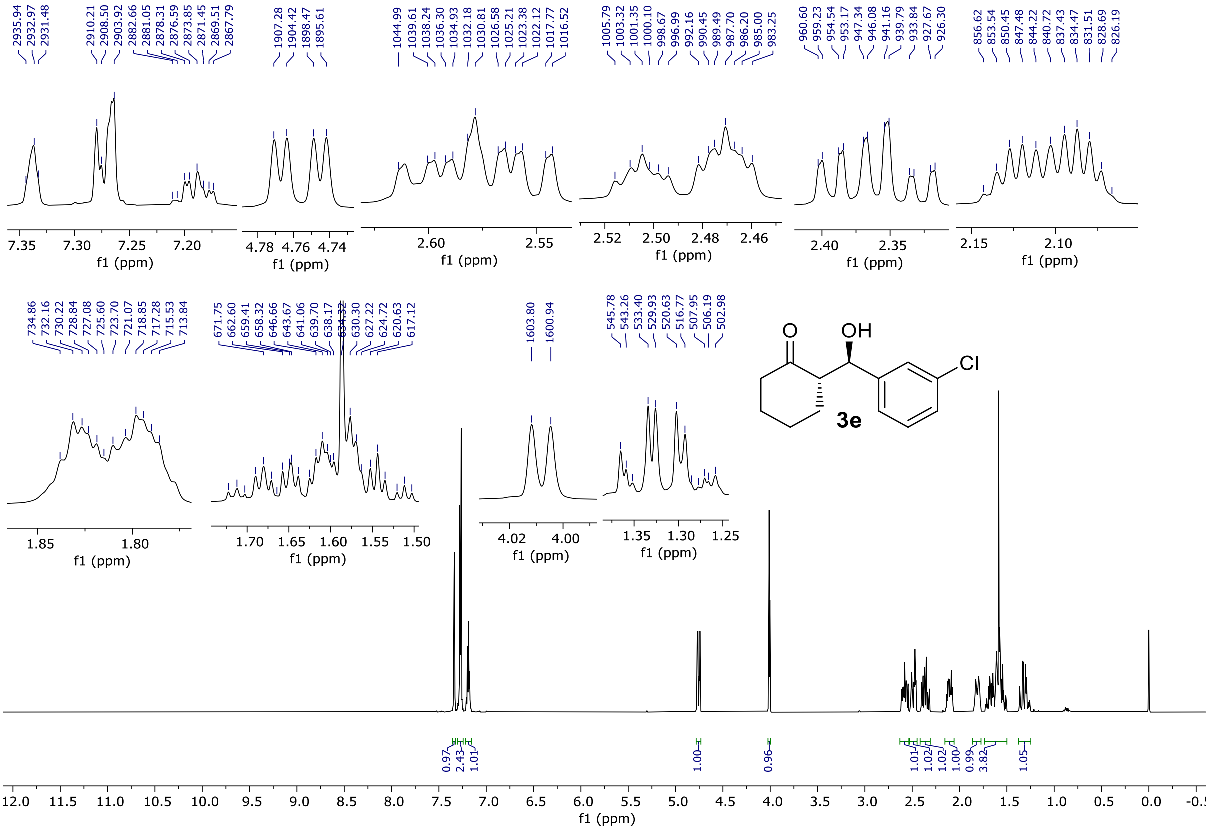

Figure S85. ^{1}H NMR spectrum of the purified *anti*-aldol (major) product **3e** (above).

Data File D:\DATA\NUGENT\AL24 RAC 5%IPANHEX 06FEB25 RE.D
Sample Name: AL24 RAC 5%IPAnHex 06feb25 re

===

Acq. Operator : Seq. Line : 2
Acq. Instrument : Instrument 1 Location : P1-A-03
Injection Date : 2/6/2025 1:35:39 PM Inj : 1
Inj Volume : 20 µl
Acq. Method : D:\Methods\Nugentlab_Patrick\AIDA.m
Last changed : 2/6/2025 1:34:31 PM
Analysis Method : D:\Methods\Nugentlab_Patrick\AIDA254NM1.m
Last changed : 2/6/2025 2:01:03 PM
(modified after loading)

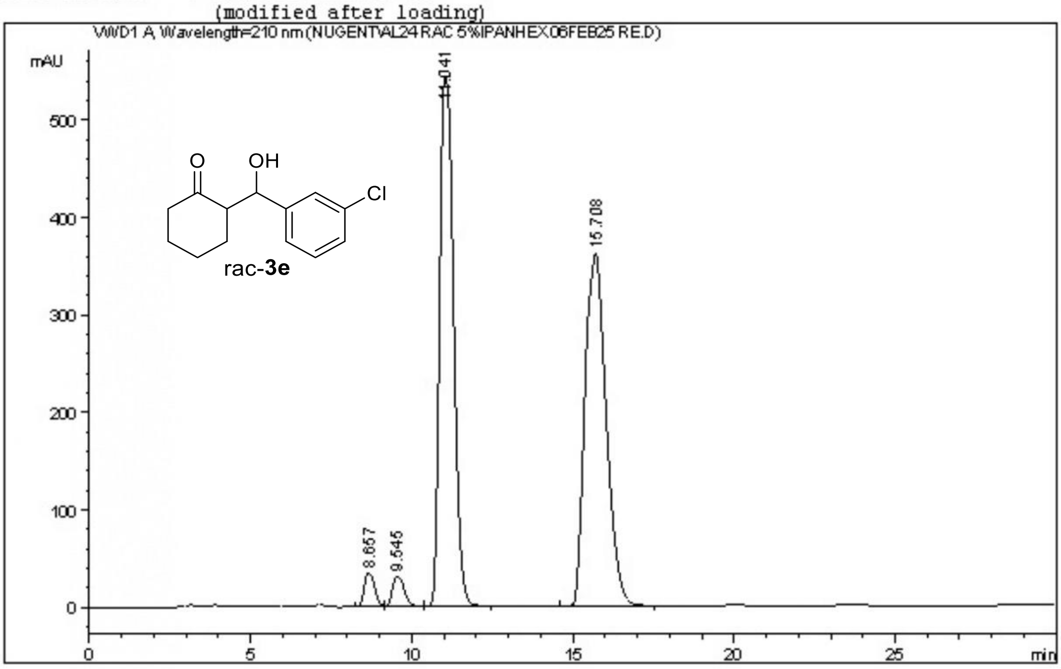

===

Area Percent Report

===

Sorted By : Signal
Multiplier : 1.0000
Dilution : 1.0000
Use Multiplier & Dilution Factor with ISTDs

Signal 1: VWD1 A, Wavelength=210 nm

Peak #	RetTime [min]	Type	Width [min]	Area mAU *s	Height [mAU]	Area %
1	8.657	BV	0.3643	785.40881	34.54132	2.2828
2	9.545	VB	0.4170	804.99719	30.80102	2.3397
3	11.041	BB	0.4747	1.62915e4	543.91724	47.3515
4	15.708	BB	0.7207	1.65235e4	361.77737	48.0260
Totals :				3.44054e4	971.03695	

===

*** End of Report ***

Instrument 1 2/6/2025 2:06:54 PM Page 1 of 1

Figure S86. HPLC chromatogram of the racemic *anti*-aldol product **3e** (above).

SHIMADZU LabSolutions **Analysis Report**

<Sample Information>

Sample Name	: WATER-32H		
Data Filename	: WATER-32H.lcd		
Method Filename	: RL5-5%IPANHEX-1000PPM-SAM[5%IPANHEX].lcm		
Vial #	: 1-9	Sample Type	: Unknown
Injection Volume	: 6 uL		
Date Acquired	: 6/11/2025 12:25:31 PM	Acquired by	: System Administrator
Date Processed	: 6/11/2025 1:00:24 PM	Processed by	: System Administrator

<Chromatogram>

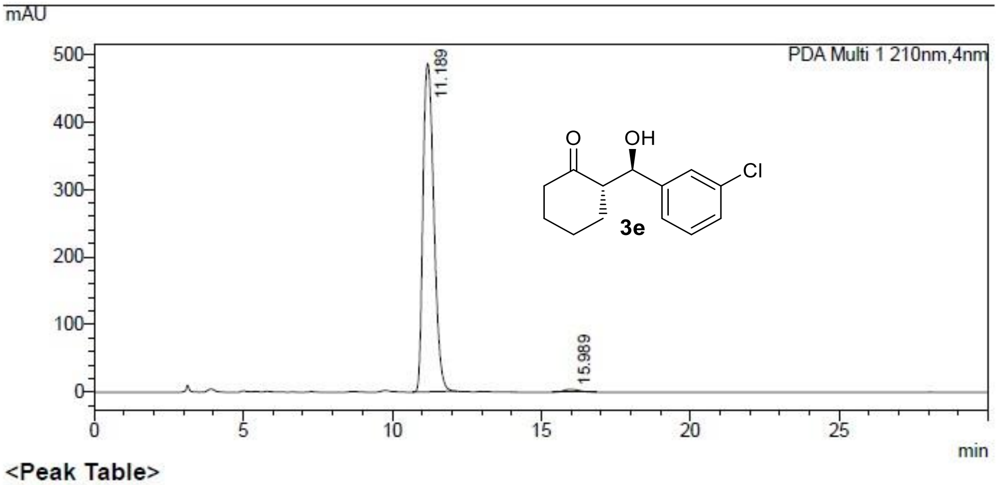

<Peak Table>

PDA Ch1 210nm

Peak#	Ret. Time	Area	Height	Area%
1	11.189	12277250	485398	98.885
2	15.989	138373	3980	1.115
Total		12415623	489378	100.000

Figure S87. HPLC chromatogram of enantioenriched *anti*-aldol (major) product **3e** (above).

Table 3, entry 6: Competition reaction between cyclohexanone and 3-(nitromethyl)-1,2-dihydronaphthalene (2c) for the limiting reactant 4-(trifluoromethyl)benzaldehyde

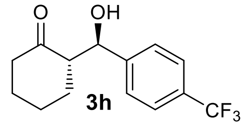

(*S*)-2-((*R*)-hydroxy(4-(trifluoromethyl)phenyl)methyl)-cyclohexan-1-one (**3h**): To a clean, screw cap, V-shaped reaction vessel (2.0 mL) equipped with a small pyramidal stir bar, 3-(nitromethyl)-1,2-dihydronaphthalene (**2c**) (MW = 189.21 g/mol, 1.50 equiv, 1.05 mmol, 199 mg), cyclohexanone (MW = 98.15 g/mol, 1.50 equiv, 1.05 mmol, 103 mg, 109 µL, density = 0.947 g/mL) and freshly purified (see Section 2) 4-(trifluoromethyl)benzaldehyde (MW = 174.12 g/mol, 1.00 equiv, 0.70 mmol, 122 mg, 95.5 µL, density = 1.275 g/mL) were added. This mixture was gently stirred for 5 min resulting in a yellow colored clear solution with no undissolved material. The stirring was terminated and the *trans*-4-(*tert*-butyldiphenylsilyloxy)-L-proline catalyst (MW = 369.54 g/mol, 2.5 mol%, 0.0175 mmol, 6.47 mg) was added to the reaction vessel. Within 30 sec after catalyst addition, distilled deoxygenated water (MW = 18.02 g/mol, 15.01 equiv, 10.54 mmol, 190 mg, 190 µL) was added with minimal disruption of the concentrated organic layer. The mixture was stirred such that the contents of the vessel did not splash up and against the walls, but the phase boundary was gently agitated. For observational purposes, the stirring was stopped after 5 min and two phases formed. No undissolved solids were noted, and the concentrated organic layer was transparent and yellow colored. At 15 min, 2 h, and 32 h (work-up) the concentrated organic layer remained the same as at 5 min. See Section 2 for the work-up procedure.

This compound was previously synthesized and characterized, see below.

Crude product ^{1}H NMR analysis (Figure S88, see below) allowed determination of the following diastereo- and chemoselectivity ratios.

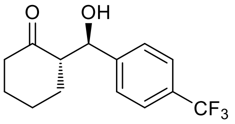

anti-aldol **3h** (major)

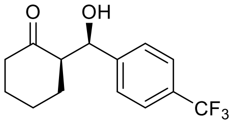

syn-aldol **4h** (minor)

Diastereoselectivity: The *anti*-aldol/*syn*-aldol product ratio was determined as 11.1:1 from the benzylic proton resonances at 4.85 ppm (d, *anti*-aldol product) and 5.45 ppm (bs, *syn*-aldol product) in the crude ^{1}H NMR spectrum (Figure S88). For literature regarding the *syn*-aldol product chemical shift, see page 14 of the Supporting Information within: Gao, J.; Bai, S.; Gao, Q.; Liu, Y.; Yang, Q. Acid Controlled Diastereoselectivity in Asymmetric Aldol Reaction of Cycloketones with Aldehydes using Enamine-Based Organocatalysts. *Chem. Commun.* **2011**, *47*, 6716–6718. For the chemical shift literature value for the *anti*-aldol product, see page S8 of the supporting information of: Al Beiruty, H.; Zhylinska, S.-S.; Kutateladze, N.; Cheong, H. K. T.; Ñíguez, J. A.; Burlingham, S. J.; Marset X.; Guillena, G.; Chinchilla, R.; Alonso, D. A.; Nugent, T. C. Enantioselective Catalytic Aldol Reactions in the Presence of Knoevenagel Nucleophiles: A Chemoselective Switch Optimized in Deep Eutectic Solvents Using Mechanochemistry. *Molecules* **2024**, *29*, 4. https://doi.org/10.3390/molecules29010004

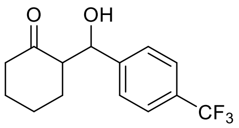

aldol products **3h**/**4h**

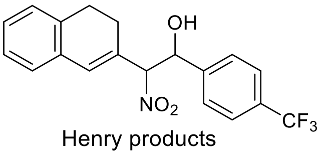

Henry products

Chemoselectivity: The *anti*- and *syn*-aldol/*anti*- and *syn*-Henry product ratio was determined to be 6.81:1, based on the benzylic proton resonances at 4.85 ppm (d, *anti*-aldol product) and 5.45 ppm (bs, *syn*-aldol product) to the benzylic proton resonances at 5.51 ppm (d, C**H**(Ph)OH, *syn*-Henry product) and 5.58 ppm (d, C**H**(Ph)OH, *anti*-Henry product) in the crude ^{1}H NMR spectrum (Figure S88). Note that the just assigned Henry products are not known in the literature and we did not synthesize them. However, our confidence in assigning those Henry resonance patterns comes from our comparison of them to the ^{1}H NMR of the related Henry products **5a** and **6a** whose ^{1}H NMRs can be found in Section 5 of this document.

Purification and yield: The crude product was loaded onto a silica gel (230-400 mesh) column (16 cm in height, 2 cm in diameter) pre-wetted with EtOAc/petroleum ether (1:19). The crude product was loaded onto the column in a minimum volume of CH_2Cl_2. The mobile phase elution began with EtOAc/petroleum ether (1:19). This solvent ratio was maintained until the starting materials were removed from the column. The polarity of the mobile phase was raised to EtOAc/petroleum ether (7:93) to remove the Henry products and the *syn*-aldol product. The *anti*-aldol product eluted after use of EtOAc/petroleum ether (1:9). Concentration of the pure fractions provided white solid (MW= 272.27 g/mol, 141 mg, 0.518 mmol, 74% yield) of the *anti*-aldol product **3h**.

R_f: 0.20 (*anti*-aldol product), EtOAc/petroleum ether (1:4).

99% *ee*: Chiralcel OD-H chiral HPLC column, iPrOH/n-hexane (20:80), flow rate = 0.5 mL/min, λ = 216 nm, injection volume = 20 µL, the sample was dissolved in 20 vol% iPrOH/n-hexane with a concentration of ≈ 1 mg/mL; *anti*-aldol product retention times: t_{major} = 11.1 min, t_{minor} = 13.2 min. (Figures S90 and S91).

^{1}H NMR (400 MHz, $CDCl_3$) (ppm) *anti*-aldol product **3h** (Figure S89): δ 7.61 (d, 2H, *J* = 8.3 Hz), 7.44 (d, 2H, *J* = 8.3 Hz), 4.84 (dd, 1H, *J* = 8.6, 2.9 Hz), 4.04 (d, 1H, *J* = 3.0 Hz), 2.65-2.55 (m, 1H), 2.53-2.45 (m, 1H), 2.42-2.30 (m, 1H), 2.11 (m, 1H), 1.81 (m, 1H), 1.73-1.47 (m, 2H), 1.41-1.30 (m, 1H).

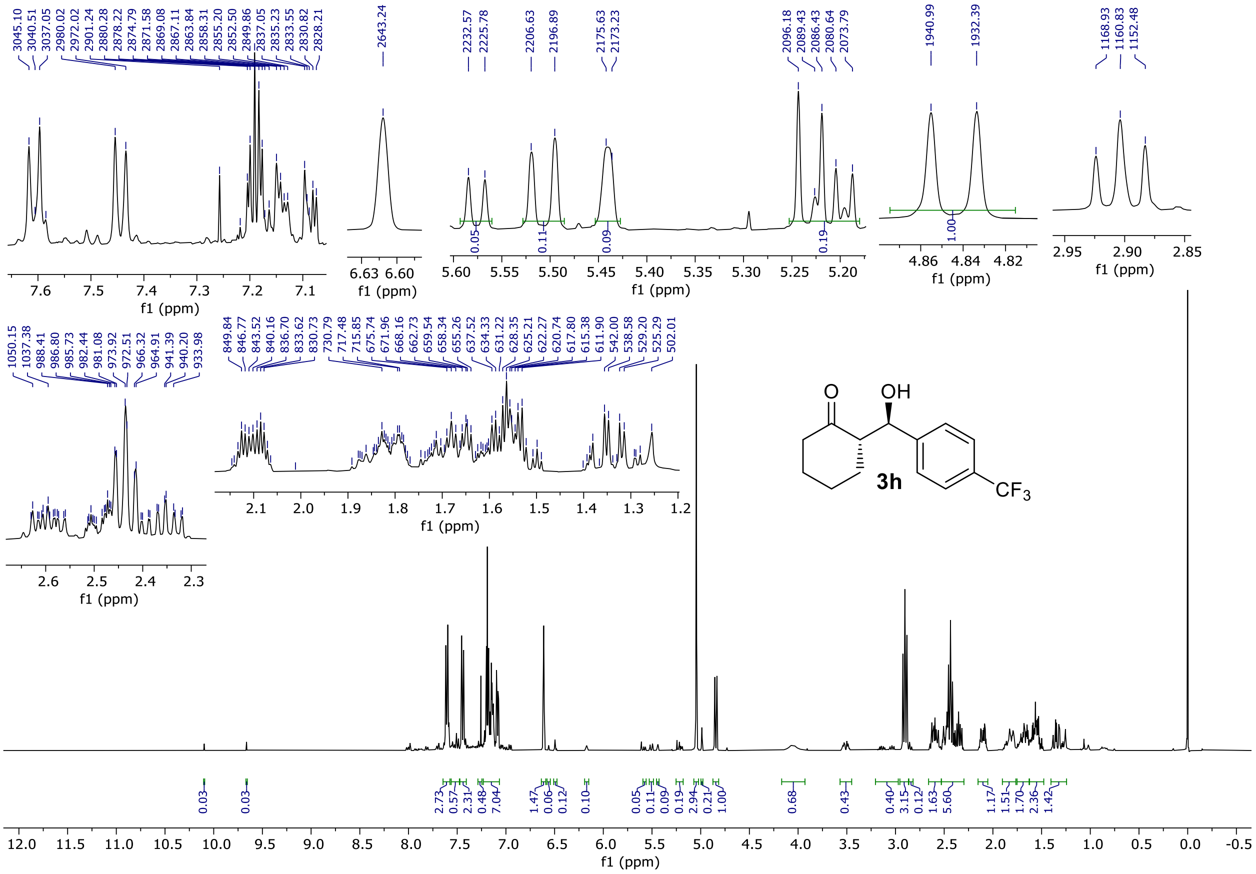

Figure S88. Crude ^{1}H NMR spectrum after high vacuum drying of **3h** (above).

Note that ^{1}H NMR data of crude product **3h**, after work-up and drying, of the reaction between cyclohexanone and 4-trifluoromethylbenzaldehyde was problematic. Each sample from the semi-solid product provided a different chemoselectivity. For that reason, the entire crude product was dissolved in $CDCl_3$ and only then did we record the ^{1}H NMR.

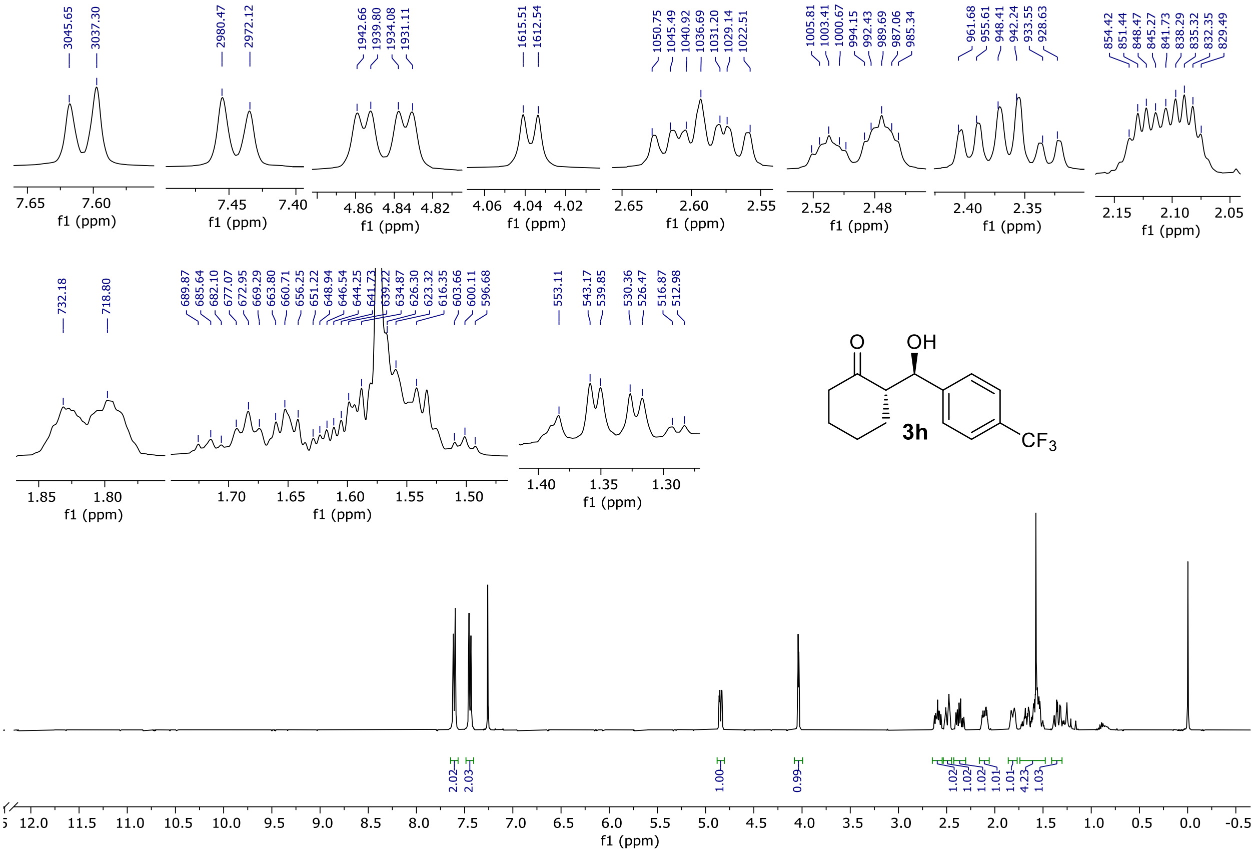

Figure S89. ^{1}H NMR spectrum of the purified *anti*-aldol (major) product **3h** (above).

SHIMADZU LabSolutions **Analysis Report**

<Sample Information>

Sample Name	: HC-I-26 20%IPAnHex 20uL 0.5 mLmin 08Feb23 1mgmL		
Sample ID	:		
Data Filename	: HC-I-26 20%IPAnHex 20uL 0.5 mLmin 08Feb23 1mgmL.lcd		
Method Filename	: trial.lcm		
Batch Filename	:		
Vial #	: 1-4	Sample Type	: Unknown
Injection Volume	: 20 uL		
Date Acquired	: 2/8/2023 3:15:20 PM	Acquired by	: System Administrator
Date Processed	: 2/8/2023 3:39:35 PM	Processed by	: System Administrator

<Chromatogram>

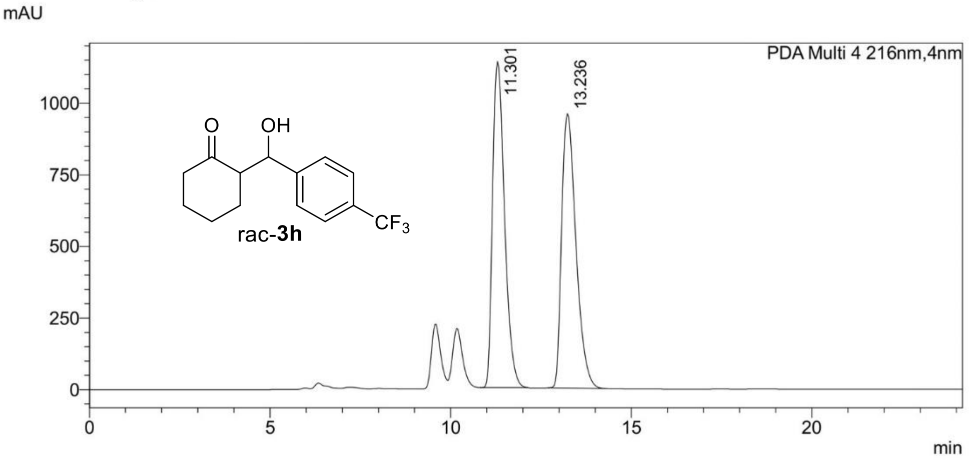

<Peak Table>

PDA Ch4 216nm

Peak#	Ret. Time	Area	Height	Area%
1	11.301	26277677	1138787	49.940
2	13.236	26341028	958736	50.060
Total		52618705	2097523	100.000

Figure S90. HPLC chromatogram of the racemic *anti*-aldol product **3h** (above).

Acq. Operator : Seq. Line : 1
Acq. Instrument : Instrument 1 Location : P1-A-05
Injection Date : 4/1/2025 11:11:57 AM Inj : 1
Inj Volume : 20 µl
Acq. Method : D:\Methods\Nugentlab_Patrick\PK 20IPA 216NM.m
Last changed : 4/1/2025 11:10:53 AM
Analysis Method : D:\Methods\Tevyashova\POLYENE 1 - VMM.m
Last changed : 4/1/2025 1:00:29 PM
(modified after loading)

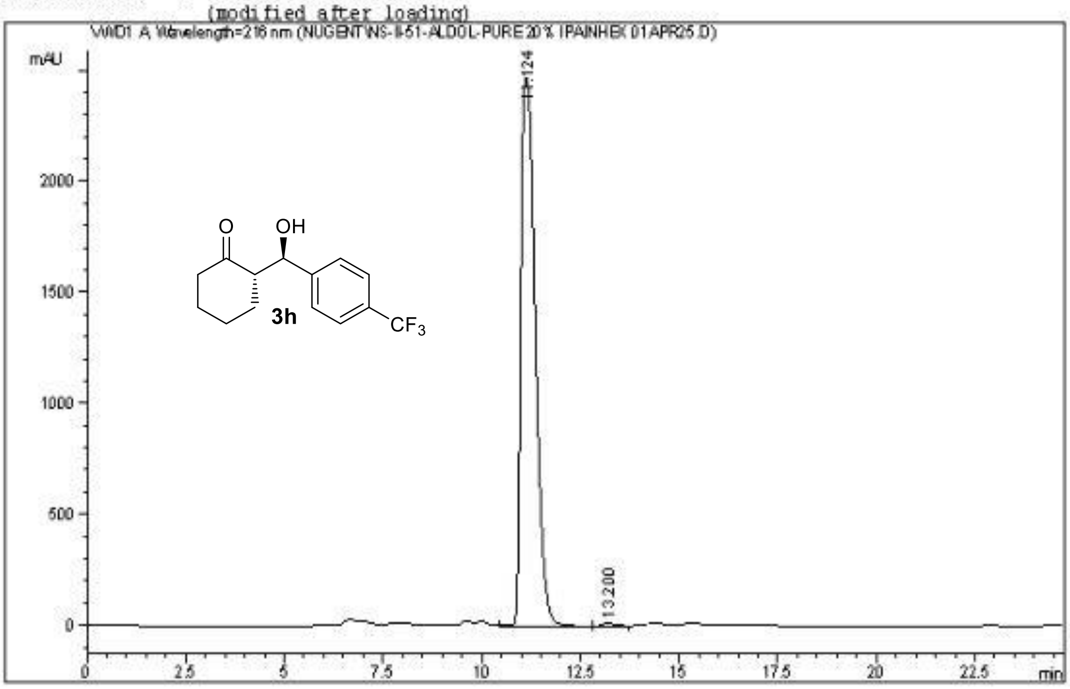

Area Percent Report

Sorted By : Signal
Multiplier : 1.0000
Dilution : 1.0000
Use Multiplier & Dilution Factor with ISTDs

Signal 1: VWD1 A, Wavelength=216 nm

Peak #	RetTime [min]	Type	Width [min]	Area mAU *s	Height [mAU]	Area %
1	11.124	VB	0.4004	6.28748e4	2469.52539	99.5222
2	13.200	BV	0.3631	301.86432	12.77106	0.4778
Totals :				6.31767e4	2482.29645	

Figure S91. HPLC chromatogram of enantioenriched *anti*-aldol (major) product **3h** (above).

Table 3, entry 7: Competition reaction between cyclohexanone and 7-bromo-3-(nitromethyl)-1,2-dihydronaphthalene (2d) for the limiting reactant 3-chlorobenzaldehyde

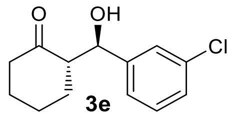

(*S*)-2-((*R*)-(3-chlorophenyl)(hydroxy)methyl)-cyclohexan-1-one (**3e**):
To a clean, screw cap, V-shaped reaction vessel (2.0 mL) equipped with a small pyramidal stir bar, mortar and pestle ground 7-bromo-3-(nitromethyl)-1,2-dihydronaphthalene (**2d**) (MW = 268.11 g/mol, 1.50 equiv, 1.05 mmol, 281.5 mg), cyclohexanone (MW = 98.15 g/mol, 1.50 equiv, 1.05 mmol, 103 mg, 109 µL, density = 0.947 g/mL) and freshly purified (see Section 2) 3-chlorobenzaldehyde (MW = 140.57 g/mol, 1.00 equiv, 0.70 mmol, 98 mg, 79 µL, density = 1.241 g/mL) were added. The liquid reactants (cyclohexanone and 3-chlorobenzaldhyde) were used to rinse the solid 7-bromo-3-(nitromethyl)-1,2-dihydronaphthalene off the walls as needed. This mixture was gently stirred for 5 min resulting in a yellowish solution with an estimated 50% of undissolved **2d**. The stirring was terminated and the *trans*-4-(*tert*-butyldiphenylsilyloxy)-L-proline catalyst (MW = 369.54 g/mol, 2.6 mol%, 0.0179 mmol, 6.6 mg) [2.6 mol%, instead of 2.5 mol%, was accidentally added to this reaction] was added to the reaction vessel. Within 30 sec after catalyst addition, distilled deoxygenated water (MW = 18.02 g/mol, 15.01 equiv, 10.54 mmol, 190 mg, 190 µL) was added with minimal disruption of the concentrated organic layer. The mixture was stirred such that the contents of the vessel did not splash up and against the walls, but the phase boundary was gently agitated. For observational purposes, the stirring was stopped after 5 min and two phases formed. The top aqueous phase was colorless and clear, whereas the concentrated organic layer was yellowish and contained ~50% undissolved solids. This was repeated at 20 min and within the bottom phase ~25% of finely divided **2d** remained as a solid. At further points in time: 1 h (bottom layer: ~15% undissolved, yellowish in color), 2.5 h (~10% undissolved, yellow) and 5 h (5% undissolved, intense yellow). After 32 h the stirring was terminated, revealing a biphasic system, the bottom layer was an intense yellow color and contained ~5% undissolved solids, whereas the top layer was clear and colorless. See Section 2 for the work-up procedure.

This compound was previously synthesized and characterized, see below.

Crude product ^{1}H NMR analysis (Figure S92, see below) allowed determination of the following diastereo- and chemoselectivity ratios.

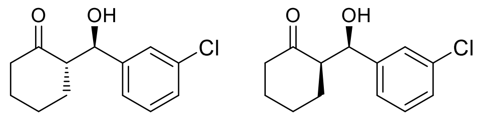

anti-aldol **3e** (major) *syn*-aldol **4e** (minor)

Diastereoselectivity: The *anti*-aldol/*syn*-aldol product ratio was determined as 14.3:1 from the benzylic proton resonances at 4.76 ppm (d, *anti*-aldol product) and 5.37 ppm (bs, *syn*-aldol product) in the crude ^{1}H NMR spectrum (Figure S92). For the chemical shift literature values of *anti*- and *syn*-aldol products, see page SI 6 of the supporting information of: Martínez-Castañeda, Á.; Rodríguez-Solla, H.; Concellón, C.; del Amo, V. TBD/Al_2O_3: A Novel Catalytic System for Dynamic Intermolecular Aldol Reactions that Exhibit Complex System Behaviour. *Org. Biomol. Chem.* **2012**, *10*, 1976-1981.

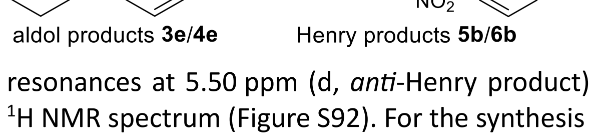

aldol products **3e**/**4e** Henry products **5b**/**6b**

Chemoselectivity: The *anti*- and *syn*-aldol/*anti*- and *syn*-Henry product ratio was determined to be 4.9:1 from the benzylic proton resonances at 4.76 ppm (d, *anti*-aldol product) and 5.37 ppm (bs, *syn*-aldol product) compared to the benzylic proton resonances at 5.50 ppm (d, *anti*-Henry product) and 5.41 ppm (d, *syn*-Henry product) in the crude ^{1}H NMR spectrum (Figure S92). For the synthesis and characterization of the Henry product reference standards **5b** and **6b**, see Section 5 of this document.

Purification and yield: Silica gel chromatography (32mm column outer diameter, 18 cm silica bed height) was performed using isocratic elution (15 vol% ethyl acetate in petroleum ether). The crude product was loaded onto the column in a minimum volume of CH_2Cl_2. The *anti*-aldol product was isolated as an off-white solid (111 mg, MW = 238.71 g/mol, 0.465 mmol, 66% yield).

R_f: 0.17 (*anti*-aldol product) (EtOAc/petroleum ether, 15:85).

99% *ee*: Chiralcel OD-H chiral HPLC column, iPrOH/n-hexane (5:95), flow rate = 1.0 mL/min, λ = 210 nm, injection volume = 20 µL, the sample was dissolved in 5 vol% iPrOH/n-hexane with a concentration of ≈ 1 mg/mL; *anti*-aldol product retention times: t_{major} = 11.2 min, t_{minor} = 16.2 min. (Figures S94 and S95).

^{1}H NMR (400 MHz, $CDCl_3$) (ppm) *anti*-aldol product **3e** (Figure S93): δ 7.35-7.33 (m, 1H), 7.29-7.26 (m, 2H), 7.22-7.17 (m, 1H), 4.76 (dd, 1H, *J* = 8.8, 2.9 Hz), 4.02 (d, 1H, *J* = 2.7 Hz), 2.62-2.54 (m, 1H), 2.52-2.45 (m, 1H), 2.41-2.31 (m, 1H), 2.15-2.07 (m, 1H), 1.85-1.77 (m, 1H), 1.73-1.50 (m, 3H), 1.37-1.25 (m, 1H).

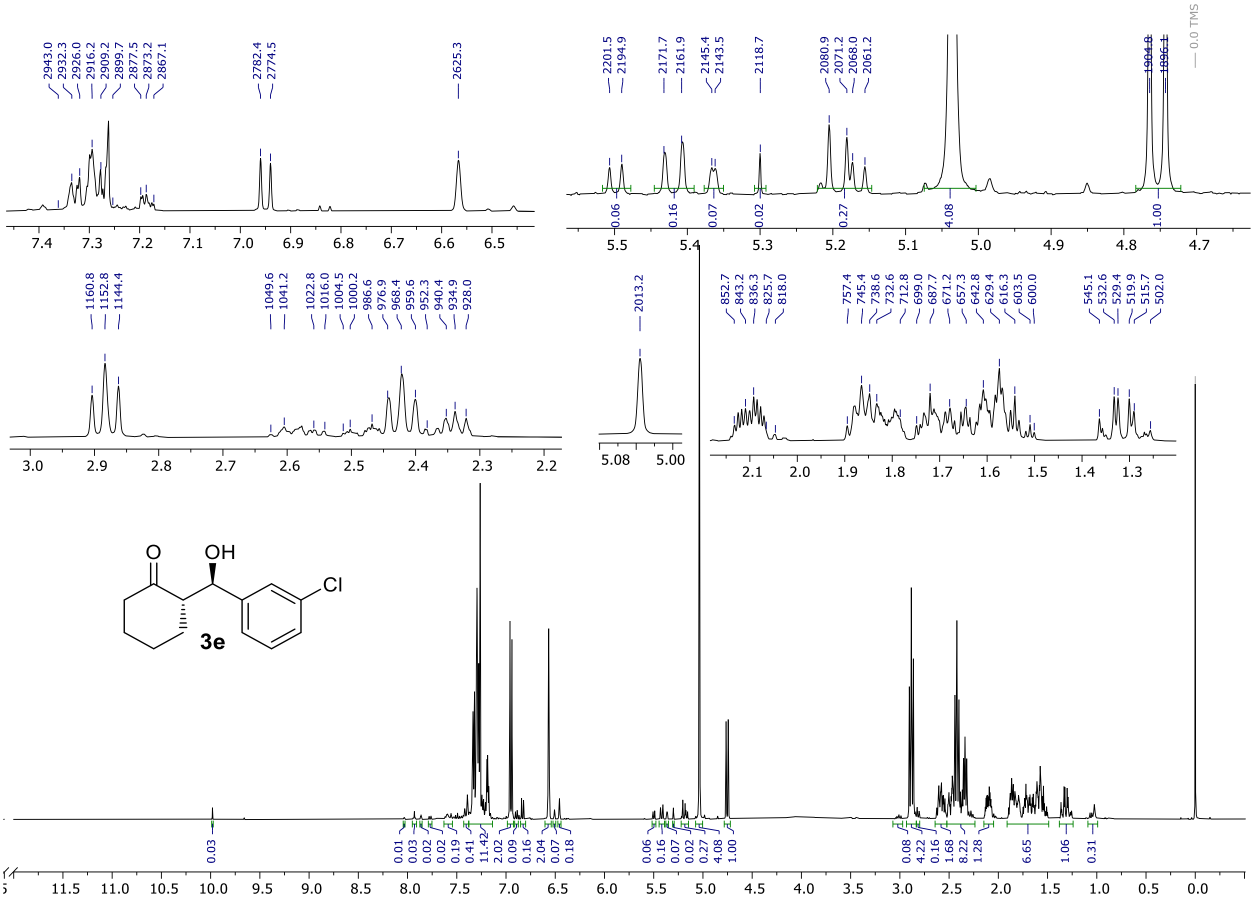

Figure S92. Crude ^{1}H NMR spectrum after high vacuum drying of **3e** (above).

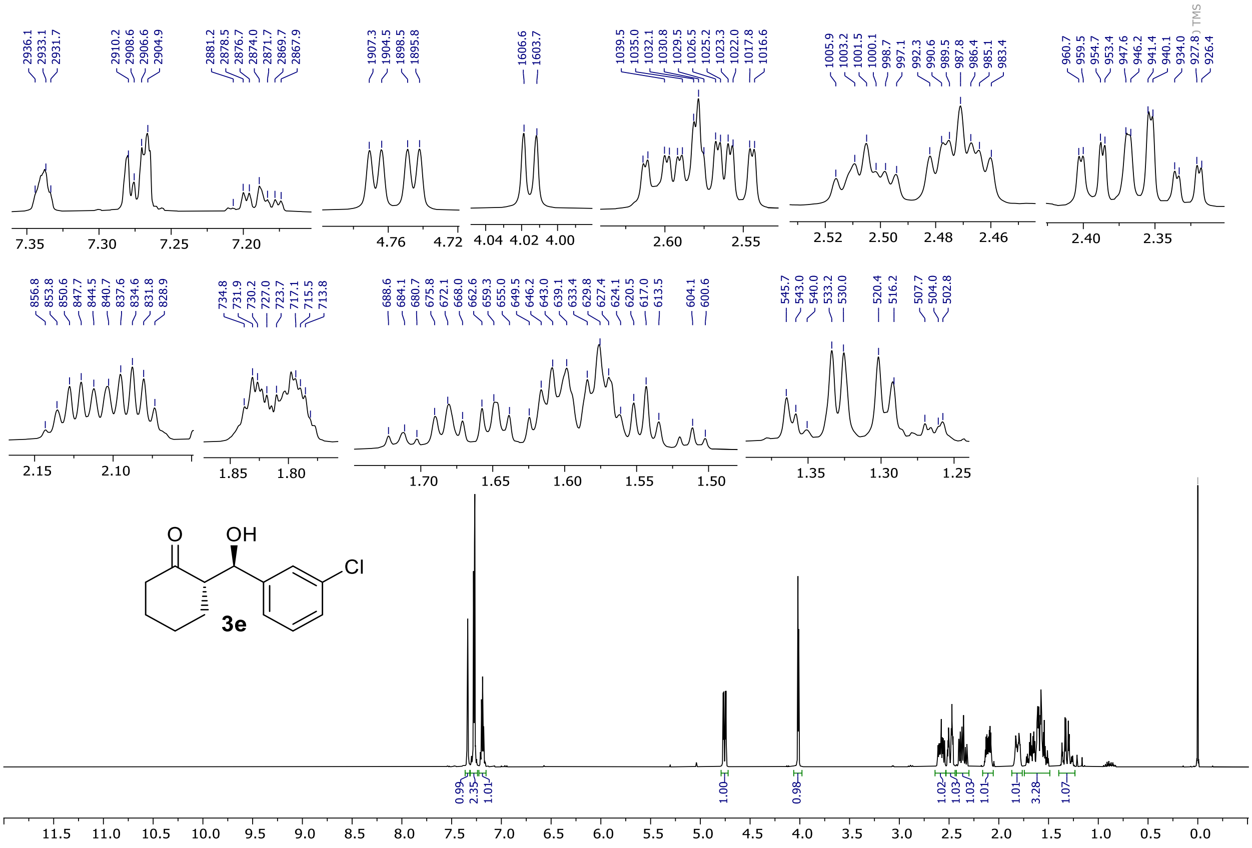

Figure S93. ^{1}H NMR spectrum of purified *anti*-aldol (major) product **3e** (above).

Data File D:\DATA\NUGENT\AL24 RAC 5%IPANHEX 06FEB25 RE.D
Sample Name: AL24 RAC 5%IPAnHex 06feb25 re

===

Acq. Operator   :   Seq. Line :   2
Acq. Instrument : Instrument 1   Location : P1-A-03
Injection Date : 2/6/2025 1:35:39 PM   Inj :   1
                                                Inj Volume : 20 µl
Acq. Method   : D:\Methods\Nugentlab_Patrick\AIDA.m
Last changed   : 2/6/2025 1:34:31 PM
Analysis Method : D:\Methods\Nugentlab_Patrick\AIDA254NM1.m
Last changed   : 2/6/2025 2:01:03 PM
                  (modified after loading)

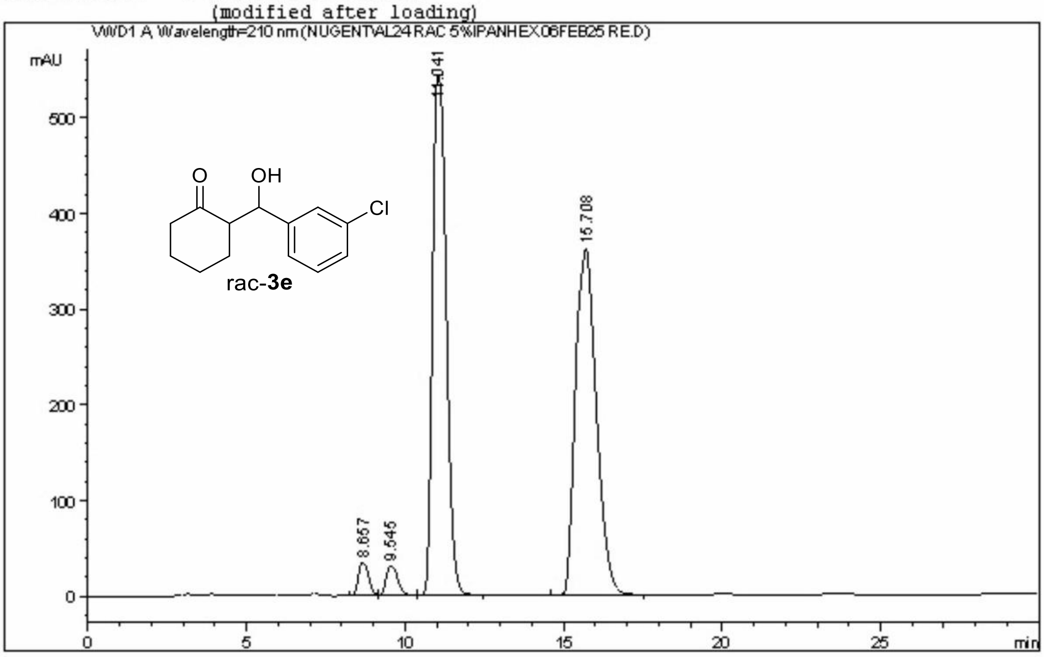

===

Area Percent Report

===

Sorted By : Signal
Multiplier : 1.0000
Dilution : 1.0000
Use Multiplier & Dilution Factor with ISTDs

Signal 1: VWD1 A, Wavelength=210 nm

Peak #	RetTime [min]	Type	Width [min]	Area mAU *s	Height [mAU]	Area %
1	8.657	BV	0.3643	785.40881	34.54132	2.2828
2	9.545	VB	0.4170	804.99719	30.80102	2.3397
3	11.041	BB	0.4747	1.62915e4	543.91724	47.3515
4	15.708	BB	0.7207	1.65235e4	361.77737	48.0260

Totals : 3.44054e4 971.03695

===

*** End of Report ***

Instrument 1 2/6/2025 2:06:54 PM

Page 1 of 1

Figure S94. HPLC chromatogram of the racemic *anti*-aldol product **3e** (above).

```
Data File D:\DATA\NUGENT\PK 028 ENANTIOENR 5% IPAINHEX 14FEB25.D
Sample Name: PK 028 ENANTIOENR 5% IPAinHEX 14FEB25
=====================================================================
Acq. Operator   :                               Seq. Line :   2
Acq. Instrument : Instrument 1                   Location : P1-A-02
Injection Date  : 2/14/2025 12:59:39 PM               Inj :   1
                                               Inj Volume : 20 µl
Acq. Method     : D:\Methods\Nugentlab Patrick\AIDA.m
Last changed    : 2/14/2025 12:58:37 PM
Analysis Method : D:\Methods\Nugentlab_Patrick\AIDA.m
Last changed    : 2/5/2025 12:02:12 PM
=====================================================================
```

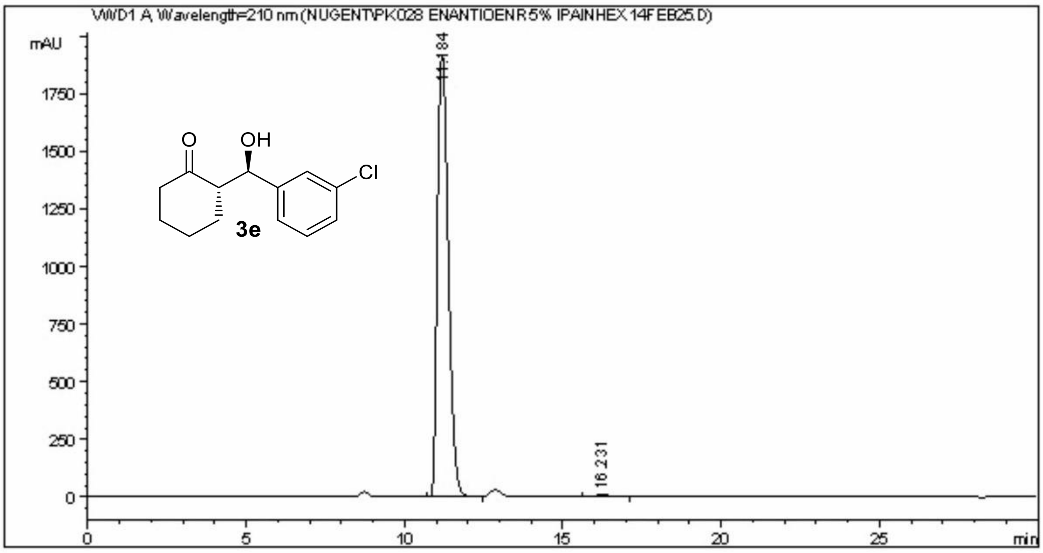


```
=====================================================================
                         Area Percent Report
=====================================================================

Sorted By             :      Signal
Multiplier            :      1.0000
Dilution              :      1.0000
Use Multiplier & Dilution Factor with ISTDs


Signal 1: VWD1 A, Wavelength=210 nm

Peak RetTime Type  Width     Area      Height     Area
  #   [min]        [min]  mAU   *s   [mAU   ]      %
----|-------|----|-------|----------|----------|--------|
   1  11.184 BV    0.3797 4.61714e4  1919.07544  99.3082
   2  16.231 VB    0.5033  321.63947   10.15862   0.6918

Totals :                  4.64930e4  1929.23406


=====================================================================
                          *** End of Report ***
```

Instrument 1 2/14/2025 4:31:39 PM   Page 1 of 1

Figure S95. HPLC chromatogram of enantioenriched *anti*-aldol (major) product **3e** (above).

Table 3, entry 10: Competition reaction between cyclohexanone and (nitromethyl)benzene (2f) for the limiting reactant 4-nitrobenzaldehyde

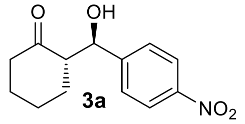

(*S*)-2-((*R*)-hydroxy(4-nitrophenyl)methyl)cyclohexan-1-one (**3a**):
To a clean, screw cap, V-shaped reaction vessel (5.0 mL) equipped with a small pyramidal stir bar, mortar and pestle ground 4-nitrobenzaldehyde (MW = 151.12 g/mol, 1.00 equiv, 1.00 mmol, 151.1 mg), cyclohexanone (MW = 98.14 g/mol, 1.50 equiv, 1.50 mmol, 147.2 mg, 155 µL, density = 0.947 g/mL), and (nitromethyl)benzene (**2f**) (MW = 137.14 g/mol, 1.50 equiv, 1.50 mmol, 205.7 mg, 178 µL, density = 1.158 g/mL) were added. The liquid reactants (cyclohexanone and (nitromethyl)benzene) were used to rinse the solid 4-nitrobenzaldehyde off the walls as needed. The heterogeneous mixture was allowed to gently stir for < 5 min, but visual inspection showed little or no dissolution of the 4-nitrobenzaldehyde. Next, the stirring was terminated and *trans*-4-(tert-butyldiphenylsilyloxy)-L-proline (MW = 369.54 g/mol, 2.50 mol%, 0.0250 mmol, 9.2 mg) was added. Distilled deoxygenated water (MW = 18.02 g/mol, 15.0 equiv, 15.0 mmol, 270.3 mg, 270 µL) was added within 30 sec. The resulting heterogeneous solution was vigorously stirred for 24 h such that the contents of the vessel did not splash against the vessel walls but allowed gentle phase boundary agitation. Within approximately 1.5 h, the solids fully dissolved in the concentrated organic phase, but for the entirety of the reaction, 24 h, an emulsion was noted. For observational purposes, the stirring was occasionally stopped for 2 min and two-phase boundaries were always noted. See Section 2 for the work-up procedure.

This compound was previously synthesized and characterized, see below.

Crude product ^{1}H NMR analysis (Figure S95, see below) allowed determination of the diastereo- and chemoselectivity ratios.

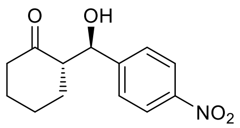

anti-aldol **3a** (major)

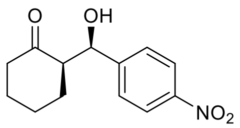

syn-aldol **4a** (minor)

Diastereoselectivity: The *anti*-aldol/*syn*-aldol ratio was determined as 14.3:1 based on the integration of the benzylic proton resonances at 4.89 ppm (d, *anti*-aldol product) and 5.48 ppm (bs, *syn*-aldol product) in the crude ^{1}H NMR spectrum (Figure S95). For the aldol literature chemical shift values, see page SI 5 (product 3e) of the Supporting Information within: Martínez-Castañeda, Á.; Rodríguez-Solla, H.; Concellón, C.; del Amo, V. TBD/Al_2O_3: A Novel Catalytic System for Dynamic Intermolecular Aldol Reactions that Exhibit Complex System Behaviour. *Org. Biomol. Chem.* **2012**, *10*, 1976-1981.

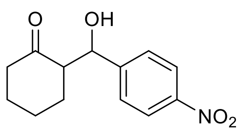

aldol products **3a**/**4a**

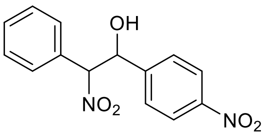

Henry products **5c**/**6c**

Chemoselectivity: The *anti*- and *syn*-aldol/*anti*- and *syn*-Henry product ratio was determined to be 5.4:1, based on the integration of the benzylic proton resonances at 4.89 ppm (d, *anti*-aldol product) and 5.48 ppm (bs, *syn*-aldol product) compared to the benzylic proton resonances at 5.74 ppm (d, C**H**(Ph)OH, *anti*-Henry product) and 5.71 ppm (d, C**H**(Ph)OH, *syn*-Henry product) in the crude ^{1}H NMR spectrum (Figure S95). For the synthesis and characterization of the Henry product reference standards **5c** and **6c**, see Section 5 of this document.

Purification and yield: Silica gel chromatography (25 mm column outer diameter, 18 cm silica bed height) was performed using gradient elution (10 to 25 vol% EtOAc in petroleum ether). The crude product was loaded onto the column in a minimum volume of 50% EtOAc in petroleum ether. A yellow solid weighing 185.8 mg (MW = 249.27 g/mol, 0.745 mmol, 75% yield) of the *anti*- and *syn*-aldol diastereomers were isolated.

R_f: *anti*-aldol product = 0.23, *syn*-aldol product = 0.30 (EtOAc/petroleum ether, 3:7).

99% *ee*: Chiralcel OD-H chiral HPLC column, *i*PrOH/n-hexane (7:93), flow rate = 1.0 mL/min, λ = 254 nm, injection volume = 20 μL, the sample was dissolved in 10 vol% *i*PrOH/n-hexane with a concentration of ≈ 1 mg/mL; *anti*-aldol product retention times: t_{major} = 20.8 min, t_{minor} = 31.8 min (Figures S97 and S98).

^{1}H NMR (400 MHz, $CDCl_3$) (ppm) *anti*-aldol product **3a** (Figure S96): δ 8.21 (d, 2H, J = 8.8 Hz), 7.50 (d, 2H, J = 8.7 Hz), 4.89 (dd, 1H, J = 8.5, 2.9 Hz), 4.08 (d, 1H, J = 3.1 Hz), 2.63-2.54 (m, 1H), 2.53-2.45 (m, 1H), 2.41-2.31 (m, 1H), 2.16-2.06 (m, 1H), 1.89-1.77 (m, 1H), 1.76-1.48 (m, 4H), 1.44-1.30 (m, 1H).

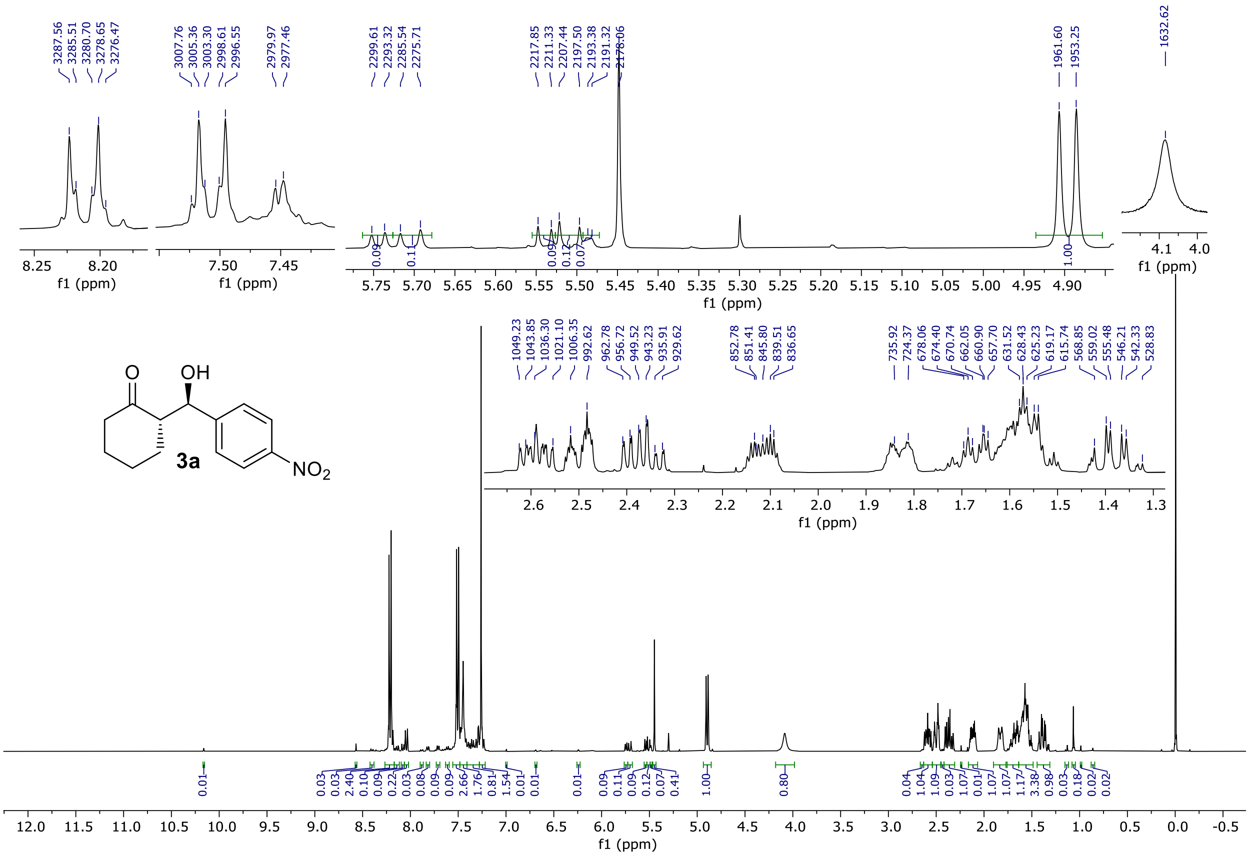

Figure S95. Crude ^{1}H NMR spectrum after high vacuum drying of **3a** (above).

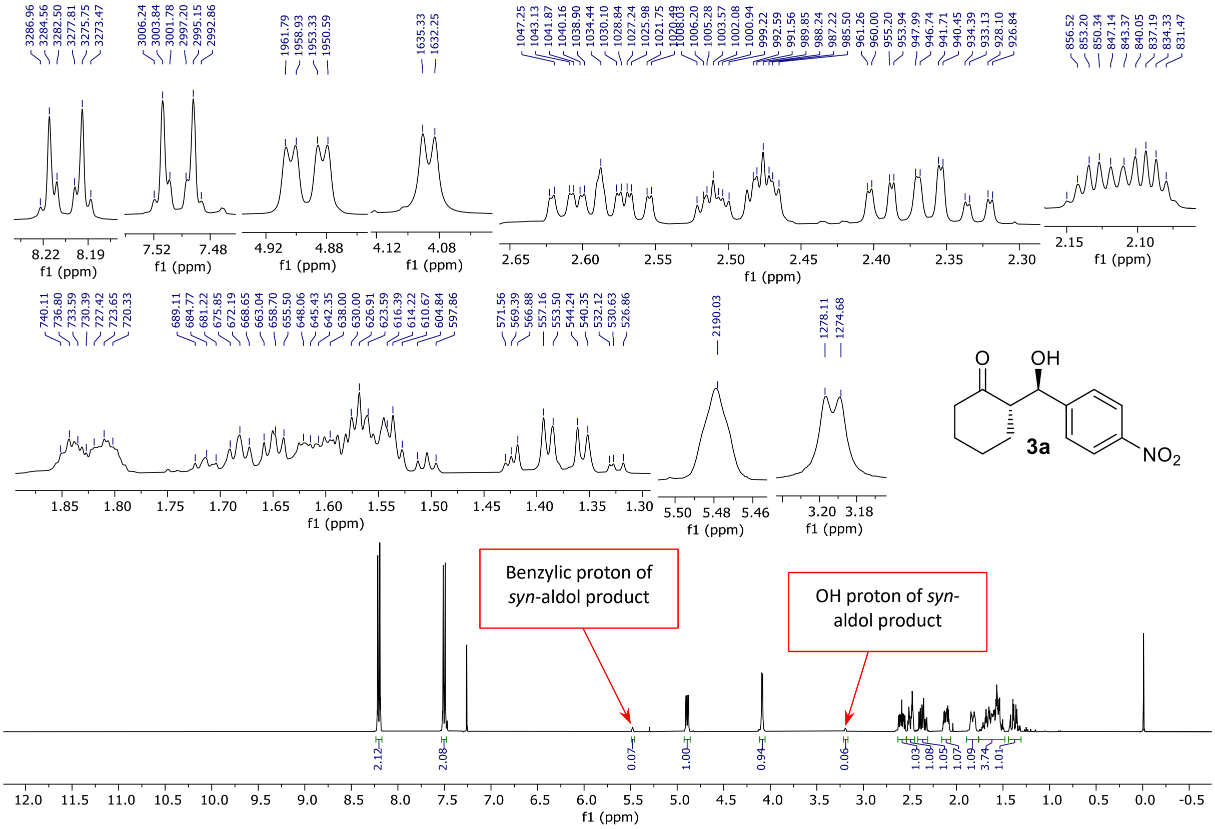

Figure S96. ^{1}H NMR spectrum of purified *anti*-(major) **3a** and *syn*-(minor) aldol product **4a** (above). The red arrows indicate the *syn*-aldol product.

Epimerization of aldol product 3a: Aldol product diastereomers of this structural category are prone to epimerization on heating or on exposure to silica gel. Consequently, crude aldol product ^{1}H NMRs can show higher dr values than ^{1}H NMRs of the chromatographed aldol products. This, in large part, is why researchers in this area decided long ago to isolate the *anti*-/*syn*-aldol products together and report the total yield. For this study, we decided to isolate only the *anti*-aldol products and record their yield, and that was possible for all products except *anti*-aldol product **3a**. Unlike the other aldol products we synthesized here, the *anti*-/*syn*-aldol products **3a**/**4a** have very similar R_f values and do epimerize during chromatography. This combination did not allow us to successfully isolate *anti*-aldol product **3a** free from the *syn*-aldol product **4a**. For this reason, researchers choose to evaluate aldol diastereoselectivity using crude ^{1}H NMR spectrums. For related material, see page S4 within the Supporting Information of: Nugent, T.C.; Umar, M. N.; Bibi, A. Picolylamine as an Organocatalyst Template for Highly Diastereo- and Enantioselective Aqueous Aldol Reactions, *Org. Biomol. Chem.* **2010**, *8*, 4085-4089.

SHIMADZU LabSolutions **Analysis Report**

ADV-A45-7% IPA/nHex 5uL 1mL/min 12May2022

Sample Name	: A46 (RACE)7% IPAnHex 5uL1mLmin 26May 2		
Sample ID	:		
Data Filename	: A46 (RACE)7% IPAnHex 5uL1mLmin 26May 2.lcd		
Method Filename	: trial.lcm		
Batch Filename	:		
Vial #	: 1-1	Sample Type	: Unknown
Injection Volume	: 20 uL		
Date Acquired	: 5/26/2022 2:38:31 PM	Acquired by	: System Administrator
Date Processed	: 5/26/2022 3:28:36 PM	Processed by	: System Administrator

ADV-A46-7% IPA/nHex 5uL 1mL/min 12May2022

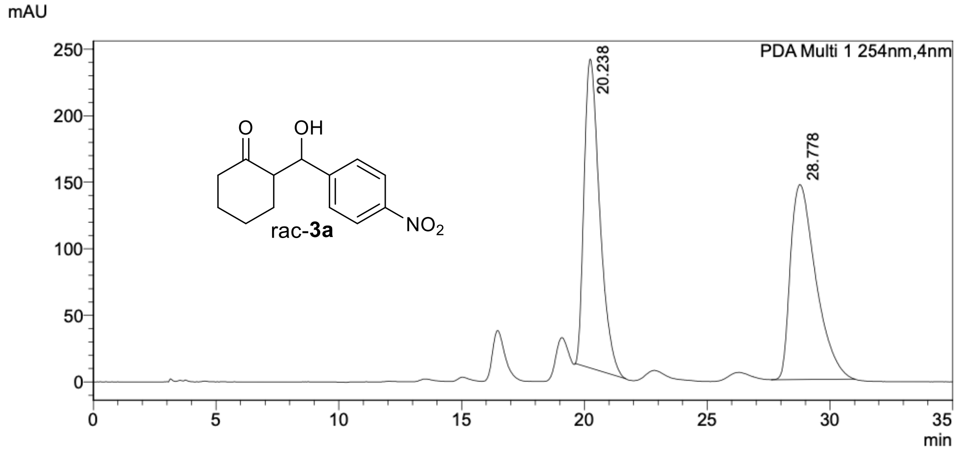

ADV-A45-7% IPA/nHex 5uL 1mL/min 12May2022

PDA Ch1 254nm

Peak#	Ret. Time	Area	Height	Area%
1	20.238	10283216	232276	49.316
2	28.778	10568658	146536	50.684
Total		20851874	378812	100.000

C:\Users\Shimadzu\Desktop\ADV\Competition aldol samples\A46 (RACE)7% IPAnHex 5uL1mLmin 26May 2.lcd

Figure S97. HPLC chromatogram of the racemic *anti*-aldol product **3a** (above).

SHIMADZU LabSolutions Analysis Report

<Sample Information>

Sample Name	: RL20-IV-7%IPANHEX-1000PPM-SAM[10%IPANHEX]		
Data Filename	: RL20-IV-7%IPANHEX-1000PPM-SAM[10%IPANHEX]001.lcd		
Method Filename	: RL1-7%IPANHEX-1000PPM-SAM[10%IPANHEX].lcm		
Vial #	: 1-12	Sample Type	: Unknown
Injection Volume	: 20 uL		
Date Acquired	: 11/3/2024 2:11:00 PM	Acquired by	: System Administrator
Date Processed	: 11/3/2024 2:51:03 PM	Processed by	: System Administrator

<Chromatogram>

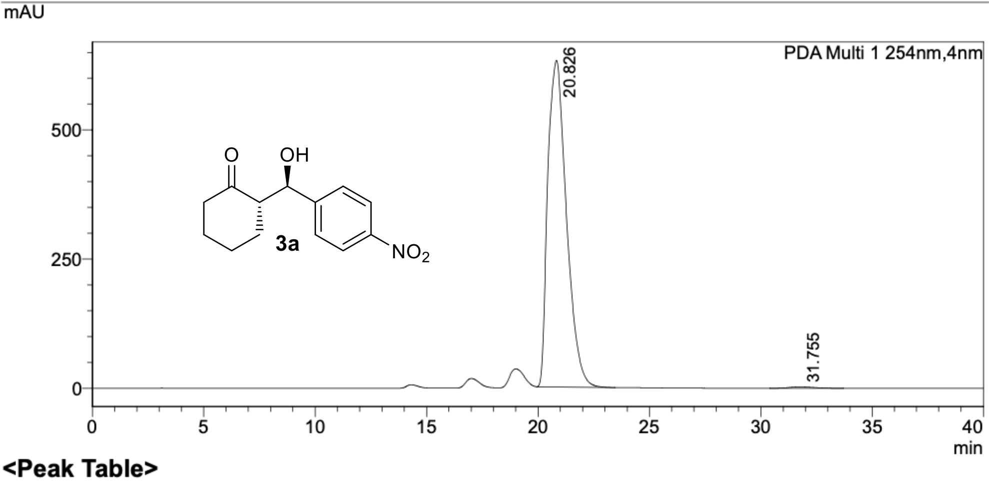

<Peak Table>

PDA Ch1 254nm

Peak#	Ret. Time	Area	Height	Area%
1	20.826	36771667	631899	99.422
2	31.755	213730	2766	0.578
Total		36985397	634666	100.000

C:\LabSolutions\Data\Project1\Data\RUSLAN\RL20-IV-7%IPANHEX-1000PPM-SAM[10%IPANHEX]001.lcd

Figure S98. HPLC chromatogram of the enantioenriched *anti*-aldol product **3a**.

Table 3, entry 11: Competition reaction between cyclohexanone and ethyl nitroacetate (2g) for the limiting reactant 3-chlorobenzaldehyde

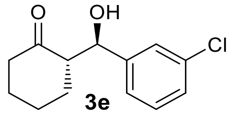

(*S*)-2-((*R*)-(3-chlorophenyl)(hydroxy)methyl)-cyclohexan-1-one (**3e**):
To a clean, screw cap, V-shaped reaction vessel (2.0 mL) equipped with a small pyramidal stir bar, ethyl nitroacetate (**2g**) (MW = 133.1 g/mol, 1.50 equiv, 1.50 mmol, 200 mg, density = 1.199 g/mL), cyclohexanone (MW = 98.15 g/mol, 1.50 equiv, 1.50 mmol, 147 mg, 155 µL, density = 0.947 g/mL) and freshly purified (see Section 2) 3-chlorobenzaldehyde (MW = 140.57 g/mol, 1.00 equiv, 1.0 mmol, 140.57 mg, 113 µL, density = 1.241 g/mL) were added. All reactants are liquids and formed a clear yellow solution. The stirring was terminated and the *trans*-4-(*tert*-butyldiphenylsilyloxy)-L-proline catalyst (MW = 369.54 g/mol, 2.5 mol%, 0.025 mmol, 9.24 mg) was added to the reaction vessel. Within 30 sec after catalyst addition, distilled deoxygenated water (MW = 18.02 g/mol, 15.01 equiv, 15.01 mmol, 270 mg, 270 µL) was added with minimal disruption of the concentrated organic layer. The mixture was stirred such that the contents of the vessel did not splash up and against the walls, but the phase boundary was always gently agitated. For observational purposes, the stirring was stopped after 5 min and two phases formed. No undissolved solids were noted and the organic phase was transparent and yellow colored. After 15 min the stirring was again stopped for better observation and the organic phase was now a suspension. At 2 h, the organic phase became fully transparent with no undissolved material and remained so until the end of reaction (32 h). See Section 2 for the work-up procedure.

This compound was previously synthesized and characterized, see below.

Crude product ^{1}H NMR analysis (Figure S99, see below) allowed an estimation of the diastereoselectivity but the chemoselectivity could not be determined, see comments below.

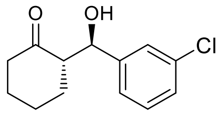

anti-aldol **3e** (major)

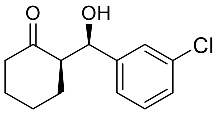

syn-aldol **4e** (minor)

Diastereoselectivity: The *anti*-aldol/*syn*-aldol product ratio was estimated >11:1 based on the benzylic proton resonances at 4.76 ppm (d, *anti*-aldol product) and 5.37 ppm (bs, *syn*-aldol product) in the crude ^{1}H NMR spectrum (Figure S99). In this instance another resonance pattern overlaps with the benzylic *anti*-aldol resonance pattern and may account for up to 33% of the integration area of 1.15 (see expansion 4.6 to 4.8 ppm). If true, then the minimum *dr* would be (1.15 x 0.33)/0.07 = 11.0/1.0. For the chemical shift literature values of *anti*- and *syn*-aldol products, see page SI 6 of the supporting information of: Martínez-Castañeda, Á.; Rodríguez-Solla, H.; Concellón, C.; del Amo, V. TBD/Al_2O_3: A Novel Catalytic System for Dynamic Intermolecular Aldol Reactions that Exhibit Complex System Behaviour. *Org. Biomol. Chem.* **2012**, *10*, 1976-1981.

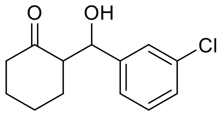

aldol products **3e**/**4e**

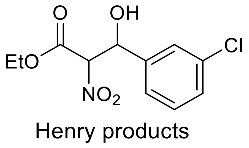

Henry products

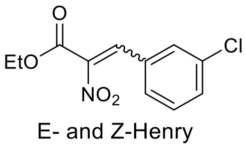

E- and Z-Henry condensation products

Chemoselectivity: No clear conclusion could be made from the crude product ^{1}H NMR (Figures S99, S101, and S102). The crude ^{1}H NMR includes resonances with chemical shifts typical of Henry products, -C**H**(NO_2)-C**H**(OH)-, between 5.2 to 5.6 ppm (see the expansion in Figure S99) albeit as minor products. However, the Henry product that can form here may not have a typical -C**H**(NO_2)- chemical shift because an ester substituent is bonded to the carbon holding the nitro group. Distinct from the other crude ^{1}H NMRs in this study and not attributable to the starting materials are the resonances between 5.7 and 5.9 ppm (see the expansion in Figure S99). However, a lack of coupling constant connectivity within that set of resonances did not allow us to suggest a Henry product.

To aid our understanding of what Henry products may have formed, we performed the Table 3, entry 11 competition reaction albeit without cyclohexanone (no experimental description provided). The crude ^{1}H NMR therefrom (Figure S101) contains the same resonances between 5.2 and 5.6 ppm and those between 5.7 and 5.9 ppm. However, the major product has distinct resonance patterns at 4.36 to 4.50 ppm. Unfortunately, only the major product could be isolated from that reaction and it appears to be a mixture of E- and Z-Henry condensation products (Figure S102). Interestingly, this condensation product is not observed in the crude ^{1}H NMR (Figure S99) of the competition reaction.

Purification and yield: The crude product was loaded onto a silica gel (230-400 mesh) column (16 cm in height, 2.0 cm in diameter) pre-wetted with EtOAc/petroleum ether (1:19). The crude product was loaded onto the column in a minimum volume of CH_2Cl_2. The mobile phase elution began with EtOAc/petroleum ether (1:19). This solvent ratio was maintained until the starting materials were removed from the column. The *anti*-aldol product eluted after use of EtOAc/petroleum ether (1:9). Concentration of the pure fractions provided white solid (MW= 238.71 g/mol, 143 mg, 0.60 mmol, 60% yield) of the *anti*-aldol product **3e**.

R_f: 0.17 (*anti*-aldol product), EtOAc/petroleum ether (15:85).

99% *ee*: Chiralcel OD-H chiral HPLC column, iPrOH/n-hexane (5:95), flow rate = 1.0 mL/min, λ = 210 nm, injection volume = 12 µL, the sample was dissolved in 5 vol% iPrOH/n-hexane with a concentration of ≈ 1 mg/mL; *anti*-aldol product retention times: t_{major} = 11.1 min, t_{minor} = 16.0 min. (Figures S103 and S104).

^{1}H NMR (400 MHz, $CDCl_3$) (ppm) *anti*-aldol product **3e** (Figure S100): δ 7.34 (s, 1H), 7.30-7.23 (m, 2H), 7.19 (m, 1H), 4.75 (dd, 1H, J = 8.7, 2.9 Hz), 4.00 (d, 1H, J = 2.7 Hz), 2.62-2.53 (m, 1H), 2.52-2.44 (m, 1H), 2.41-2.30 (m, 1H), 2.10 (m, 1H), 1.86-1.76 (m, 1H), 1.74-1.48 (m, 4H), 1.31 (m, 1H).

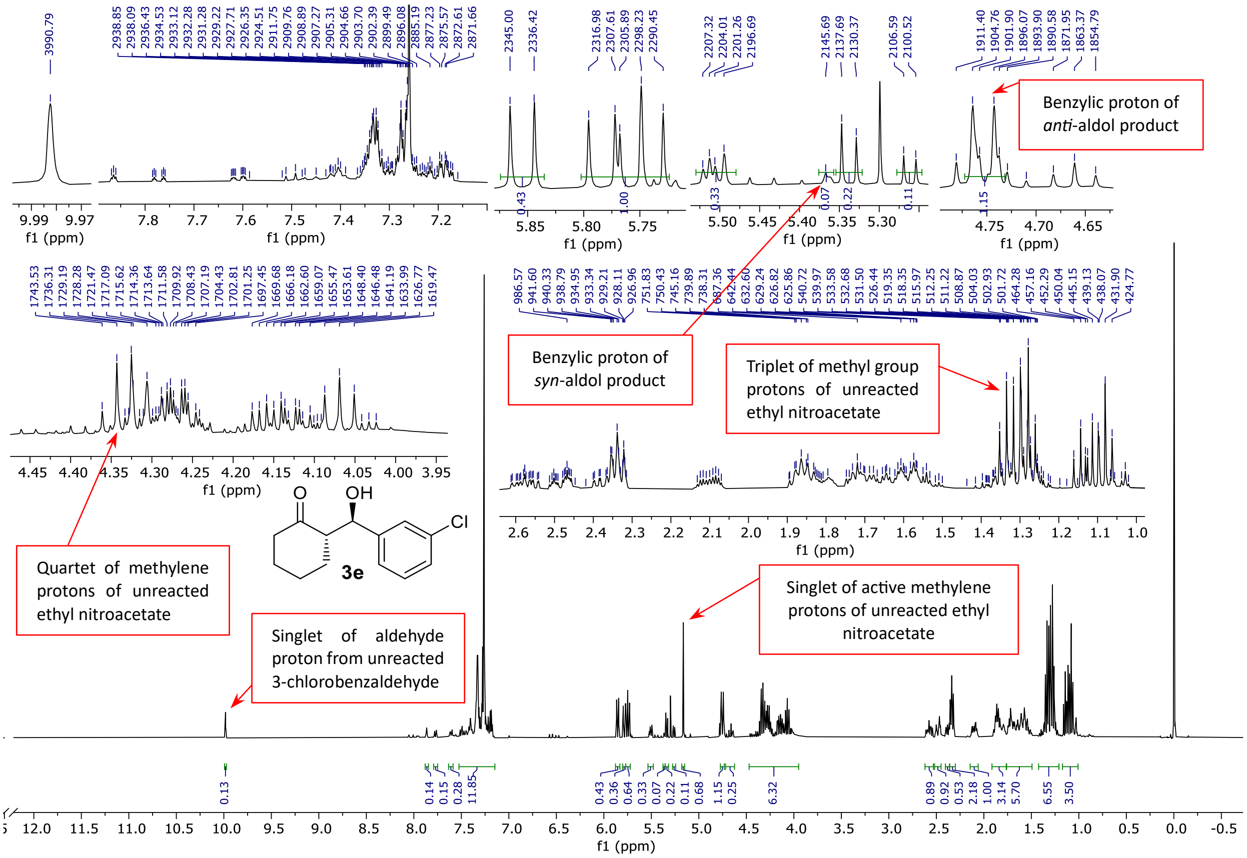

Figure S99. Crude ^{1}H NMR spectrum after high vacuum drying of **3e** (above).

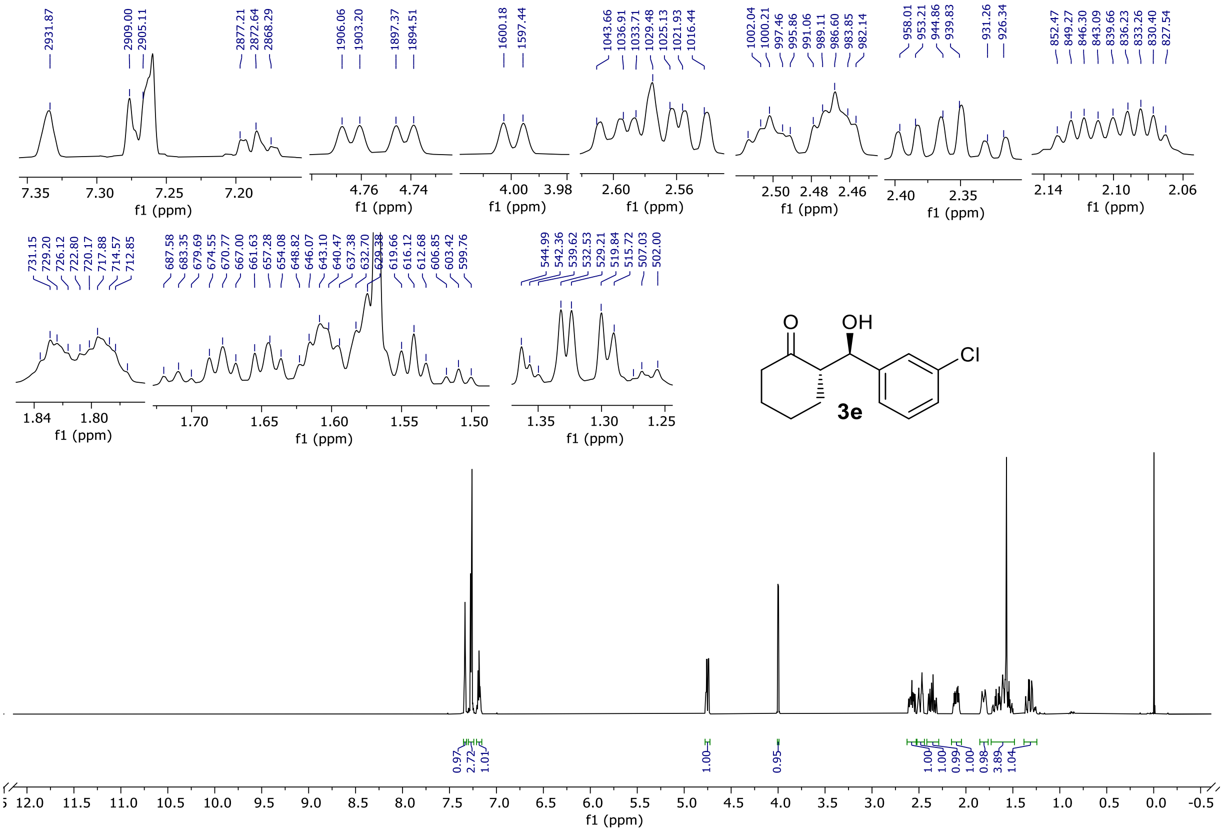

Figure S100. ^{1}H NMR spectrum of purified *anti*-aldol (major) product **3e** (above).

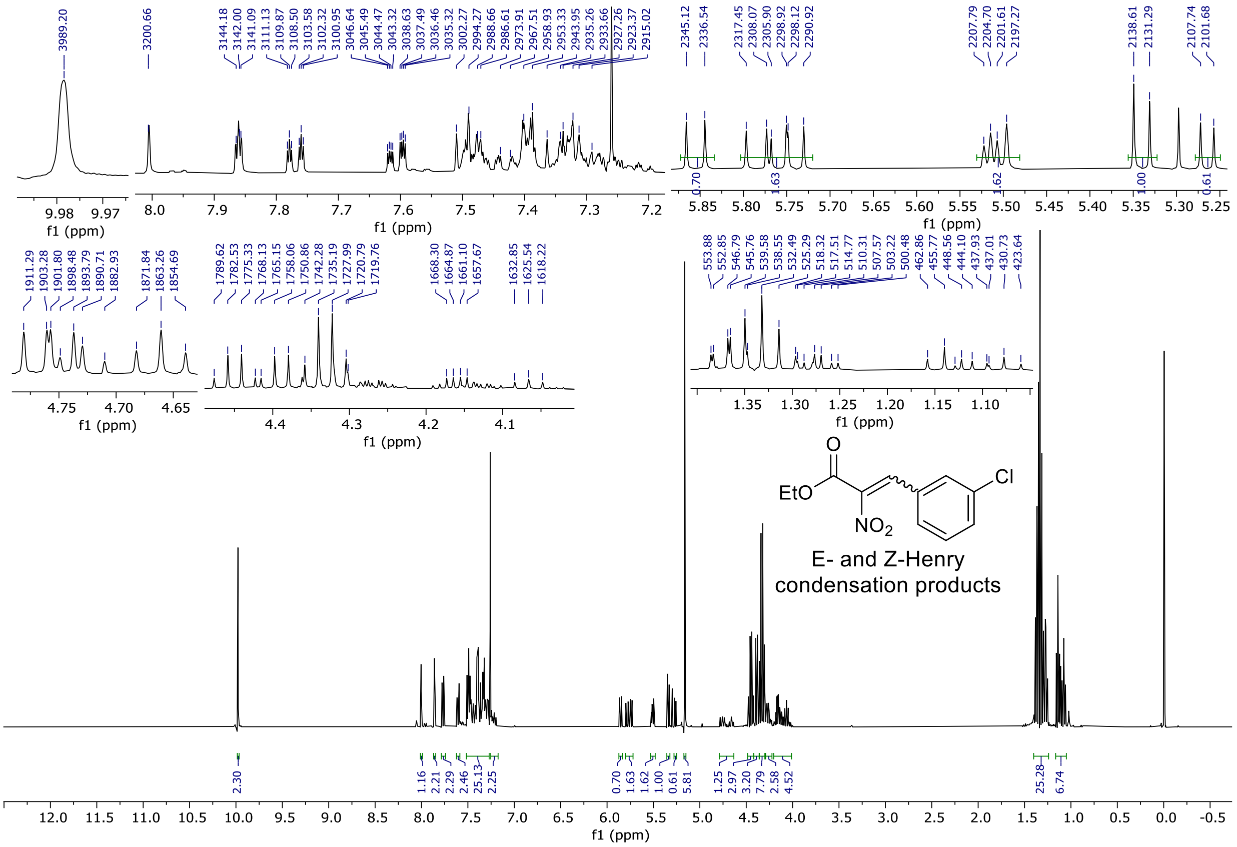

Figure S101. Crude ^{1}H NMR of (Henry) products after reaction of ethyl nitroacetate and 3-chlorobenzaldehyde, without cyclohexanone, and with 5 mol% of catalyst **1**.

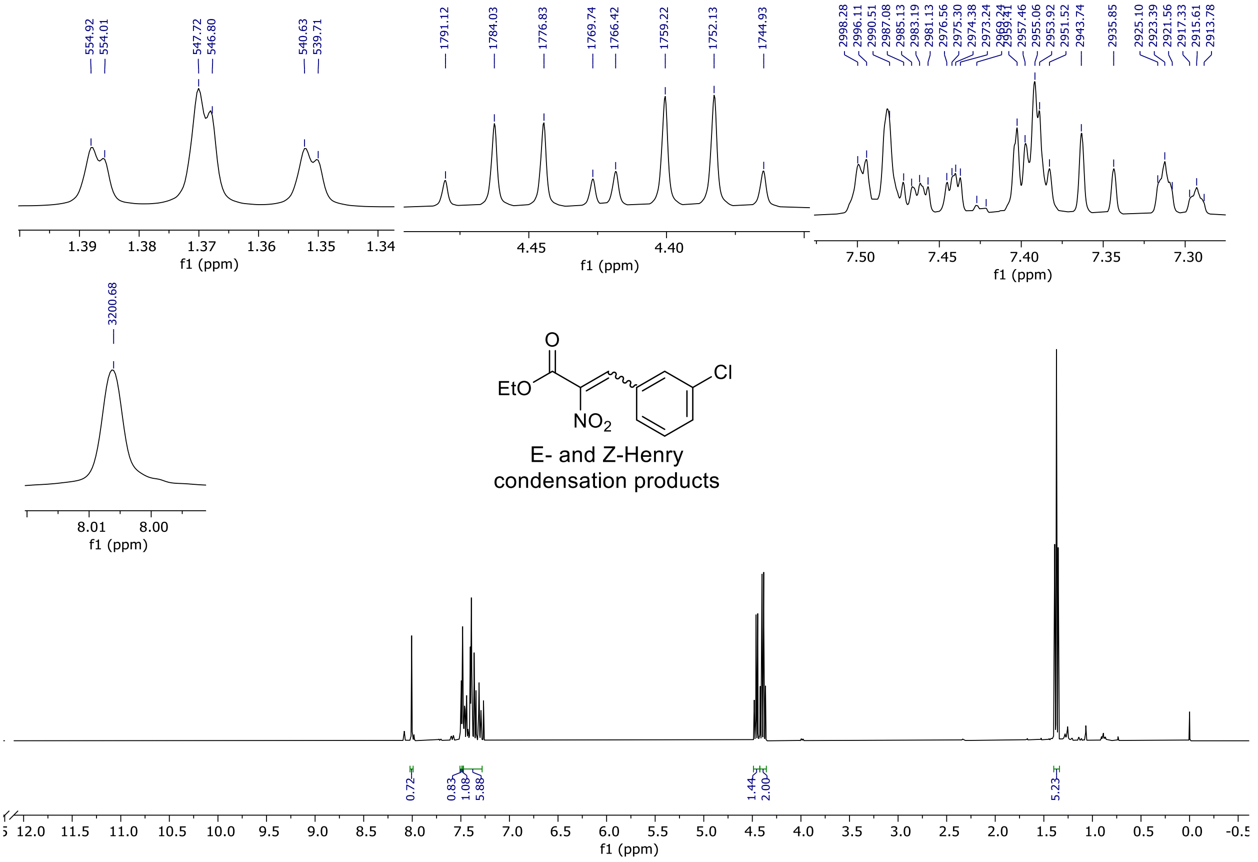

Figure S102. ^{1}H NMR of Henry condensation product isolated from the crude material noted in Figure S101.

Data File D:\DATA\NUGENT\AL24 RAC 5%IPANHEX 06FEB25 RE.D
Sample Name: AL24 RAC 5%IPAnHex 06feb25 re

```
=====================================================================
Acq. Operator   :                                   Seq. Line :   2
Acq. Instrument : Instrument 1                       Location : P1-A-03
Injection Date  : 2/6/2025 1:35:39 PM                     Inj :   1
                                                   Inj Volume : 20 µl
Acq. Method     : D:\Methods\Nugentlab_Patrick\AIDA.m
Last changed    : 2/6/2025 1:34:31 PM
Analysis Method : D:\Methods\Nugentlab_Patrick\AIDA254NM1.m
Last changed    : 2/6/2025 2:01:03 PM
                  (modified after loading)
```

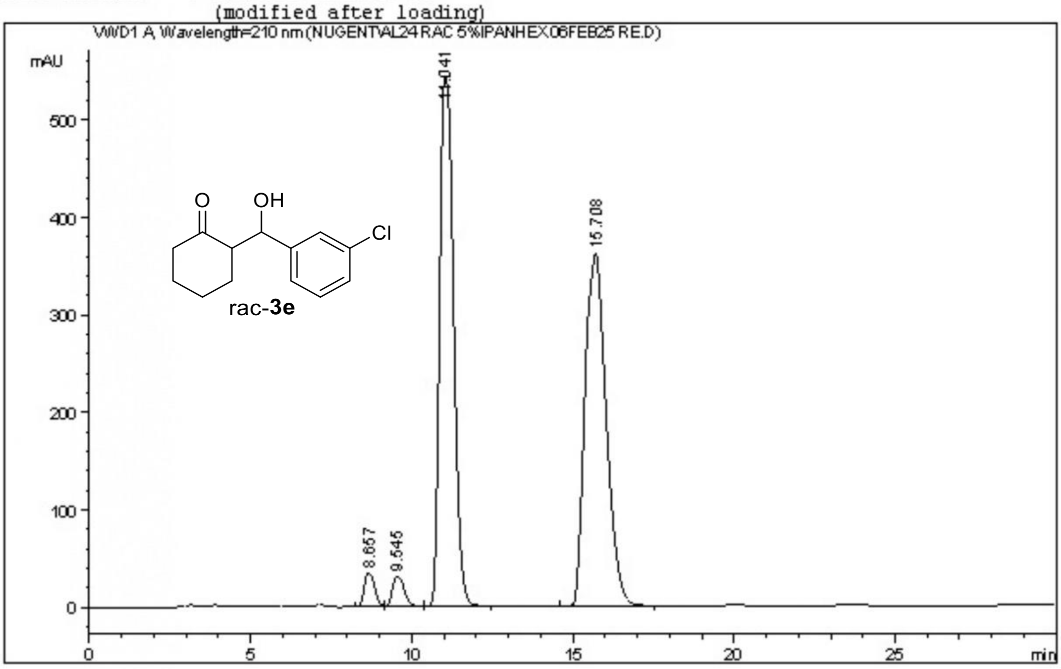


```
=====================================================================
                         Area Percent Report
=====================================================================

Sorted By             :      Signal
Multiplier            :      1.0000
Dilution              :      1.0000
Use Multiplier & Dilution Factor with ISTDs


Signal 1: VWD1 A, Wavelength=210 nm
```

Peak #	RetTime [min]	Type	Width [min]	Area mAU *s	Height [mAU]	Area %
1	8.657	BV	0.3643	785.40881	34.54132	2.2828
2	9.545	VB	0.4170	804.99719	30.80102	2.3397
3	11.041	BB	0.4747	1.62915e4	543.91724	47.3515
4	15.708	BB	0.7207	1.65235e4	361.77737	48.0260
Totals :				3.44054e4	971.03695	

```
=====================================================================
                        *** End of Report ***
```

Instrument 1 2/6/2025 2:06:54 PM

Page 1 of 1

Figure S103. HPLC chromatogram of the racemic *anti*-aldol product **3e** (above).

```
Data File D:\DATA\NUGENT\NS-II-53-ALDOL-PI 5% IPAHEX RERU31MAR25.D
Sample Name: NS-II-53-ALDOL-PI 5% IPAHEX RERU31MAR25
    ==================================================================
    Acq. Operator   :                              Seq. Line :   1
    Acq. Instrument : Instrument 1                  Location : P1-A-02
    Injection Date  : 3/31/2025 5:14:34 PM               Inj :   1
                                                  Inj Volume : 20 µl
    Different Inj Volume from Sequence !     Actual Inj Volume : 12 µl
    Acq. Method     : D:\Methods\Nugentlab_Patrick\AIDA 5IPA 210NM.m
    Last changed    : 3/31/2025 5:13:53 PM
    Analysis Method : D:\Methods\Nugentlab_Patrick\Aida 5IPA 210nm.m
    Last changed    : 2/5/2025 12:02:12 PM
    ==================================================================
```

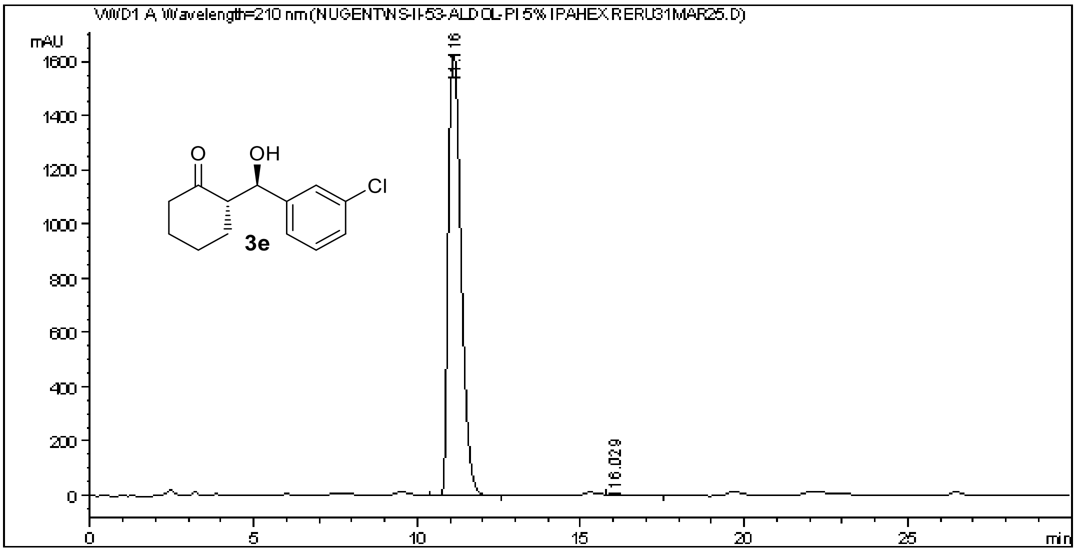


```
    ==================================================================
                             Area Percent Report
    ==================================================================

    Sorted By             :      Signal
    Multiplier            :      1.0000
    Dilution              :      1.0000
    Use Multiplier & Dilution Factor with ISTDs


    Signal 1: VWD1 A, Wavelength=210 nm

    Peak RetTime Type  Width     Area      Height     Area
      #   [min]        [min]  mAU   *s    [mAU  ]      %
    ----|-------|----|-------|----------|----------|--------|
       1  11.116 VB    0.4318 4.47051e4  1631.70605  99.3401
       2  16.029 VB    0.5256  296.98553    8.23500   0.6599

    Totals :                  4.50021e4  1639.94105


    ==================================================================
                             *** End of Report ***
```


Figure S104. HPLC chromatogram of enantioenriched *anti*-aldol (major) product **3e** (above).

Section 9. Ball-milling experiment: Table 3, entry 10

Table S2. Results of the competition reaction between cyclohexanone, (nitromethyl)-benzene and 4-nitrobenzaldehyde.[a]

**Entry**	Henry nucleophile	Aldehyde	Mixture	Chemo-selectivity Aldol:Henry[b]	Yield (%)[c]	dr (*anti*: *syn*)[d]	ee[e]
1	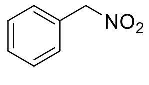 1.5 equiv.	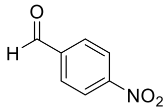	H_2O (15 eq.)	5:1	43	7.0:1	> 99

[a]Standard reaction conditions: 1.5 equiv. of cyclohexanone, 1.0 equiv. of aldehyde, 1.5 equiv. of nitroalkane, 2,5 mol% of catalyst and the corresponding solvent were added into a 2 mL plastic Eppendorf, along with three 3 mm stainless steel balls. The snap-cap Eppendorf was closed and the reaction was mixed in a ball mill (Retsch vibrating mill MM 200) for 9 h at a frequency of 16 Hz under air and r.t. conditions. During mixing, the shaking was periodically stopped every 90 min for 2–3 min to prevent excessive heating. Upon completion of the reaction, it was extracted with EtOAc (5 mL × 3) and water (10 mL), washed with brine (5 mL), dried over $MgSO_4$ and concentrated under reduced pressure to afford the crude reaction mixture. [b] Determined by ^1H NMR analysis of crude reaction mixtures. [c] Isolated yield of both *anti*- and *syn*- aldol products. [d] Diastereomeric ratio for the aldol reaction determined by ^1H NMR analysis of crude reaction mixtures. [e] Determined by chiral HPLC (Chiralcel OD-H column, *i*-PrOH/n-hexane (5:95), 1 mL /min).

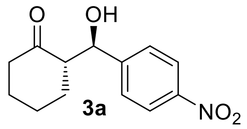

(*S*)-2-((*R*)-hydroxy(4-nitrophenyl)methyl)cyclohexan-1-one (**3a**):
To a clean plastic Eppendorf (2 mL) equipped with three 3 mm stainless steel balls, 4-nitrobenzaldehyde (MW = 151.12 g/mol, 1.00 equiv, 0.12 mmol, 18.0 mg), cyclohexanone (MW = 98.14 g/mol, 1.50 equiv, 0.18 mmol, 19.2 µL, density = 0.947 g/mL), and (nitromethyl)benzene (MW = 137.14 g/mol, 1.50 equiv, 0.18 mmol, 15 µL, density = 1.158 g/mL) and *trans*-4-(*tert*-butyldiphenylsilyloxy)-L-proline (MW = 369.54 g/mol, 2.50 mol%, 0.003 mmol, 1.1 mg) were added. The snap-cap Eppendorf was closed and the reaction was mixed in a ball mill (Retsch vibrating mill MM 200) for 9 h at a frequency of 16 Hz under air and r.t. conditions. During mixing, the shaking was periodically stopped every 90 min for 2–3 min to prevent excessive heating. Then, it was extracted with EtOAc (5 mL × 3) and water (10 mL), washed with brine (5 mL), dried over $MgSO_4$, filtered and concentrated under reduced pressure to afford the crude reaction mixture.

References for ball milling in organic synthesis
(i) Wang, G.-W. Mechanochemical Organic Synthesis. *Chem. Soc. Rev.* **2013**, *42* (18), 7668–7700.
(ii) Egorov, I. N.; Santra, S.; Kopchuk, D. S.; Markevich, A. V.; Gordeev, E. G.; Ananikov, V. P. Ball Milling: An Efficient and Green Approach for Asymmetric Organic Syntheses. *Green Chem.* **2020**, *22* (10), 3026–3050.

Purification and yield: Silica gel chromatography (10 mm column outer diameter, 10 cm silica bed height) was performed using gradient elution (10 to 25 vol% EtOAc in n-hexane). The crude product was loaded onto the column in a minimum volume of 50% EtOAc in n-hexane. A yellow solid weighing 12.9 mg (MW = 249.27 g/mol, 0.0517 mmol, 43% yield) of the *anti*- and *syn*-aldol diastereomers was isolated.
Conditions: *n*-Hexane:2-propanol 95:5, 1 mL /min, OD-H Column, , λ = 254 nm, injection volume = 20 µL, the sample was dissolved in *i*PrOH with a concentration of ≈ 1 mg/mL).
Racemic version of aldol reaction between cyclohexanone and 4-nitrobenzaldehyde
Enantiomers:
syn: 39.2 min, 44.7 min
anti: 50.2 min, 73.7 min

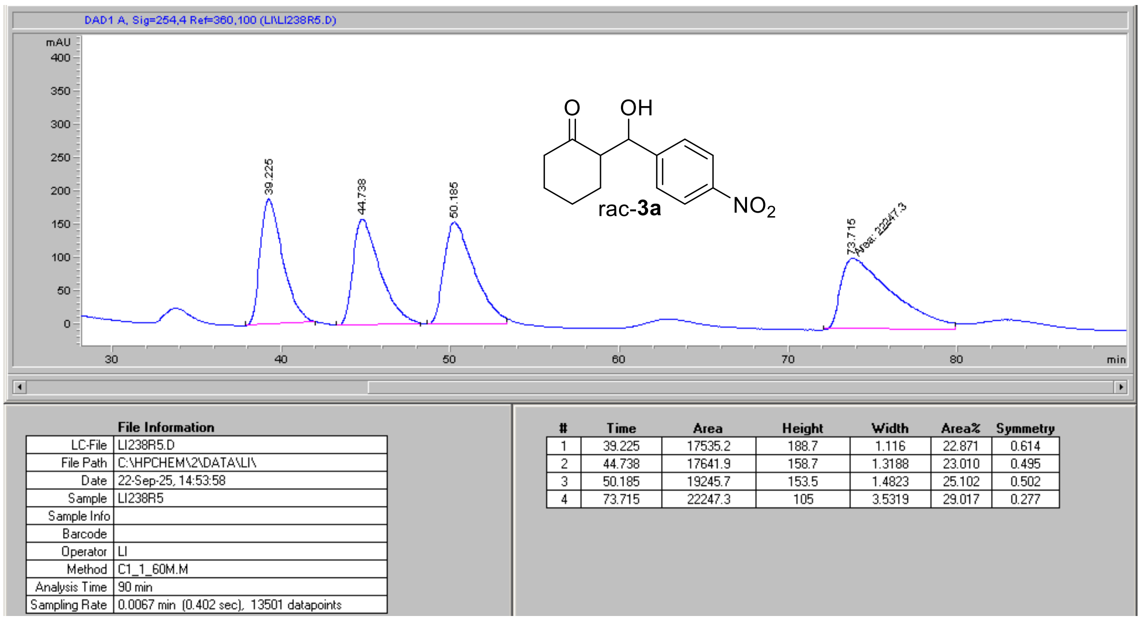

File Information

LC-File	LI238R5.D
File Path	C:\HPCHEM\2\DATA\LI\
Date	22-Sep-25, 14:53:58
Sample	LI238R5
Sample Info	
Barcode	
Operator	LI
Method	C1_1_60M.M
Analysis Time	90 min
Sampling Rate	0.0067 min (0.402 sec), 13501 datapoints

#	Time	Area	Height	Width	Area%	Symmetry
1	39.225	17535.2	188.7	1.116	22.871	0.614
2	44.738	17641.9	158.7	1.3188	23.010	0.495
3	50.185	19245.7	153.5	1.4823	25.102	0.502
4	73.715	22247.3	105	3.5319	29.017	0.277

Chiral

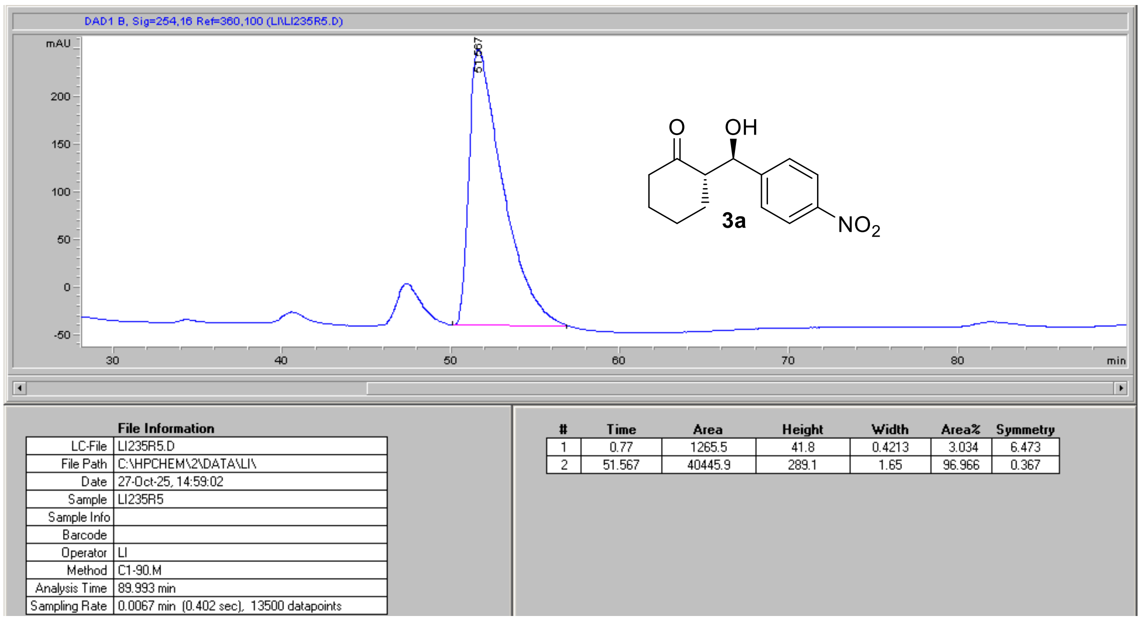

File Information

LC-File	LI235R5.D
File Path	C:\HPCHEM\2\DATA\LI\
Date	27-Oct-25, 14:59:02
Sample	LI235R5
Sample Info	
Barcode	
Operator	LI
Method	C1-90.M
Analysis Time	89.993 min
Sampling Rate	0.0067 min (0.402 sec), 13500 datapoints

#	Time	Area	Height	Width	Area%	Symmetry
1	0.77	1265.5	41.8	0.4213	3.034	6.473
2	51.567	40445.9	289.1	1.65	96.966	0.367

Section 10. Unidentified product: Table 4, entry 3

The ^{1}H and ^{13}C NMRs, MS, and HRMS (with a software chosen molecular formula) follow.

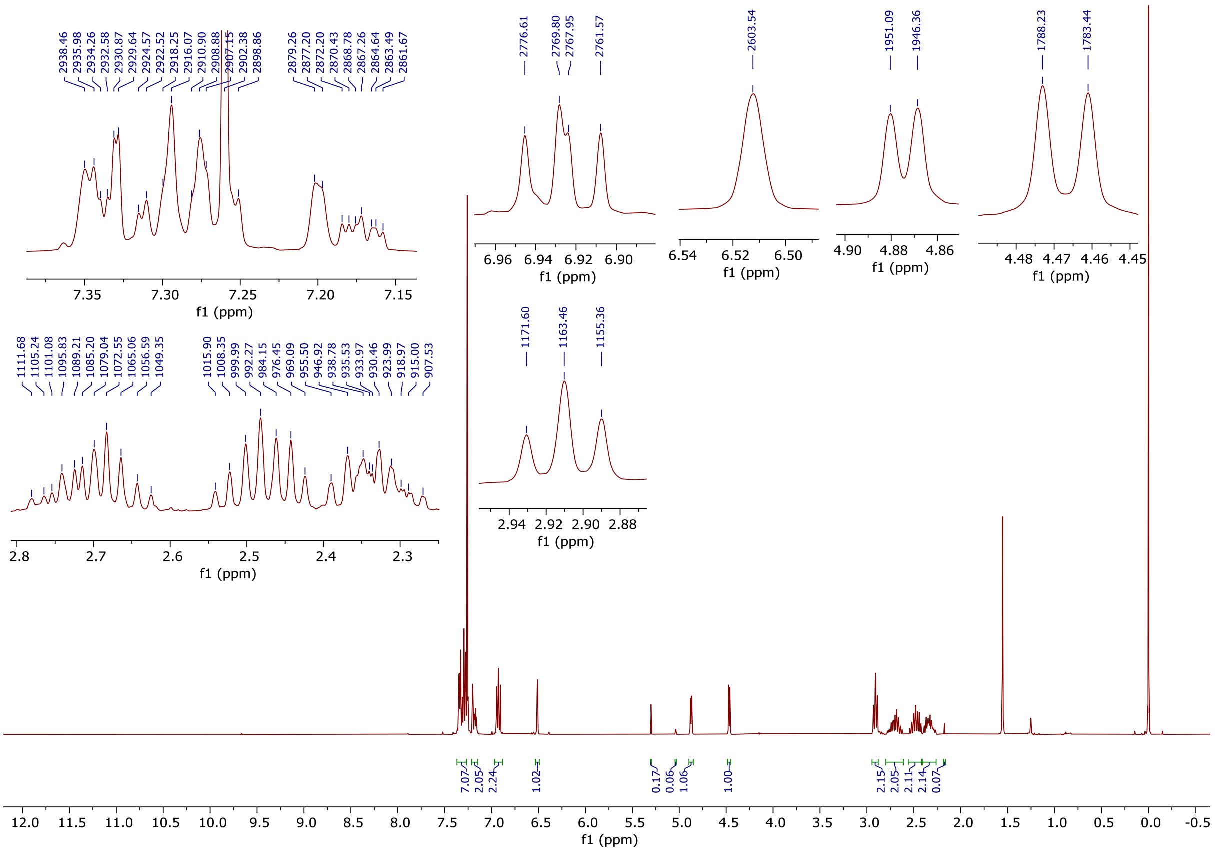

Figure S105. ^{1}H NMR of unidentified product in DMSO-d_6 (Table 4, entry 3).

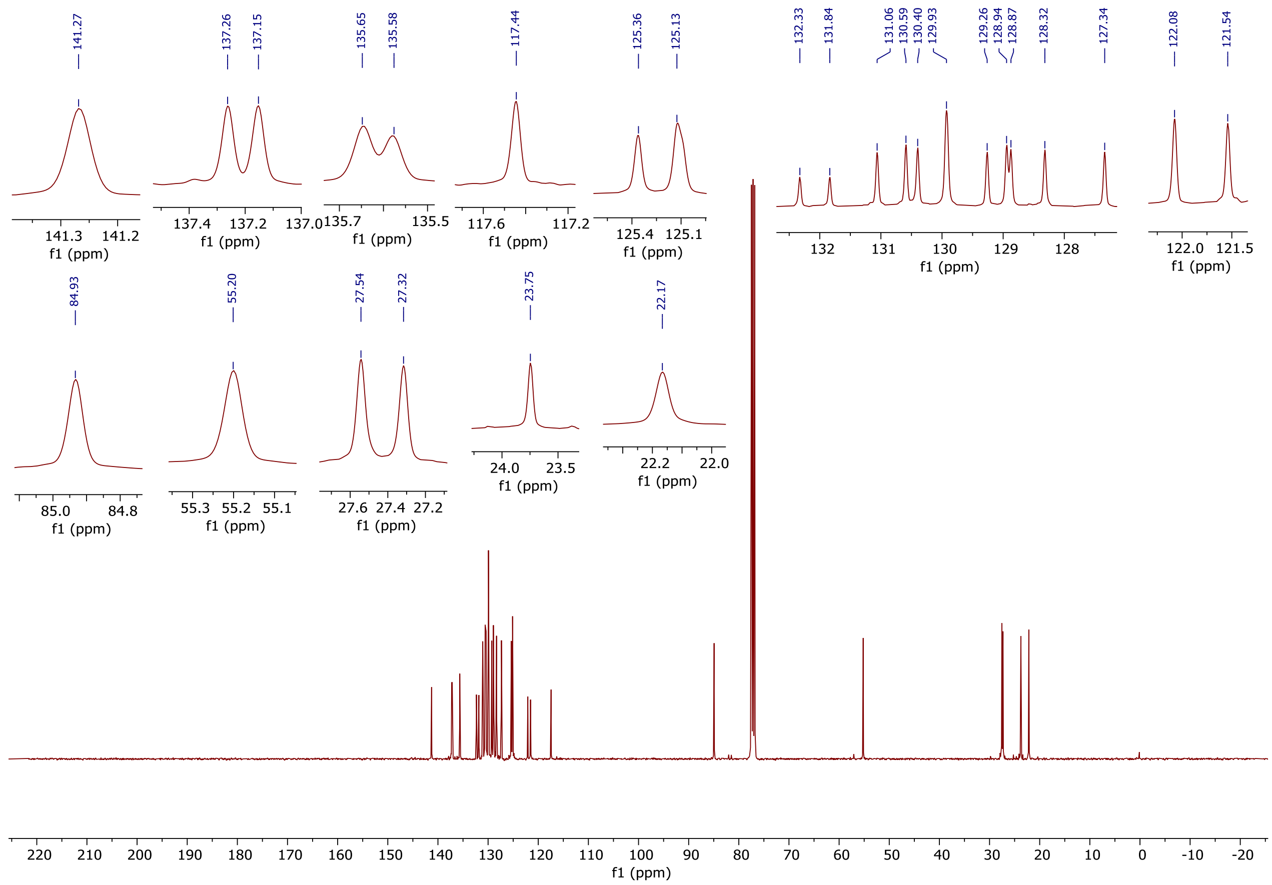

Figure S106. ^{13}C NMR of unidentified product in DMSO-d_6 (Table 4, entry 3).

Display Report

Analysis Info

Analysis Name	C:\Users\jhoelscher\OneDrive - Constructor University\Nugent\MS data\Kritika\Mar 25\KD-II-79-RL-purified_neg_10ppm.d	Acquisition Date	18.03.2025 13:12:07
Method	Tune_neg_Standard.m	Operator	BDAL@DE
Sample Name	KD-II-79-RL-purified_neg_10ppm	Instrument	impact HD 1819696.00180
Comment			

Acquisition Parameter

Source Type	ESI	Ion Polarity	Negative	Set Nebulizer	0.3 Bar
Focus	Not active	Set Capillary	4500 V	Set Dry Heater	200 °C
Scan Begin	50 m/z	Set End Plate Offset	-500 V	Set Dry Gas	4.0 l/min
Scan End	2000 m/z	Set Charging Voltage	2000 V	Set Divert Valve	Source
		Set Corona	0 nA	Set APCI Heater	0 °C

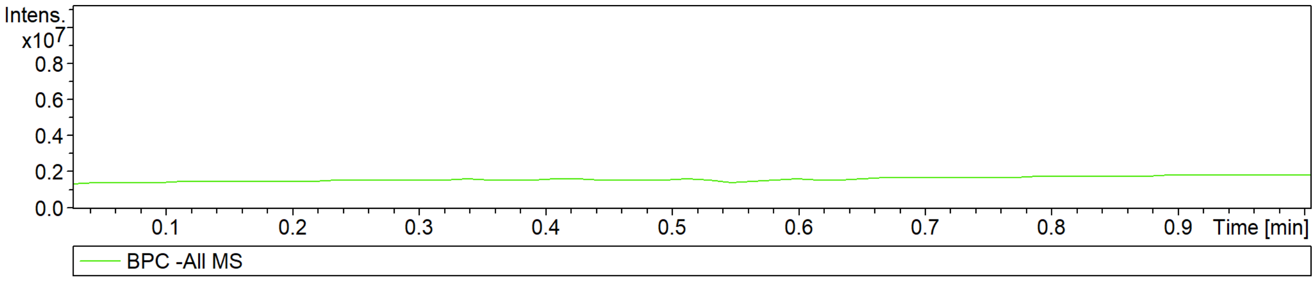

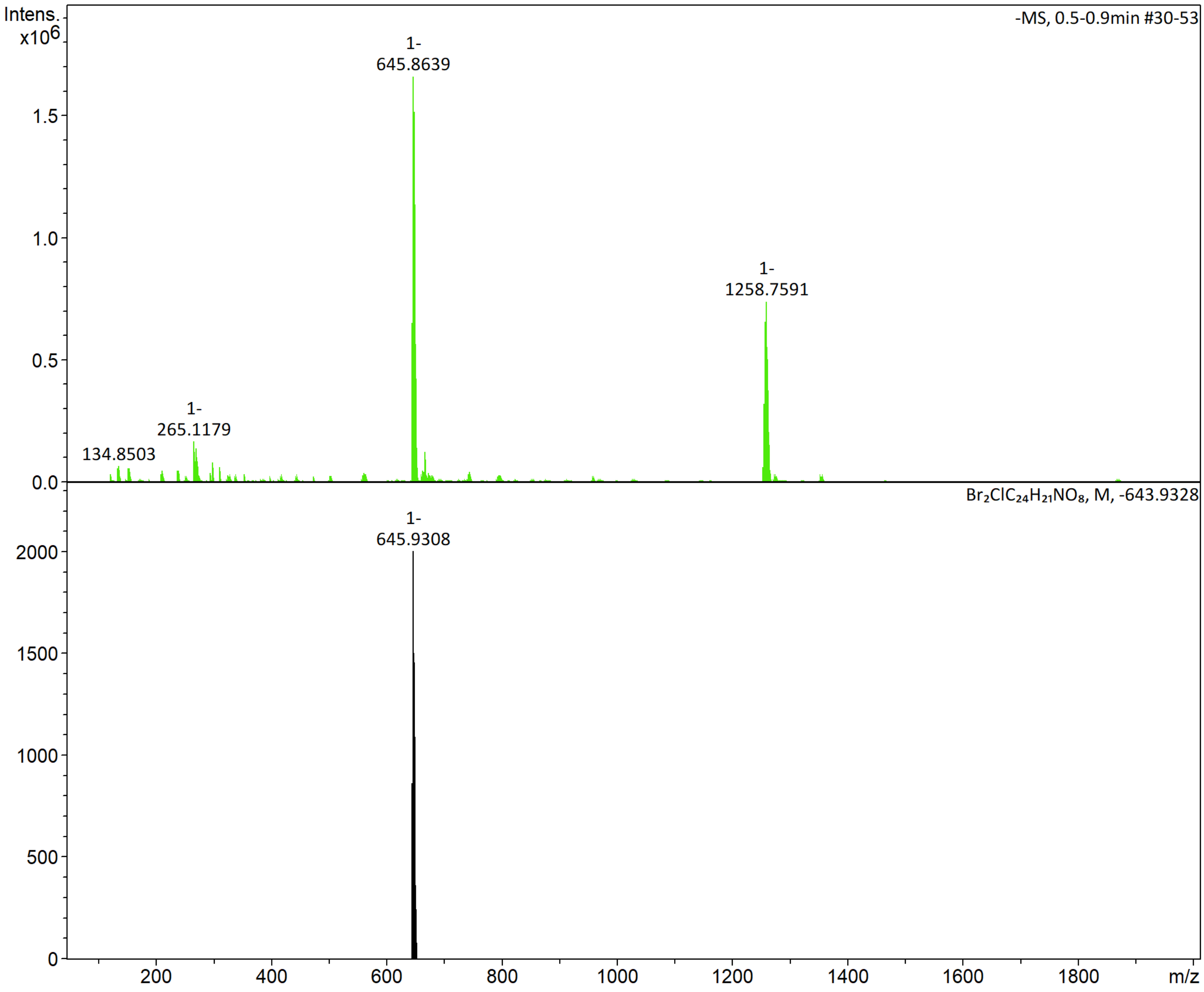

Figure S107. Mass spectrum of unidentified product.

Display Report

Analysis Info

Analysis Name	C:\Users\jhoelscher\OneDrive - Constructor University\Nugent\MS data\Kritika\Mar 25\KD-II-79-RL-purified_neg_10ppm.d	Acquisition Date	18.03.2025 13:12:07
Method	Tune_neg_Standard.m	Operator	BDAL@DE
Sample Name	KD-II-79-RL-purified_neg_10ppm	Instrument	impact HD 1819696.00180
Comment			

Acquisition Parameter

Source Type	ESI	Ion Polarity	Negative	Set Nebulizer	0.3 Bar
Focus	Not active	Set Capillary	4500 V	Set Dry Heater	200 °C
Scan Begin	50 m/z	Set End Plate Offset	-500 V	Set Dry Gas	4.0 l/min
Scan End	2000 m/z	Set Charging Voltage	2000 V	Set Divert Valve	Source
		Set Corona	0 nA	Set APCI Heater	0 °C

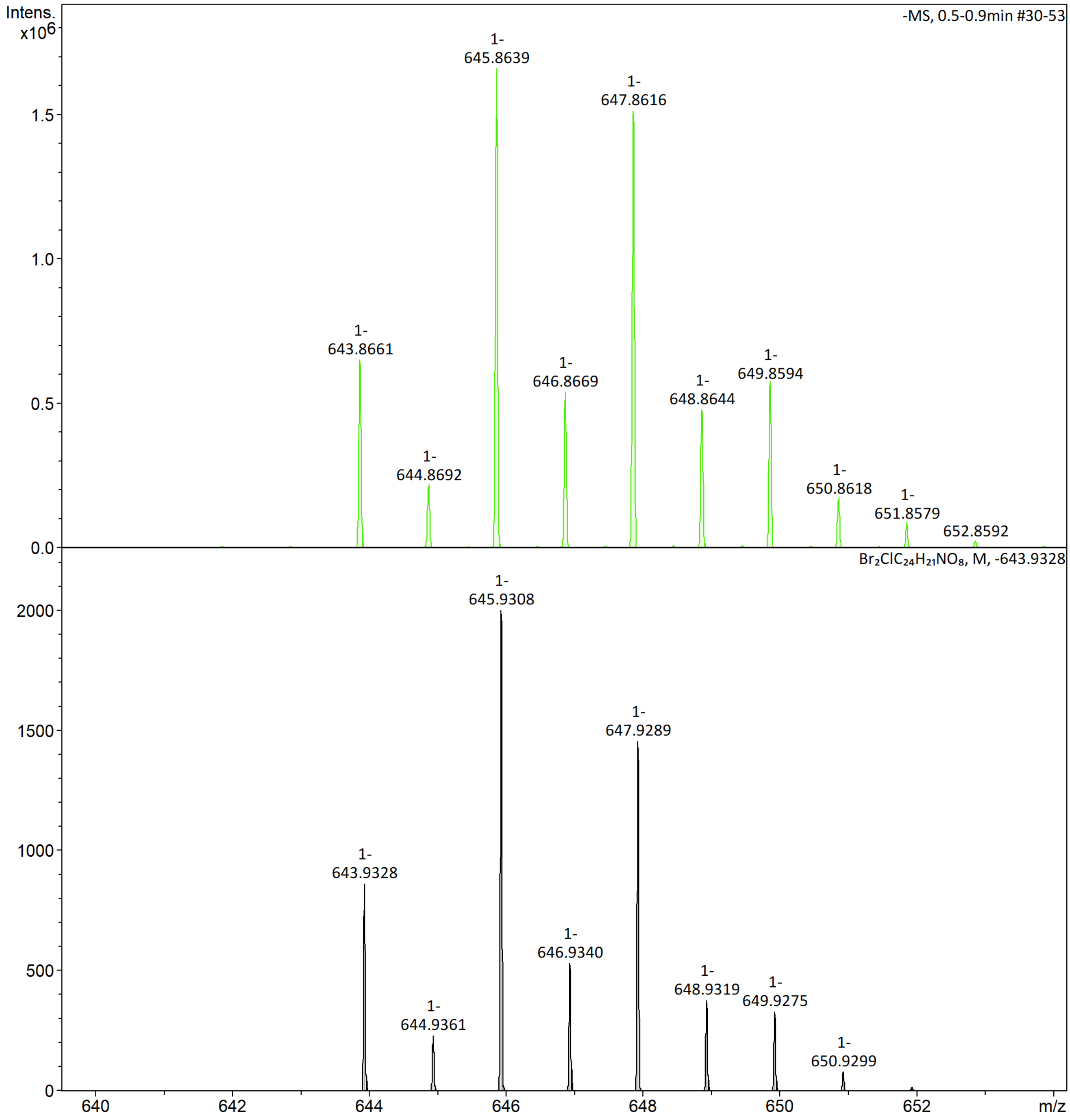

KD-II-79-RL-purified_neg_10ppm.d

Figure S108. High Resolution MS of unidentified product
